# Host–Guest Exchange of Viologen Guests in Porphyrin Cage Compounds as Studied by Selective Exchange Spectroscopy (1D EXSY) NMR

**DOI:** 10.1002/anie.202010335

**Published:** 2020-11-18

**Authors:** Anne Swartjes, Paul B. White, Marijn Lammertink, Johannes A. A. W. Elemans, Roeland J. M. Nolte

**Affiliations:** ^1^ Radboud University Institute for Molecules and Materials Heyendaalseweg 135 6525 AJ Nijmegen The Netherlands

**Keywords:** 1D EXSY NMR, host–guest exchange, kinetics, porphyrins cage, viologen guest

## Abstract

Dynamics in complexes of porphyrin cage compounds and viologen‐derived guest molecules are investigated by selective exchange NMR spectroscopy (1D EXSY). Exchange rates were found to be independent of excess guest concentration, revealing a dissociative exchange mechanism, which is accompanied by negative activation entropies, indicating significant reorganization of the host–guest complex during dissociation. Nonsymmetric viologen guests with bulky head groups had more unidirectional binding and slower exchange rates than guests with less‐bulky head groups. Thermodynamic and kinetic studies revealed that the exchange process is primarily driven by the thermodynamics of binding and that guest binding can be influenced by introducing steric and electronic groups on the host . Exchange studies with guests bearing a polymer chain revealed that both slippage and full dissociation takes place and the rate constants for both processes were determined. The slippage rate constant revealed that for smaller guests exchange takes place nearly exclusively under thermodynamic control.

## Graphical abstract

Dynamics in complexes of porphyrin cage compounds and viologen‐derived guest molecules are investigated by selective exchange NMR spectroscopy (1D EXSY). These studies explore the mechanism of host–guest exchange, how steric and electronic properties influence the rate of exchange, and whether the exchange process is governed by kinetic or thermodynamic factors.

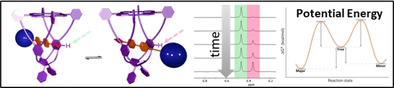

## Introduction

Since the advent of supramolecular chemistry, host‐guest complexes have been studied as models for receptor‐substrate interactions in nature and as mimics of the action of enzymes.^[1]^ Many different types of hosts and guests have been investigated, well‐known examples being crown ethers,[^2^, ^3^] cyclodextrins,[^4^, ^5^, ^6^] calixarenes,^[7]^ cucurbiturils,[^8^, ^9^] and pillararenes.^[10]^ In the past we have developed host compounds derived from the concave molecule glycoluril containing a porphyrin roof.^[11]^ We have shown that these porphyrin cages bind viologen guests very strongly (*K*_a_≈10^4^–10^6^ M^−1^).[^12^, ^13^] Furthermore, these cage compounds display cooperative binding behavior and can act as biomimetic catalysts in the epoxidation of low molecular weight and polymeric alkenes.^[14]^ More recently, we have started a program to write digital information onto single polymer chains in the form of chiral epoxides (*R*,*R*‐epoxide=digit 1, *S*,*S*‐epoxide=digit 2) with the help of chiral porphyrin cage compounds derived from glycoluril.^[15]^ As part of this project we are studying the threading and binding of viologen‐based guest molecules, which are blocked on one side with a bulky stopper, in these host molecules,[^16^, ^17^] and we have observed unidirectional binding depending on the type of viologen guest. These studies are a first important step in the design of porphyrin cage catalysts that can thread onto a polymer chain containing alkene double bonds and move along it, while converting the alkene functions into epoxides (digital printing process). To ensure smooth and processive writing, it is critical to investigate to what extent host‐guest exchange processes interfere with the epoxidation reaction and what factors govern the chemical exchange of the host and guest. Since the host‐guest exchange appears to be in the slow regime (0.1–2 s^−1^), NMR spectroscopy would be ideally suited to study this process.

**Figure 1:**
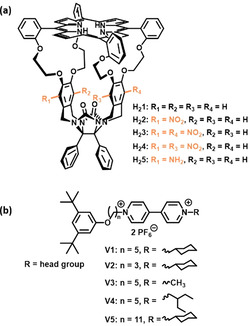
a) Chemical structures of the differently substituted porphyrin cage compounds. b) Chemical structure of the viologen‐derived guest compounds.

In this paper we describe our studies in this direction using selective exchange NMR spectroscopy, abbreviated 1D EXSY NMR. In the same decade as the first publication on two‐dimensional NMR in 1971, 2D EXSY NMR experiments have been performed with the first reports appearing in the late 1970s.[^18^, ^19^] 2D EXSY has been applied in the field of chemistry and biochemistry to study exchange between RNA conformers,^[20]^ to measure rotational barriers for *N*‐alkenyl amide bonds,^[21]^ to determine halogen exchange of phenylhalostannyl derivatives,^[22]^ and to study binding of small substrates to proteins (e.g. γ‐picoline binding to cytochrome C).^[23]^ 2D EXSY experiments are quite time‐consuming and could be replaced later by more time‐efficient 1D EXSY studies,[^24^, ^25^] wherein a signal of interest is selectively irradiated and followed over time by varying the mix times. 1D EXSY NMR can be more complicated than 2D EXSY NMR since signals of interest need to be isolated, which makes it less applicable for more complex systems. Nevertheless, the 1D EXSY technique can be used to study a variety of problems, such as dynamics in paramagnetic materials,^[26]^ interconversion between acid‐catalyzed diastereomers,^[27]^ and host‐guest complexation and exchange.[^28^, ^29^] The latter will be our focus of interest. As host compounds for our studies we selected the parent porphyrin cage compound **H_2_1** and a series of chiral and achiral derivatives of it (**H_2_2**‐**H_2_5**, see Figure 1 a). The guest molecules contain a viologen binding site, which is open at one end and capped at the other end with a 3,5‐di‐*tert*‐butyl‐1‐alkoxy moiety (Figure 1 b) to ensure that binding begins only from one side of the guest.

## Results and Discussion

In 2018 our group reported a study on the unidirectional binding of viologen‐derived guests to porphyrin cage compounds, and chemical exchange was observed from the 2D ROESY spectrum (Figure SI22).^[16]^ Since exchange is pertinent to processive catalysis, we undertook 1D EXSY studies to investigate the factors that govern chemical exchange. For the host‐guest complex between mono‐nitrated porphyrin cage compound **H_2_2** and a viologen guest with a methyl cyclohexyl head group (**V1**), the 1D ^1^H NMR spectrum revealed the presence of two different species in different ratios. Based on the integration of the guest signals relative to the host signals, the strongly‐shielded chemical shifts of the bound viologens in each of the complexes, and the ROESY cross peaks from sets of sidewall protons to the viologen protons, it was evident that each species represents a complex in which the guest has a directionally‐unique orientation within the host rather than a complex in which the guest is rotationally or positionally fixed (see Supporting Information pages 109–110). This is because **V1** is an nonsymmetrically substituted viologen, which can adopt two different directional orientations upon binding in the cavity of **H_2_2**. Furthermore, the two portals of the porphyrin cage compound are inequivalent, resulting in significant chemical shift differences for the protons on the xylylene sidewalls. The 2D ROESY ^1^H NMR spectrum shows chemical exchange of these xylylene protons between the two isomeric species, and therefore prompted follow‐up 1D EXSY experiments to characterize this exchange.

### 1D EXSY studies

**Figure 2:**
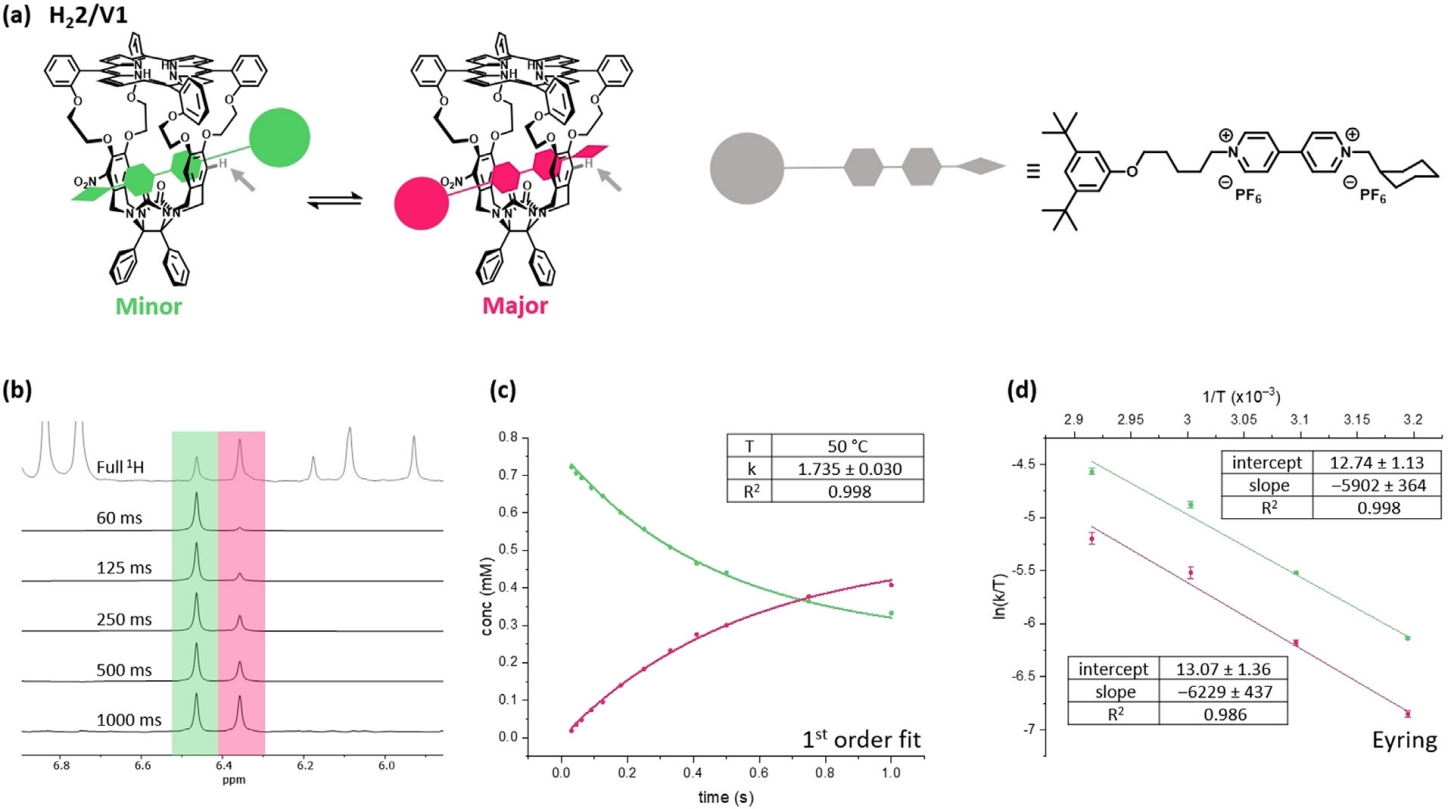
a) Scheme of the equilibrium between the two orientations of **V1** in the cavity of **H_2_2**; indicated by the grey arrow is the proton selected for the 1D NOESY measurements; b) Stacked 1D NOESY spectra of the **H_2_2/V1** complex in which the mix times increase (70 °C, 3:1 guest:host molar ratio, in chloroform‐*d*: [D_3_]acetonitrile, 1:1 (v/v)); c) Concentration of minor species (conc_*t*=0_=0.802 (mM)) plotted versus time (s) and the first order approaching equilibrium fit of the data to give the rate constant *k*; d) Eyring plots obtained from the variable temperature experiments.

In a first series of experiments we investigated the exchange processes occurring between the two complexes of **H_2_2** with **V1** in more detail. With the help of 1D EXSY NMR we were able to follow the change in the orientation of **H_2_2** during the chemical exchange process. Here, we selected the xylylene sidewall proton of **H_2_2** at 6.48 ppm (indicated by the grey arrow in Figure 2 a) and measured the exchange by incrementally increasing the mix times and integrating its signal (Figure 2 b). The rate constants could be calculated from fitting the change in concentration versus mix time to a first order reaction approaching equilibrium (Figure 2 c). The 1D EXSY studies were repeated between 40 and 70 °C with 10 °C increments. At every temperature point, a quantitative ^1^H NMR spectrum using pentafluorobenzaldehyde as an internal standard was recorded to accurately calculate the concentrations of the major and minor species. From the resulting Eyring plot (Figure 2 d), the activation enthalpies and entropies for the exchange of the minor to the major species (*k*_min_) as well as for the exchange of the major to the minor species (*k*_maj_) could be calculated. These resulted in the activation enthalpies Δ*H* ^≠^_maj_=12.53±0.88 kcal mol^−1^ and Δ*H* ^≠^_min_=11.87±0.73 kcal mol^−1^, and the activation entropies Δ*S* ^≠^_maj_=−21.23±1.84 cal K^−1^ mol^−1^ and Δ*S* ^≠^_min_=−21.90±1.95 cal K^−1^ mol^−1^, which indicates that there is not a significant difference in activation parameters to reach the transition state when going from the major to the minor species or vice versa.

### Mechanism of the exchange process

We found that for the above and all subsequently investigated systems the activation entropy was negative. For many systems in which host‐guest exchange occurs (involving hemicarcerands, cucurbiturils, and metal‐ligand frameworks) it has been reported that the activation entropy is slightly negative or close to zero for associative processes, and positive for dissociative processes.[^30^, ^31^, ^32^, ^33^, ^34^, ^35^, ^36^] For our host‐guest systems this would tentatively indicate that exchange occurs through an associative mechanism.

To determine whether the exchange indeed proceeds via such an associative mechanism or rather a dissociative pathway, the initial rates of the process were measured in the presence of different concentrations of guest. In an associative process, a higher exchange rate can be expected at a higher guest concentration, whereas in the case of a dissociative mechanism, we expect no dependence on the concentration of the guest.

We studied the exchange of the complex between the unsubstituted, symmetric host **H_2_1** and guest **V1** using three different concentrations of guest (4.3 mM, 16 mM, and 26 mM), while [**H_2_1**] was kept constant at 2 mM.

The initial rates were obtained from 1D EXSY studies and the order of the reaction was determined from the slope of a log(rate) versus log([**V1**]) plot (Table 1). This slope was 0.1386±0.0618, that is, very close to zero, which means that the exchange follows zero‐order kinetics in **V1** and is thus independent of [**V1**]. This indicates that the exchange process follows a dissociative mechanism. This result appears contradictory to the trend for earlier mentioned host‐guest complexes. However, other factors may also contribute to a negative activation entropy, such as solvent reorganization and re‐solvation of the host and guest upon dissociation and association. Solvent coordination to the porphyrin host could offer an explanation for the unfavorable dissociation entropy. In earlier X‐ray diffraction experiments we have seen that chloroform binds inside the cavity.^[12]^ Neutron diffraction studies from Salzmann et al.^[37]^ showed that liquid chloroform displays a high level of organization and therefore challenges the perception of chloroform as an unstructured solvent.^[38]^ Further studies on the entropy of binding could help offer more insight in the origin of the entropy of the exchange process for our studied systems. However, this is beyond the scope of this paper.

**Table 1:** Exchange rates for the complex between **H_2_1** and **V1** at different guest concentrations, determined by 1D EXSY NMR in [D_3_]acetonitrile: chloroform‐*d* (1:1, v/v) at 298 K.

[V1]:[H_2_1]	[V1] [mM]	Log([V1])	Log(rate)
2.6:1	4.3	−3.37	−3.32±0.02
9.6:1	16	−1.80	−3.22±0.01
15.2:1	26	−1.59	−3.22±0.02

### Determination of association constants (*K*_a_)

To determine whether the thermodynamic properties of the host/guest system of **H_2_2** and **V1** are in line with the kinetic information obtained from the exchange studies, fluorescence‐quenching host‐guest titrations were performed. From these measurements, information about the thermodynamics of host‐guest complex formation can be obtained and later compared to the kinetic data, allowing comparisons between the thermodynamic and kinetic factors in the exchange reaction to be made.

When a solution of a porphyrin cage compound is irradiated at a wavelength of 419 nm, the porphyrin gives rise to two fluorescence bands at 650 and 716 nm. These bands are subsequently quenched when a viologen‐derived guest binds inside the host, and through titration, the binding constants and the related Δ*G* ^⊖^_bind_ values can be determined. For the **H_2_2**/**V1** complex, the binding constants were determined to be *K*_a,min_=6.42×10^5^ M^−1^ and *K*_a,maj_=1.24×10^6^ M^−1^ (see Figure SI23 for calculation). The lower binding constant for the minor complex coincides with the lower activation enthalpy for the dissociation of the minor complex (Δ*H* ^≠^_min_=11.87±0.73 kcal mol^−1^), compared to that of the major counterpart (Δ*H* ^≠^_maj_=12.53±0.88 kcal mol^−1^). This indicates that thermodynamic factors play a role in the chemical exchange in these systems.

### Influence of host structure on exchange

**Figure 3:**
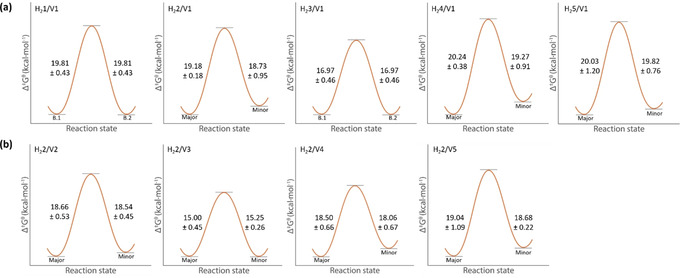
Schematic representation of the activation Gibbs free energies (Δ*G* ^≠^ in kcal mol^−1^) for different host–guest systems. B.1 and B.2 denote the Gibb's free energies of the two different guest orientations for which the system shows no preference as in the case of **H_2_1** and **H_2_3**. Major and minor indicate the major and minor abundant host–guest complex species. a) Δ*G* ^≠^ values of complexes of differently substituted hosts with **V1**, b) Δ*G* ^≠^ values of the complexes of substituted viologen‐derived guest molecules **V2**–**V5** with **H_2_2**. The diagrams focus on the kinetic factors and visualize the relative energy difference for complexes of differently substituted hosts between the ground state of the major abundant complex and the transition state and that of the minor abundant complex and the transition state. The ground states of the two possible complexes are not to scale.

Ongoing studies in our group to enantioselectively epoxidize polymer chains utilize porphyrin cages that are nonsymmetrically functionalized at their sidewalls. For this reason, we expanded the scope of host‐guest complexes and investigated the influence of unsubstituted, mono‐ and di‐substituted side walls of the porphyrin cage compounds (**H_2_1**‐**H_2_5**, Figure 1) on the exchange of **V1**. All studied hosts were racemic mixtures of enantiomers. Similar to **H_2_2**, when **V1** is bound inside the cavity of **H_2_4** or **H_2_5**, two different host‐guest complexes are formed in different abundances due to the nonsymmetrical nature of both the host and guest. NMR studies showed that for each of these host‐guest systems the major complex is the one in which the C_5_‐linker connected to the blocking group is located at the nitro‐ or amine‐substituted cavity portal. After having determined the rate constants of exchange at different temperatures, the activation Gibbs free energies (Δ*G* ^≠^) could be extracted from the Eyring plots. These energies are visualized in Figure 3 a and compiled in Table 2. The complexes of **V1** with the bis‐nitrated host compounds comprise both the lowest and the highest activation free Gibbs energy barriers, viz. Δ*G* ^≠^=16.97±0.46 kcal mol^−1^ for the *anti*‐facial bis‐nitrated host **H_2_3** and Δ*G* ^≠^=20.24±0.38 kcal mol^−1^ for the *syn*‐facial bis‐nitrated host **H_2_4**. Remarkably, the highest degree of unidirectional binding of the guest (ratio of major to minor=4:1) was observed for the complex between **H_2_4** and **V1**. All determined activation free energies are well within the ranges reported for other supramolecular systems in which small guests exchange.[^30^, ^39^, ^40^, ^41^, ^42^] The fact that the complex between **H_2_3** and **V1** has the lowest energy barrier for exchange (Δ*G* ^≠^=16.97±0.46 kcal mol^−1^) of all complexes may be explained by the required passage of a cation across an additional nitro‐plane. Since **H_2_3** has two nitro functionalities in an *anti*‐facial manner, both sides of the host destabilize the binding of **V1**, resulting in the weakest binding of the guest inside the cavity of the measured systems (*K*_a_=4.5×10^4^ M^−1^, Table SI4), which coincides with a smaller energy barrier for dissociation and exchange.

**Table 2:** Activation enthalpies, entropies, and Gibbs free energies (at 40 °C) for the exchange between **V1** and various hosts.^[a]^

Host (Guest: V1)	Δ*H* ^≠^ [kcal mol^−1^]	Δ*S* ^≠^ [cal K^−1^ mol^−1^]	Δ*G* ^≠^ [kcal mol^−1^]	*K*_maj/min_
H_2_1	13.36±0.29	−20.67±0.85	19.81±0.43	1.0
H_2_2 (*k*_maj_)	12.53±0.88	−21.23±1.84	19.18±1.05	2.0
H_2_2 (*k*_min_)	11.87±0.73	−21.90±1.95	18.73±0.95	
H_2_3	10.30±0.41	−21.28±0.69	16.97±0.46	1.0
H_2_4 (*k*_maj_)	15.97±0.37	−13.65±0.24	20.24±0.38	4.2
H_2_4 (*k*_min_)	14.38±0.20	−15.62±0.29	19.27±0.91	
H_2_5 (*k*_maj_)	10.53±0.96	−30.35±2.29	20.03±1.20	1.5
H_2_5 (*k*_min_)	10.85±0.44	−28.67±1.97	19.82±0.76	

[a] *k*_maj_ and *k*_min_ indicate the exchange rate constants for the major and minor abundant complexes, respectively. *K*_maj/min_ is the ratio between the major and minor abundant complex at equilibrium.

For comparison, the unsubstituted host **H_2_1** does not display these steric and electronic effects, making that the guest binds stronger (*K*_a_=6.0×10^5^ M^−1^, Table SI4), and the activation Gibbs free energy is significantly higher (Δ*G* ^≠^=19.81±0.43 kcal mol^−1^). The exchange of **V1** in mono‐nitrated host **H_2_2** shows activation barriers (Δ*G* ^≠^_maj_=19.18±1.05 kcal mol^−1^, Δ*G* ^≠^_min_=18.73±0.95 kcal mol^−1^) that are in between the values observed for guest exchange in **H_2_3** and **H_2_1**. This observation suggests that the steric and electronic effects imposed by the nitro groups influence the Δ*G* ^≠^ of exchange. The *syn*‐facial bis‐nitrated host **H_2_4** showed the highest activation Gibbs free energy barrier for exchange with **V1**. In this host, the two nitro groups are located at one of the cavity portals, which likely complicates dissociation of the guest via that side of the cavity. The two nitro‐groups may “pinch” the guest while it is bound and therefore increase the activation energy for exchange. Preliminary molecular modeling calculations using the Spartan program (Figures SI26 and SI27) supports this hypothesis.

For further comparison, differences in the activation enthalpy and entropy values were investigated and compiled (Table 2). The activation enthalpy of exchange for the complex between **H_2_4** and **V1** is significantly larger than the activation enthalpies for the other nitro‐containing hosts, which is in line with the hypothesis of **H_2_4** pinching the guest, resulting in an increase of the activation barrier for its dissociation. Interestingly, despite displaying the highest activation enthalpies, the activation entropies for this host‐guest system (Δ*S* ^≠^_maj_=−13.65±0.24 cal K^−1^ mol^−1^, Δ*S* ^≠^_min_=−15.62±0.29 cal K^−1^ mol^−1^) are significantly lower than the activation entropies for the other systems. We explain this observation by a gain in flexibility of the guest during its dissociation, in particular of the C_5_‐linker, which is close to the nitro groups.

Remarkably, for complex **H_2_5/V1** the activation Gibbs free energy values are slightly higher than those of **H_2_2/V1**. We explain this increase by the electron‐donating nature of the amino‐group, which can strengthen the π‐π stacking interactions between the more electron‐rich cavity sidewall and the electron‐poor 4,4′‐bipyridinium moiety of **V1**, thereby increasing the energy barriers for exchange.

### Influence of guest structure on exchange

In previous studies we demonstrated that the size of the group at the open end of the viologen moiety strongly affects the unidirectionality of guest binding in the porphyrin cage, with larger groups yielding a larger population difference between complexes with different directionality.^[16]^ With this in mind, we further explored how steric factors impact the exchange process, that is, by varying the viologen end‐group as well as the length of the linker between the viologen and the blocking group (guests **V1**–**V5**, see Figure 5). The rationale for shortening the linker length is to create a steric competition between the blocker and head group, as a shorter linker moves the blocker group closer to the host. The kinetic parameters for the exchange processes were determined and the results are shown in Table 3. The Gibbs free energies of activation for the guest exchange in the complexes with **H_2_2** indicate that shortening the linker between the blocking group and the viologen moiety leads to a significant decrease in unidirectionality of the binding, compare *K*_maj/min_=1.2 for **V2** (*n*=3) with *K*_maj/min_=2 for **V1** (*n*=5) (Table 3). This confirms the hypothesis that a more closely positioned blocker group to the viologen‐moiety results in a decrease in population difference. The guest destabilizes the host/guest complex, as follows from the binding constants, **V2**: *K*_bind,maj_=4.21×10^5^±2.04×10^4^ M^−1^, *K*_bind,min_=3.51×10^5^±1.70×10^4^ M^−1^ (see Table SI4); **V1**: *K*_bind,maj_=1.24×10^6^±4.22×10^4^ M^−1^, *K*_bind,min_=6.42×10^5^±2.18×10^4^ M^−1^ (see Table SI4). This also coincided with lower Gibbs free energies of activation of **V2** exchanging in **H_2_2** (Δ*G* ^≠^_maj_=18.66±0.53 kcal mol^−1^, Δ*G* ^≠^_min_=18.54±0.45 kcal mol^−1^) compared to the Gibbs free energy of activation for the exchange of **V1** in **H_2_2** (Δ*G* ^≠^_maj_=19.18±1.05 kcal mol^−1^, Δ*G* ^≠^_min_=18.73±0.95 kcal mol^−1^). In contrast, **V5**, which contains a C_11_‐linker displays overall similar energy barriers for dissociation as **V1** (*n*=5), indicating that after a linker length of C_5_ there is no significant impact on the dissociation process. Furthermore, fluorescence binding studies show that the thermodynamics of binding of **V5** and **V1** in **H_2_2** do not differ significantly (**V5**: *K*_bind,maj_=1.40×10^6^±3.56×10^4^ M^−1^, *K*_bind,min_=7.02×10^5^±1.78×10^4^ M^−1^.

**Table 3:** Activation enthalpies, entropies and Gibbs free energies (at 40 °C) for the exchange between **H_2_2** and various guests.^[a]^

Host H_2_2	Δ*H* ^≠^ [kcal mol^−1^]	Δ*S* ^≠^ [cal K^−1^ mol^−1^]	Δ*G* ^≠^ [kcal mol^−1^]	*K*_maj/min_
V1 (*k*_maj_)	12.53±0.88	−21.23±1.84	19.18±1.05	2.0
V1 (*k*_min_)	11.87±0.73	−21.91±1.95	18.73±0.95	
V2 (*k*_maj_)	10.51±0.39	−26.03±1.14	18.66±0.53	1.2
V2 (*k*_min_)	10.69±0.31	−25.07±1.05	18.54±0.45	
V3 (*k*_maj_)	9.61±0.21	−18.03±0.51	15.25±0.26	2.0
V3 (*k*_min_)	8.87±0.41	−19.57±0.58	15.00±0.45	
V4 (*k*_maj_)	10.96±0.40	−24.06±1.08	18.50±0.52	1.9
V4 (*k*_min_)	9.98±0.34	−25.79±1.25	18.06±0.52	
V5 (*k*_maj_)	11.96±1.08	−22.60±0.50	19.04±1.09	2.0
V5 (*k*_min_)	12.68±0.18	−19.16±0.37	18.68±0.22	

[a] *k*_maj_ and *k*_min_ indicate the exchange rate constants for the major and minor abundant complexes, respectively. *K*_maj/min_ is the ratio between the major and minor abundant complex at equilibrium.

For the combination **H_2_2/V1** the activation Gibbs free energy is higher for the conversion of the major to the minor abundant complex (Δ*G* ^≠^_maj_=19.18±1.05 kcal mol^−1^) than that for the reverse conversion (Δ*G* ^≠^_min_=18.73±0.95 kcal mol^−1^). This trend is also present in the systems **H_2_4/V1** and **H_2_5/V1**. We attribute this difference to the lower ground state energy of the major species, as opposed to the minor species, which will be further discussed below (vide infra). We also investigated a guest with a methyl instead of a methylcyclohexyl group (**V3**, Figure 3 b) to reduce the steric hindrance of the group at the open side. As expected, the activation Gibbs free energy values for the combination **H_2_2/V3** were lower (Δ*G* ^≠^_maj_=15.25±0.26 kcal mol^−1^, Δ*G* ^≠^_min_=15.00±0.45 kcal mol^−1^). However, the Gibbs free energy difference between major and minor species (ΔΔ*G* ^≠^_maj‐min_) showed no significant difference for **H_2_2/V1** and **H_2_2/V3**, which shows that a higher energy barrier for the major to the minor species can be found than vice versa.

The energy barrier for the exchange of **V3** in **H_2_2** is significantly lower than that of the guest with the methylcyclohexyl end‐group (**V1**). Going from methylcyclohexyl to methyl significantly decreases the bulkiness of the guest, which provides it with more flexibility to dissociate from the cavity of the host. The system might have gained more flexibility as indicated by the small difference in entropy cost for the exchange of **V3** (Δ*S* ^≠^_maj_=−19.57±0.58 cal K^−1^ mol^−1^) compared to that of **V1** (Δ*S* ^≠^_maj_=−21.23±1.84 cal K^−1^ mol^−1^). A big difference between the dissociation processes of **V3** and **V1** is the lower activation enthalpy measured for **V3** (Δ*H* ^≠^_maj_=8.87±0.41 kcal mol^−1^) compared to that of **V1** (Δ*H* ^≠^_maj_=12.53±0.88 kcal mol^−1^). We attribute this difference to the differences in the van der Waals interactions between host and guest that need to be broken and formed during the exchange process. The question whether this effect results from a lower activation barrier or a higher ground state energy will be discussed in the next section. The difference in population between the major and minor species for the complex between **H_2_2** and **V3** is 2:1, which is the same value as observed for the complex between **H_2_2** and **V1**. These results demonstrate that the degree of unidirectionality in the bound viologen guest is determined by the length of the linker between the blocking group and the viologen moiety, which increases the bulkiness of the guest close to the cavity of the host if this linker is short. Guest **V4** contains a 2‐ethylbutyl group as head group and was expected to be less sterically encumbering than **V1**, but bulkier than **V3**. The 1D EXSY studies confirm this hypothesis as the activation Gibbs free energies for **H_2_2/V4** (Δ*G* ^≠^_maj_=18.50±0.66 kcal mol^−1^, Δ*G* ^≠^_min_=18.06±0.67 kcal mol^−1^) are higher than those for **H_2_2/V3**, containing a methyl group, but lower than those for **H_2_2/V1**, containing a methylcyclohexyl group.

### Kinetics versus thermodynamics

**Figure 4:**
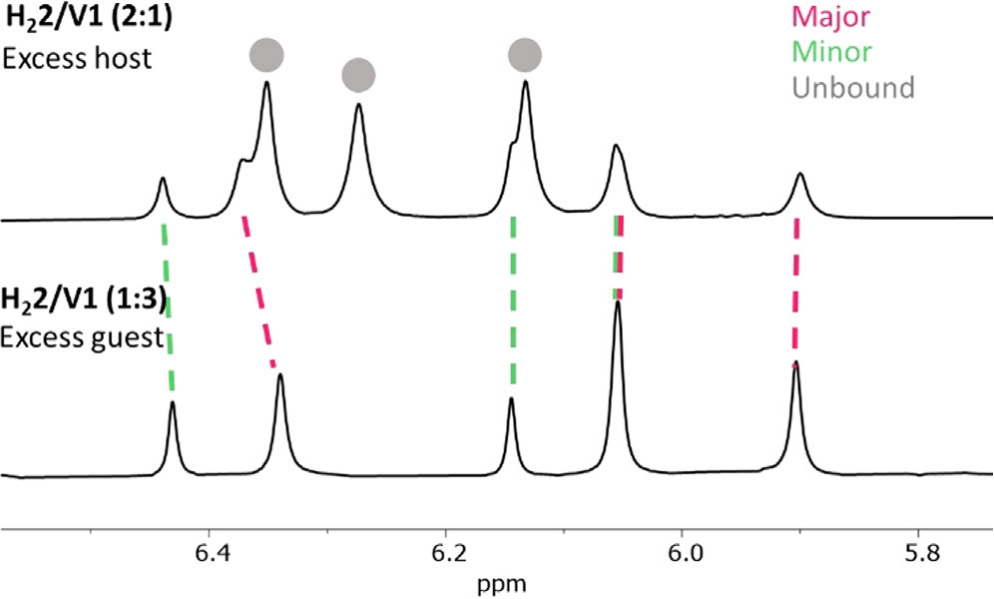
^1^H NMR spectra of the xylylene sidewall region of complexes between **H_2_2** and **V1** (CDCl_3_:CD_3_CN 1:1, v/v at 70 °C). Top: **H_2_2/V1** (4 mM:2 mM) showing the major and the minor abundant complexes, as well as the free host. Bottom: **H_2_2/V1** (2 mM:6 mM).

**Figure 5:**
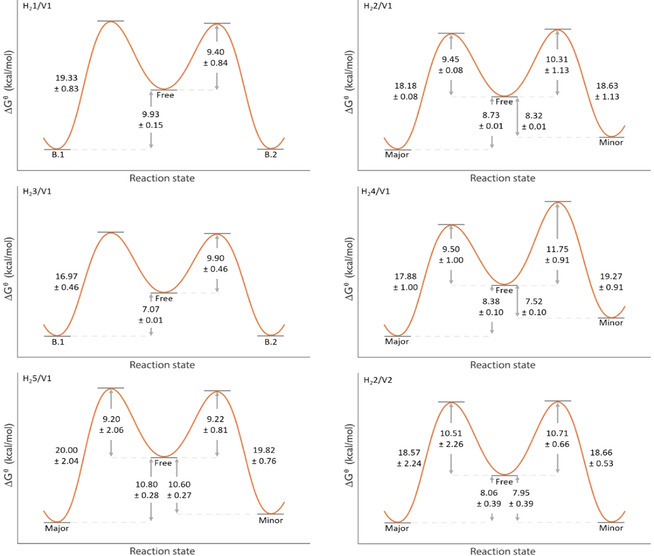
Energy diagrams of binding and exchange processes of various porphyrin cage‐viologen complexes.

In order to obtain more insight in the individual steps of the exchange reaction, the 1D EXSY studies of the complex between **H_2_2** and **V1** were repeated, but this time using an excess of host to follow the exchange of free to bound host and vice versa. In the top spectrum in Figure 4 an extra set of sidewall peaks is visible, but isolated peaks can still be selected to perform 1D EXSY studies. The activation Gibbs energies of these separate steps were compared to those of the previously determined overall process of exchange between the two complexed states (bound‐1 and bound‐2), see Table 4. It can be seen that the Gibbs free energy values for the individual steps do not differ significantly from the energy value of the overall process. This can be explained by assuming that, under the condition of excess of host, all guest is bound. In this case, when the exchange from free to bound host is followed, a preceding step will occur in which a guest first dissociates from another host. This process explains why the activation Gibbs free energy difference is insignificant, since the rate‐determining step for the exchange process is the dissociation of the guest from the other host. This experiment therefore provides additional evidence that the mechanism of guest exchange is dissociative. To find out whether the effect of structural variations in the hosts and guests on the rate of exchange is kinetic or thermodynamic in origin, we combined the previously obtained activation free energies of exchange with the differences in the ground state energies to construct overall potential energy diagrams (Figure 5). Since we already determined the overall energy barriers for exchange, we could calculate the energy barrier in going from the minor abundant complex to the free components by measuring the most energy‐costly step (exchange from major abundant complex to free components) by 1D EXSY and calculating the difference from the overall energy barrier.

**Table 4:** Activation free energy (Δ*G* ^≠^) values of the exchange processes in the complex between **H_2_2** and **V1**, in which the individual exchange steps are compared the overall process.

Exchange process	Δ*G* ^≠^ (at 40 °C, kcal mol^−1^)
Free to bound	19.31±0.87
Bound to free	19.33±0.83
Bound‐1 to Bound‐2	19.81±0.43

From these diagrams it is clear that there are significant differences between the kinetic and thermodynamic factors of exchange. For example, the binding between **H_2_1** and **V1** is stronger than that between the minor abundant complex between **H_2_4** and **V1** (Δ*G* ^⊖^_bind_=9.93±0.15 kcal mol^−1^ compared to Δ*G* ^⊖^_bind_=7.52±0.10 kcal mol^−1^, respectively), while in contrast the activation barrier for binding a guest in the free host is significantly lower for the complex between **H_2_4** and **V1** (Δ*G* ^≠^=9.40±0.84 kcal mol^−1^) compared to that for the minor abundant complex between **H_2_1** and **V1** (Δ*G* ^≠^_min_=11.75±0.92 kcal mol^−1^). This means that slow exchange between the components of the host‐guest complex does not necessarily indicate stronger host‐guest binding, and vice versa. The activation barriers going from a bound to a free complex decrease in energy upon an increase in the number of nitro‐groups on the xylylene sidewalls of the host, with the exception of **H_2_4**. To investigate whether only steric interactions play a role in the decrease of the activation barrier or also electronic ones, we investigated with 1D EXSY the properties of **H_2_5**, in which the strongly electron‐withdrawing nitro‐group is replaced by the strongly electron‐donating amino‐group. Remarkably, the activation barriers going from a bound to free complex in the case of **H_2_5** and **V1** are in the same range as that for the complex between **H_2_2** and **V1**. This result suggests that mostly steric interactions affect the kinetics of guest binding. However, the ground state energy difference between the free and the bound state is significantly larger for the complex of **H_2_5** than for any of the other complexes with **V1**. This difference suggests that electronic effects determine the thermodynamics of binding. The latter effect is explained by the occurrence of more favorable π‐π interactions between the electron‐poor viologen and the electron‐rich sidewall of **H_2_5** compared to the interaction of this guest with the unsubstituted host **H_2_1**. With regard to the structure of the guest, the complexes between **H_2_2** and **V1** and between **H_2_2** and **V2** display similar energy barriers for exchange. Despite this similarity, the degree of unidirectionality (*K*_maj/min_) of guest binding is significantly different, viz. 2 for **H_2_2/V1** and 1.2 for **H_2_2/V2**. This difference suggests that the longer linker between the blocking group and the viologen moiety of **V1** reduces the steric interactions between this blocking group and the nitro‐group of the host enough to direct the group at the open end to the unsubstituted face of the host, resulting in a higher degree of unidirectionality. 1D EXSY measurements on the complex between **H_2_2** and **V3** under the conditions of excess host were unsuccessful due to excessive broadening of the proton signals as a result of a too fast exchange of the components. Even at lower temperatures (down to −30 °C) the signals could not be accurately selected.

### Polymer exchange

**Figure 6:**
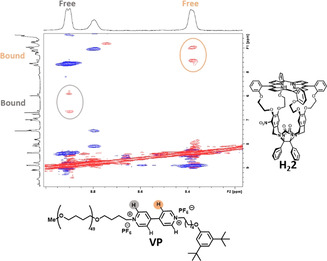
2D ROESY spectrum of a mixture of **H_2_2** and **VP** (1:1.2 molar ratio; chloroform‐*d*‐[D_3_]acetonitrile, 1:1 (v/v)), depicting exchange signals for the *ortho* and *meta* protons of **VP** of free and bound guest.

After having performed host‐guest exchange studies with low molecular weight viologen guests, we decided to investigate whether we could apply 1D EXSY to follow the exchange process of a viologen guest with a polymeric side chain. An NMR sample containing mono‐nitrated porphyrin cage **H_2_2** and a poly‐(THF)_49_‐substituted viologen, capped with a blocking group (**VP**, for structure see Figure 6) in [D_3_]acetonitrile:chloroform‐*d*, 1:1 (v/v) showed observable exchange of the polymer in the 2D ROESY spectrum after a mix time of 400 ms (Figure 6). The intensity of the exchange cross‐peaks was relatively low, which indicates slow exchange. From the 2D ROESY spectrum it was unclear whether full dissociation of the polymer from the host occurred, or just movement of the host along the polymer chain away from the viologen moiety, followed by re‐association (slippage). To investigate this process in more detail, we carried out exchange studies on the **H_2_2/VP** complex, following both the exchange in the orientation of the host on the viologen moiety as a function of time, requiring full dissociation and thus excluding slippage, and the dissociation of the guest by following bound guest to unbound guest. If the rate constants obtained from the two types of experiments do not differ, only full dissociation occurs. If the rate constant of the dissociation from bound to free guest is larger than the rate constant of the change in orientation, slippage as well as full dissociation takes place. From the performed EXSY studies at 60 °C, a higher rate constant was obtained for the dissociation from bound to free guest (*k*_b→f_: 1.998±0.089 s^−1^) compared to the orientation change which requires full dissociation (*k*_or_: 0.374±0.028 s^−1^). This means that both slippage and full dissociation occurs in this host/guest system and that at the measured temperature of 60 °C the slippage rate constant is *k*_b→f_−*k*_or_=1.625±0.094 s^−1^. This rate constant is in the same range of magnitude as the rate constants we found for the porphyrin host with the small guest **H_2_2/V1** at 60 °C (*k*_min_=2.525±0.077 s^−1^ and *k*_maj_=1.337±0.058 s^−1^), which means that exchange with small guest molecules (**V1**–**V5**) is almost fully under thermodynamic control.

Focusing on the extent of unidirectionality, the ratio of the major to the minor abundant host‐guest complex was 1.18 to 1 for **H_2_2/VP** (compared to 2 to 1 for **H_2_2/V1**), which indicates that the system shows less unidirectionality when the chain at the open end of the viologen is elongated. To investigate whether a thermodynamic equilibrium had already been reached for the host‐polymer guest complex and whether the major to minor ratio is purely a result of the thermodynamic equilibrium, a week after sample preparation the *K*_maj/min_ ratio was determined again; it had remained constant (Figure SI29). A duplicate experiment was performed later using the same conditions, which resulted within experimental error in the same major to minor ratio (1.23 to 1).

## Conclusion

From our studies it is evident that selective exchange spectroscopy (1D EXSY) NMR is a suitable method to monitor the exchange kinetics of host‐guest systems involving porphyrin cage compounds and viologen guest molecules, including polymeric ones. By measuring exchange rates at different guest concentrations, we were able to demonstrate that the host‐guest exchange mechanism follows a dissociative process. This mechanism was corroborated by the observation that the overall energy involved in going to different bound states is not different from the energies linked to individual dissociation and re‐association steps in the process of going from a free to a bound host, and vice versa. The introduction of sterically bulky groups on one side of the host, as in the *syn*‐dinitro compound **H_2_4**, increases the population difference of the major and minor occurring complex significantly, that is, in the direction of a higher degree of unidirectional binding. Increasing the steric hindrance in the guest by shortening the linker between the blocking group and the viologen moiety resulted in a smaller degree of unidirectional binding, although it increased the overall energy barrier for exchange. Decreasing the steric bulk at the open end of the viologen moiety from a methylcyclohexyl to a methyl group did not affect the degree of unidirectional binding, but resulted in an overall decrease in activation energy of exchange. By comparing the host‐guest binding behavior of the *syn*‐dinitro‐substituted host **H_2_4** with that of the other hosts, it appears that steric factors of the substituents have a larger impact on the activation energy barriers of host‐guest exchange than electronic factors. The results obtained with the amino‐and nitro‐substituted hosts suggest that electronic effects of the host substituents have a larger impact on the energies of the ground states of the complexes than steric effects. Taking all previous observations together, we can conclude that more unidirectional binding can be achieved if bulkier groups are introduced to one face of the porphyrin cage compound. For instance, one nitro‐group on one side of the porphyrin cage compound leads to a selectivity of 67 % major product and 33 % minor product, whereas two nitro‐groups on the same side of the host already increases this selectivity to 80 % major and 20 % minor product. Lastly, we have shown that the EXSY studies are also applicable to exchange studies with guests containing a polymer chain (**H_2_2/VP**). Furthermore, we determined that full dissociation as well as slippage takes place and that the slippage‐rate constant was in the same range of magnitude as the rate constant for exchange with a smaller guest (**H_2_2/V1**), which confirms the statement that exchange of small guests (**V1**–**V5**) takes place almost fully under thermodynamic control.

## Conflict of interest

The authors declare no conflict of interest.

## Supporting information

As a service to our authors and readers, this journal provides supporting information supplied by the authors. Such materials are peer reviewed and may be re‐organized for online delivery, but are not copy‐edited or typeset. Technical support issues arising from supporting information (other than missing files) should be addressed to the authors.

Supplementary
