# Supplementary material for: Host–Guest Exchange of Viologen Guests in Porphyrin Cage Compounds as Studied by Selective Exchange Spectroscopy (1D EXSY) NMR
Source: Angew Chem Int Ed Engl. 2020 Nov 18;60(3):1254–62. doi: 10.1002/anie.202010335 (PMC7839762; doi:10.1002/anie.202010335)
Supplement: Supplementary file 1 — Supplementary [file ANIE-60-1254-s001.pdf]

Angewandte Chemie
Eine Zeitschrift der Gesellschaft Deutscher Chemiker

Supporting Information

Host–Guest Exchange of Viologen Guests in Porphyrin Cage Compounds as Studied by Selective Exchange Spectroscopy (1D EXSY) NMR

Anne Swartjes, Paul B. White, Marijn Lammertink, Johannes A. A. W. Elemans,* and Roeland J. M. Nolte**

anie_202010335_sm_miscellaneous_information.pdf

Author Contributions

R.N. Conceptualization: Lead; Data curation: Lead; Funding acquisition: Lead; Investigation: Lead; Methodology: Lead; Project administration: Lead; Supervision: Equal; Validation: Equal; Writing—Original Draft: Lead; Writing—Review & Editing: Lead
A.S. Data curation: Equal; Formal analysis: Equal; Investigation: Equal; Methodology: Equal; Validation: Equal; Writing—Original Draft: Lead; Writing—Review & Editing: Lead
P.W. Conceptualization: Equal; Data curation: Equal; Formal analysis: Lead; Methodology: Lead; Supervision: Equal; Validation: Equal; Writing—Review & Editing: Equal
M.L. Data curation: Supporting; Formal analysis: Supporting; Investigation: Supporting; Validation: Supporting; Writing—Review & Editing: Supporting
J.E. Conceptualization: Equal; Data curation: Equal; Formal analysis: Equal; Investigation: Equal; Methodology: Supporting; Supervision: Equal; Validation: Equal; Visualization: Equal; Writing—Original Draft: Equal; Writing—Review & Editing: Equal.

Contents

Synthesis of viologen-derived guests 4

1-((5-Bromopentyl)oxy)-3,5-di-tert-butylbenzene (1a) 4

1-(5-(3,5-Di-tert-butylphenoxy)pentyl)-[4,4'-bipyridin]-1-ium hexafluorophosphate (2a) 5

1-(3-(3,5-Di-tert-butylphenoxy)propyl)-[4,4'-bipyridin]-1-ium hexafluorophosphate (2b) 5

Guest V1 5

Guest V2 6

Guest V3 6

Guest V4 7

Guest V5 7

NMR spectra of synthesized guests (precursors) 8

Synthesis of porphyrin cage compounds 18

$H_2$5 18

Previous work 21

General setup and information on performed 1D EXSY experiments 22

Experimental procedure for the 1D EXSY experiments involving the change in orientation of host (host: 2 mM, guest: 6 mM) 22

Sample preparation 22

Setup of the 1D EXSY experiments 22

Data processing and equations 22

Experimental procedure for 1D EXSY experiments involving the conversion of bound to free host (host: 4 mM, guest: 2 mM) 23

Sample preparation 23

Setup of the 1D EXSY experiments 24

1D EXSY Pulse sequence 24

Data processing and equations 24

Experimental procedure for the fluorescence binding titrations 28

Sample preparation 28

Fluorescence titrations 28

Data processing 28

Error propagation calculation 30

Construction of potential energy diagrams 30

T_1 determination and an overview of the 1D EXSY NMR studies 31

Overview of the results of the fluorescence titration experiments 32

Fluorescence binding studies fits 33

Spartan calculations 34

1D EXSY studies with polymer (VP) and H_22 36

Integration data of the exchange for H_22/V1 39

Fits of the 1D EXSY studies of all systems following the change in orientation of the guest in the host 65

Fits of the 1D EXSY studies of all systems following the conversion of bound host to free host: initial rates 74

Overview of chemical shifts of different host/guest complexes 80

Determination of the guest orientations by 2D ROESY and HSQC 81

Evidence for directional versus rotational or positional exchange 110

References **111**

Synthesis of viologen-derived guests

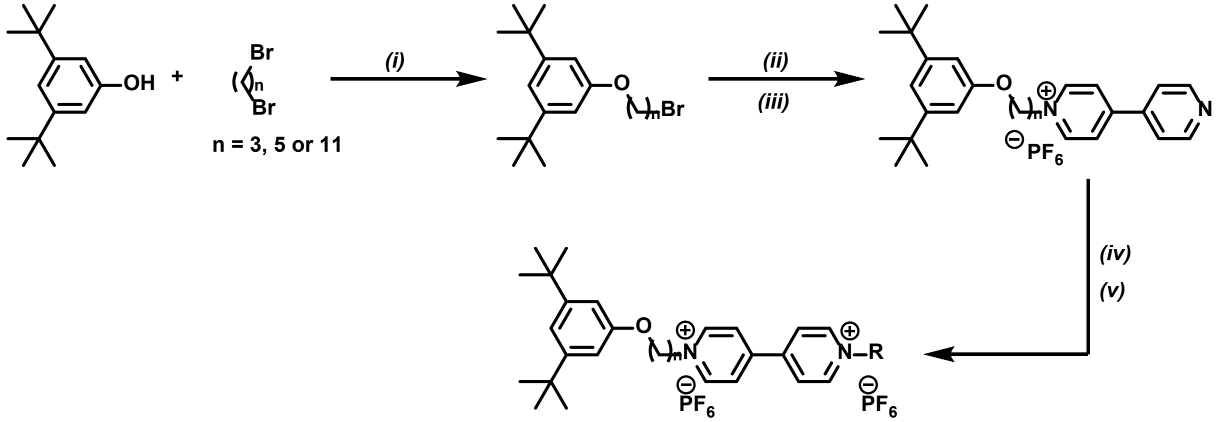

Scheme SI1. Reaction scheme for the synthesis of the viologen-derived guests. Reagents and conditions: *(i) K_2CO_3 (2 eq.), DMF, reflux; (ii) 4,4'-bipyridine (5 eq.), DMF, 90 °C; (iii) saturated solution of NH_4PF_6 (aq); (iv) for R= methylcyclohexyl (bromomethyl)cyclohexane (10 eq.), DMF, 70 °C; (iv) for R=CH_3 MeI (10 eq.), DMF, 40 °C; (iv) for R=2-ethylbutyl 1-bromo-2-ethylbutane (10 eq.), DMF, 90 °C; (v) saturated solution of NH_4PF_6 (aq).*

1-((5-Bromopentyl)oxy)-3,5-di-tert-butylbenzene (1a)[1]**.** A mixture in DMF (70 mL) containing 1,5-dibromopentane (14.2 mL, 10.6 mmol), K_2CO_3 (3.0 g, 21.7 mmol), and 3,5-di-*tert*-butylphenol (2.2 g, 10.6 mmol) was stirred for 3 hours at 90 °C. The solution was allowed to cool, filtered, and evaporated *in vacuo*. The remaining dibromopentane was removed using a Kugelrohr at 160 °C under reduced pressure. The resulting brown solid was purified by column chromatography (silica, eluent n-heptane/DCM = 7:3, v/v) to give **1a** as a white solid in a yield of 31%.

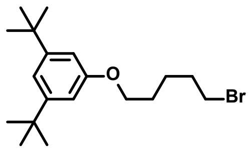

^{1}H NMR (400 MHz, chloroform-*d*): δ (ppm) = 7.05 (t, *J* = 1.67 Hz, 1H, Ar*H*), 6.73 (d, *J* =1.67 Hz, 2H, Ar*H*), 4.11 (t, *J* = 6.28 Hz, 2H, O*CH₂*), 3.45 (t, *J* = 6.83 Hz, 2H, C*H₂*Br), 1.8 (m, 4H, C*H₂*), 1.51 (m, 2H, C*H₂*), 1.29 (s, 18H, C*H₃*).

1-(2-Bromoethoxy)-3,5-di-tert-butylbenzene (1b)[2]. A mixture in DMF (70 mL) containing 1,3-dibromopropane (11 mL, 106 mmol, 10 eq.), K_2CO_3 (3.0 g, 21.7 mmol) and 3,5-di-*tert*-butylphenol (2.2 g, 10.6 mmol, 1 eq.) was stirred under argon atmosphere for 3 hours at 90 °C. After completion, the solution was allowed to cool to room temperature, filtered, and evaporated *in vacuo*. The remaining dibromopropane was distilled off using a Kugelrohr at 160 °C under reduced pressure. The obtained brown crude solid was purified by column chromatography (silica, eluent n-heptane/DCM = 7:3, v/v) to give **1b** as a white solid in a yield of 28%.

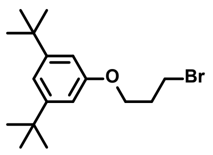

^{1}H NMR (400 MHz, chloroform-*d*): δ (ppm) = 7.02 (t, *J* = 1.60 Hz, 1H, Ar*H*), 6.75 (d, *J* = 1.60 Hz, 2H, Ar*H*), 3.98 (t, *J* = 5.74 Hz, 2H, C*H₂*), 3.63 (t, *J* = 6.48 Hz, 2H, C*H₂*), 1.64 (tt, J_t = 6.11, 6.18 Hz, 2H, C*H₂*), 1.32 (s, 18H, C*H₃*,).

1-((11-bromoundecyl)oxy)-3,5-di-tert-butylbenzene (1c) [3]

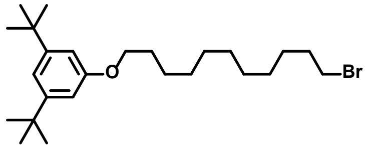

A mixture in DMF (35 mL) containing 1,11-dibromoundecane (11 mL, 40 mmol, 10 eq.), K_2CO_3 (1.0 g, 10 mmol) and 3,5-di-*tert*-butylphenol (1.0 g, 5 mmol, 1 eq.) was stirred under argon atmosphere for 2 days at 21 °C. After completion,

the solution was filtered and evaporated *in vacuo*. The remaining 1,11-dibromopundecane was distilled off using a Kugelrohr at 195 °C under reduced pressure. The obtained brown crude oil was purified by column chromatography (silica, eluent n-heptane/DCM = 7:3, v/v) to give **1c** as a colorless oil in a yield of 30%.

^{1}H NMR (500 MHz, chloroform-*d*): δ (ppm) = 7.01 (t, *J* = 1.47 Hz, 1H, Ar*H*), 6.75 (d, *J* = 1.54 Hz, 2H, Ar*H*), 3.96 (t, *J* = 6.68 Hz, 2H, C*H₂*), 3.41 (t, *J* = 6.94 Hz, 2H, C*H₂*), 1.85 (tt, *J* = 14.37, 6.99 Hz, 2H, C*H₂*), 1.78 (tt, *J* = 13.84, 6,79 Hz, 2H, C*H₂*), 1.51-1.38 (m, 6H, C*H₂*), 1.39-1.33 (m, 4H, C*H₂*), 1.31 (s, 18H, C*H₃*), 1.30-1.28 (m, 4H, C*H₂*) .

1-(5-(3,5-Di-tert-butylphenoxy)pentyl)-[4,4'-bipyridin]-1-ium hexafluorophosphate (2a). A mixture in DMF (20 mL) was prepared containing **1a** (1.0 g, 3 mmol) and 4,4'-bipyridine (2.0 g, 14 mmol). The reaction mixture was stirred at 90 °C for 72 hours and concentrated *in vacuo*. The resulting crude product was added to a saturated NH_4PF_6 solution (200 mL). The precipitate was filtered off and washed with water. The product was purified by column chromatography (silica, eluent DCM/MeOH/MeNO = 6:1:1) to give **2a** as a white solid in a yield of 54%.

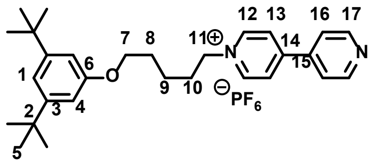

^{1}H NMR (400 MHz, acetonitrile-*d₃*): δ (ppm) = 8.79 (dd, *J* = 4.30, 1.41 Hz, 2H, **12**), 8.75 (dd, *J* = 4.40, 1.37 Hz, 2H, **13**), 8.18 (dd, *J* = 4.39, 1.37 Hz, 2H, **17**), 7.60 (dd, *J* = 4.33, 1.60 Hz, 2H, **16**), 7.01 (t, *J* = 1.57 Hz, 1H, **1**), 6.72 (d, *J* = 1.66 Hz, 2H, **4**), 4.65 (t, *J* = 7.51 Hz, 2H, **7**), 3.99 (t, *J* = 6.06 Hz, 2H, **11**), 2.11 (m, 2H, **8**), 1.85 (m, 2H, **10**), 1.59 (m, 2H, **9**), 1.29 (s, 18H, **5**).

^{13}C{^{1}H} NMR (125 MHz, acetonitrile-*d*₃): δ (ppm) = 158.60 (**6**), 154.28 (**14**), 152.23 (**3**), 151.29 (**12**), 144.90 (**13**), 141.10 (**15**), 126.13 (**16**), 121.77 (**17**), 114.82 (**1**), 108.82 (**4**), 67.16 (**11**), 61.59 (**7**), 34.84 (**2**), 31.20 (**5**), 30.96 (**8**), 28.70 (**10**), 22.57 (**9**).

MALDI-TOF: m/z: 431.055 $(M+H)^+$; calculated for $C_{29}H_{39}N_2O + H^+$ m/z: 431.640.

1-(3-(3,5-Di-tert-butylphenoxy)propyl)-[4,4'-bipyridin]-1-ium hexafluorophosphate (2b). In DMF (20 mL) compound **1b** (1.0 g, 3 mmol) was dissolved together with 4,4'-bipyridine (2.0 g, 14 mmol). The resulting mixture was stirred under an argon atmosphere at 90 °C for 72 hours. After cooling, the reaction mixture was concentrated *in vacuo* and was dissolved in a minimum amount of acetonitrile. A saturated solution of NH_4PF_6 (200 mL) was added. The precipitate was filtered off and washed with water. The product was purified by column chromatography (silica, eluent DCM/MeOH/$MeNO_2$ = 6:1:1, v/v) to give **2b** as a white solid in a yield of 50%.

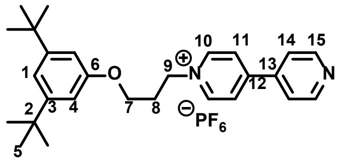

^{1}H NMR (500 MHz, acetonitrile-*d*₃): δ (ppm) = 8.90-8.85 (m, 4H, **10** and **11**), 8.36 (dd, *J* = 4.90, 1.61 Hz, 2H, **15**), 7.84 (dd, *J* = 4.35, 1.74 Hz, 2H, **14**), 7.07 (t, *J* = 1.63 Hz, 1H, **1**), 6.60 (d, *J* = 1.60 Hz, 2H, **4**), 4.81 (t, *J* = 6.44 Hz, 2H, **9**), 4.16 (t, *J* = 5.47 Hz, 2H, **7**), 2.52 (m, 2H, **8**), 1.26 (s, 18H, **5**).

^{13}C{^{1}H} NMR (125 MHz, acetonitrile-*d*₃): δ (ppm) = 158.57 (**13**), 158.43 (**6**), 154.61 (**3**), 151.77 (**10**), 145.93 (**11**), 141.62 (**12**), 126.00 (**14**), 122.34 (**15**), 115.85 (**1**), 109.18 (**4**), 65.02 (**7**), 60.20 (**9**), 35.11 (**2**), 31.16 (**5**), 30.69 (**8**).

MALDI-TOF: m/z: 402.977 $(M+H)^+$; calculated for $C_{27}H_{35}N_2O + H^+$ m/z: 403.270.

Guest V1. (Bromomethyl)cyclohexane (0.034 g, 0.19 mmol) and **2a** were dissolved in DMF (1 mL) and the resulting mixture was stirred for 3 days at 70 °C. The solution was allowed to cool and the product was precipitated by adding diethyl ether.

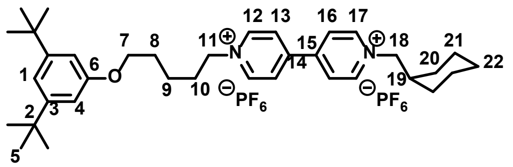

The precipitate was filtered off and mixed with acetone (2 mL). The resulting suspension was added dropwise to a saturated solution of NH_4PF_6 (100 mL). The suspension was filtered and the precipitate was washed with water and multiple times with dichloromethane. The product was obtained as a white solid in a yield of 37%.

^{1}H NMR (500 MHz, acetonitrile-d_3): δ (ppm) = 8.60 (dd, *J* = 4.40, 1.40 Hz, 2H, **12**), 8.51 (dd, *J* = 4.17, 1.40 Hz, 2H, **17**), 8.05 (m, 4H, **13** and **16**), 6.72 (t, *J* = 1.66 Hz, 1H, **1**), 6.42 (d, *J* = 1.59 Hz, 2H, **4**), 4.34 (t, *J* = 7.45 Hz, 2H, **11**), 4.15 (d, *J* = 7.51 Hz, 2H, **18**), 3.70 (t, *J* = 6.29, 2H, **7**), 1.80 (m, 2H, **10**), 1.67 (m, 1H, **19**), 1.50 (m, 2H, **8**), 1.45 (m, 2H, **22**), 1.27 (m, 4H, **9**), 0.97 (s, 18H, **5**), 0.92 (m, 4H, **20**), 0.80 (m, 2H, **21**).

^{13}C{^{1}H} NMR (125 MHz, acetonitrile-d_3): δ (ppm) = 158.66 (**6**), 152.24 (**3**), 149.92 (**14**, **15**), 145.73 (**12**), 145.13 (**17**), 127.35 (**16**), 127.23 (**13**), 114.87 (**1**), 108.82 (**4**), 67.61 (**18**), 67.07 (**7**), 62.23 (**11**), 39.55 (**20**), 34.82 (**2**), 31.14 (**5**), 31.03 (**10**), 29.33 (**21**), 28.56 (**8**) 25.65 (**19**), 25.08 (**22**), 22.61 (**9**).

MALDI-TOF: m/z: 528.564 $(M+H)^+$; calculated for $C_{36}H_{52}N_2O + H^+$ m/z: 528.820.

Guest V2. A solution was prepared containing **2b** (0.068 g, 18.0 mmol) and (bromomethyl)cyclohexane (0.034 g, 0.19 mmol) in anhydrous DMF (1 mL). The reaction mixture was stirred for 3 days at 70 °C. The solution was cooled to room temperature and the product was precipitated in diethyl ether. The precipitate was filtered off and mixed with acetone (2 mL). The resulting suspension was added dropwise to a saturated solution of NH_4PF_6 (100mL). The suspension was filtered and the precipitate was washed with water and dichloromethane. Compound **V2** was obtained as a white solid in a yield of 35%.

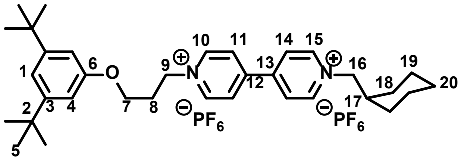

^{1}H NMR (500 MHz, acetonitrile-d_3): δ (ppm) = 9.01 (d, *J* = 4.57, 1.36 Hz, 2H, **10**), 8.87 (d, 2H, *J* = 4.57, 1.36 Hz, **15**), 8.43 (m, 4H, **11** and **14**), 7.08 (t, *J* = 1.65 Hz, 1H, **1**), 6.65 (d, *J* = 1.41 Hz, 2H, **4**), 4.89 (t, *J* = 6.65 Hz, 2H, **9**), 4.51 (d, *J* = 7.18 Hz, 2H, **16**), 4.16 (t, *J* = 4.84 Hz, 2H, **7**), 2.54 (m, 2H, **8**), 2.01 (m, 1H, **17**), 1.72 (m, 2H, **20**), 1.62 (m, 4H, **18**), 1.28 (s, 18H, **5**), 1.14 (m, 4H, **19**).

^{13}C{^{1}H} NMR (125 MHz, acetonitrile-d_3): δ (ppm) = 158.95 (**6**), 152.28 (**3**), 150.56 (**12**, **13**), 145.92 (**10**), 145.56 (**15**), 126.97 (**11**, **14**), 116.15 (**1**), 108.81 (**4**), 67.30 (**16**), 64.39 (**7**), 60.19 (**9**), 39.53 (**17**), 35.10 (**2**), 30.70 (**5**), 30.34 (**8**), 29.56 (**18**), 29.36 (**19**), 25.83 (**20**).

MALDI-TOF: m/z: 500.183 $(M+H)^+$ calculated for $C_{34}H_{48}N_2O + H^+$ m/z: 500.770.

Guest V3. A pressure tube was charged with **2a (**1.0 g, 2.072 mmol) and anhydrous DMF (10 mL). A large excess of methyl iodide (1 mL, 16 mmol) was added to the mixture and the tube was sealed. The reaction mixture was heated at 40 °C for 3 days. The mixture was allowed to cool to room temperature and diethyl ether was added. The suspension was filtered and the precipitate was suspended in a small amount of acetone (1 – 2 mL). The suspension was added dropwise to a saturated NH_4PF_6 (100 mL) solution. The resulting white precipitate was filtered, washed with water and n-pentane and was dried in high vacuum after which **V3** was obtained in a yield of 36%.

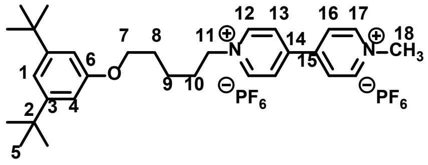

^{1}H NMR (500 MHz, acetonitrile-d_3): δ (ppm) = 8.91 (d, *J* = 6.77 Hz, 2H, **12**), 8.84 (d, *J* = 6.65 Hz, 2H, **17**), 8.38 (d, *J* = 6.67 Hz, 2H, **13**), 8.35 (d, *J* = 6.67 Hz, 2H, **16**), 7.04 (d, *J* = 1.65 Hz, 2H, **1**), 6.73 (t, *J* = 1.61 Hz, 1H, **4**), 4.00 (t, *J* = 6.24 Hz, 2H, **7**), 4.39 (s, 3H, **18**), 4.65 (t, *J* = 7.38 Hz, 2H, **11**), 2.09 (m, 2H, **10**), 1.82 (m, 2H, **8**), 1.57 (m, 2H, **9**), 1.29 (s, 18H, **5**).

$^{13}C\{^{1}H\}$ NMR (126 MHz, acetonitrile-d_3): δ (ppm) = 157.84 (**6**), 152.32 (**3**), 149.97 (**14**), 149.71 (**15**), 146.49 (**17**), 145.72 (**12**), 127.67 (**16**), 126.80 (**13**), 114.88 (**1**), 108.83 (**4**), 66.39 (**7**), 62.84 (**11**), 49.37 (**18**), 33.98 (**2**), 31.16 (**10**), 30.63 (**5**), 28.41 (**8**), 22.39 (**9**).

MALDI-TOF: m/z: 446.035 $(M+H)^+$ calculated for $C_{30}H_{42}N_2O + H^+$ m/z: 447.680.

Guest V4.

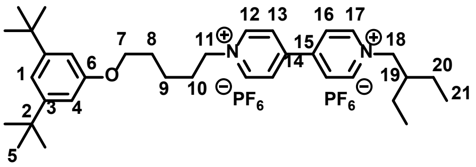

In DMF (20 mL) compound **2a** (1 g, 2 mmol) was dissolved and 2-ethylbutyl bromide (3 mL, 20 mmol) was added. The resulting mixture was stirred under an argon atmosphere at 90 °C for 72 hours. After cooling, the crude was concentrated *in vacuo* until a small amount of DMF was left. The solution was added dropwise to diethyl ether. The resulting suspension was filtered and the precipitate was suspended in a small amount of acetone (1 – 2 mL). The suspension was added dropwise to a saturated NH_4PF_6 (100 mL) solution. The resulting white precipitate was filtered, washed with water and DCM (3 times) and n-pentane and was dried in high vacuum after which **V4** was obtained as a white powder with a yield of 9%.

^{1}H NMR (500 MHz, acetonitrile-d_3): δ (ppm) = 8.92 (d, J = 6.68 Hz, 2H, **12**), 8.86 (d, J = 6.74 Hz, 2H, **17**), 8.36 (m, 4H, **13** and **16**), 7.05 (t, J = 1.41 Hz, 1H, **1**), 6.74 (d, J = 1.49 Hz, 2H, **4**), 4.66 (t, J = 7.67 Hz, 2H, **11**), 4.53 (d, J = 7.58 Hz, 2H, **18**), 4.00 (t, J = 6.19 Hz, 2H, **7**), 2.12-2.09 (m, 2H, **10**), 2.03-1.97 (m, 1H, **19**), 1.88-1.77 (m, 2H, **8**), 1.63-1.52 (m, 2H, **9**), 1.48-1.31 (m, 4H, **20**), 1.35 (1.29 (s, 18H, **5**), 0.93 (t, J = 7.45 Hz, 6H, **21**).

$^{13}C\{^{1}H\}$ NMR (126 MHz, acetonitrile-d_3): δ (ppm) = 158.90 (**6**), 152.31 (**3**), 149.93 (**14**), 145.85 (**17**), 145.78 (**15**), 145.45 (**12**), 127.22 (**13** and **16**), 114.93 (**1**), 108.90 (**4**), 67.07 (**7**), 65.14 (**18**), 62.02 (**11**), 42.42 (**19**), 34.55 (**2**), 30.82 (**5**), 30.75 (**10**), 28.36 (**8**), 22.38 (**9**), 21.96 (**20**), 9.35 (**21**).

MALDI-TOF: m/z: 517.326 $(M+H)^+$ calculated for $C_{42}H_{64}N_2O + H^+$ m/z: 516.810.

Guest V5.

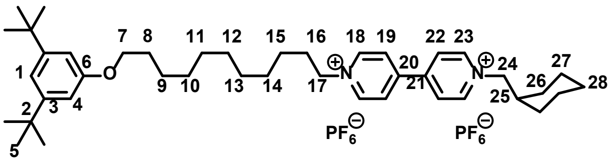

In DMF (20 mL) compound **1c** (700 mg, 1.59 mmol) was dissolved together with 4,4'-bipyridine (993 mg, 6.36 mmol). The resulting mixture was stirred under an argon atmosphere at 90 °C for 72 hours. After cooling, the crude was concentrated *in vacuo* and purified by column chromatography (silica, eluent DCM/MeOH/$MeNO_2$ = 6:1:1, v/v). The resulting yellow solid (800 mg, 1.34 mmol) was dissolved in anhydrous DMF (10 mL), (bromomethyl)cyclohexane (1.9 mL, 13.40 mmol) was added and the resulting mixture was heated to 90 °C and stirred under argon atmosphere for 3 days. The mixture was allowed to cool to room temperature and diethyl ether was added. The suspension was filtered and the precipitate was suspended in a small amount of acetone (1 – 2 mL). The suspension was added dropwise to a saturated NH_4PF_6 (100 mL) solution. The resulting white precipitate was filtered, washed with water and DCM (3 times) and n-pentane and was dried in high vacuum after which **V5** was obtained as a white powder in a yield of 3.2% over 2 steps.

^{1}H NMR (500 MHz, acetonitrile-d_3): δ (ppm) = 8.89 (d, J = 6.09 Hz, 2H, **18**), 8.82 (d, J = 5.92 Hz, 2H, **23**), 8.36 (m, 4H, **19** and **22**), 7.03 (t, J = 1.70 Hz, 1H, **1**), 6.73 (d, J = 1.67 Hz, 2H, **4**), 4.60 (t, J = 7.56 Hz, 2H, **17**), 4.46 (d, J = 7.40 Hz,

2H, **24**), 3.97 (t, J = 6.49 Hz, 2H, **7**), 2.05-1.98 (m, 4H), 1.80-1.67 (m,), 1.60-1.57 (m), 1.50-1.30 (m), 1.29 (s, 18H, **5**), 1.26-1.22 (m), 1.17-1.06 (m).

$^{13}C\{^1H\}$ NMR (126 MHz, acetonitrile-d_3): δ (ppm) = 158.99 (**6**), 151.87 (**3**), 149.75 (**20**), 145.66 (**18**), 145.63 (**21**), 145.46 (**23**) 127.03 (**19** and **22**), 114.66 (**1**), 108.75 (**4**), 67.49 (**7**), 67.19 (**24**), 62.01 (**17**), 39.26 (**25**) 34.78 (**2**), 30.84 (**16**), 30.50 (**5**), 29.14 (**12**, **13**, **14**), 28.74 (**10**, **11**, **15** and **26**-**28**), 25.78 (**9** and **10**), 24.93 (**8**).

MALDI-TOF: m/z: 615.347 $(M+H)^+$ calculated for $C_{42}H_{64}N_2O + H^+$ m/z: 612.990.

NMR spectra of synthesized guests (precursors)

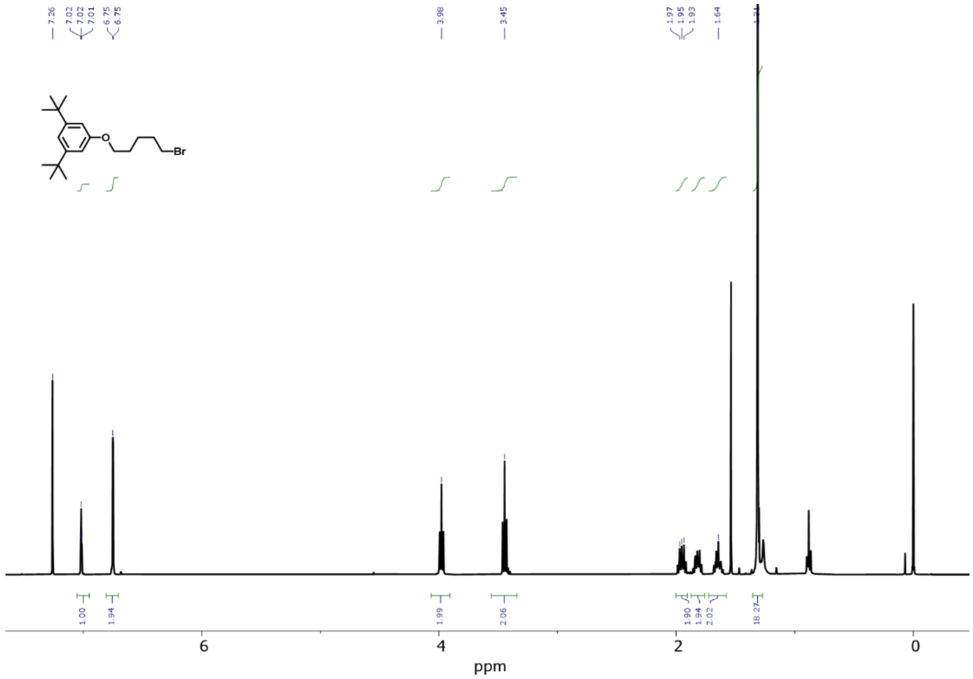

Figure SI1. ^1H NMR spectrum of **1a** (500 MHz, $CDCl_3$, 298 K).

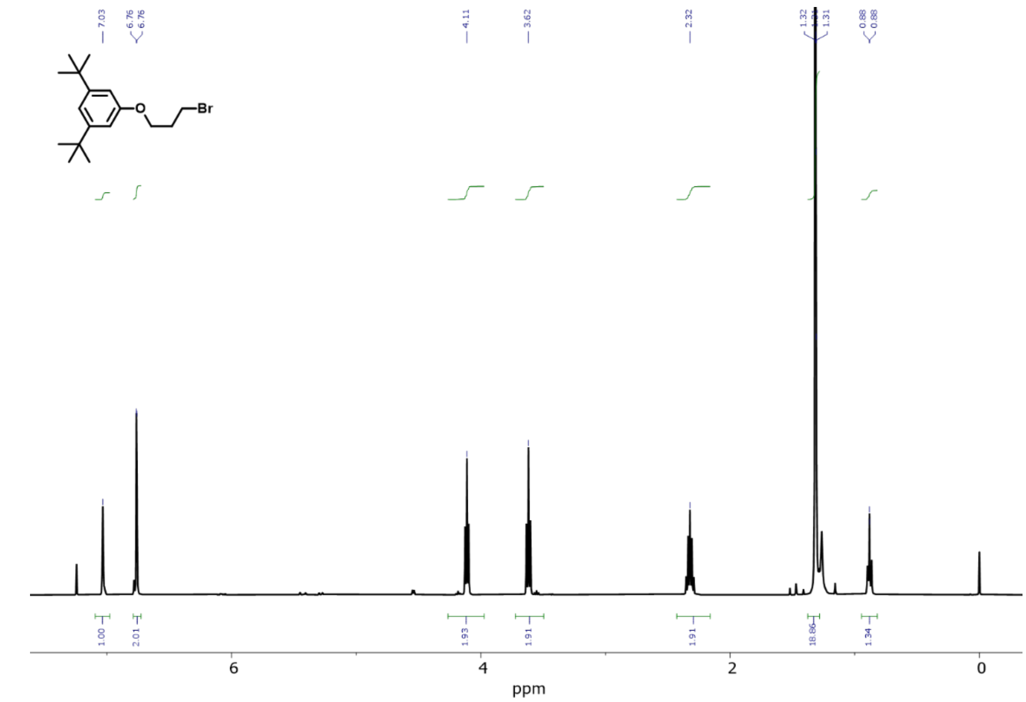

Figure SI2. ^{1}H NMR spectrum of **1b** (500 MHz, $CDCl_3$, 298 K).

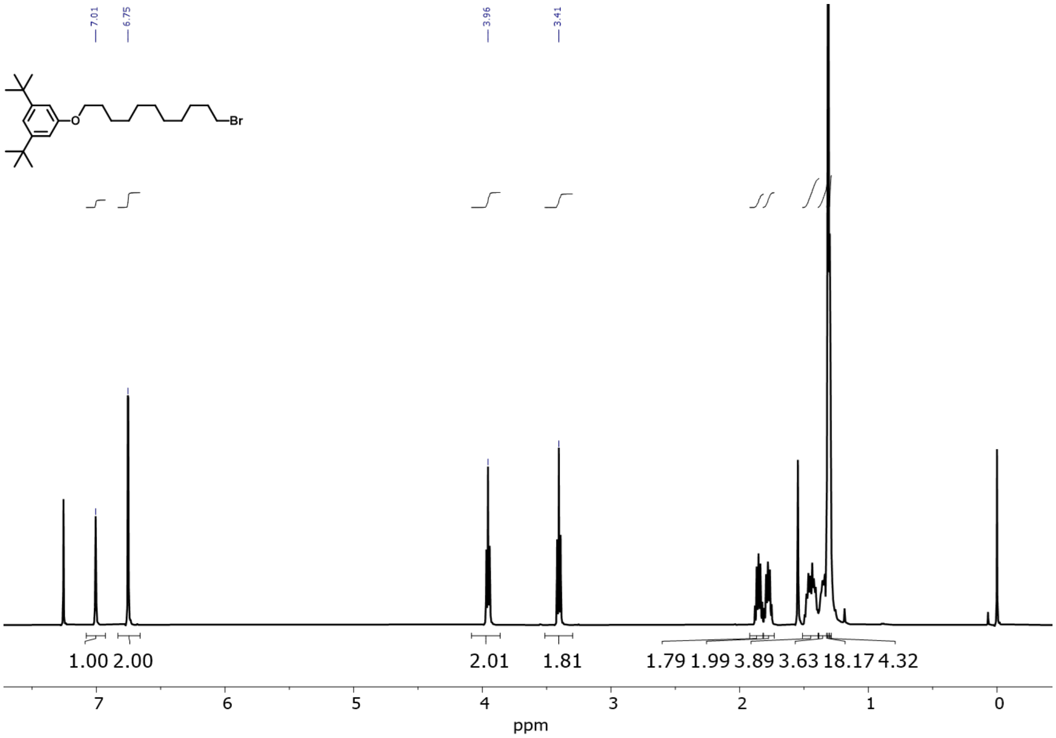

Figure SI3. ^{1}H NMR spectrum of **1c** (500 MHz, $CDCl_3$, 298 K).

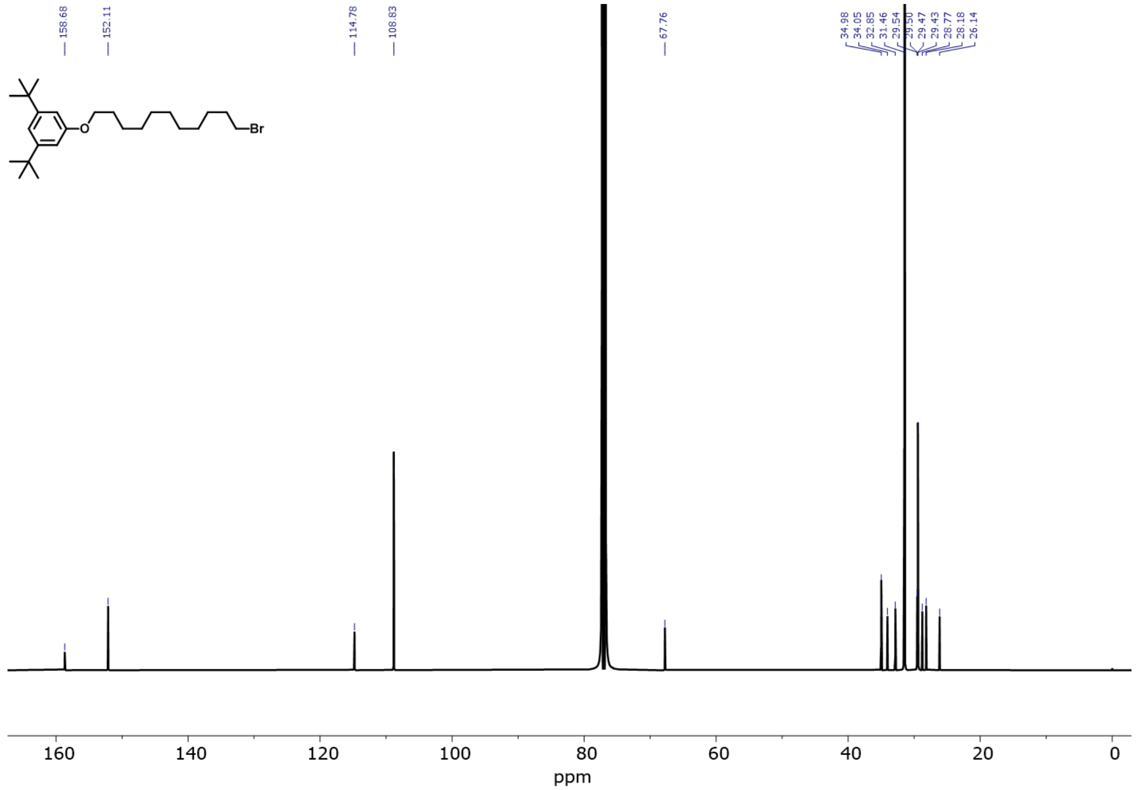

Figure SI4. ^{13}C NMR spectrum of **1c** (125 MHz, $CDCl_3$, 298 K).

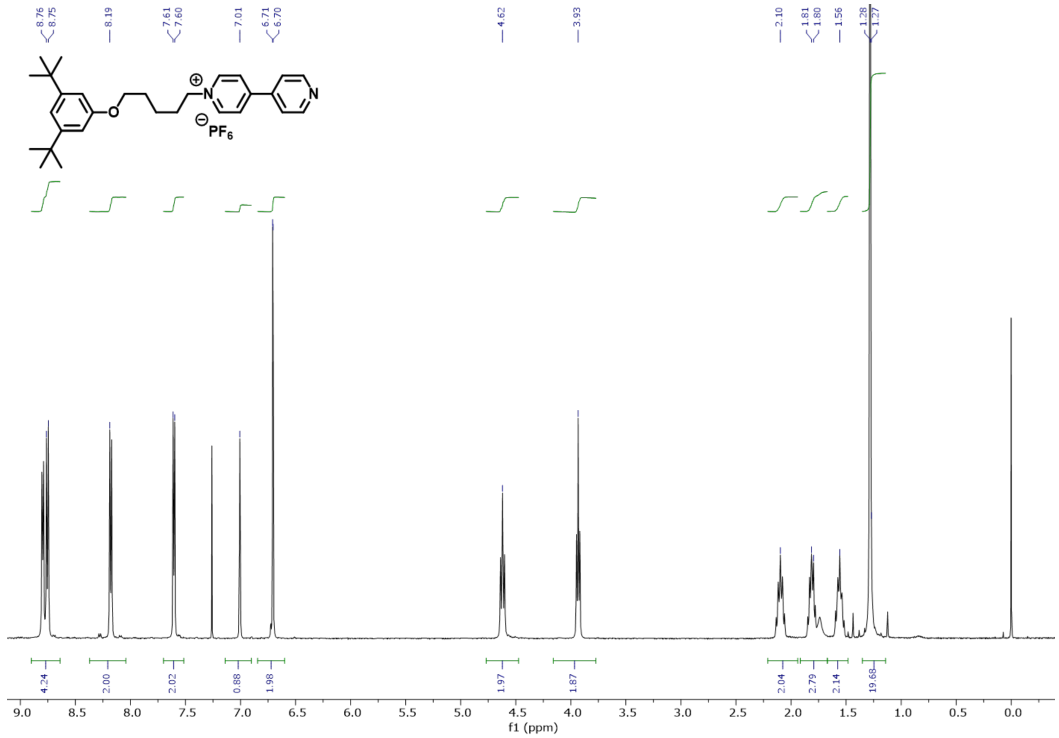

Figure SI5. ^{1}H NMR spectrum of **2a** (500 MHz, CD_3CN, 298 K).

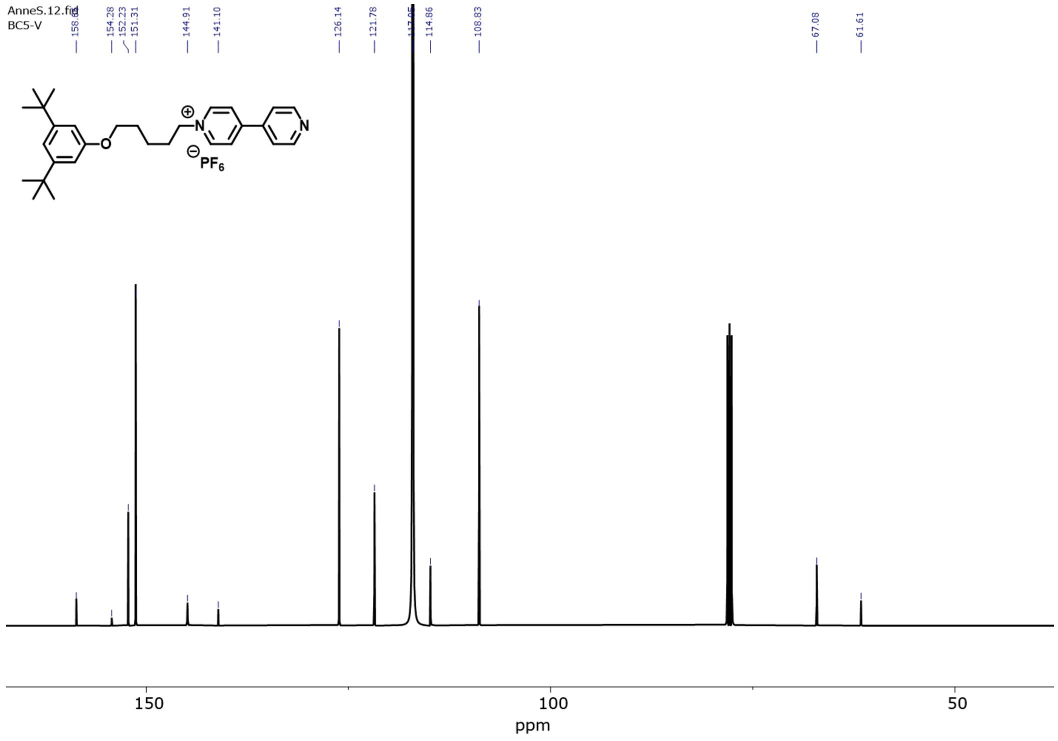

Figure SI6. ^{13}C NMR spectrum of **2a** (125 MHz, CD_3CN, 298 K).

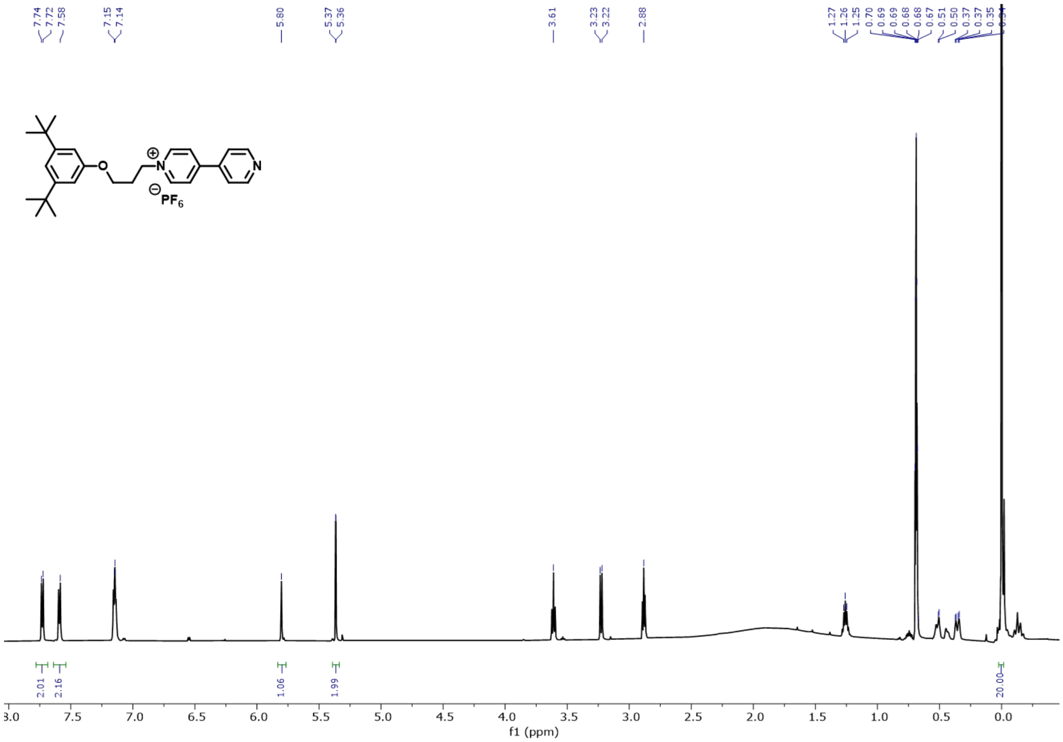

Figure SI7. ^{1}H NMR spectrum of **2b** (500 MHz, CD_3CN, 298 K).

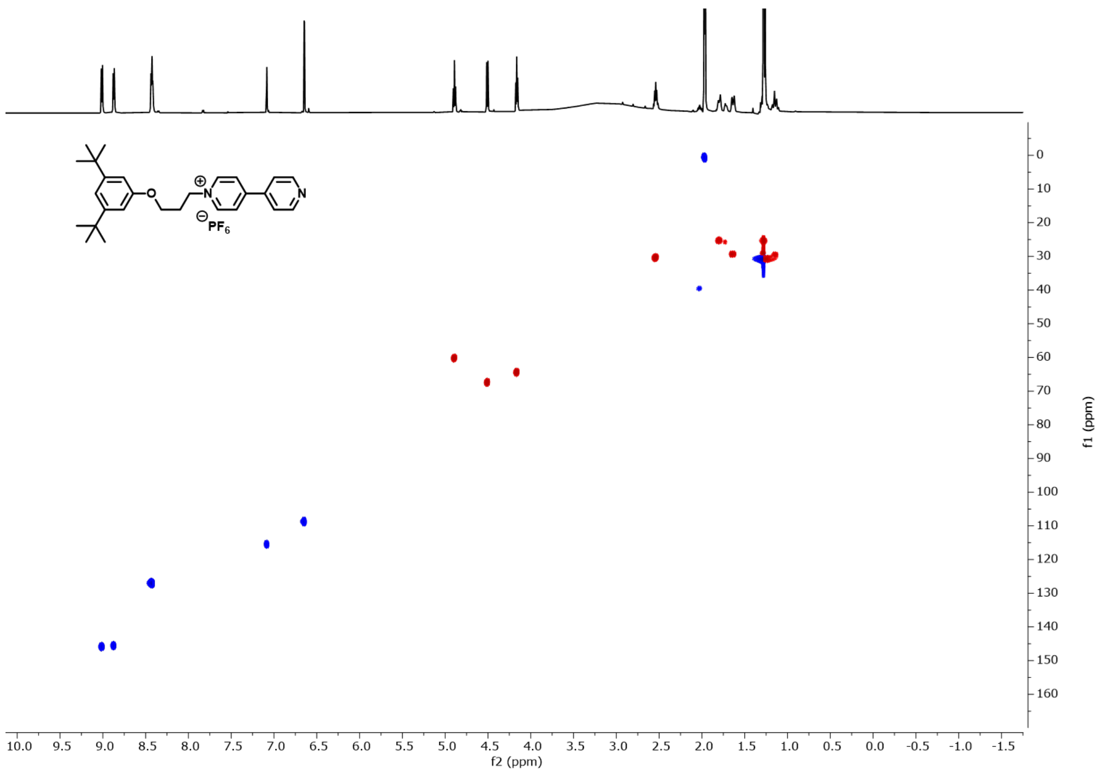

Figure SI8. ${}^{1}H$-${}^{13}C$ HSQC NMR spectrum of **2b** (${}^{1}H$: 500 MHz, ${}^{13}C$: 125 MHz, CD_3CN, 298 K).

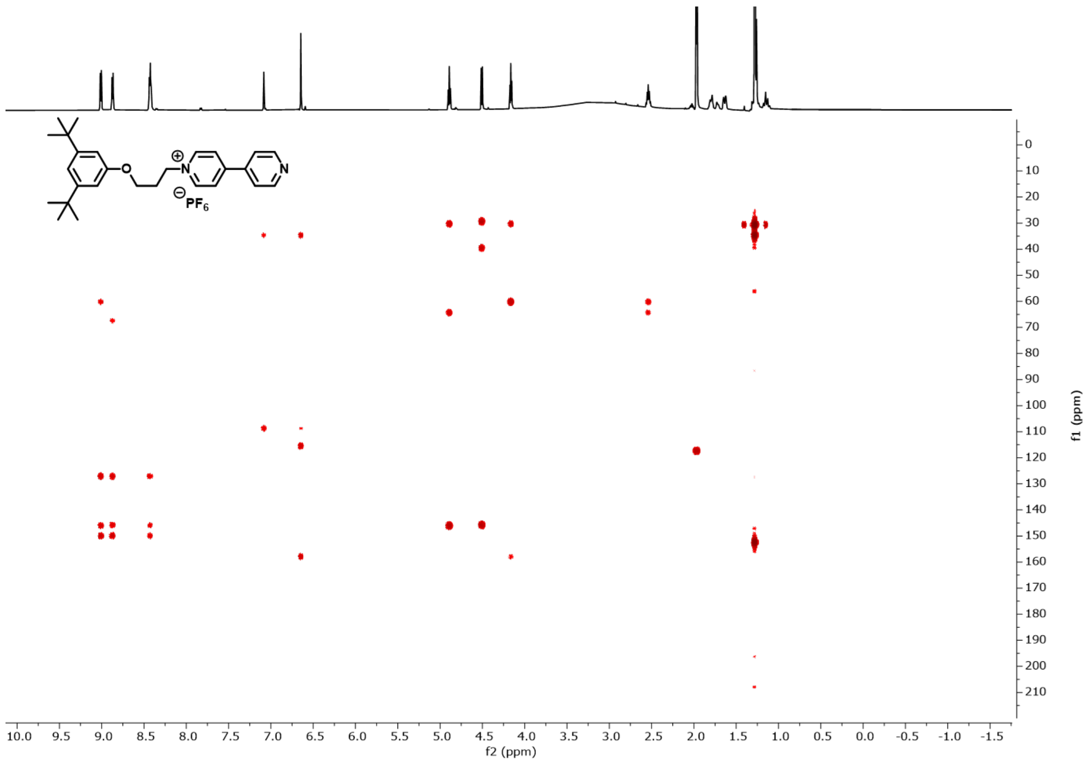

Figure SI9. ${}^{1}H$-${}^{13}C$ HMBC NMR spectrum of **2b** (${}^{1}H$: 500 MHz, ${}^{13}C$: 125 MHz, CD_3CN, 298 K).

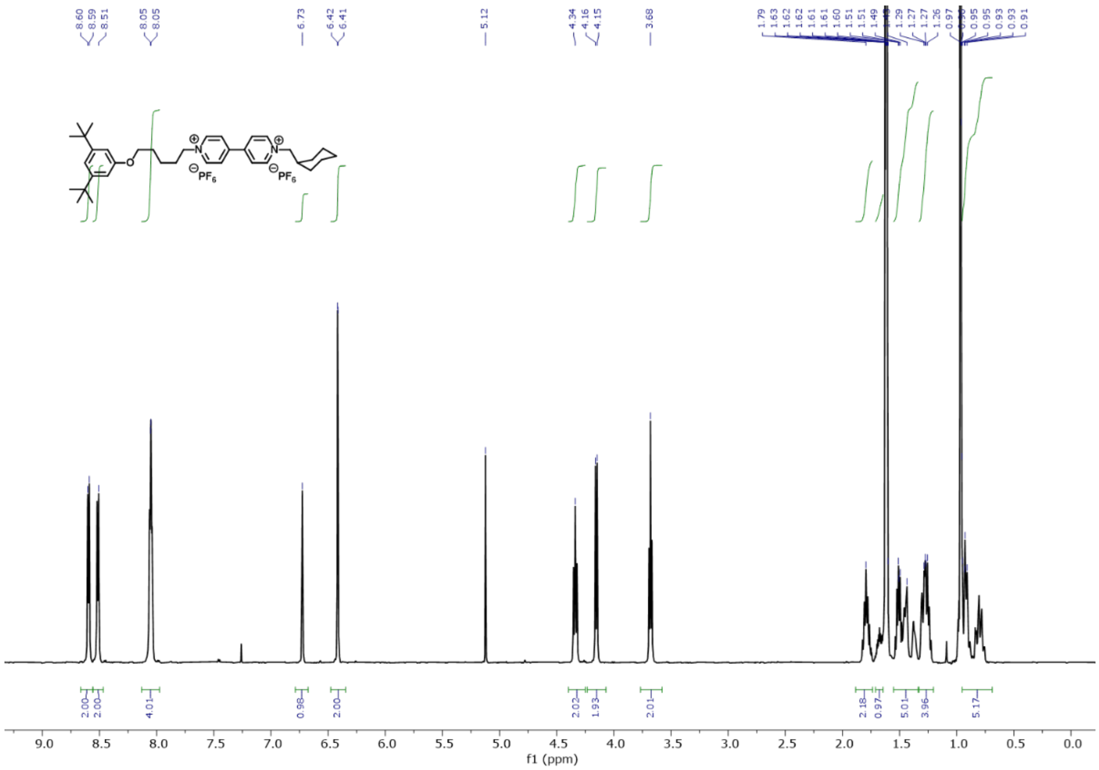

Figure SI10. ^{1}H NMR spectrum of **V1** (500 MHz, CD_3CN, 298 K).

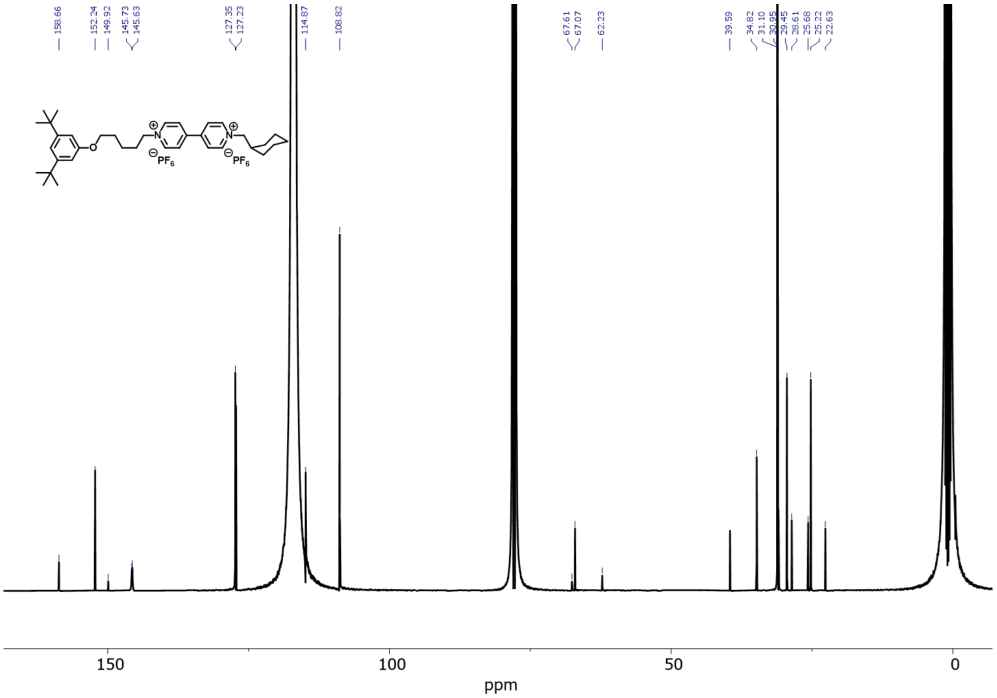

Figure SI11. ^{13}C NMR spectrum of **V1** (125 MHz, CD_3CN, 298 K).

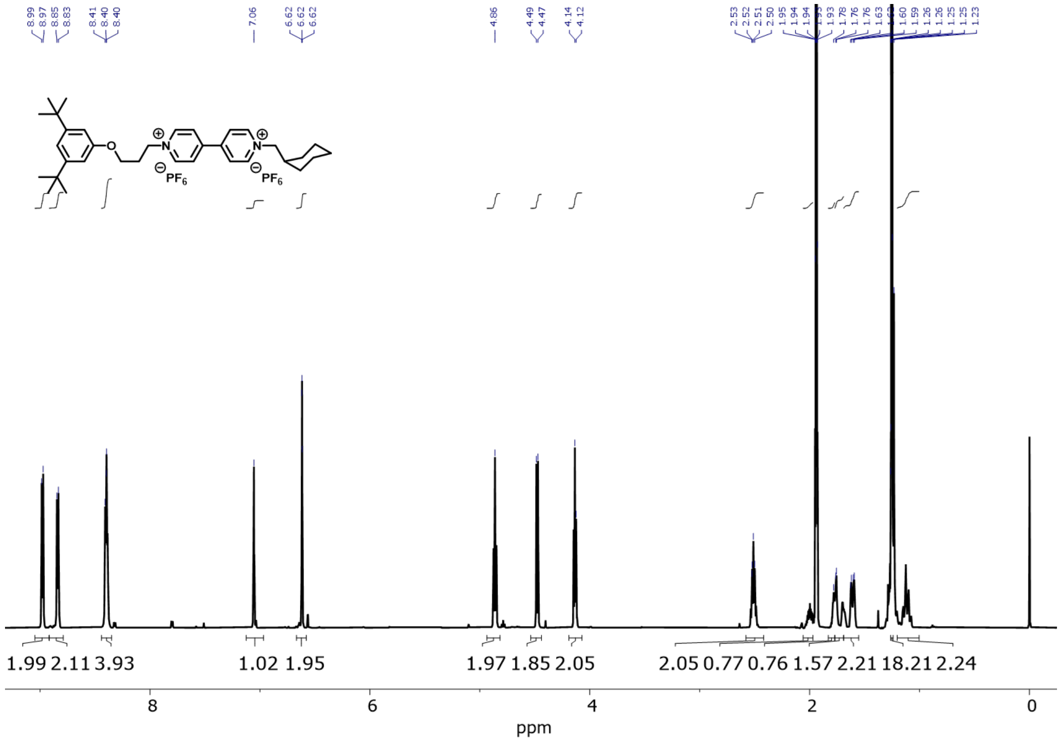

Figure SI12. ^{1}H NMR spectrum of **V2** (500 MHz, CD_3CN, 298 K).

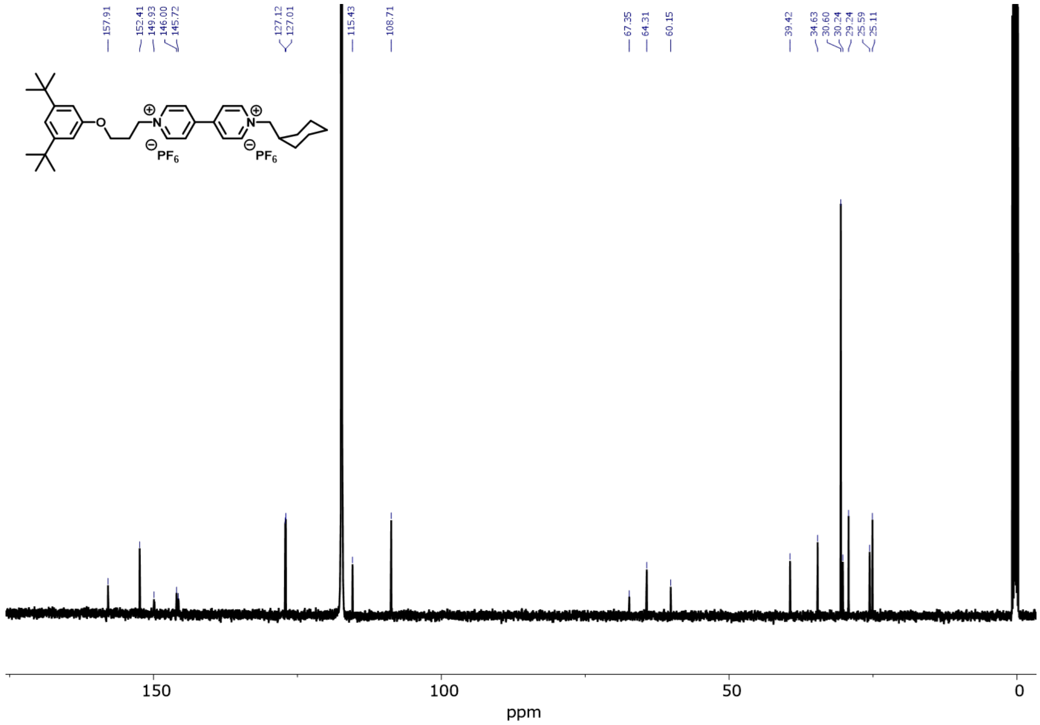

Figure SI13. ^{13}C NMR spectrum of **V2** (125 MHz, CD_3CN, 298 K).

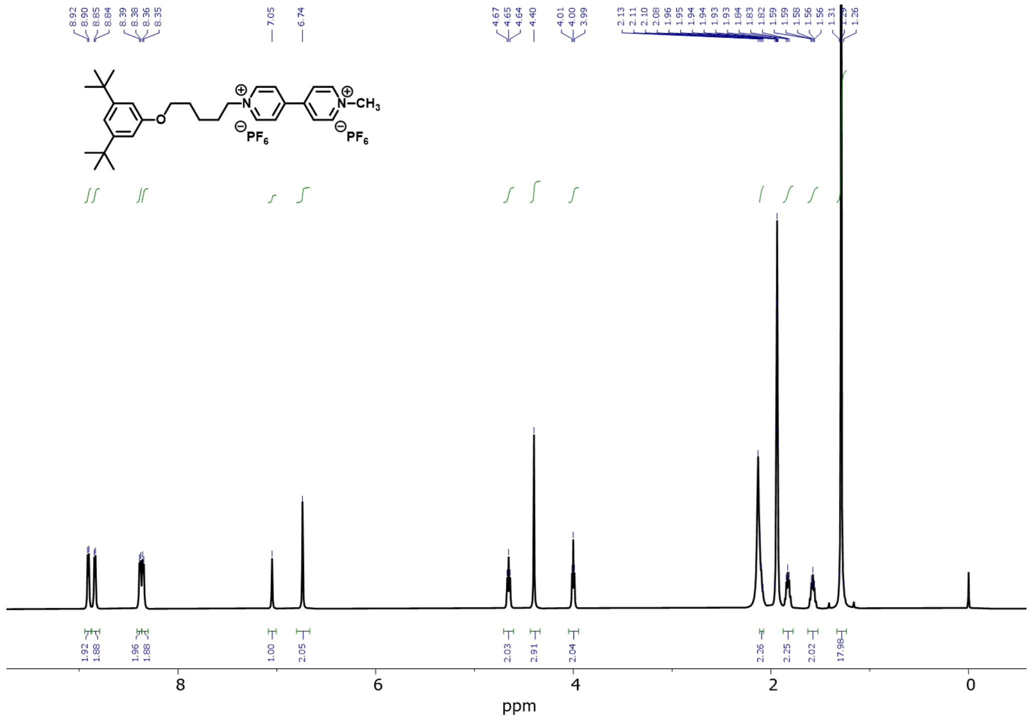

Figure SI14. ^{1}H NMR spectrum of **V3** (500 MHz, CD_3CN, 298 K).

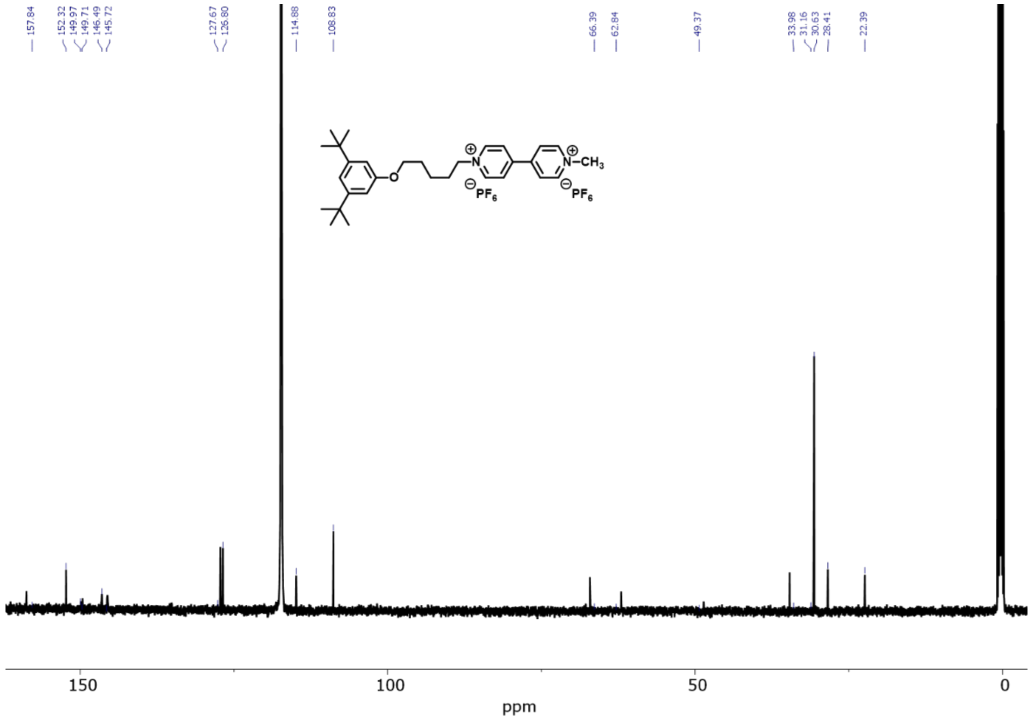

Figure SI15. ^{13}C NMR spectrum of **V3** (125 MHz, CD_3CN, 298 K).

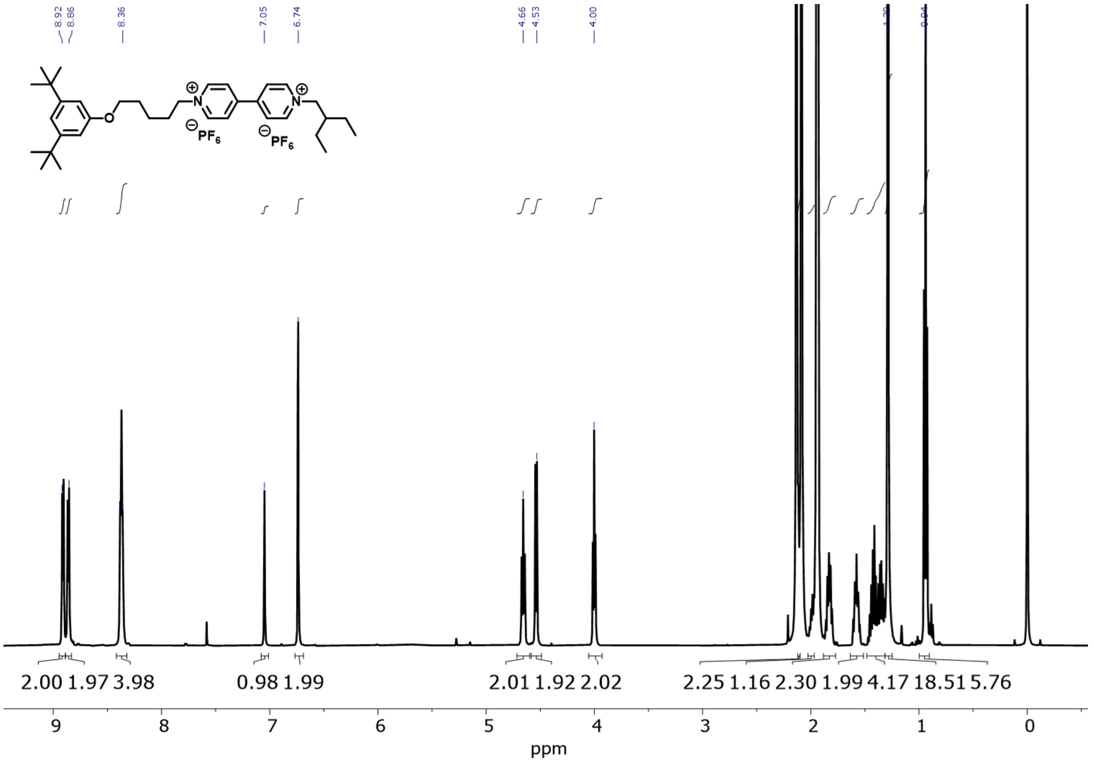

Figure SI16. ^{1}H NMR spectrum of **V4** (500 MHz, CD_3CN, 298 K).

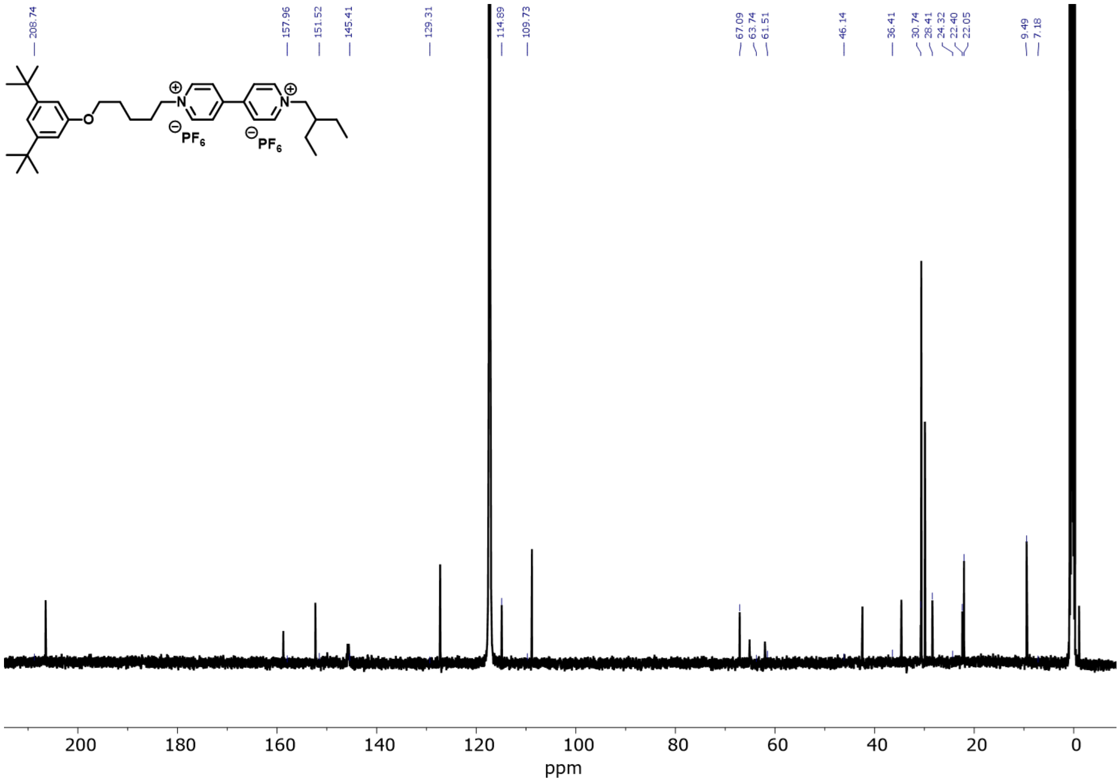

Figure SI17. ^{13}C NMR spectrum of **V4** (126 MHz, CD_3CN, 298 K).

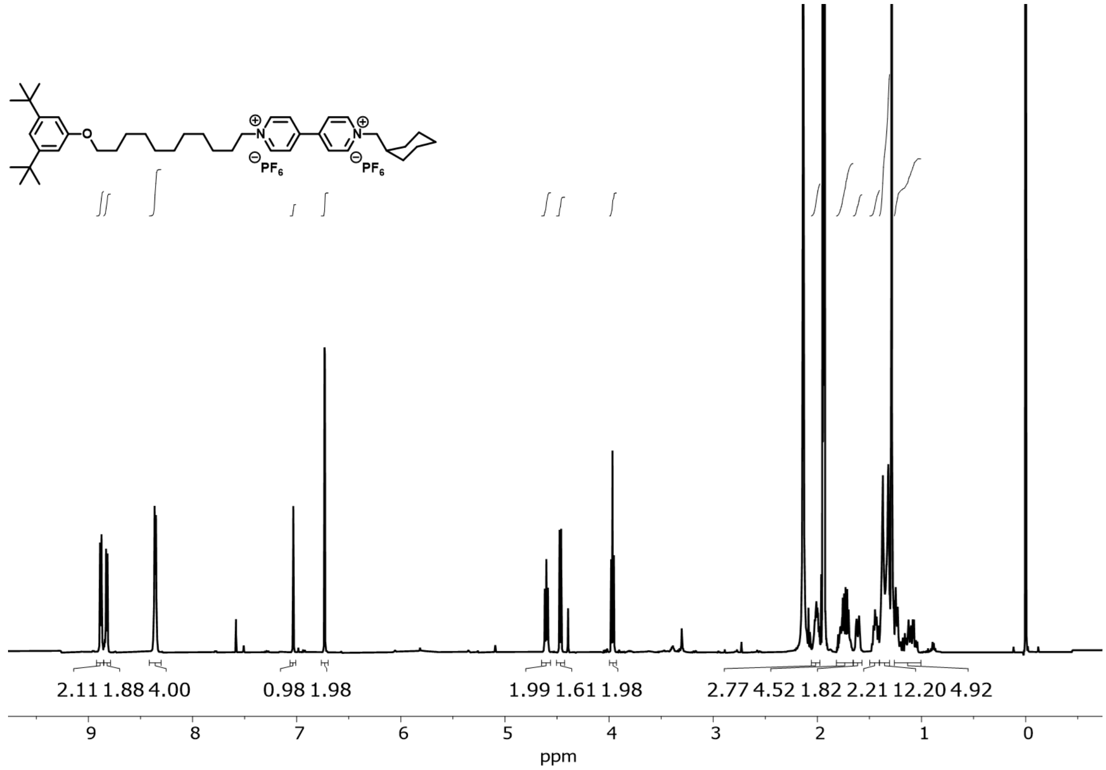

Figure SI18. ^{1}H NMR spectrum of **V5** (500 MHz, CD_3CN, 298 K).

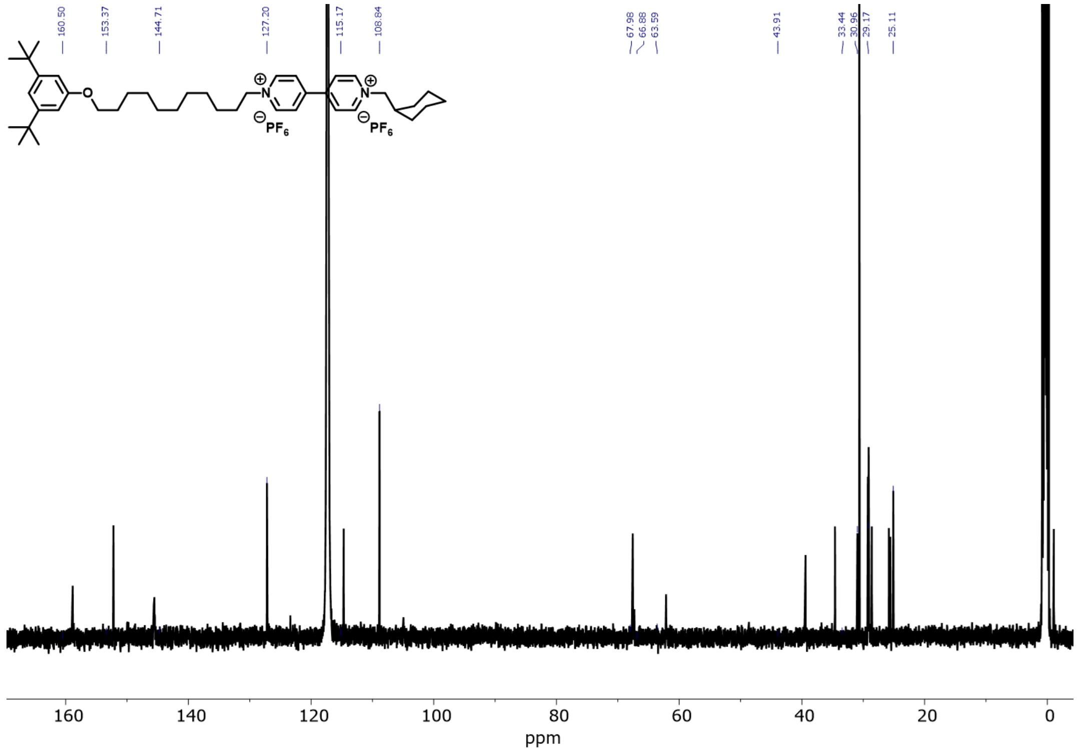

Figure SI19. ^{13}C NMR spectrum of **V5** (126 MHz, CD_3CN, 298 K).

Synthesis of porphyrin cage compounds

The syntheses of host compounds **$H_2$1**[4], **$H_2$2**[5], **$H_2$3**[5], **$H_2$4**[5] have been reported before.

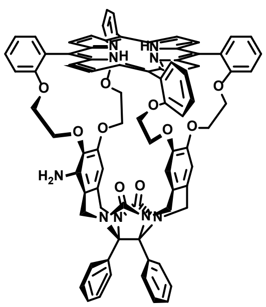

$H_2$5. A Schlenk bomb was evacuated and charged with **$H_2$2** (50 mg, 0.036 mmol) and tin(II) chloride (272.8 mg, 1.44 mmol). A solution of 4M HCl in 1,4-dioxane was added together with a drop of concentrated HCl (37%, 12M). The Schlenk bomb was sealed and heated at 70 °C for 2 hours. Afterwards, the solution was allowed to cool to room temperature and an aqueous saturated solution of sodium bicarbonate was added. The purple mixture was washed with sodium bicarbonate (2x) and brine (2x). The organic phase was collected and dried with sodium sulfate, filtered and concentrated in vacuo. The crude was precipitated from DCM and n-heptane, washed with n-pentane and dried under high vacuum to give **$H_2$5** as a purple solid in a yield of 73.5%.

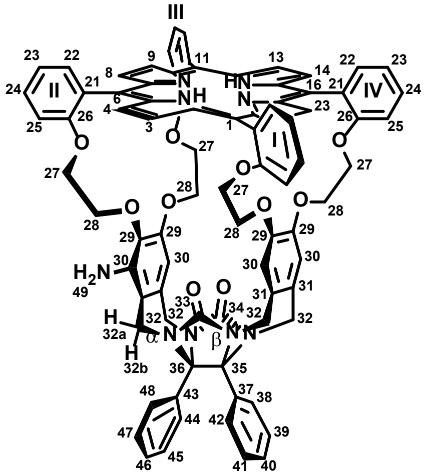

^{1}H NMR (500 MHz, chloroform-*d*): δ = 8.86 – 8.58 (m, 8H, β-pyrrole-H-**3**,**4**,**8**,**9**,**13**,**14**,**18**,**19**), 8.36 (dd, *J* = 7.40, 1.60 Hz, 1H, ArH-**22(II)**), 8.19 (dd, *J* = 7.46, 1.63 Hz, 1H, ArH-**22(IV)**), 8.03 (dd, *J* = 7.37, 1.67 Hz, 1H, ArH-**22**(**I or III**)), 7.99 (dd, *J* = 7.37, 1.64 Hz, 1H, ArH-**22**(I or III)), 7.70 – 7.81 (m, 4H, **24**), 7.48 (td, *J* = 7.47, 0.88 Hz, 1H, ArH-**23**(II)), 7.41 (td, *J* = 7.48, 0.83 Hz, 1H, ArH-**23(IV)**), 7.39 – 7.26 (m, 5H, ArH-**23(I,III)**, **25(I,III,IV)**), 7.07 – 7.02 (m, 2H, ArH-**41,45**), 7.01 – 6.95 (m, 2H, ArH-**40,46**), 6.95 – 6.81 (m, 4H, ArH-**39,42,44,47**), 6.79 – 6.73 (m, 1H, ArH-**38**), 6.28 (s, 1H, **30(III)**), 6.06 (s, 1H, **30(I)**), 5.67 (s, 1H, **30(IV)**), 4.43 (d, *J* = 16.37 Hz, 1H, CH_2-**32a(III)**), 4.30 – 4.15 (m, 6H, CH_2-**32a(I,II,IV), 27a(I,III,IV)**), 4.15 – 4.10 (m, 2H, CH_2-**27b(III,IV)**), 4.09 – 3.88 (m, 4H, CH_2-**27b(I,II)**, CH_2-**32b(III)**), 3.82 – 3.63 (m, 5H, CH_2-**32b(I,II,IV)**, CH_2-**28a(I,III)**), 3.63 – 3.56 (m, 1H, CH_2-**28(II)**), 3.56 – 3.41 (m, 2H, CH_2-**28b(III,IV)**), 3.40 – 3.33 (m, 1H, CH_2-**28b(I)**), 3.32 – 3.16 (m, 1H, CH_2-**28a(II)**), 3.15 – 3.05 (m, 1H, CH_2-**28b(II)**), -2.67 (s, 2H, PyrNH)

^{13}C{^{1}H} NMR (126 MHz, chloroform-*d*): δ = 159.17 (ArC-**26(II)**), 159.08/158.90/158.86 (ArC-**26(I,III,IV)**), 158.14 (C=O-**34**), 156.95 (C=O-**33**), 152.00 (ArC-**29(III)**), 149.36 (ArC-**29(I)**), 147.57 (ArC-**30(II)**), 146.05 (ArC-**29(IV)**), 139.57 (ArC-**29(II)**), 135.20 (ArC-**22(II)**), 135.46 (ArC-**22(IV)**), 135.91/135.72 (ArC-**22(I,III)**), 134.67 (ArC-**31(III)**), 133.41/133.25 (ArC-**37**,**43**), 132.10/132.01/131.88/131.76 (ArC-**21**), 130.78 (ArC-**31(I)**), 129.83 (ArC-**24(II)**), 129.67/129.60 (ArC-**24(I,III,IV)**), 128.60 – 127.64 (ArC-**38-42,44-48**, ArC-**31(IV)**), 120.18 (ArC-**31(II)**), 120.02 (ArC-**23(II)**), 119.89 (ArC-**23(IV)**), 119.79 (ArC-**23(I,III)**), 117.83 (ArC-**30(IV)**), 115.33/115.30/115.08 (ArC-**1**,**11**,**16**), 114.98 (ArC-**6**), 114.80 (ArC-**30(III)**), 113.66 (ArC-**30(I)**), 112.54 (ArC-**25(II)**), 112.40/112.12/111.85 (ArC-**25(I,III,IV)**), 85.22 (C-**35**), 84.95 (C-**36**), 70.34 (CH_2-**28(II)**), 68.33 (CH_2-**28(IV)**), 67.96 (CH_2-**28(III)**), 67.87 (CH_2-**27(I)**), 67.26 (CH_2-**27(III)**,**28(I)**), 66.91 (CH_2-**27(IV)**), 66.82 (CH_2-**27(II)**), 44.91 (CH_2-**32(IV)**), 44.65 (CH_2-**32(III)**), 44.29 (CH_2-**32(I)**), 38.76 (CH_2-**32(II)**

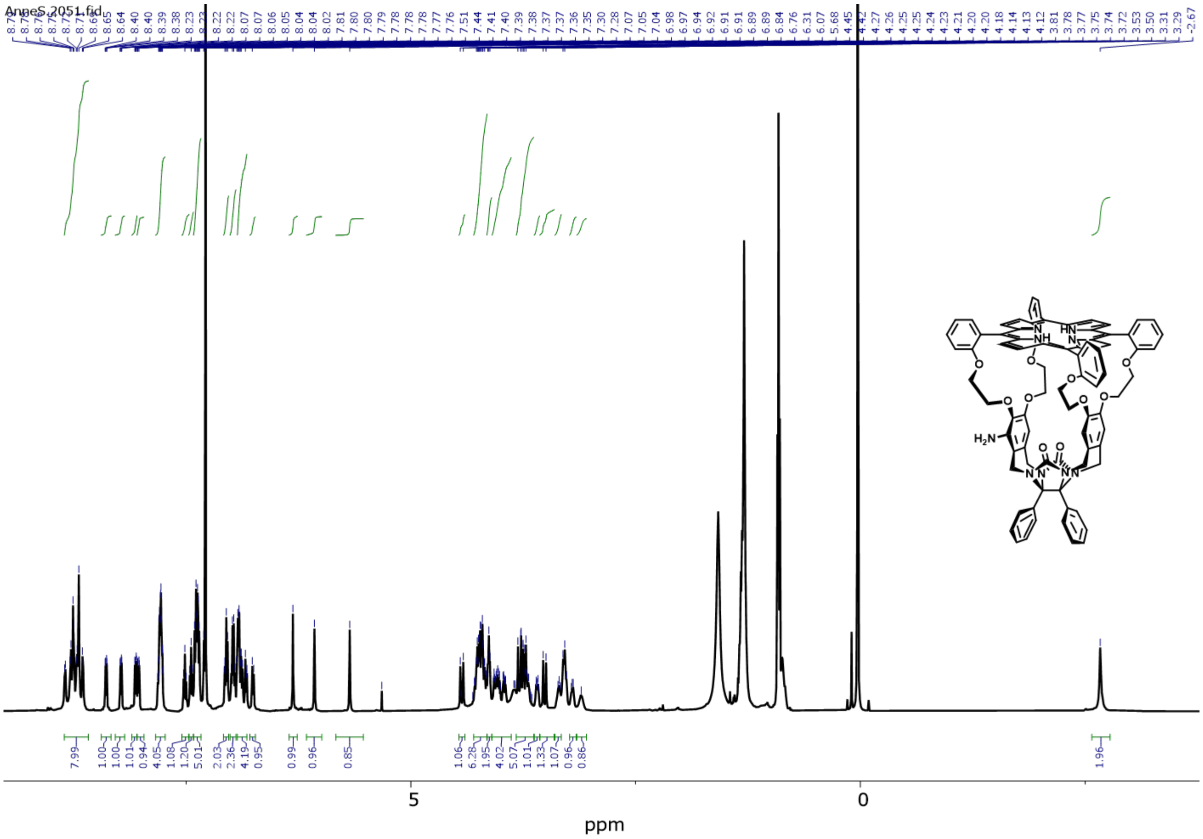

Figure SI20. ^{1}H NMR spectrum of $\mathbf{H_25}$ (500 MHz, $CDCl_3$, 298 K).

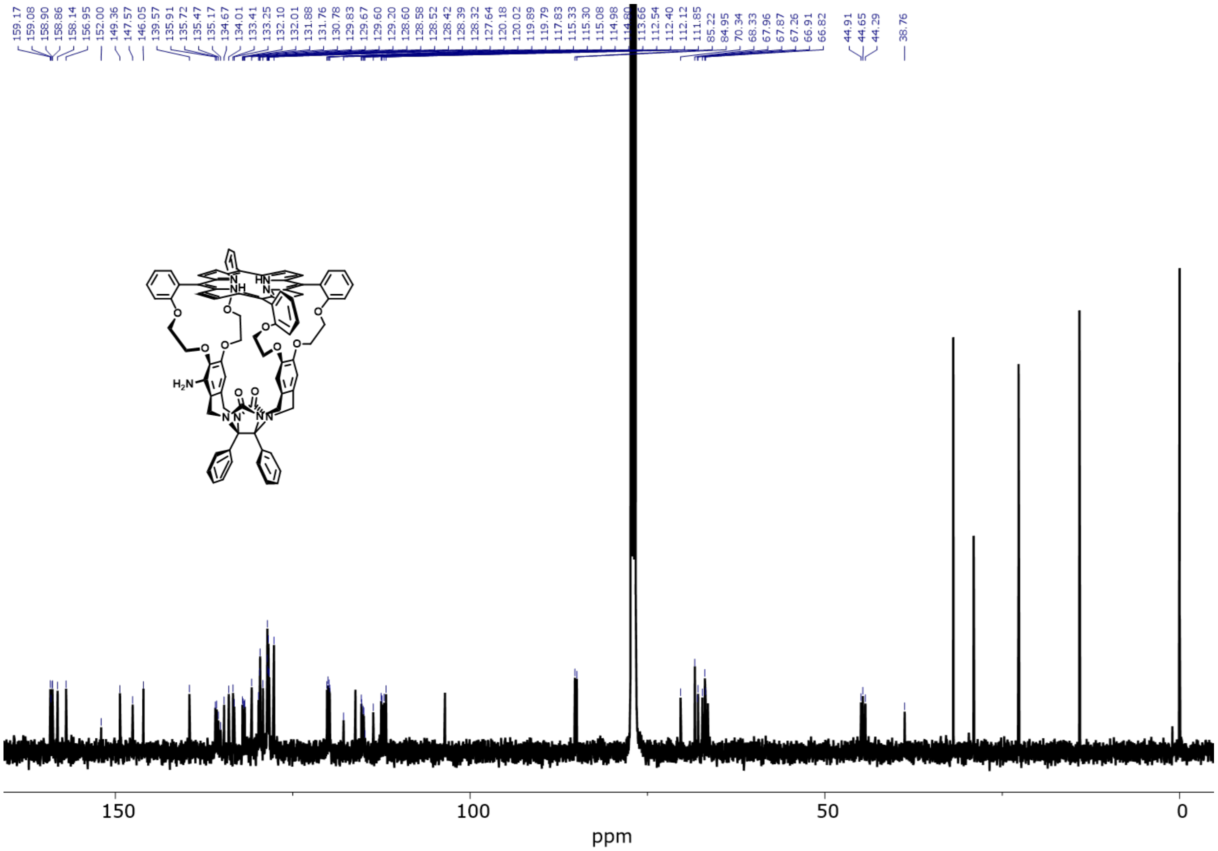

Figure SI21. ^{13}C NMR spectrum of **$H_2$5** (125 MHz, $CDCl_3$, 298 K).

Previous work

In earlier work, NMR spectra of host/guest complexes such as **$H_2$2/V1** were studied to determine the degree of unidirectional binding. Figure SI22 shows a 2D-ROESY ^1H-^1H NMR spectrum in which the exchange cross-peaks of the xylylene sidewall protons are depicted in grey, showing exchange between two species.

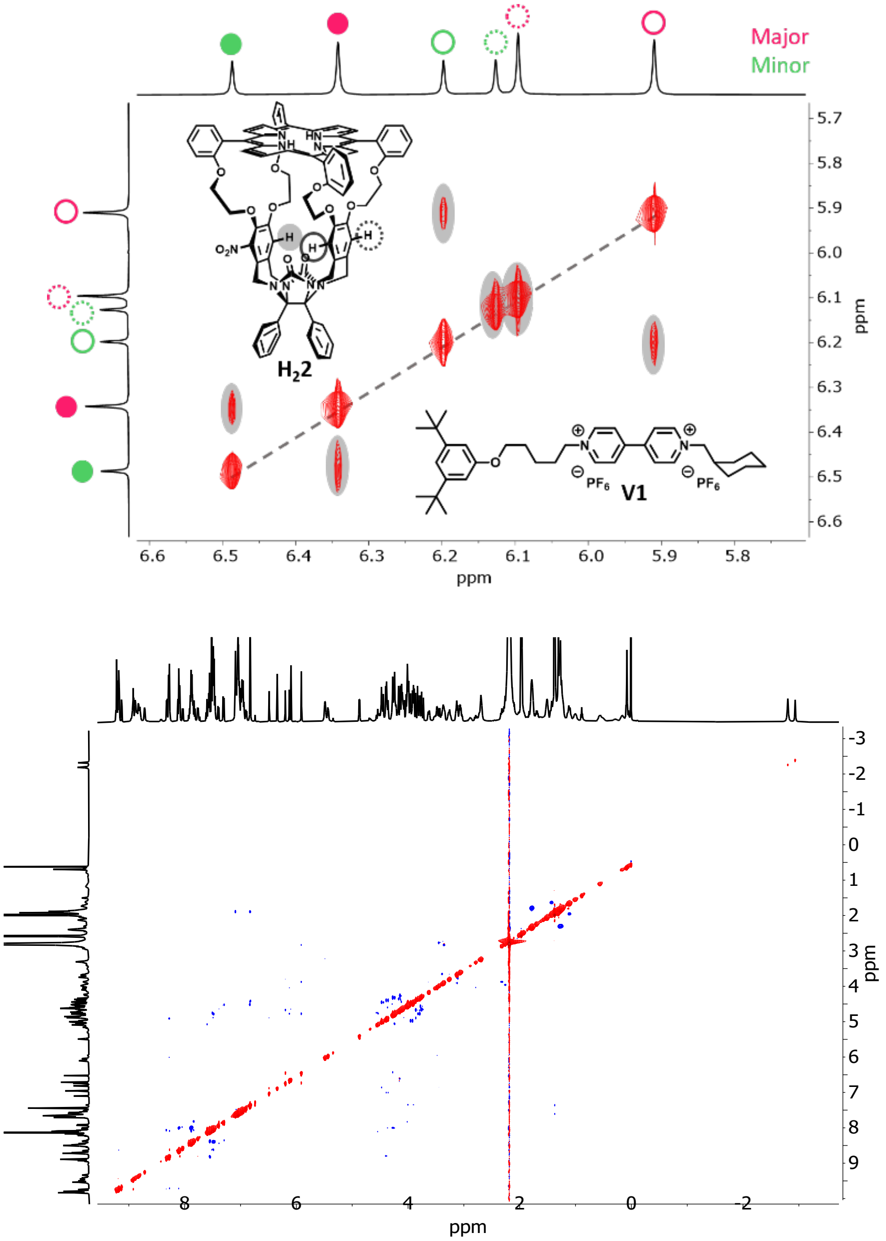

Figure SI22. 2D ROESY NMR spectra of the complex between **$H_2$2** and **V1** (1:1.5 molar ratio) in (CD_3CN:$CDCl_3$, 1:1, v/v). Top: depicting the region of the xylylene sidewall protons, which show cross-peaks caused by chemical exchange of the two different host-guest complexes (major abundant species indicated in red, minor abundant species in green); bottom: full 2D ROESY spectrum with the same peak intensity as the zoom region.

General setup and information on performed 1D EXSY experiments

The exchange processes of interest can be measured by focusing on either the NMR signals of the host or on those of the guest. In both cases, the exchange can be followed by monitoring in time the conversion between the isomeric complexes (major to minor and vice versa), or by following the host-guest system going from the free components to the bound state. We chose to focus on the NMR signals of the host because both the free host and the host in its major and minor complexes with **V1** had similar T_1 values (Figure SI24 and SI25), which simplifies the modeling of the exchange process. We decided to first monitor the exchange of the minor to the major complex (change in orientation of host) in the presence of excess guest, in order to have the maximum amount of host molecules present in a complexed form. In later experiments we monitored the exchange of the bound host to the free host and we therefore used an excess of host.

Experimental procedure for the 1D EXSY experiments involving the change in orientation of host (host: 2 mM, guest: 6 mM)

Sample preparation

Porphyrin cage compound (1.4 µmol) and internal standard pentafluorobenzaldehyde (2.8 µmol) were dissolved in 0.35 mL chloroform-*d*. To the solution, the guest (4.2 µmol) in acetonitrile-d_3 (0.35 mL) was added and the resulting host/guest mixture was transferred to a 5 mm NMR tube. The sample was capped with a 1.5 mm × 3.9 mm sleeve stopper septum and sealed with parafilm to keep the concentration constant. The samples were stored at −8 °C.

Setup of the 1D EXSY experiments

All experiments were performed on a Bruker 500 MHz Avance III spectrometer equipped with a prodigy BB cryoprobe. For each experiment, four different temperature points were used for the exchange measurements. The temperature range was selected per sample such that a suitable mix-time range could be obtained. For slower systems, higher temperatures (40 °C to 70 °C) were selected and for faster systems lower temperatures were used. Prior to each exchange measurement, the temperature of the probe was calibrated using pure ethylene glycol for temperatures ≥ 20 °C and methanol for temperatures < 20 °C. Afterwards, the probe was tuned and shimmed and the 90° pulse and the T_1 for the xylylene sidewall protons were measured for the sample at each temperature point. Then, 6-8 data points were set up as 1D NOESY experiments irradiated at the frequency of one of the xylylene sidewall protons at different mix times, followed by a quantitative ^{1}H NMR spectrum. For every temperature point, a quantitative proton spectrum was measured to ensure that the concentration of the sample stayed the same over time.

Data processing and equations

After processing and integrating the spectra, the concentrations were plotted against time and fitted according to a first-order-approaching-equilibrium equation.[6] Equation SI1 describes the decay in concentration of the selected species [$Bound_1$] and equation SI2 the growth of the formed product [$Bound_2$]. The term $[Bound_1]_0$ is calculated from the quantitative proton spectrum and $[Bound_1]_{eq}$ and $[Bound_2]_{eq}$ are calculated from equations SI3 and SI4, to give eq. SI5 and SI6.

$$[Bound_1] = [Bound_1]_0 - [Bound_1]_{eq} \cdot e^{-k_{obs} \cdot t} + [Bound_1]_{eq} \qquad \textbf{eq. SI1}$$

$$[Bound_2] = [Bound_2]_{eq} \cdot e^{-k_{obs} \cdot t} + [Bound_2]$$ **eq. SI2**

$$K_{eq} = \frac{[Bound_2]}{[Bound_1]}$$ **eq. SI3**

$$[Bound_1]_{eq} = [Bound_1]_0 - [Bound_2]_{eq}$$ **eq. SI4**

$$[Bound_2]_{eq} = \frac{K_{eq} \cdot [Bound_1]_0}{1 + K_{eq}}$$ **eq. SI5**

$$[Bound_1]_{eq} = [Bound_1]_0 - \frac{K_{eq} \cdot [Bound_1]_0}{1 + K_{eq}}$$ **eq. SI6**

After obtaining the observed rate constant (k_{obs}), k_1 and k_{-1} could be calculated by combining equation SI7 and SI8 to give SI9 and SI10.

$$k_{obs} = k_1 + k_{-1}$$ **eq. SI7**

$$K_{eq} = \frac{k_1}{k_{-1}}$$ **eq. SI8**

$$k_{-1} = \frac{k_{obs}}{K_{eq} + 1}$$ **eq. SI9**

$$k_1 = k_{obs} - \frac{k_{obs}}{K_{eq} + 1}$$ **eq. SI10**

To obtain the activation enthalpy and entropy values for the different systems, the natural log of the rate constant divided by the temperature (ln(k/T)) was plotted against the inverse temperature (eq. SI11). From the slope, the activation enthalpy can be obtained and from the intercept the activation entropy. In this equation, R is the gas constant (8.3145 $J \cdot mol^{-1} \cdot K^{-1}$), κ is the transmission coefficient (=1), k_B the Boltzmann constant ($1.3806 \cdot 10^{-23}$ $m^2 \cdot kg \cdot s^{-2} \cdot K^{-1}$) and h Planck's constant ($6.6261 \cdot 10^{-34}$ $m^2 \cdot kg \cdot s^{-1}$).

$$\ln\left(\frac{k}{T}\right) = -\frac{\Delta^{\ddagger} H^{\theta}}{R} \cdot \frac{1}{T} + \ln\left(\frac{\kappa k_B}{h}\right) + \frac{\Delta^{\ddagger} S^{\theta}}{R}$$ **eq. SI11**

Experimental procedure for 1D EXSY experiments involving the conversion of bound to free host (host: 4 mM, guest: 2 mM)

Sample preparation

Porphyrin cage compound (2.8 µmol) and internal standard pentafluorobenzaldehyde (2.8 µmol) were dissolved in 0.35 mL chloroform-*d*. To this solution, guest (1.4 µmol) in acetonitrile-d_3 (0.35 mL) was added and the resulting host/guest mixture was transferred to a 5 mm NMR tube. The sample was capped with a 1.5 mm × 3.9 mm sleeve stopper septum and sealed with parafilm to keep the concentration constant. The samples were stored at −8 °C. In order to obtain the potential energy diagram, for **$H_2$3** the studies did not have to be repeated for an excess of host because the host is symmetric. Hence, the energy barrier calculated earlier can be used for constructing the potential energy diagram. This was confirmed by repeating the studies with symmetric host **$H_2$1** (Figure SI29 and SI30). No significant differences between the energy barriers in both studies were obtained.

Setup of the 1D EXSY experiments

The setup was the same as previously described (p16). However, in this the case mix times (d8) were chosen such that the conversion from bound host to free host was not more than 10-20%, allowing initial rates to be calculated.

Data processing and equations

Since only data points for a conversion of bound to free host of around 10-20% are required, the data was fitted according to first order kinetics from the initial-rates approximation.[7] Hence, the rate could be directly obtained from the slope of the decay of the concentration of the bound host. Consequently, the rate constant was obtained by dividing the rate by the concentration of the bound host (eqs. SI12 and SI13).

$$rate = k[Bound] \qquad \textbf{eq. SI12}$$

$$\frac{rate}{[Bound]} = k \qquad \textbf{eq. SI13}$$

From the Eyring equation, the Gibbs free energies of activation could be calculated (eq. SI14). Here, k is the rate constant and T the temperature in Kelvin, κ the transmission coefficient (=1), k_B the Boltzmann constant ($1.3806\cdot10^{-23}$ $m^2\cdot kg\cdot s^{-2}\cdot K^{-1}$) and h Planck's constant ($6.6261\cdot10^{-34}$ $m^2\cdot kg\cdot s^{-1}$).

$$ln\left(\frac{k}{T}\right) = -ln\left(\frac{\kappa k_B}{h}\right)\frac{\Delta^{\ddagger}G^{\theta}}{RT} \qquad \textbf{eq. SI14}$$

1D EXSY Pulse sequence

The pulse sequence has been developed by Dr. Charles Fry at the University of Wisconsin-Madison to be similar to the Varian Chempack NOESY1D experiment but operative on Bruker instruments. The core elements of the pulse sequence are two gradient-enhanced Gaussian-shaped 180° refocusing pulses following the initial hard 90° pulse. The magnetization is then stored longitudinally via another hard 90° after which the mix time occurs whereby magnetization is transferred either by NOE or chemical exchange. During the mixing time, a series of gradient and shaped adiabatic chirp pulses are applied to clean-up artifacts, unless the mix time drops below 75 ms, at which point these extra pulses are skipped due to their duration and simple delay period is used instead. A read-out pulse is applied at the end of the mixing time for detection.

The selection of the peak is made through integrating the peak of interest in a 1D 1H spectrum, and the shaped pulse duration and power is determined from the calibrated 90° using the "sel1d" au routine in Topspin. The "sel1d" au was modified to refer to this other pulse sequence and its parameter set rather than the default Bruker sequence and parameter set.

Pulse Sequence used in all 1D EXSY studies:

```
;noesy1d.UW is near-identical to NOESY1D vnmr6.1c ChemPack 3.1 version
;avance-version (02/05/31)
;1D NOESY using selective refocussing with two shaped pulses
;dipolar coupling may be due to noe or chemical exchange.
;according to noesy1d.c vnmr6.1ca
;  thanks to CGA 2013June for initial coding
;  20130716: some changes commenting and param set cgfry
;
;H. Kessler, H. Oschkinat, C. Griesinger & W. Bermel,
;   J. Magn. Reson. 70, 106 (1986)
;J. Stonehouse, P. Adell, J. Keeler & A.J. Shaka, J. Am. Chem. Soc 116,
;   6037 (1994)
```

```
;K. Stott, J. Stonehouse, J. Keeler, T.L. Hwang & A.J. Shaka,
;   J. Am. Chem. Soc 117, 4199-4200 (1995)
;
;$CLASS=HighRes Extra
;$DIM=1D
;$TYPE=
;$SUBTYPE=
;$COMMENT=


#include <Avance.incl>
#include <Grad.incl>

#define zero ph=0.0


"p2=p1*2"


;"d20=d8*0.31-p16*2-p32*0.5-d16-8u"
;"d21=d8*0.49-p16*3-p32-d16*2-6u"
;"d22=d8*0.2-p16*2-p32*0.5-d16-3u"

"spoff29=0"

#   ifdef CALC_SPOFFS
"spoff2=bf1*(cnst21/1000000)-o1"
#   else
#   endif /*CALC_SPOFFS*/


"acqt0=-p1*2/3.1416"


1 ze
2 30m
  20u pl1:f1 BLKGRAD
# ifdef SPOIL
  50u UNBLKGRAD
  p16:gp0
  d16
  p1 zero
  5u
  p16:gp0
  d16
  d12 BLKGRAD
# endif
# ifdef PRESAT
  d12 pl9:f1
  d1 cw:f1 ph1
  d12 do:f1
  5u pl1:f1
# else
  d1
#endif
  50u UNBLKGRAD
  (p1 ph1):f1
```

```
  3u
  p16:gp1
  d16 pl0:f1
  p12:sp2:f1 ph2:r
  3u
  p16:gp1
  d16
  3u
  p16:gp2
  d16 pl0:f1
  p12:sp2:f1 ph3
  3u
  p16:gp2
  d16 pl1:f1
  (p1 ph1):f1
/* mixing time */
  5u
  p16:gp3
  d20
  p16:gp4
  d16
  (p32:sp29 zero):f1
  3u
  p16:gp4*-1
  d16
  d21
  p16*2:gp4*-1
  d16
  3u pl0:f1
  (p32:sp29 zero):f1
  3u
  p16*2:gp4
  d16
  d22 pl1:f1
/* if the mix time is <75 ms then comment out above section and uncomment the
line below */
/*  d8 pl1:f1 */
/* end mixing */
  (p1 ph1):f1
  go=2 ph31
  30m mc #0 to 2 F0(zd)
  20u BLKGRAD
exit

/* original phases
ph1=0 2
ph2=0 0 1 1 2 2 3 3
ph3=0 0 0 0 0 0 0 0 1 1 1 1 1 1 1 1
    2 2 2 2 2 2 2 2 3 3 3 3 3 3 3 3
ph4=0
ph5=0
ph6=0
ph31=0 2 2 0 0 2 2 0 2 0 0 2 2 0 0 2
*/
/* phases according to noes1dy.c */
ph1=0 0 0 0 0 0 0 0 0 0 0 0 0 0 0 0
    1 1 1 1 1 1 1 1 1 1 1 1 1 1 1 1
```

```
    2 2 2 2 2 2 2 2 2 2 2 2 2 2 2 2
    3 3 3 3 3 3 3 3 3 3 3 3 3 3 3 3
ph2=0 1 2 3 0 1 2 3 0 1 2 3 0 1 2 3
    1 2 3 0 1 2 3 0 1 2 3 0 1 2 3 0
    2 3 0 1 2 3 0 1 2 3 0 1 2 3 0 1
    3 0 1 2 3 0 1 2 3 0 1 2 3 0 1 2
ph3=0 0 0 0 1 1 1 1 2 2 2 2 3 3 3 3
    1 1 1 1 2 2 2 2 3 3 3 3 0 0 0 0
    2 2 2 2 3 3 3 3 0 0 0 0 1 1 1 1
    3 3 3 3 0 0 0 0 1 1 1 1 2 2 2 2
ph31=0 2 0 2 2 0 2 0 0 2 0 2 2 0 2 0
     1 3 1 3 3 1 3 1 1 3 1 3 3 1 3 1
     2 0 2 0 0 2 0 2 2 0 2 0 0 2 0 2
     3 1 3 1 1 3 1 3 3 1 3 1 1 3 1 3


;pl0 : 120dB
;pl1 : f1 channel - power level for pulse (default)
;sp2: f1 channel - shaped pulse
;sp29: f1 channel - shaped pulse (adiabatic)
;p1 : f1 channel -  90 degree high power pulse
;p2 : f1 channel - 180 degree high power pulse
;p12: f1 channel - 180 degree shaped pulse
;p16: homospoil/gradient pulse                            [1 msec]
;p32: f1 channel - 180 degree shaped pulse (adiabatic)    [20 msec]
;     smoothed chirp (sweepwidth, 20% smoothing, 10000 points)
;d1 : relaxation delay; 2-5 * T1
;d8 : mixing time from 0.05 to ~T1 for small molecules
;d16: delay for homospoil/gradient recovery
;cnst21: chemical shift for selective pulse (offset, in ppm) set by sel1d au
;NS: 8 * n, ALWAYS USE A MULTIPLE OF 8 SCANS, total number of scans: NS * TD0
;DS: 4


;current setup places no requirements on O1 (within 5ppm or so),
;  but selection width currently must >10Hz (or topspin produces an error)


;use gradient ratio:    gp 1 : gp 2 : gp 3 : gp 4 : -gp 4
;                         13 :   11 :   17 :   40 :  -40

;for z-only gradients:
;gpz1: 13%
;gpz2: 11%
;gpz3: 17%
;gpz4: 40%

;use gradient files:
;gpnam1: SMSQ10.100
;gpnam2: SMSQ10.100
;gpnam3: SMSQ10.100
;gpnam4: SMSQ10.100
```

Experimental procedure for the fluorescence binding titrations

Sample preparation

For all fluorescence titrations anhydrous chloroform (>99%) was used and acetonitrile was dried and distilled over $CaCl_2$. Before use, the chloroform was filtered over dry K_2CO_3. Then, a 1:1 mixture of acetonitrile and chloroform was prepared and used as the solvent mixture. For each titration of host with guest, a measured amount (between 1 and 2 mg) of host was weighed and dissolved in the solvent mixture. From the concentrated host stock solution (H_{stock}), three measuring solutions, in order to perform the studies in triplo, were prepared with a concentration of 3.0 µM in 10 mL of solvent mixture. Furthermore, a known amount of guest (around 5 mg) was weighed and dissolved in the solvent mixture (G_{stock}). From G_{stock} a titration solution (10 mL) was prepared containing 3 µM of host and 80 µM of guest. The titration experiments were started by measuring the fluorescence of the measuring solution solely containing host. Afterwards, aliquots of the titration solution were added with increasing equivalents of guest to measure the decay in fluorescence signal. An overview of the added aliquots of titration solution can be found on p19.

Fluorescence titrations

Fluorescence quenching titrations were performed using a JASCO FP-8300ST Spectrofluorometer and a quartz cuvette with 1 cm path length. The baseline was recorded using the solvent mixture. The sample was irradiated with 419 nm light, after which the fluorescence was measured from 800 nm to 400 nm. The fluorescence titration was performed in triplo.

Data processing

The measured spectra were loaded into SpectraGryph® and the fluorescence intensities at 650 nm and 716 nm were extracted. The binding constant was calculated by loading the change in the intensity of the fluorescence against the concentration of guest into BindFit (http://app.supramolecular.org/bindfit/)[8] assuming a 1:1 binding process. The fits are given on p23. The free Gibbs free energy was calculated with the help of eq. SI15.

$$\Delta G^{\theta} = -RTln(K_a) \qquad \textbf{eq. SI15}$$

The individual binding constants for the conversion of free host to the major species and the free host to the minor species could be calculated from considering the observed association constant as a weighted average (eq. SI16). By combining eqs. SI16 and eq. SI17, eqs. SI18 and SI19 could be derived for calculating $K_{a,major}$ and $K_{a,minor}$.

$$K_{eq} = \frac{[major]}{[minor]} = \frac{[K_{a_{major}}]}{[K_{a_{minor}}]} \qquad \textbf{eq. SI16}$$

$$K_{avg} = \alpha K_{a_{major}} + \beta K_{a_{minor}} \text{ in which } \alpha = \frac{[major]}{[total\ bound]} \text{ and } \beta = \frac{[minor]}{[total\ bound]} \qquad \textbf{eq. SI17}$$

$$K_{a_{minor}} = \frac{K_{avg}}{\alpha K_{eq}+\beta} \qquad \textbf{eq. SI18}$$

$$K_{a_{major}} = \alpha \left(K_{avg} - \frac{\beta K_{avg}}{\alpha K_{eq}+\beta}\right) \qquad \textbf{eq. SI19}$$

Table SI1. Titration table showing the added amounts of guest solution for the fluorescence binding measurements. Each system was studied in triplo for which thirty datapoints each were measured, going up to ~11.6 equivalents of guest. The first measurement contains no guest and 2500 µL of host solution (3 µM), after which aliquots of the guest stock solution are added with each measurement. The number of equivalents is calculated according to the system that is studied, keeping the addition table constant. The system shown below is **$H_2$2/V1**.

Measurement #	Equivalents guest	To add from titration solution [µL]
1	0	0
2	0.106013805	10
3	0.211606919	10
4	0.31678267	10
5	0.421544343	10
6	0.525895186	10
7	0.732972734	20
8	0.938451228	20
9	1.142355174	20
10	1.344708519	20
11	1.54553467	20
12	1.744856504	20
13	1.942696392	20
14	2.42827057	50
15	2.905142701	50
16	3.373619197	50
17	3.833990562	50
18	4.286532483	50
19	4.731506813	50
20	5.169162486	50
21	5.599736352	50
22	6.023453947	50
23	6.4405302	50
24	7.249328526	100
25	8.034268494	100
26	8.796717346	100
27	9.537928069	100
28	10.25905178	100
29	10.96114848	100
30	11.64519642	100

Error propagation calculation

Error propagation rules were followed for calculating the error, where e is the error and C is a constant[9].

Table SI2. Used error propagation equations

Function	Uncertainty
$y = x_1 + x_2$	$e_y = \sqrt{e_{x_1}^2 + e_{x_2}^2}$
$y = x_1 - x_2$	$e_y = \sqrt{e_{x_1}^2 + e_{x_2}^2}$

Function	Uncertainty
$y = \ln(x)$	$e_y = \frac{e_x}{x}$
$y = \frac{x}{C}$	$e_y = \frac{e_x}{C}$

Construction of potential energy diagrams

The potential energy diagrams for the different host/guest systems was constructed from several measurements and calculations, as shown in Figure SI23. The energy barrier for the conversion of the major species to the unbound host was directly derived from the 1D EXSY measurements (in blue). For all systems, the energy barrier for the conversion of the major species to the free host is lower in energy than the energy barrier for the conversion of the minor species to the free host. Using this information, the energy barrier for the conversion of the minor species to the free host (in black) could be calculated from the overall energy barrier of the major to the minor species, which was directly measured by the 1D EXSY studies (orange). The ground state differences between the free and bound host were derived from the fluorescence binding titrations. By using the K_{eq} (=$K_{maj/min}$, green) from the 1D EXSY studies, the differences in ground state energies of the major and free species, and the minor species and the free host could be calculated (pink). Eventually, the energy barrier for the conversion of the minor species to the free host (black) could be calculated from the energy difference resulting from the K_{eq} (green) between the ground states of the major and the minor species and subtracting this from the overall energy barrier for the conversion of the major to the minor species (orange).

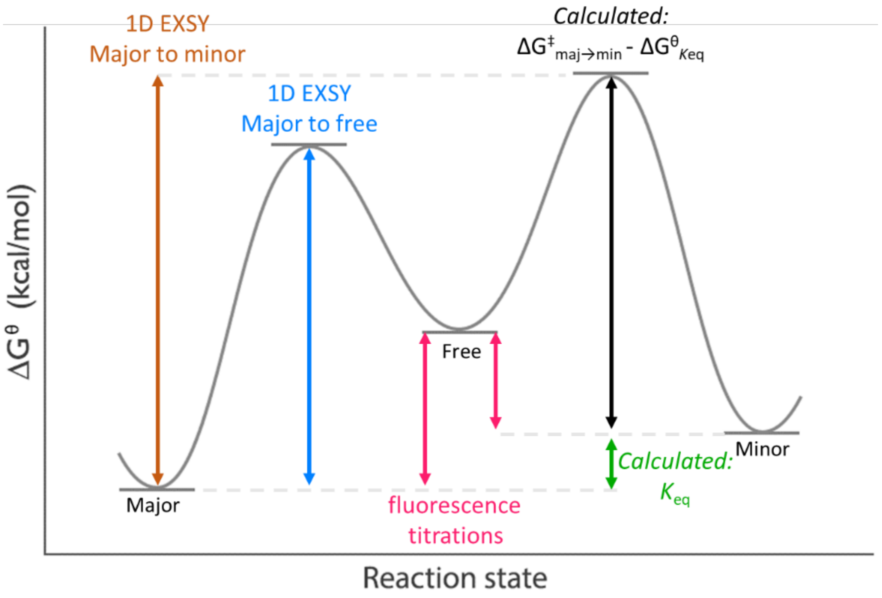

Figure SI23. Schematic representation of the measured and calculated energy barriers, which could be combined to construct a potential energy diagram.

T_1 determination and an overview of the 1D EXSY NMR studies

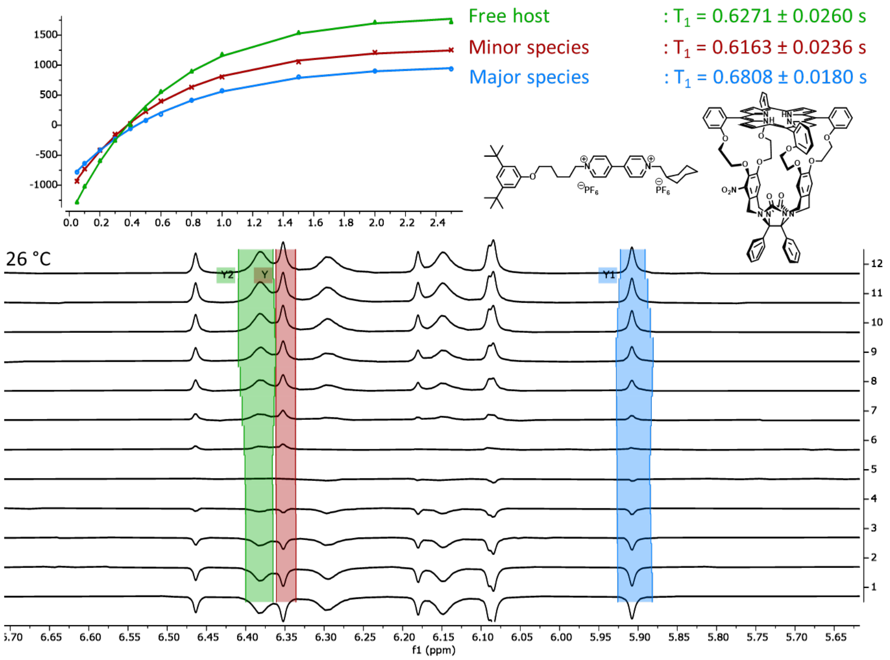

Figure SI24. The calculated T_1 relaxation delays calculated through inverse-recovery for the free host and bound host (guest: **V1**), demonstrating the difference between these delays. (500 MHz, guest:host = 1:2, 2 mM: 4 mM, $CDCl_3$:CD_3CN, 1:1, v/v) at 26 °C.

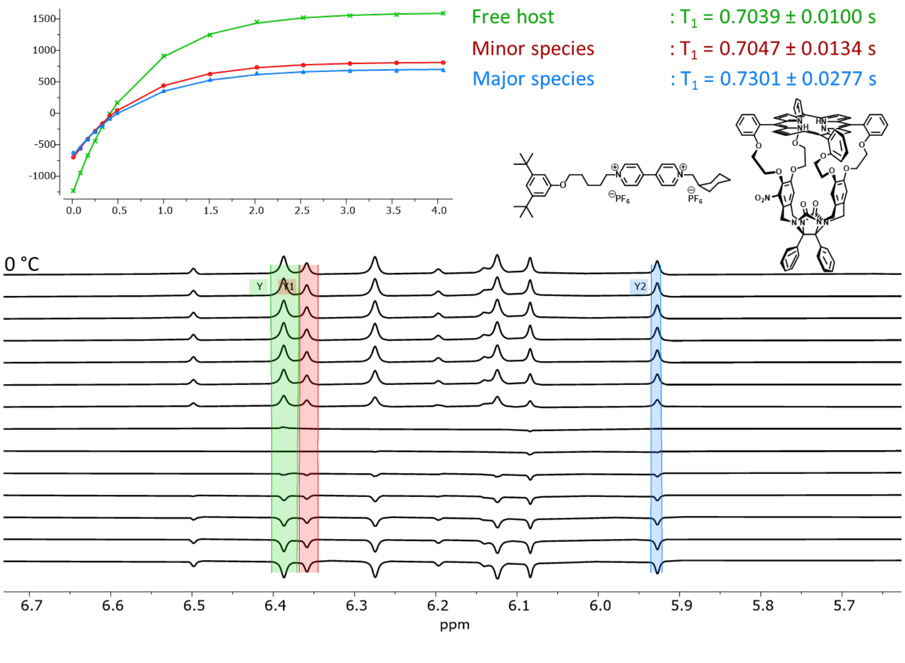

Figure SI25. The calculated T_1 relaxation delays calculated through inverse-recovery for the free host and bound host (guest: **V1**), demonstrating the difference between these delays. (500 MHz, guest:host = 1:2, 2 mM: 4 mM, $CDCl_3$:CD_3CN, 1:1, v/v) at 0 °C in the (near) absence of exchange.

Table SI3. Calculated values of the activation enthalpy ($\Delta H^‡$), activation entropy ($\Delta S^‡$) and the activation Gibbs free energy ($\Delta G^‡$) for different hosts and guests together with K_{eq}, which is the ratio of major to minor product (host: 2 mM, guest: 6 mM).

Host (Guest: V1)	$\Delta H^‡$ (kcal·mol^{-1})	$\Delta S^‡$ (cal·mol^{-1})	$\Delta G^‡$ (kcal·mol^{-1})	$K_{maj/min}$
**$H_2$1**	13.36 ± 0.52	−20.67 ± 0.85	19.81 ± 0.43	-
**$H_2$2 (k_{maj})**	12.53 ± 0.88	−21.23 ± 1.84	19.18 ± 1.05	2
**$H_2$2 (k_{min})**	11.87 ± 0.73	−21.90 ± 1.95	18.73 ± 0.95	
**$H_2$3**	10.30 ± 0.41	−21.28 ± 0.69	16.97 ± 0.46	-
**$H_2$4 (k_{maj})**	15.97 ± 0.37	−13.65 ± 0.24	20.24 ± 0.38	4
**$H_2$4 (k_{min})**	14.38 ± 0.20	−15.62 ± 0.29	19.27 ± 0.91	
**$H_2$5 (k_{maj})**	10.53 ± 0.96	−30.35 ± 2.29	20.03 ± 1.20	1.5
**$H_2$5 (k_{min})**	10.85 ± 0.44	−28.67 ± 1.97	19.82 ± 0.76	

Guest (Host: $H_2$2)	$\Delta H^‡$ (kcal·mol^{-1})	$\Delta S^‡$ (cal·mol^{-1})	$\Delta G^‡$ (kcal·mol^{-1})	$K_{maj/min}$
**V1 (k_{maj})**	12.53 ± 0.88	−21.23 ± 1.84	19.18 ± 1.05	2
**V1 (k_{min})**	11.87 ± 0.73	−21.91 ± 1.95	18.73 ± 0.95	
**V2 (k_{maj})**	10.51 ± 0.39	−26.03 ± 1.14	18.66 ± 0.53	1.2
**V2 (k_{min})**	10.69 ± 0.31	−25.07 ± 1.05	18.54 ± 0.45	
**V3 (k_{maj})**	8.87 ± 0.41	−19.57 ± 0.58	15.00 ± 0.45	2
**V3 (k_{min})**	9.61 ± 0.21	−18.03 ± 0.51	15.25 ± 0.26	
**V4 (k_{maj})**	10.96 ± 0.40	−24.06 ± 1.08	18.50 ± 0.52	1.9
**V4 (k_{min})**	9.98 ± 0.34	−25.79 ± 1.25	18.06 ± 0.52	
**V5 (k_{maj})**	11.96 ± 1.08	−22.60 ± 0.50	19.04 ± 1.09	2.0
**V5 (k_{min})**	12.68 ± 0.18	−19.16 ± 0.37	18.68 ± 0.22	

Overview of the results of the fluorescence titration experiments

Table SI4. The calculated binding constants and Gibbs free energy values (ΔG^θ) for different hosts and guests (host: 3 mM).

Host	K_{bind} (M^{-1})	ΔG^θ (kcal/mol)
**$H_2$1**	8.48×10^6 ± 2.08×10^6	−9.93 ± 0.15
**$H_2$2** ($K_{bind,maj}$)	1.24×10^6 ± 4.22×10^4	−8.32 ± 0.02
**$H_2$2** ($K_{bind,min}$)	6.42×10^5 ± 2.18×10^4	−8.73 ± 0.02
**$H_2$3**	8.65×10^4 ± 1.17×10^3	−7.07 ± 0.01
**$H_2$4** ($K_{bind,maj}$)	7.09×10^5 ± 3.04×10^3	−8.38 ± 0.14
**$H_2$4** ($K_{bind,min}$)	1.77×10^5 ± 1.21×10^4	−7.52 ± 0.13
**$H_2$5** ($K_{bind,maj}$)	3.69×10^7 ± 1.23×10^7	−10.80 ± 3.60
**$H_2$5** ($K_{bind,min}$)	2.54×10^7 ± 8.44×10^6	−10.60 ± 3.52

Guest	K_{bind} (M^{-1})	ΔG^θ (kcal/mol)
**V1** ($K_{bind,maj}$)	1.24×10^6 ± 4.22×10^4	−8.32 ± 0.02
**V1** ($K_{bind,min}$)	6.42×10^5 ± 2.18×10^4	−8.73 ± 0.02
**V2** ($K_{bind,maj}$)	4.21×10^5 ± 2.04×10^4	−7.95 ± 0.04
**V2** ($K_{bind,min}$)	3.51×10^5 ± 1.70×10^4	−8.06 ± 0.04
**V3** ($K_{bind,maj}$)	9.98×10^5 ± 3.20×10^4	−8.60 ± 0.02
**V3** ($K_{bind,min}$)	4.99×10^5 ± 1.60×10^4	−8.16 ± 0.02
**V4** ($K_{bind,maj}$)	5.05×10^5 ± 1.90×10^4	−8.17 ± 0.31
**V4** ($K_{bind,min}$)	2.73×10^5 ± 1.03×10^4	−7.79 ± 0.29
**V5** ($K_{bind,maj}$)	1.40×10^6 ± 3.56×10^4	−8.81 ± 0.22
**V5** ($K_{bind,min}$)	7.02×10^5 ± 1.78×10^4	−8.38 ± 0.21

Fluorescence binding studies fits

Table SI5. The URLs referring to the bind fits at http://supramolecular.org/ for all triplo measurements at 650 nm and 716 nm.

Host (Guest: V1)	715 nm	650 nm
H_21	**1:** http://app.supramolecular.org/bindfit/view/0ef79b10-b5dc-4751-af9c-912a8f6a4b67<br>**2:** http://app.supramolecular.org/bindfit/view/1497f121-2be1-4bdc-b4b4-425da9aa19a5<br>**3:** http://app.supramolecular.org/bindfit/view/46a0d970-cfe8-45a5-b33a-82bc86618b95	**1:** http://app.supramolecular.org/bindfit/view/a447541f-1881-4256-b106-c8b8c71fdf95<br>**2:** http://app.supramolecular.org/bindfit/view/55e2be53-c99d-4a92-8821-c1e03982adab<br>**3:** http://app.supramolecular.org/bindfit/view/18ed8697-b46f-4e2b-87cb-8dbb2fc220cd
H_22	**1:** http://app.supramolecular.org/bindfit/view/16c1aa86-733b-487e-9dff-3ea27fea63eb<br>**2:** http://app.supramolecular.org/bindfit/view/2afc9820-df4d-431b-bd1c-918ffc335ee3<br>**3:** http://app.supramolecular.org/bindfit/view/1cea438a-5bed-4ee7-93eb-17b266113f7c	**1:** http://app.supramolecular.org/bindfit/view/2ade3c86-c29b-4362-a019-32b85431ab1b<br>**2:** http://app.supramolecular.org/bindfit/view/c172f11a-22d7-47b4-b1a2-30a2a051d2bb<br>**3:** http://app.supramolecular.org/bindfit/view/84c43939-f462-4c94-b505-3b5f589c5c6d
H_23	**1:** http://app.supramolecular.org/bindfit/view/358ffa91-e3af-4e62-91df-1016c0116519<br>**2:** http://app.supramolecular.org/bindfit/view/9ac5ea5a-4a04-4762-944c-441cbeddab4b<br>**3:** http://app.supramolecular.org/bindfit/view/8ace631c-66ae-4a99-8bd8-00a35caef525	**1:** http://app.supramolecular.org/bindfit/view/22372af4-1283-465a-b358-cc81b42658c5<br>**2:** http://app.supramolecular.org/bindfit/view/1cd282c1-613b-4350-a3b6-3b70840d06f4<br>**3:** http://app.supramolecular.org/bindfit/view/81df5d28-02ba-4edc-9904-ffc234508e1d
H_24	**1:** http://app.supramolecular.org/bindfit/view/1c60ff03-b026-4526-b2fc-ea2bbd5abcb4<br>**2:** http://app.supramolecular.org/bindfit/view/bf3fd11d-1cb6-4f87-beba-9e7b5934ea5b<br>**3:** http://app.supramolecular.org/bindfit/view/801a071b-3162-46f2-aa4f-5e87daf0a0d4	**1:** http://app.supramolecular.org/bindfit/view/27c05d3c-0a54-4bf1-b49b-dc36c04c7239<br>**2:** http://app.supramolecular.org/bindfit/view/a13467d3-f43f-44c8-b109-7f03a45fe66f<br>**3:** http://app.supramolecular.org/bindfit/view/2515a6c7-e719-426b-bac0-704b1e9ba2ba
H_25	**1:** http://app.supramolecular.org/bindfit/view/59c32951-ec2c-4b28-bf3c-97b364013b1c<br>**2:** http://app.supramolecular.org/bindfit/view/6ef347b2-8685-46d2-80cc-2314e32badbd<br>**3:** http://app.supramolecular.org/bindfit/view/188d9430-5bad-4982-8a02-07ad617cc3eb	**1:** http://app.supramolecular.org/bindfit/view/a0472778-7c11-4452-8918-e7b67d4a2c6d<br>**2:** http://app.supramolecular.org/bindfit/view/2ebc91e2-15f9-4d21-aa0d-2639da33b573<br>**3:** http://app.supramolecular.org/bindfit/view/32db33d0-290d-490d-a328-1270236f4129

Guest (Host: H_22)	715 nm	650 nm
V1	**1:** http://app.supramolecular.org/bindfit/view/16c1aa86-733b-487e-9dff-3ea27fea63eb<br>**2:** http://app.supramolecular.org/bindfit/view/2afc9820-df4d-431b-bd1c-918ffc335ee3<br>**3:** http://app.supramolecular.org/bindfit/view/1cea438a-5bed-4ee7-93eb-17b266113f7c	**1:** http://app.supramolecular.org/bindfit/view/2ade3c86-c29b-4362-a019-32b85431ab1b<br>**2:** http://app.supramolecular.org/bindfit/view/c172f11a-22d7-47b4-b1a2-30a2a051d2bb<br>**3:** http://app.supramolecular.org/bindfit/view/84c43939-f462-4c94-b505-3b5f589c5c6d
V2	**1:** http://app.supramolecular.org/bindfit/view/a1a4fc35-c1d3-4806-9ce6-00f4c8dbbd71<br>**2:** http://app.supramolecular.org/bindfit/view/2c3c70d8-7f00-497e-8faf-318e71a46092<br>**3:** http://app.supramolecular.org/bindfit/view/b284c4b8-eac8-4f83-bd6c-7a07e13122dd	**1:** http://app.supramolecular.org/bindfit/view/cbfc7960-c878-4e6b-9909-10b82222c8fb<br>**2:** http://app.supramolecular.org/bindfit/view/69cd466a-24d3-4578-8da1-1299d068be8f<br>**3:** http://app.supramolecular.org/bindfit/view/5003350e-58e5-4ea6-9f5c-ef755698a966
V3	**1:** http://app.supramolecular.org/bindfit/view/4607bf9c-7f5f-4307-b104-58c30dee31ab<br>**2:** http://app.supramolecular.org/bindfit/view/2288b294-3d0f-4eed-b521-1f601e9734de<br>**3:** http://app.supramolecular.org/bindfit/view/da4ed179-8371-4684-8e6d-47b37a550af4	**1:** http://app.supramolecular.org/bindfit/view/54a45aea-df90-43a7-8f58-aef0c9bd2b13<br>**2:** http://app.supramolecular.org/bindfit/view/b47b9803-ab03-4c1b-bfb2-089d2217ab1d<br>**3:** http://app.supramolecular.org/bindfit/view/18b01753-8817-47b5-9f04-dd398c95ed63
V4	**1:** http://app.supramolecular.org/bindfit/view/2c26fafb-44f7-446f-88a8-a0c39ea80f12<br>**2:** http://app.supramolecular.org/bindfit/view/317dfbb4-6e54-4d59-9dd7-e1020265b3e3<br>**3:** http://app.supramolecular.org/bindfit/view/9ef5b718-05ea-4729-a284-416159777aa8	**1:** http://app.supramolecular.org/bindfit/view/5ad88942-753e-4ad4-8464-a04005616416<br>**2:** http://app.supramolecular.org/bindfit/view/19d4a4be-e7d0-49b2-a494-4903c1781c98<br>**3:** http://app.supramolecular.org/bindfit/view/5be78dfb-21f8-412a-b848-3ed2a449aa8d
V5	**1:** http://app.supramolecular.org/bindfit/view/0f4020c1-812e-4dea-94d2-282ba4d11248<br>**2:** http://app.supramolecular.org/bindfit/view/de0a3ccb-2944-4e86-984f-b0f07569879d<br>**3:** http://app.supramolecular.org/bindfit/view/3ce79043-c2b5-4f1f-9289-979cf1d3ac57	**1:** http://app.supramolecular.org/bindfit/view/8baf5f46-321a-4916-bd6f-78fff98230a8<br>**2:** http://app.supramolecular.org/bindfit/view/abad00ac-bffe-45ae-958f-9bf4444e7fee<br>**3:** http://app.supramolecular.org/bindfit/view/aa885b73-f94f-4f80-aa62-4fd021eea253

Spartan calculations

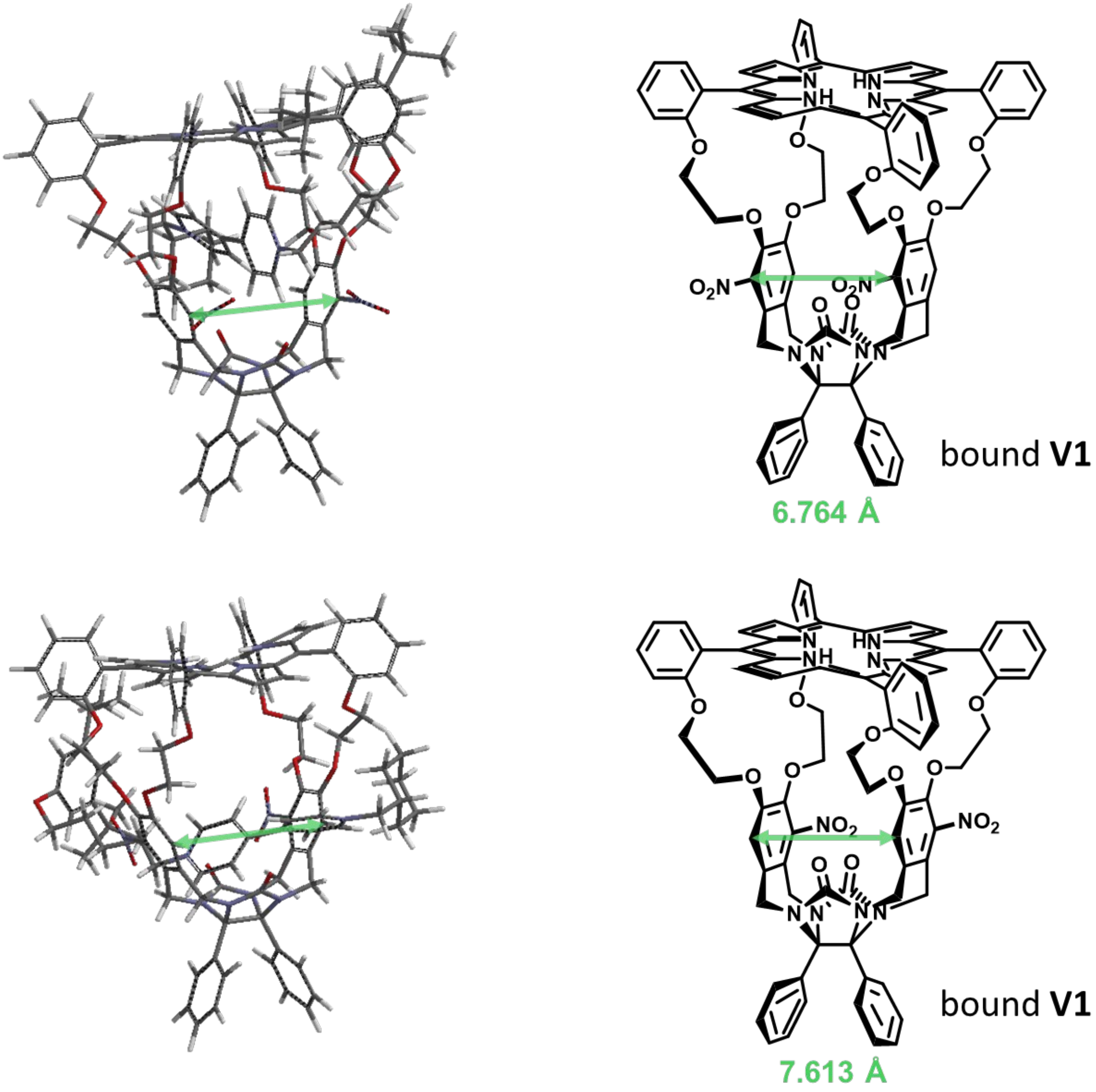

Figure SI26. Calculated molecular model (calculated with equilibrium geometry at ground state in gas with semi-empirical AM1) of the complex between **$H_2$4** and **V1** (left, view on the two faces of the cage compound) and corresponding molecular structures (right); the green arrows indicate the distances between the sidewall carbon atoms. These calculations reveal that the distance between the two xylylene sidewalls is larger on the side opposite to the nitro-groups (7.613 Å) than on the side where the nitro groups are located (6.764 Å).

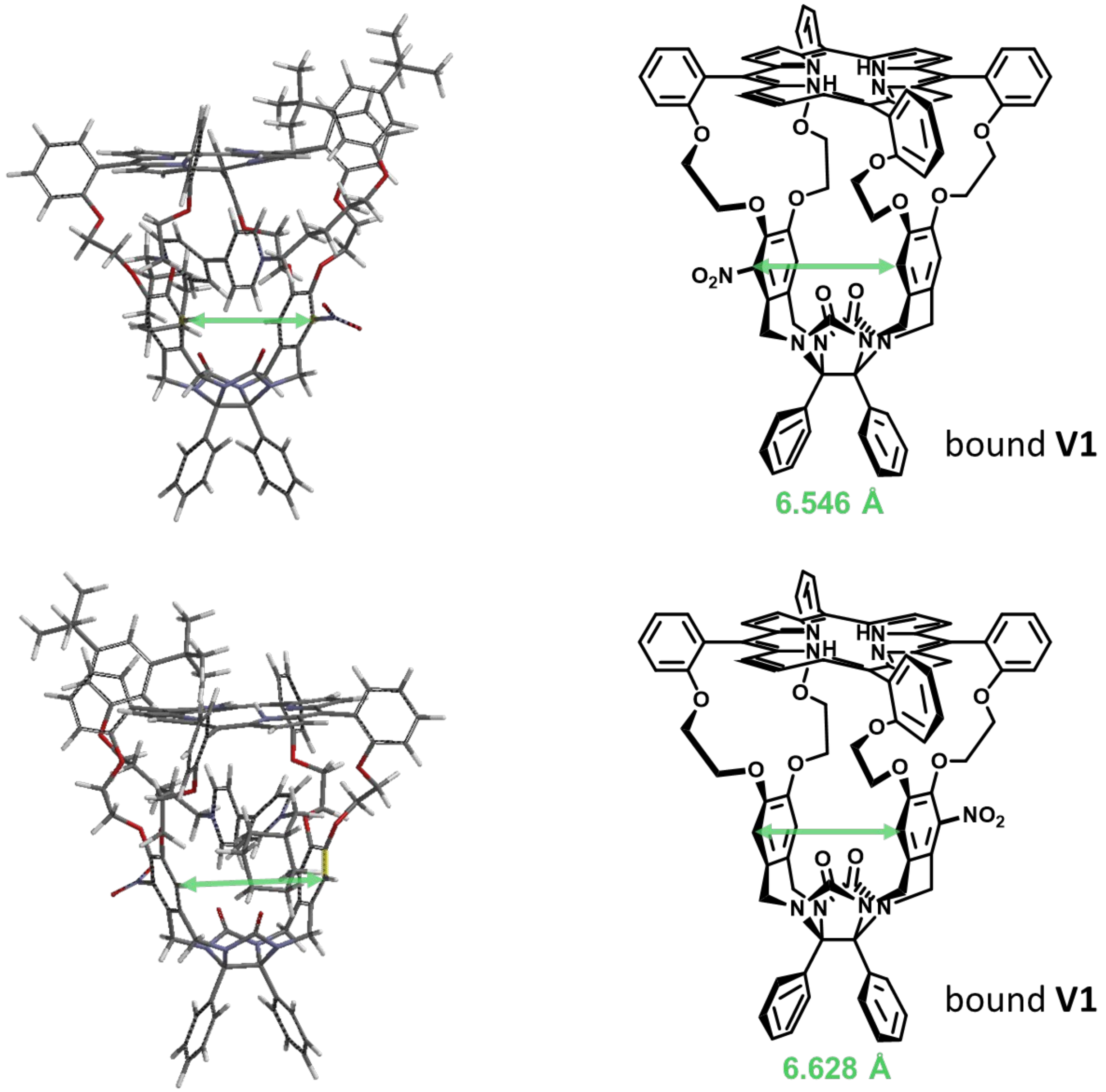

Figure SI27. Calculated molecular model (calculated with equilibrium geometry at ground state in gas with semi-empirical AM1) of the complex between **$H_2$2** and **V1** (left, view on the two faces of the cage compound) and corresponding molecular structures (right); the green arrows indicate the distances between the sidewall carbon atoms. These calculations show that the distance between the two xylylene sidewalls is slightly larger on the side opposite to the nitro-group (6.628 Å) than on the side where the nitro-group (6.546 Å) is located. This difference (0.082 Å) is significantly smaller for **$H_2$2/V1** than for **$H_2$4/V1** (0.849 Å), which suggests pinching of the guest in the latter case.

1D EXSY studies with polymer (VP) and $H_2$2

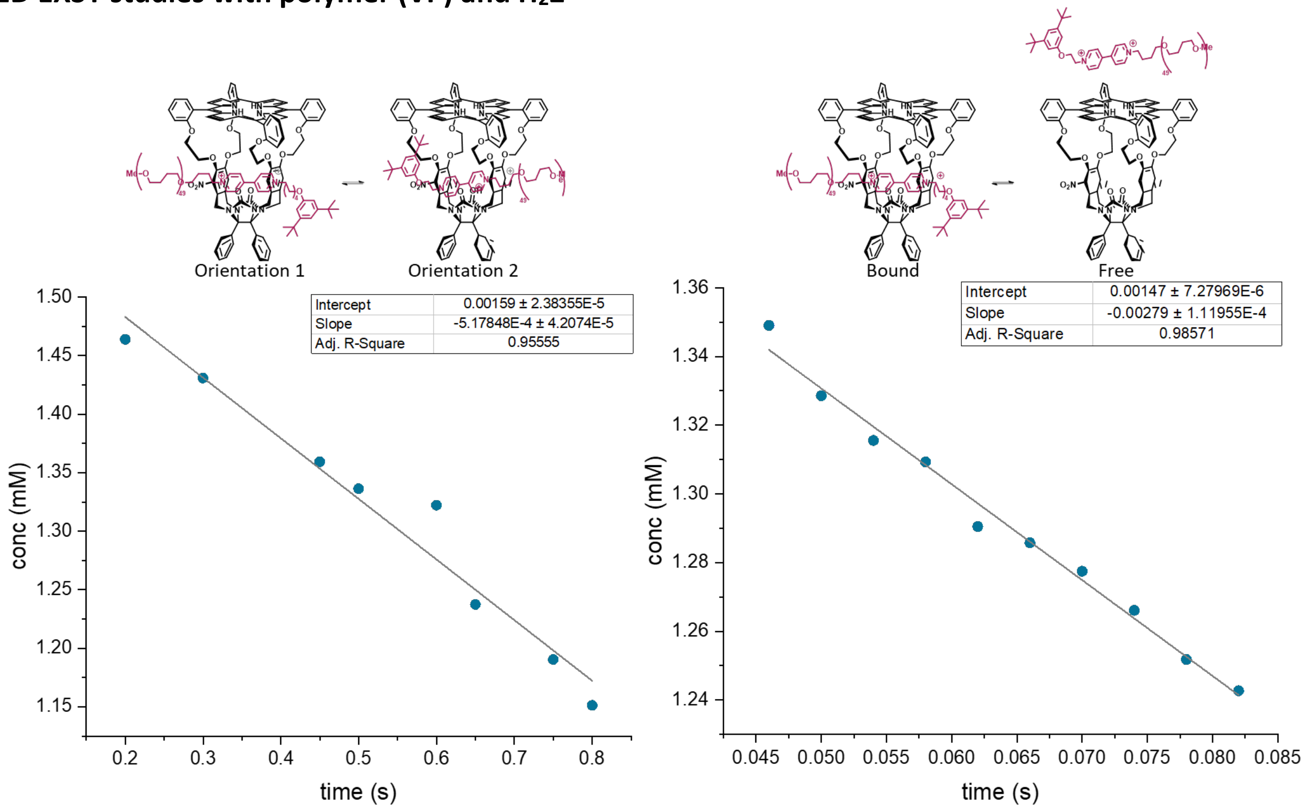

Figure SI28. 1D EXSY studies of the polymer (VP) and mono nitrated porphyrin cage compound (**$H_2$2**) at 60 °C following first order kinetics. Left: the decay of orientation 1 as it converts into orientation 2 after the xylylene sidewall proton was selected for 1D EXSY measurements. This requires full dissociation of the guest from the cavity of the porphyrin cage compound. Right: the decay of the bound guest species as the bipyridine leaves the cavity, this encompasses both the slippage of the viologen in and out the cavity as well as full dissociation.

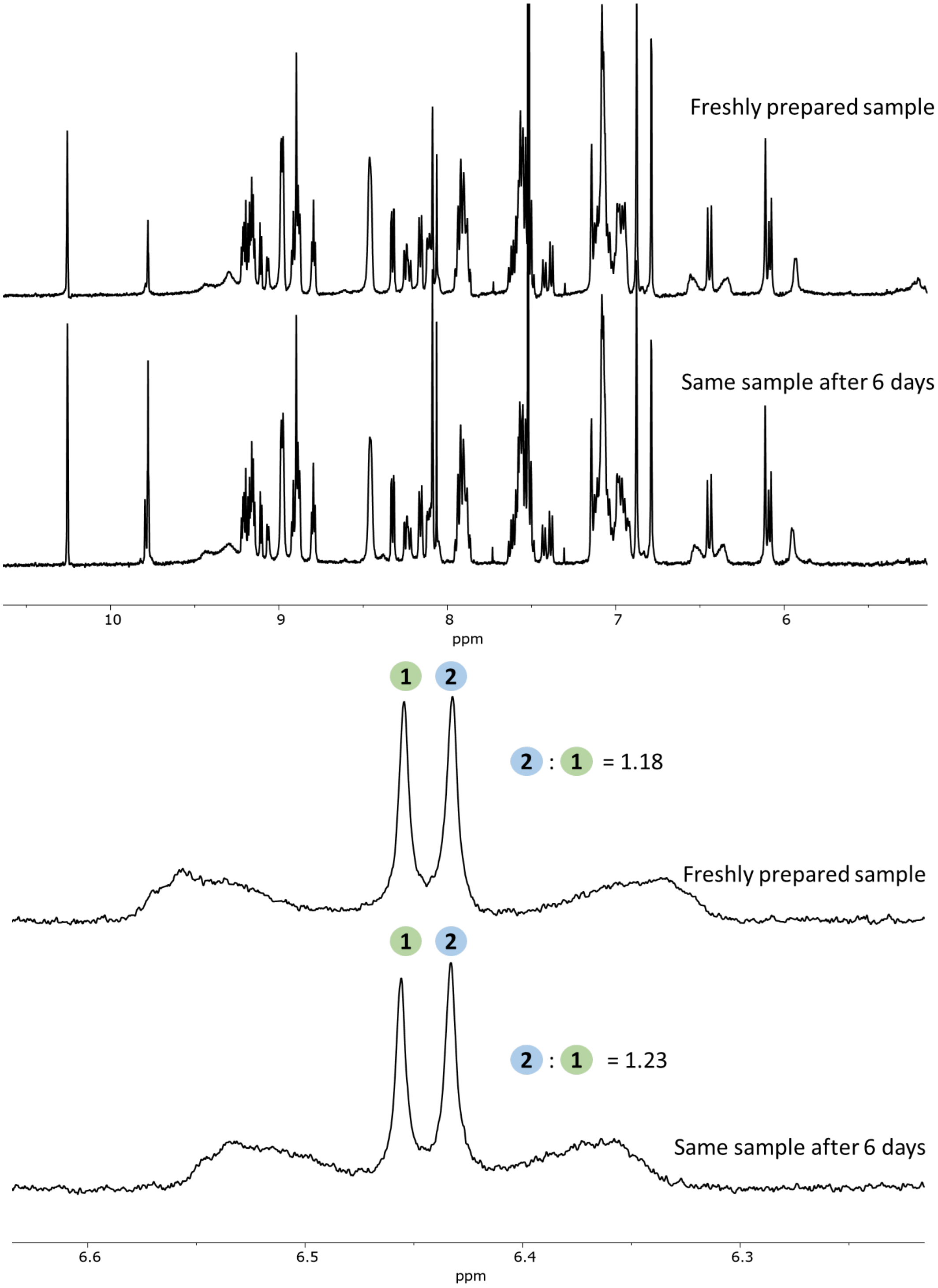

Figure SI29. Stacked ^{1}H-NMR spectra of **$H_2$2/VP** (500 MHz, 1:3, 2 mM : 6 mM, $CDCl_3$:CD_3CN, 1:1, v/v) taken 6 days apart. Bottom shows a zoomed in region of the spectrum with the xylylene-sidewall protons present. The ratio between the two does not differ significantly over the course of 6 days. This confirms that the polymer was already fully threaded upon starting the 1D EXSY measurements and that these measurements could be accurately performed within this timespan without any significant changes to the system.

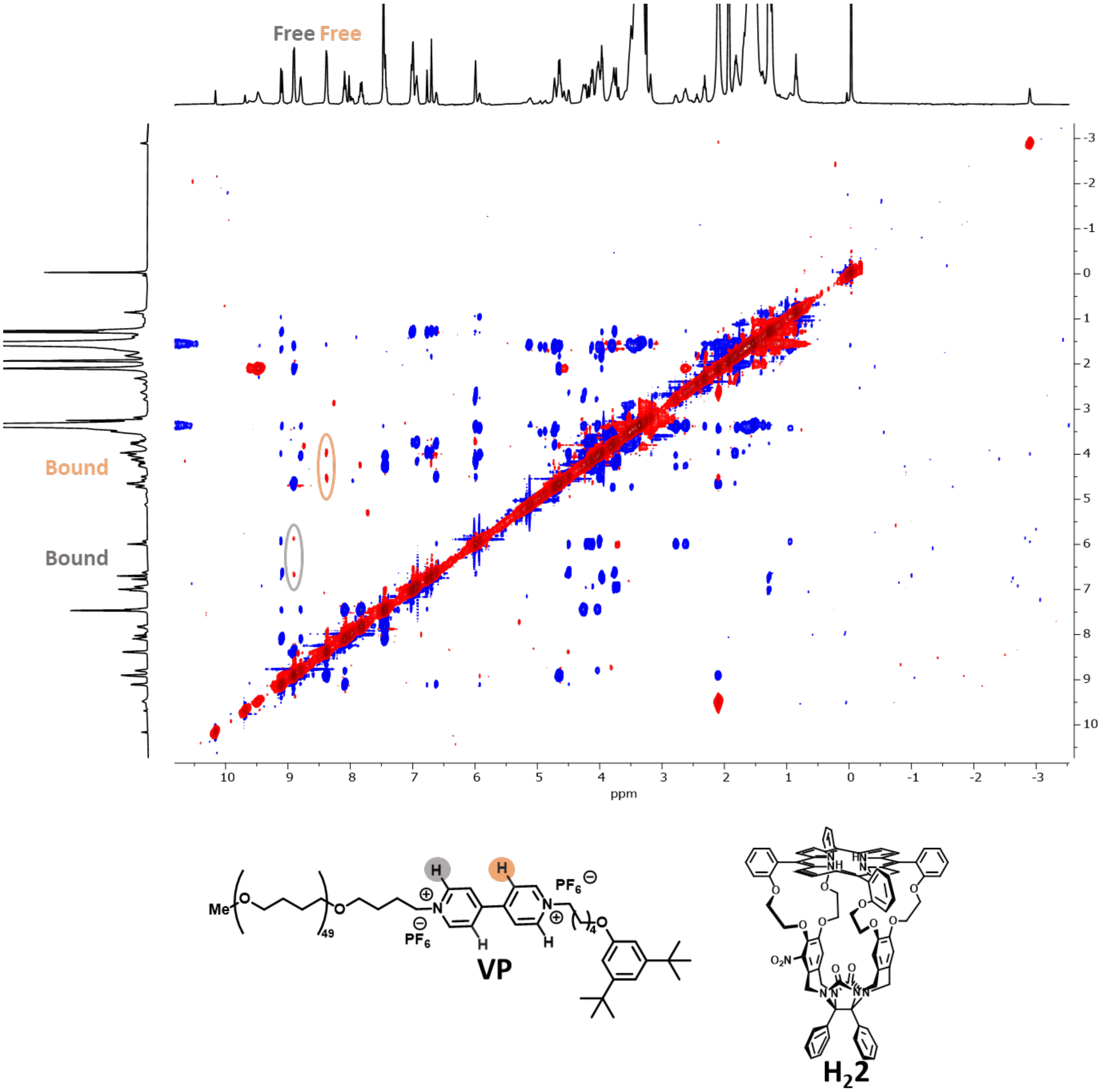

Figure SI30. Full ^{1}H-^{1}H 2D ROESY spectrum of **$H_2$2/VP** (^{1}H: 500 MHz, $CDCl_3$:CD_3CN, 1:1, v/v, host : guest, 1:3, 2 mM : 6 mM, T = 298 K)

Integration data of the exchange for $H_2$2/V1

The integration data of one system (**$H_2$2/V1**) is shown. The integration data for the other complexes can be obtained upon request by e-mail from the corresponding authors. At 40 °C the line fitting tool was used to obtain integrals for the EXSY data due to the signals not having a smooth line shape. For temperatures 50 to 70 °C the conventional integration method was used.

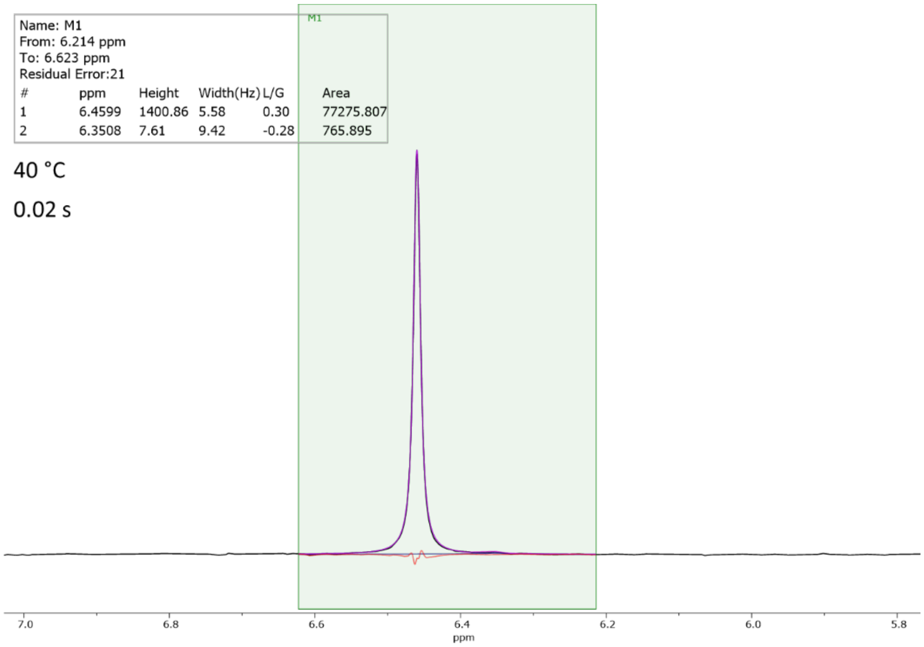

Figure SI31. 1D NOESY experiment of **$H_2$2/V1** (500 MHz, 1:3, 2 mM: 6 mM, $CDCl_3$:CD_3CN, 1:1, v/v) at 40 °C and a mix time of 0.02 s. The integrals are indicated under ‘’Area’’ and were obtained after applying line fitting in MestReNova version 14.1.0.

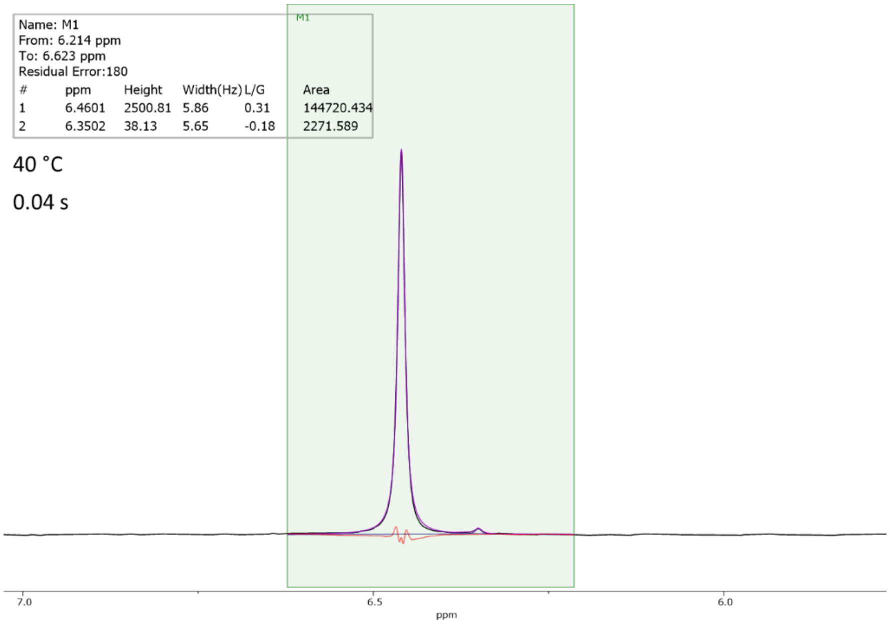

Figure SI32. 1D NOESY experiment of **H$_2$2/V1** (500 MHz, 1:3, 2 mM: 6 mM, $CDCl_3$:CD_3CN, 1:1, v/v) at 40 °C and a mix time of 0.04 s. The integrals are indicated under ''Area'' and were obtained after applying line fitting in MestReNova version 14.1.0.

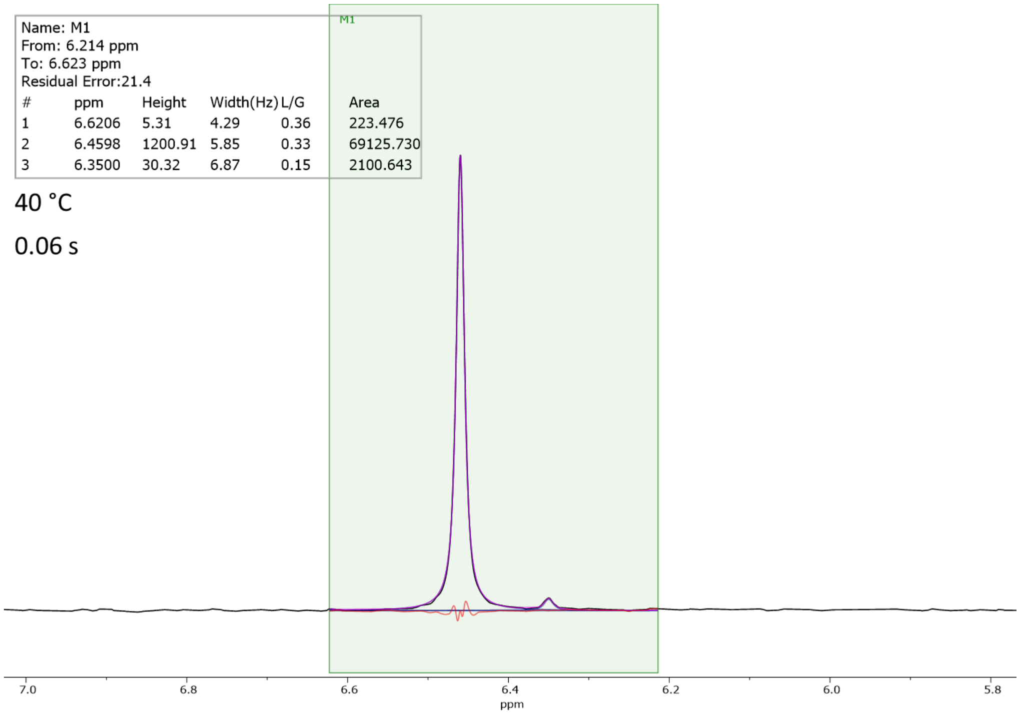

Figure SI33. 1D NOESY experiment of **H$_2$2/V1** (500 MHz, 1:3, 2 mM: 6 mM, $CDCl_3$:CD_3CN, 1:1, v/v) at 40 °C and a mix time of 0.06 s. The integrals are indicated under ''Area'' and were obtained after applying line fitting in MestReNova version 14.1.0.

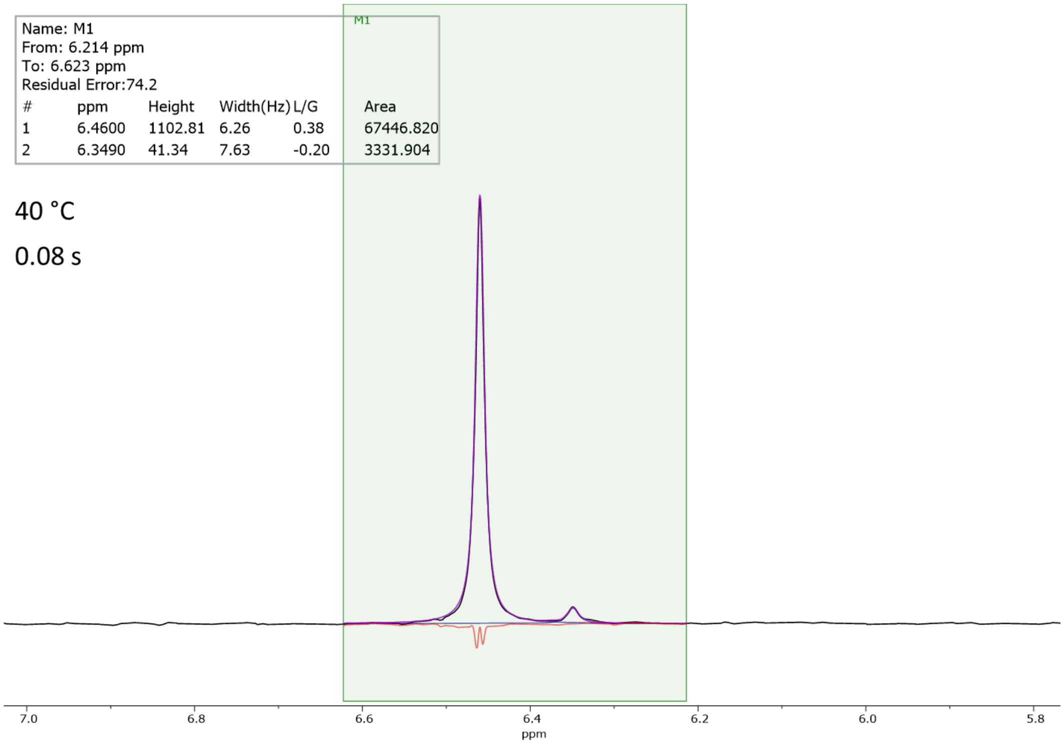

Figure SI34. 1D NOESY experiment of **$H_2$2/V1** (500 MHz, 1:3, 2 mM: 6 mM, $CDCl_3$:CD_3CN, 1:1, v/v) at 40 °C and a mix time of 0.08 s. The integrals are indicated under ''Area'' and were obtained after applying line fitting in MestReNova version 14.1.0.

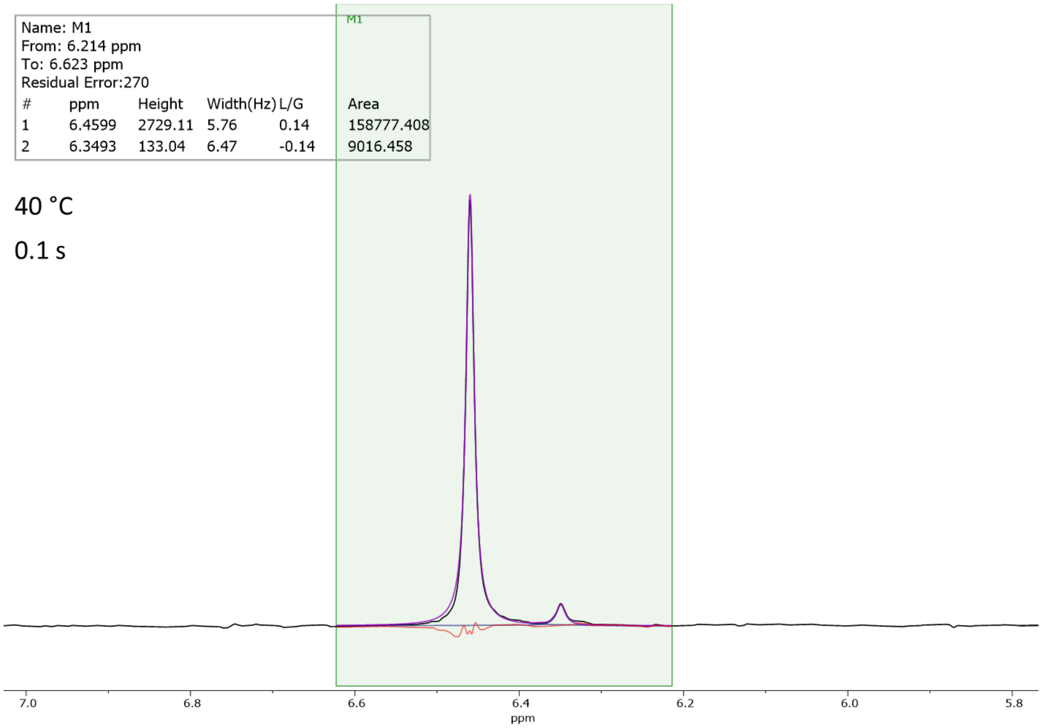

Figure SI35. 1D NOESY experiment of **$H_2$2/V1** (500 MHz, 1:3, 2 mM: 6 mM, $CDCl_3$:CD_3CN, 1:1, v/v) at 40 °C and a mix time of 0.1 s. The integrals are indicated under ''Area'' and were obtained after applying line fitting in MestReNova version 14.1.0.

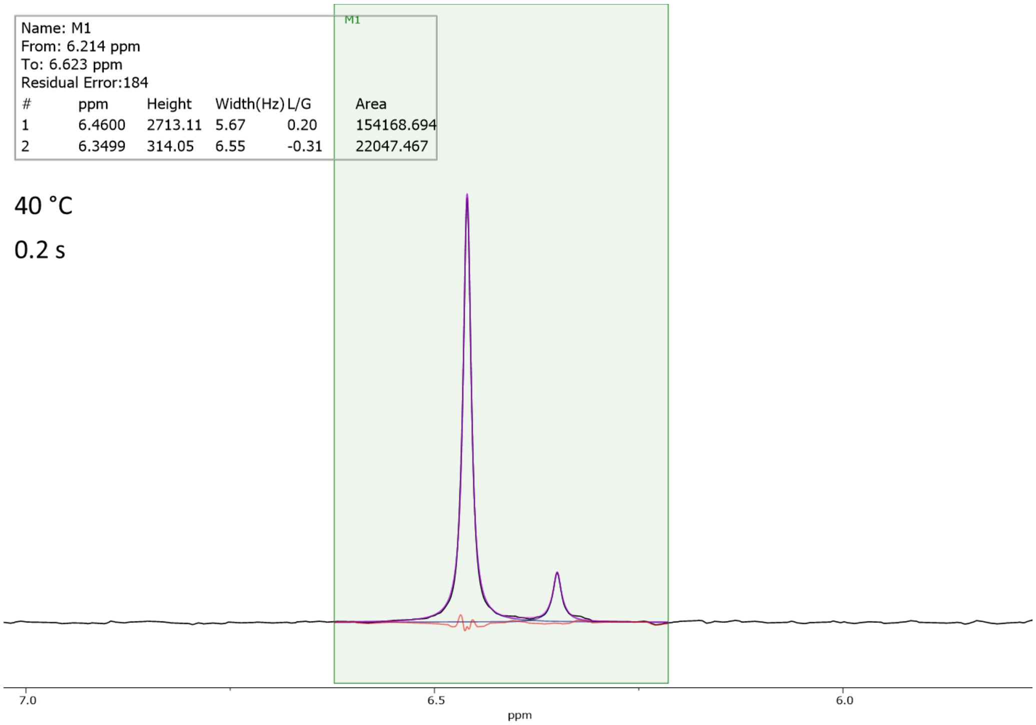

Figure SI36. 1D NOESY experiment of **$H_2$2/V1** (500 MHz, 1:3, 2 mM: 6 mM, $CDCl_3$:CD_3CN, 1:1, v/v) at 40 °C and a mix time of 0.2 s. The integrals are indicated under ''Area'' and were obtained after applying line fitting in MestReNova version 14.1.0.

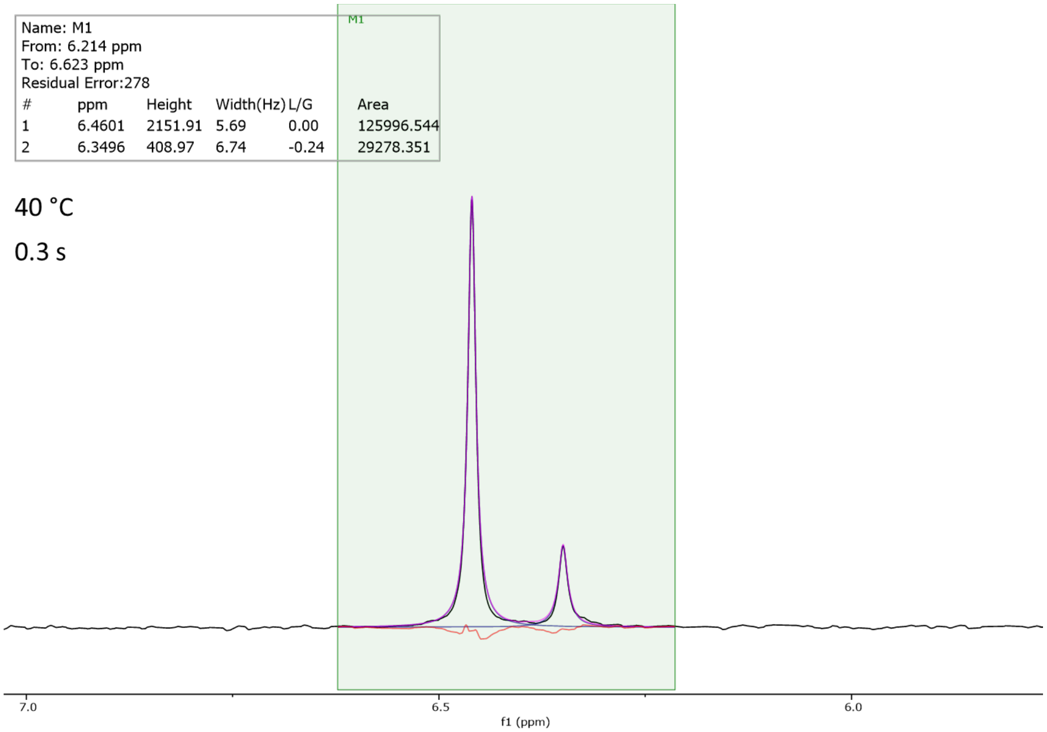

Figure SI37. 1D NOESY experiment of **$H_2$2/V1** (500 MHz, 1:3, 2 mM: 6 mM, $CDCl_3$:CD_3CN, 1:1, v/v) at 40 °C and a mix time of 0.3 s. The integrals are indicated under ''Area'' and were obtained after applying line fitting in MestReNova version 14.1.0.

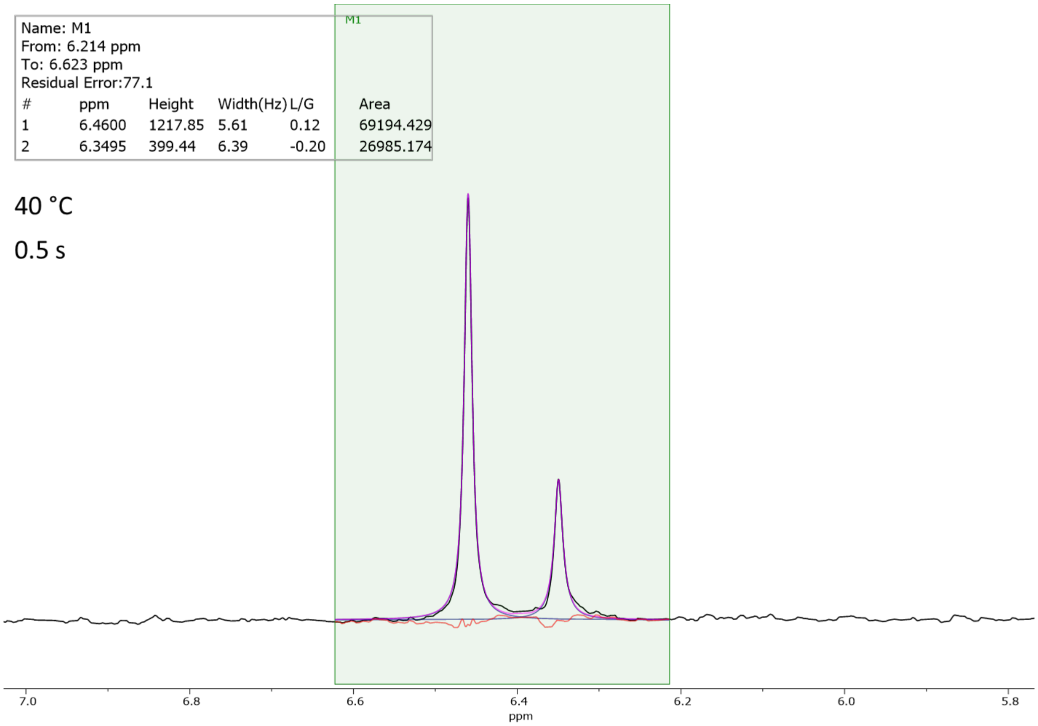

Figure SI38. 1D NOESY experiment of **$H_2$2/V1** (500 MHz, 1:3, 2 mM: 6 mM, $CDCl_3$:CD_3CN, 1:1, v/v) at 40 °C and a mix time of 0.5 s. The integrals are indicated under ''Area'' and were obtained after applying line fitting in MestReNova version 14.1.0.

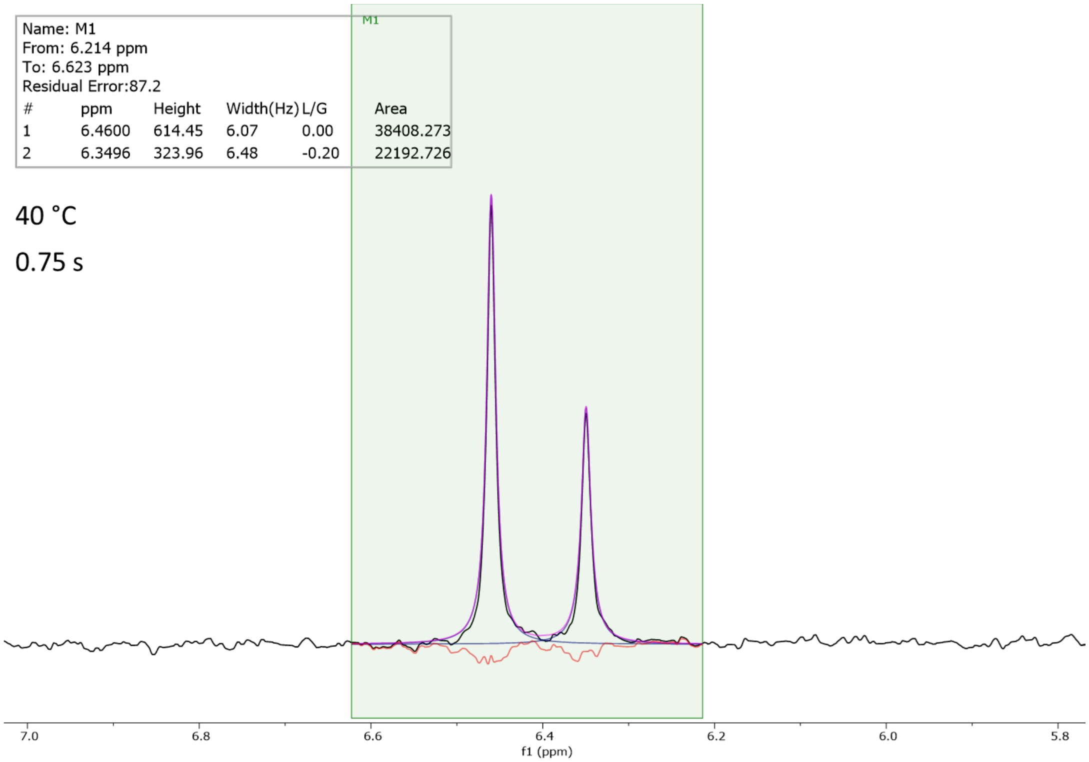

Figure SI39. 1D NOESY experiment of **$H_2$2/V1** (500 MHz, 1:3, 2 mM: 6 mM, $CDCl_3$:CD_3CN, 1:1, v/v) at 40 °C and a mix time of 0.75 s. The integrals are indicated under ''Area'' and were obtained after applying line fitting in MestReNova version 14.1.0.

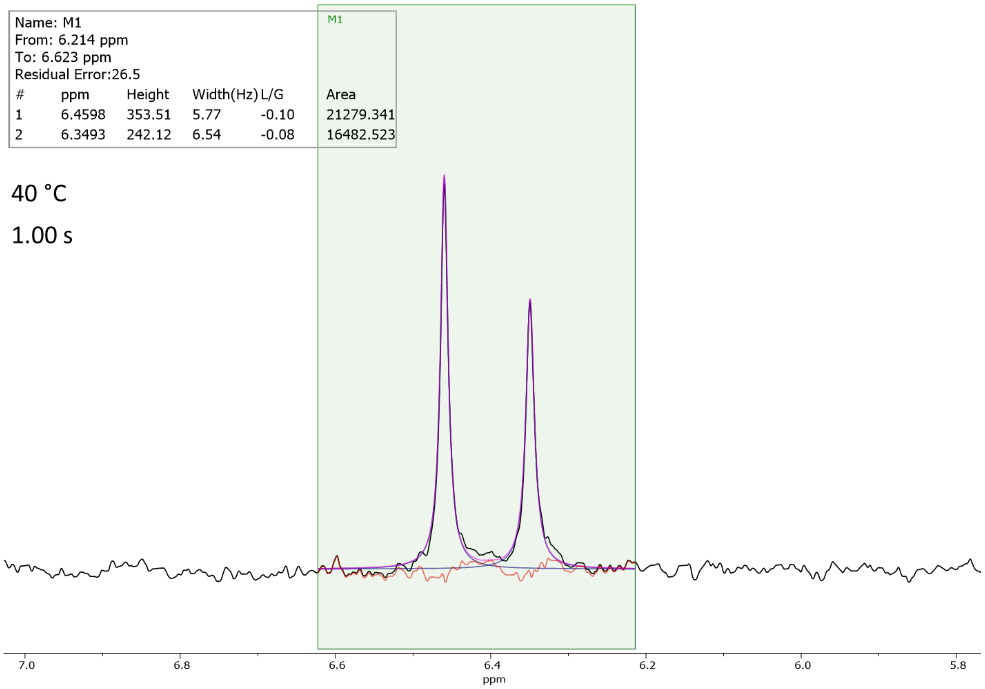

Figure SI40. 1D NOESY experiment of **$H_2$2/V1** (500 MHz, 1:3, 2 mM: 6 mM, $CDCl_3$:CD_3CN, 1:1, v/v) at 40 °C and a mix time of 1.00 s. The integrals are indicated under ''Area'' and were obtained after applying line fitting in MestReNova version 14.1.0.

Table SI6. Overview of 1D EXSY data for **$H_2$2/V1** at 40 °C. The integrals are shown as well as the decrease in concentration of the (irradiated) minor abundant species and the increase of the major abundant species.

40 °C		Integrals		Percentages		Concentrations	
No. scans	Time (s)	Minor	Major	Minor%	Major%	[Minor] (mM)	[Major] (mM)
512	0.02	77275.81	765.895	99.01861	0.981392	0.732936	0.007264
1024	0.04	144720.4	2271.589	98.45462	1.545383	0.728761	0.011439
512	0.06	69125.73	2100.643	97.05075	2.949249	0.71837	0.02183
512	0.08	67446.82	3331.904	95.29251	4.707494	0.705355	0.034845
512	0.1	158777.4	9016.458	94.62647	5.373533	0.700425	0.039775
512	0.2	154168.7	22047.47	87.4884	12.5116	0.647589	0.092611
512	0.3	125996.5	29278.35	81.14418	18.85582	0.600629	0.139571
512	0.5	69194.43	26985.17	71.94293	28.05707	0.532522	0.207678
512	0.75	38408.27	22192.73	63.37894	36.62106	0.469131	0.271069
512	1	21279.34	16582.52	56.20257	43.79743	0.416011	0.324189

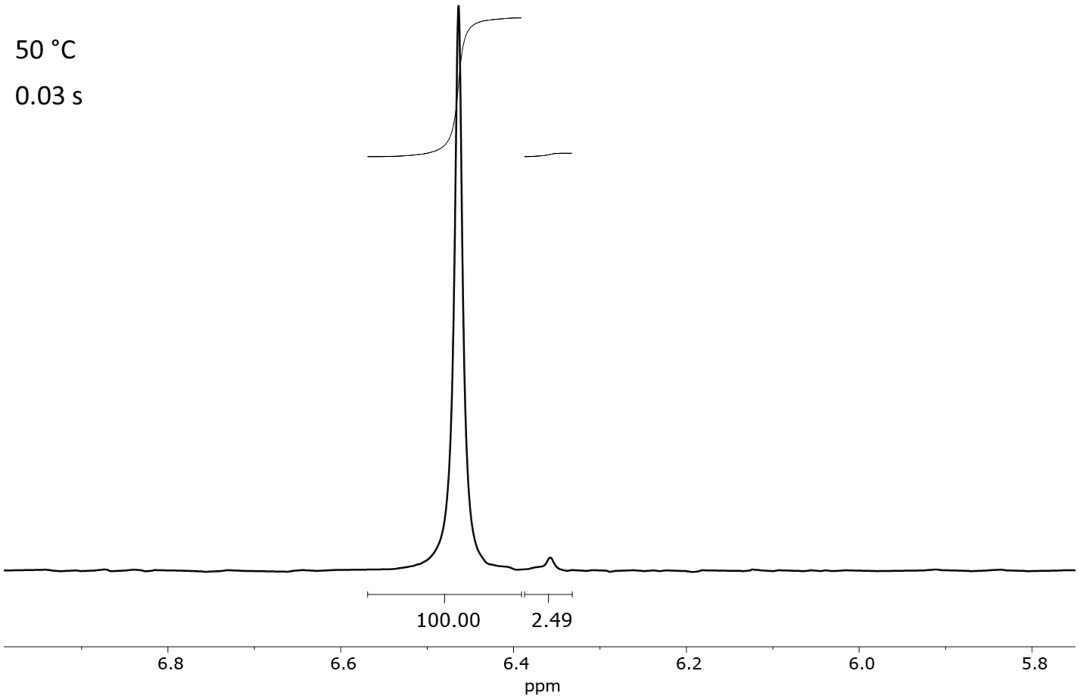

Figure SI41. 1D NOESY experiment of **$H_2$2/V1** (500 MHz, 1:3, 2 mM: 6 mM, $CDCl_3$:CD_3CN, 1:1, v/v) at 50 °C and a mix time of 0.03 s.

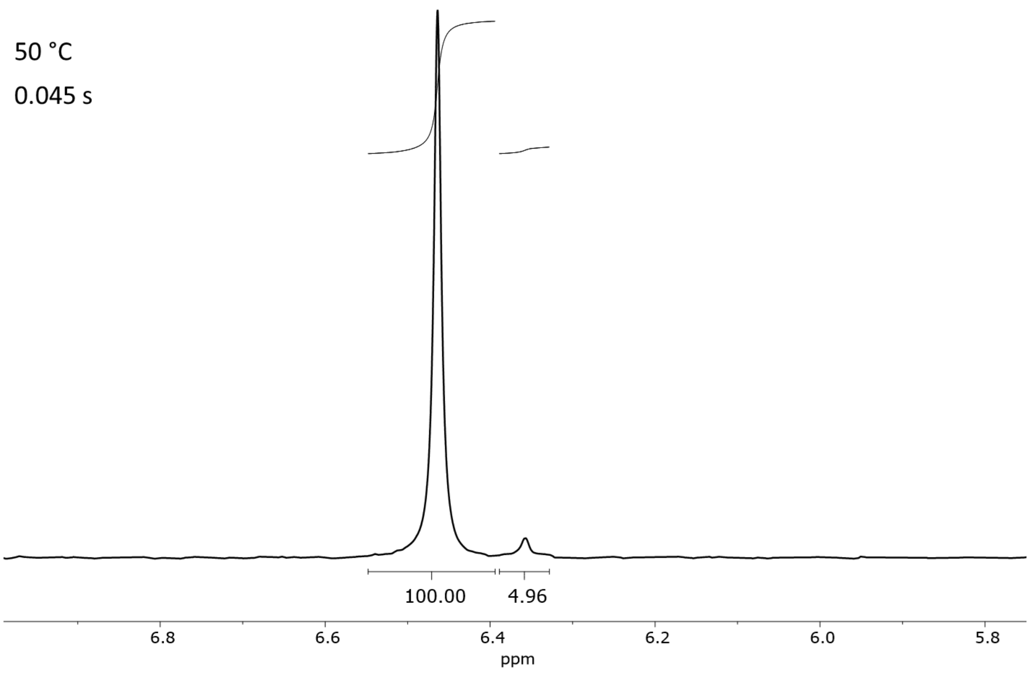

Figure SI42. 1D NOESY experiment of **$H_2$2/V1** (500 MHz, 1:3, 2 mM: 6 mM, $CDCl_3$:CD_3CN, 1:1, v/v) at 50 °C and a mix time of 0.045 s.

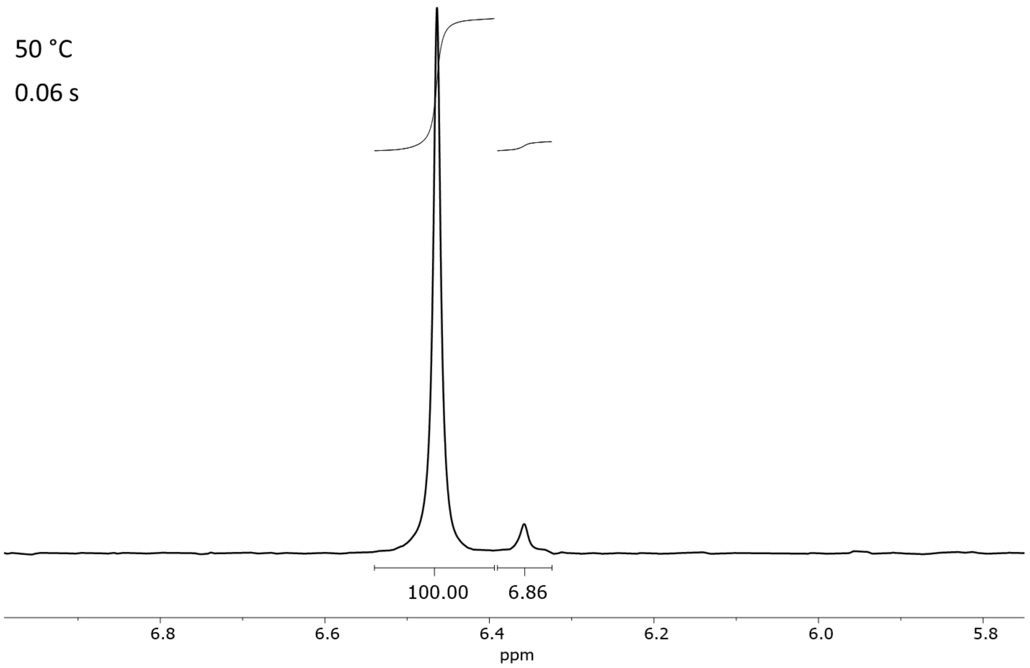

Figure SI43. 1D NOESY experiment of **$H_2$2/V1** (500 MHz, 1:3, 2 mM: 6 mM, $CDCl_3$:CD_3CN, 1:1, v/v) at 50 °C and a mix time of 0.06 s.

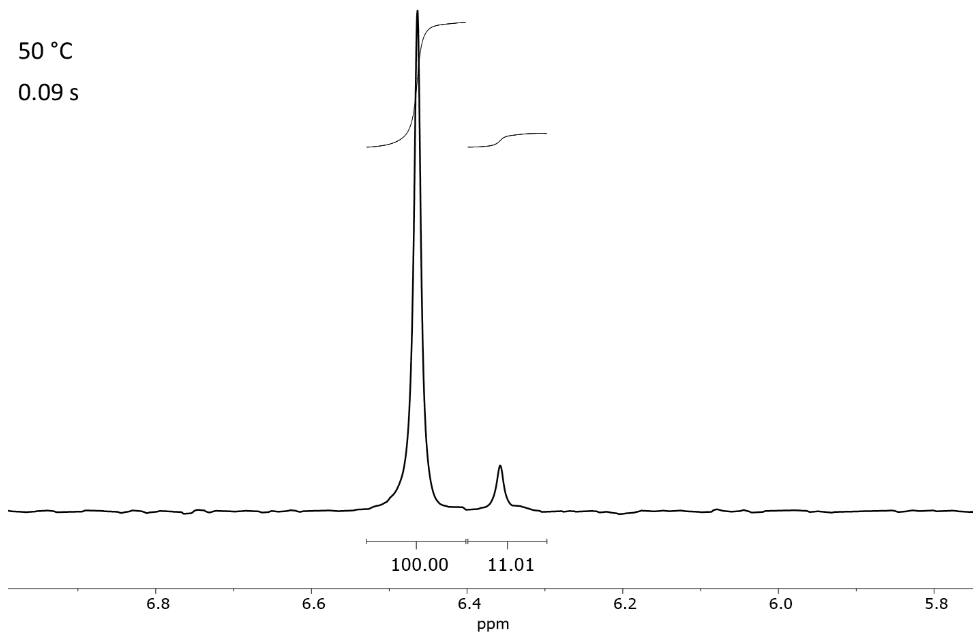

Figure SI44. 1D NOESY experiment of **$H_2$2/V1** (500 MHz, 1:3, 2 mM: 6 mM, $CDCl_3$:CD_3CN, 1:1, v/v) at 50 °C and a mix time of 0.09 s.

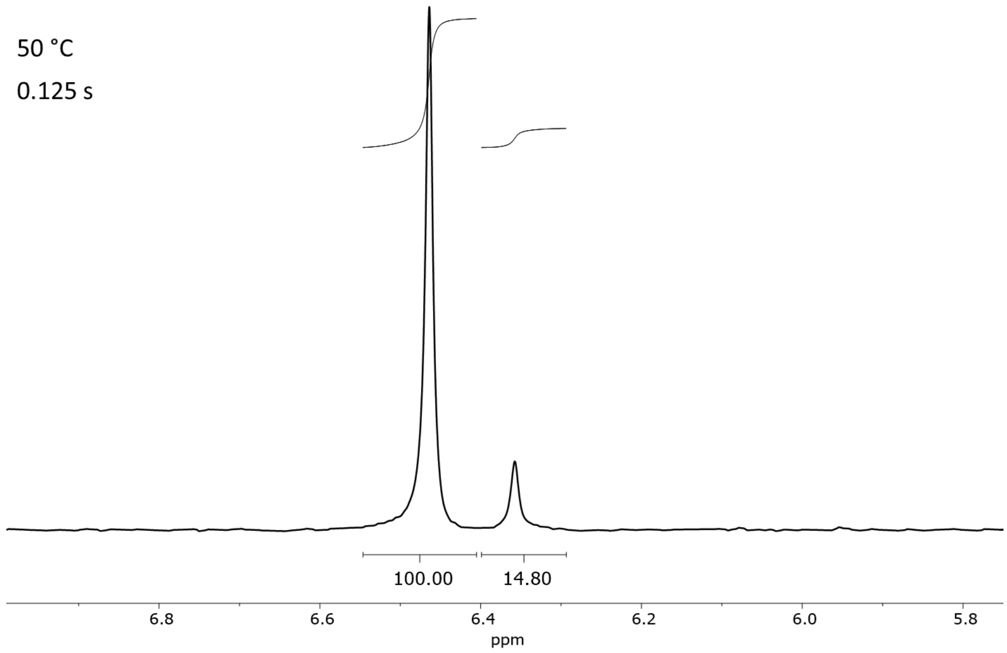

Figure SI45. 1D NOESY experiment of **$H_2$2/V1** (500 MHz, 1:3, 2 mM: 6 mM, $CDCl_3$:CD_3CN, 1:1, v/v) at 50 °C and a mix time of 0.125 s.

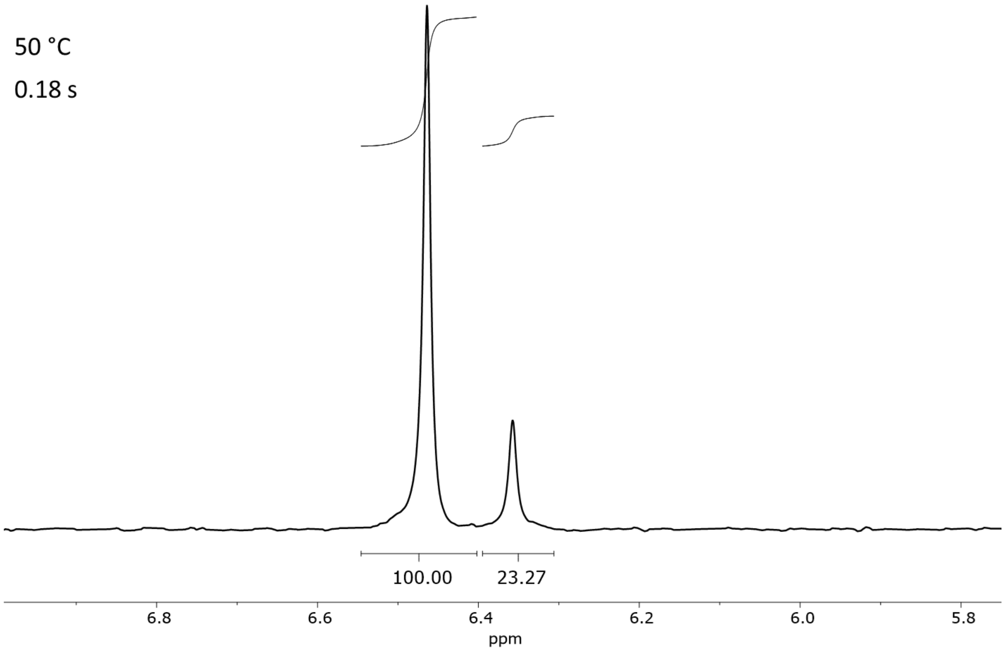

Figure SI46. 1D NOESY experiment of **$H_2$2/V1** (500 MHz, 1:3, 2 mM: 6 mM, $CDCl_3$:CD_3CN, 1:1, v/v) at 50 °C and a mix time of 0.18 s.

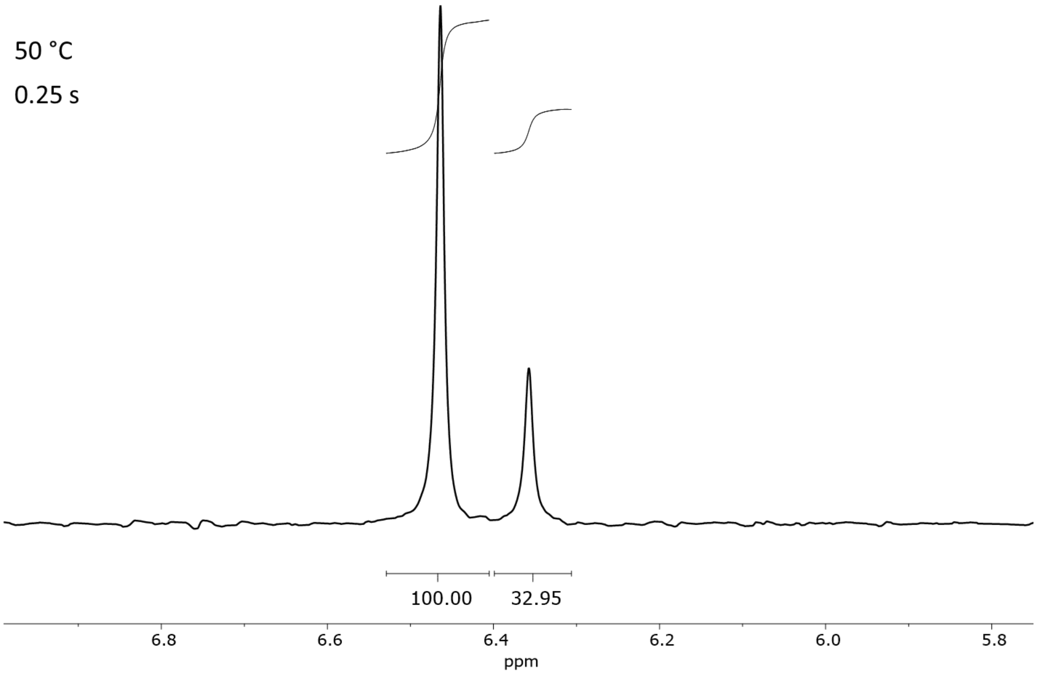

Figure SI47. 1D NOESY experiment of **$H_2$2/V1** (500 MHz, 1:3, 2 mM: 6 mM, $CDCl_3$:CD_3CN, 1:1, v/v) at 50 °C and a mix time of 0.25 s.

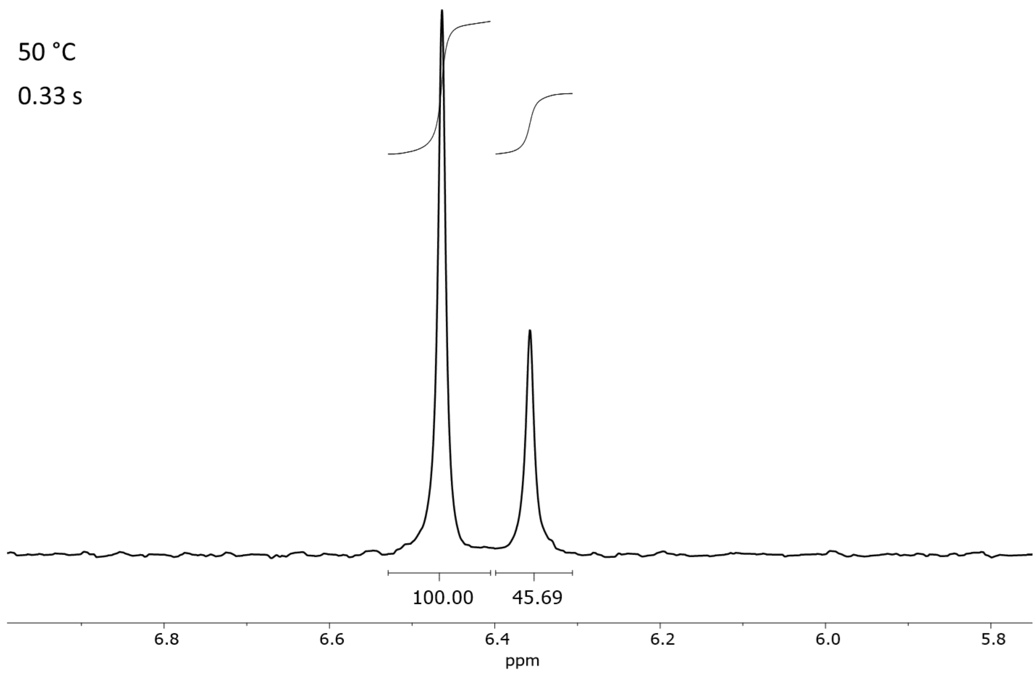

Figure SI48. 1D NOESY experiment of **$H_2$2/V1** (500 MHz, 1:3, 2 mM: 6 mM, $CDCl_3$:CD_3CN, 1:1, v/v) at 50 °C and a mix time of 0.33 s.

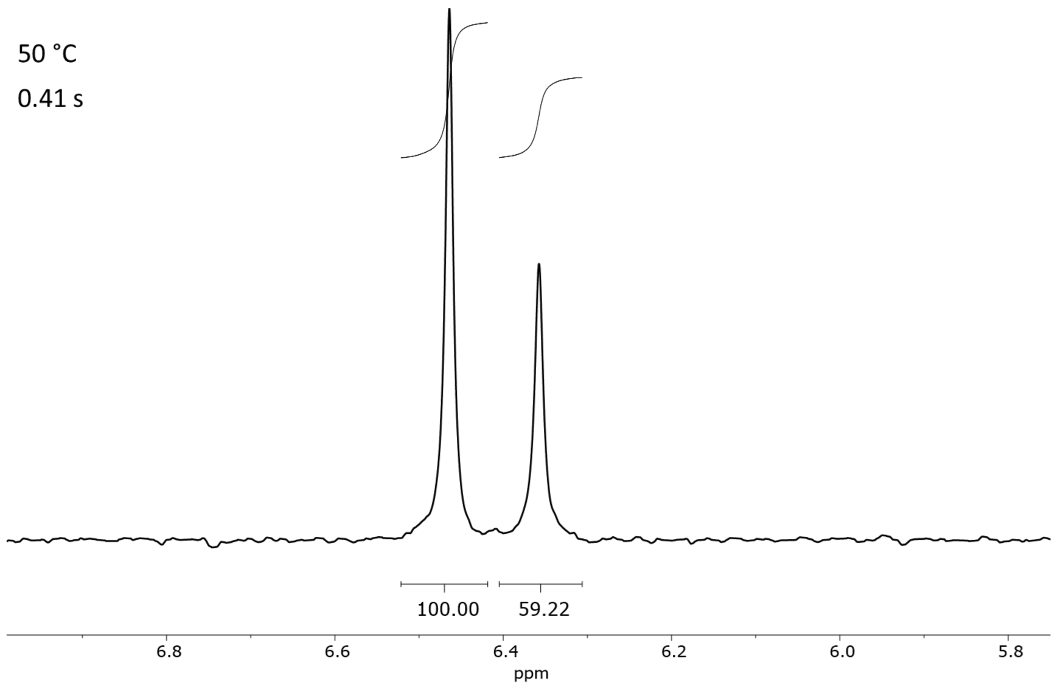

Figure SI49. 1D NOESY experiment of **$H_2$2/V1** (500 MHz, 1:3, 2 mM: 6 mM, $CDCl_3$:CD_3CN, 1:1, v/v) at 50 °C and a mix time of 0.41 s.

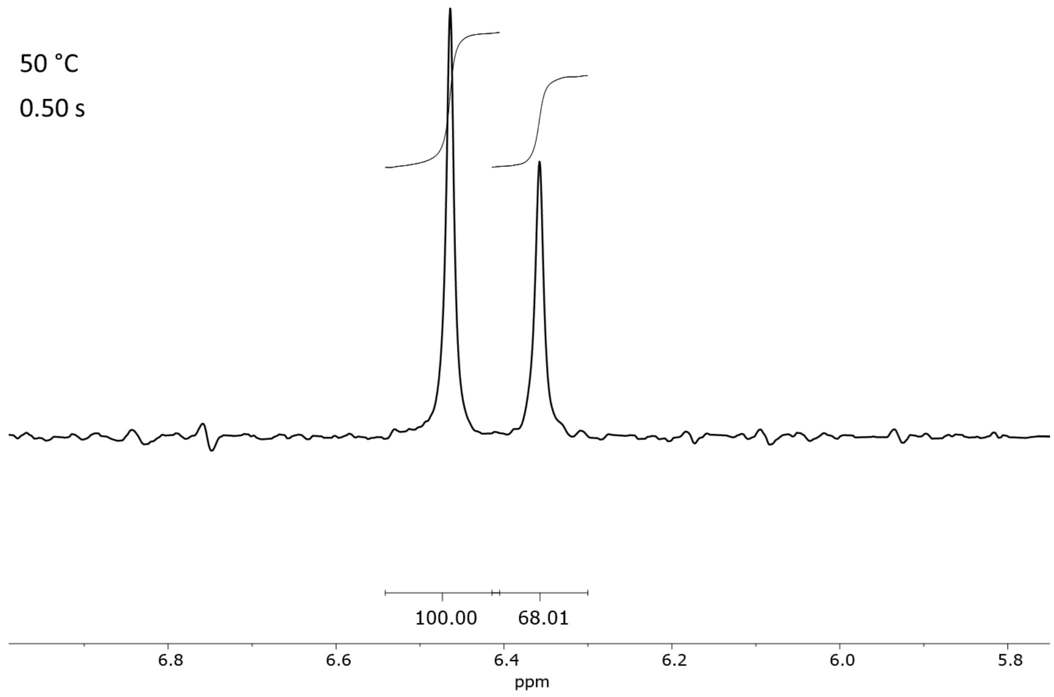

Figure SI50. 1D NOESY experiment of **$H_2$2/V1** (500 MHz, 1:3, 2 mM: 6 mM, $CDCl_3$:CD_3CN, 1:1, v/v) at 50 °C and a mix time of 0.50 s.

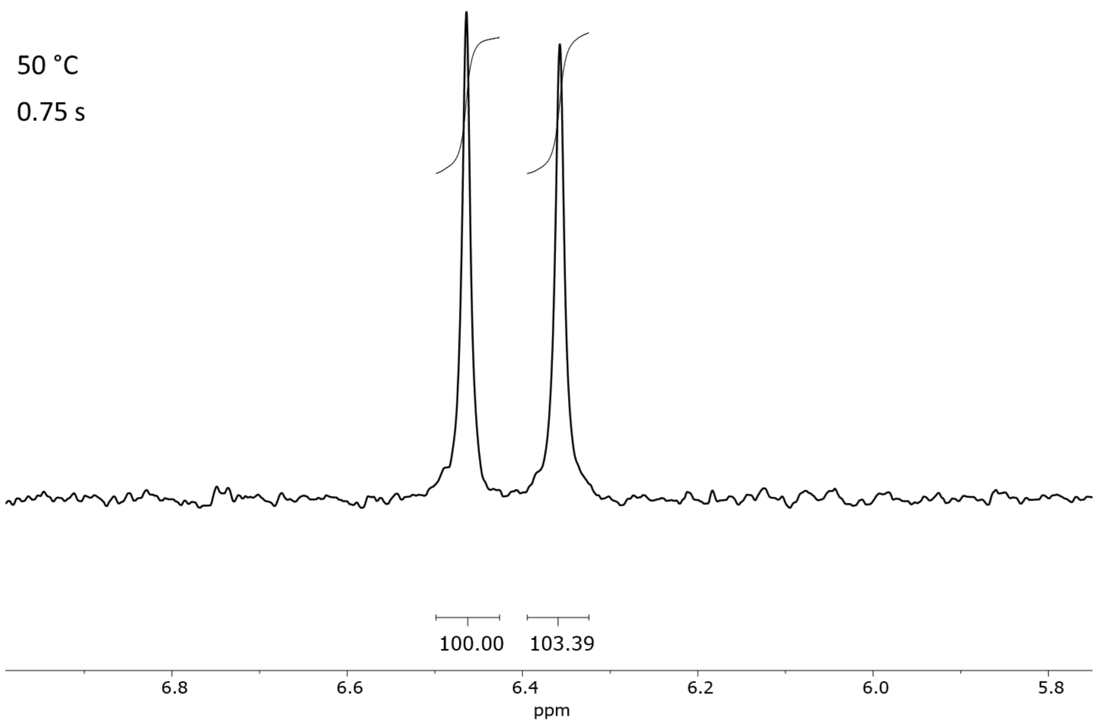

Figure SI51. 1D NOESY experiment of **$H_2$2/V1** (500 MHz, 1:3, 2 mM: 6 mM, $CDCl_3$:CD_3CN, 1:1, v/v) at 50 °C and a mix time of 0.75 s.

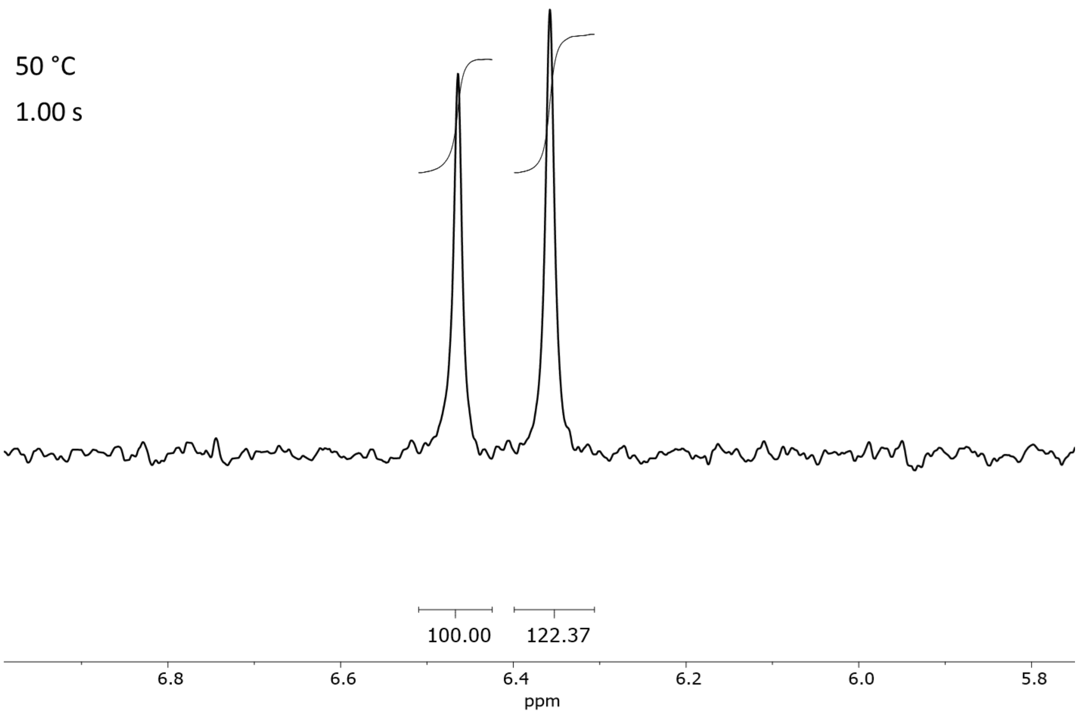

Figure SI52. 1D NOESY experiment of **$H_2$2/V1** (500 MHz, 1:3, 2 mM: 6 mM, $CDCl_3$:CD_3CN, 1:1, v/v) at 50 °C and a mix time of 1.00 s.

Table SI7. Overview of 1D EXSY data for **$H_2$2/V1** at 50 °C. The integrals are shown as well as the decrease in concentration of the (irradiated) minor abundant species and the increase of the major abundant species.

50 °C		Integrals		Percentages		Concentrations	
No. scans	Time (s)	Minor	Major	Minor%	Major%	[Minor] (mM)	[Major] (mM)
512	0.03	100	2.49	97.570495	2.429505	0.7222168	0.0179832
512	0.045	100	4.96	95.27439	4.72561	0.705221	0.034979
512	0.06	100	6.86	93.580386	6.419614	0.692682	0.047518
512	0.09	100	11.01	90.081975	9.918025	0.6667868	0.0734132
512	0.125	100	14.8	87.108014	12.89199	0.6447735	0.0954265
512	0.18	100	23.27	81.122739	18.87726	0.6004705	0.1397295
512	0.25	100	32.95	75.216247	24.78375	0.5567507	0.1834493
512	0.33	100	45.69	68.638891	31.36111	0.5080651	0.2321349
512	0.41	100	59.22	62.80618	37.19382	0.4648913	0.2753087
512	0.5	100	68.17	59.463638	40.53636	0.4401498	0.3000502
512	0.75	100	103.65	49.103855	50.89615	0.3634667	0.3767333
512	1	100	122.37	44.970095	55.02991	0.3328686	0.4073314

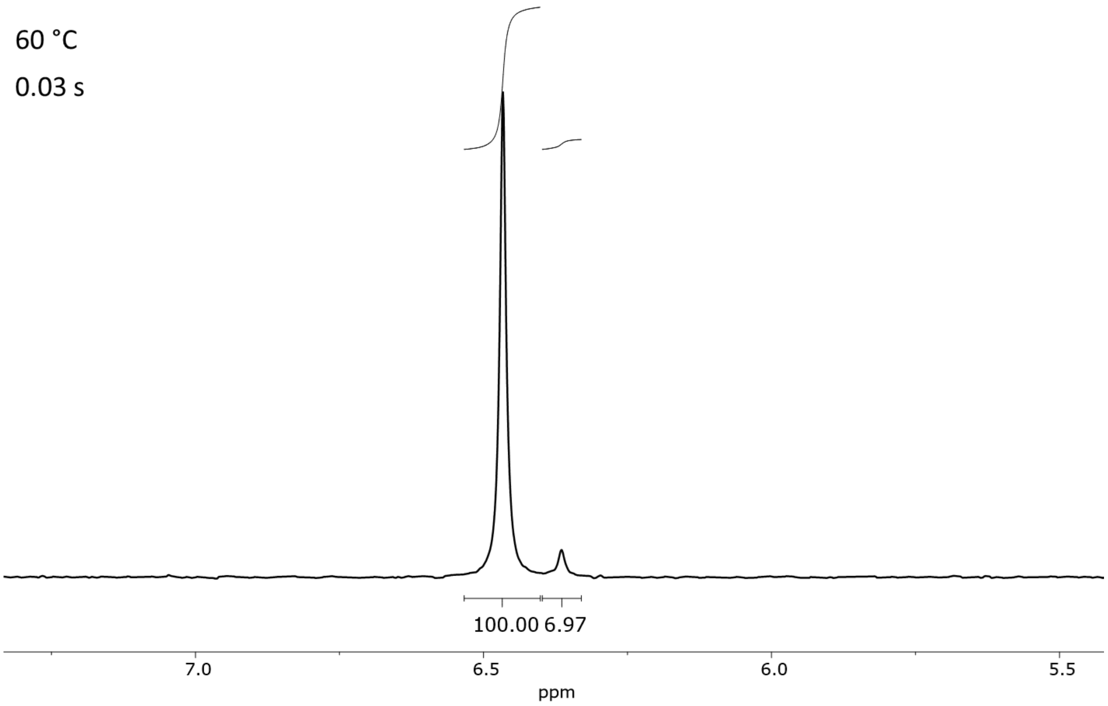

Figure SI53. 1D NOESY experiment of **$H_2$2/V1** (500 MHz, 1:3, 2 mM: 6 mM, $CDCl_3$:CD_3CN, 1:1, v/v) at 60 °C and a mix time of 0.03 s.

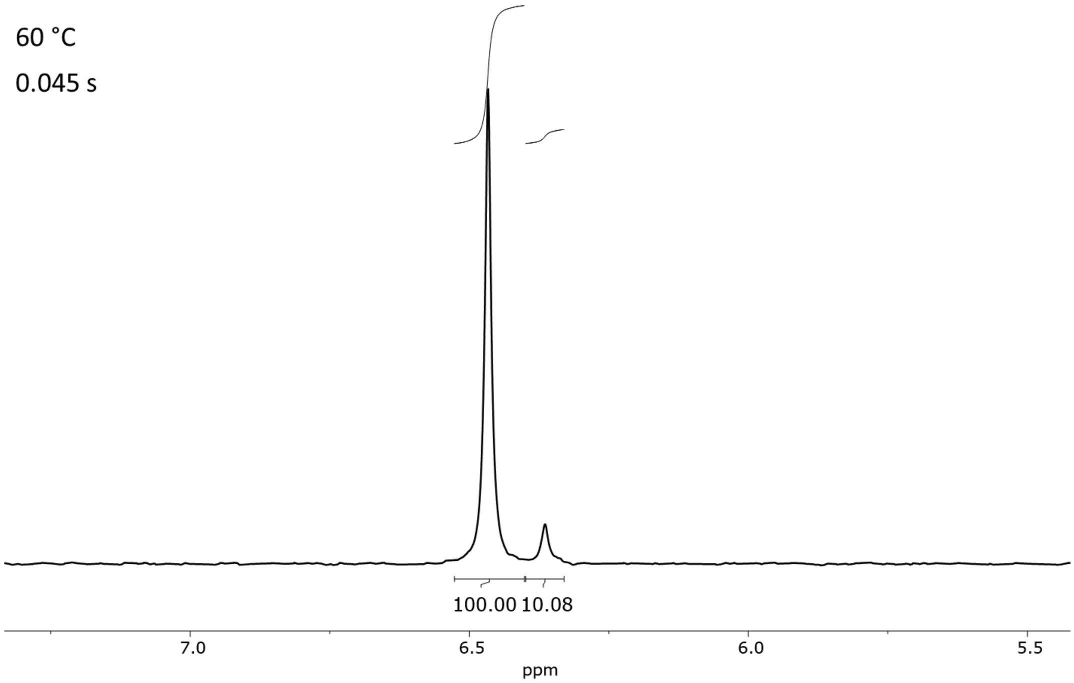

Figure SI54. 1D NOESY experiment of **$H_2$2/V1** (500 MHz, 1:3, 2 mM: 6 mM, $CDCl_3$:CD_3CN, 1:1, v/v) at 60 °C and a mix time of 0.045 s.

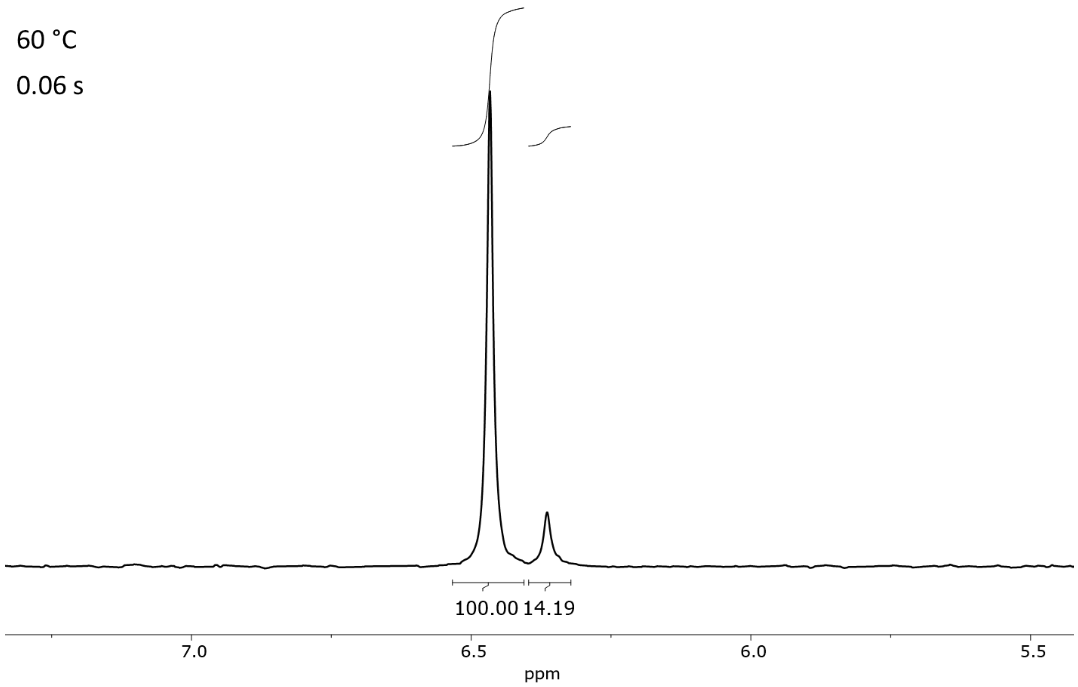

Figure SI55. 1D NOESY experiment of **$H_2$2/V1** (500 MHz, 1:3, 2 mM: 6 mM, $CDCl_3$:CD_3CN, 1:1, v/v) at 60 °C and a mix time of 0.06 s.

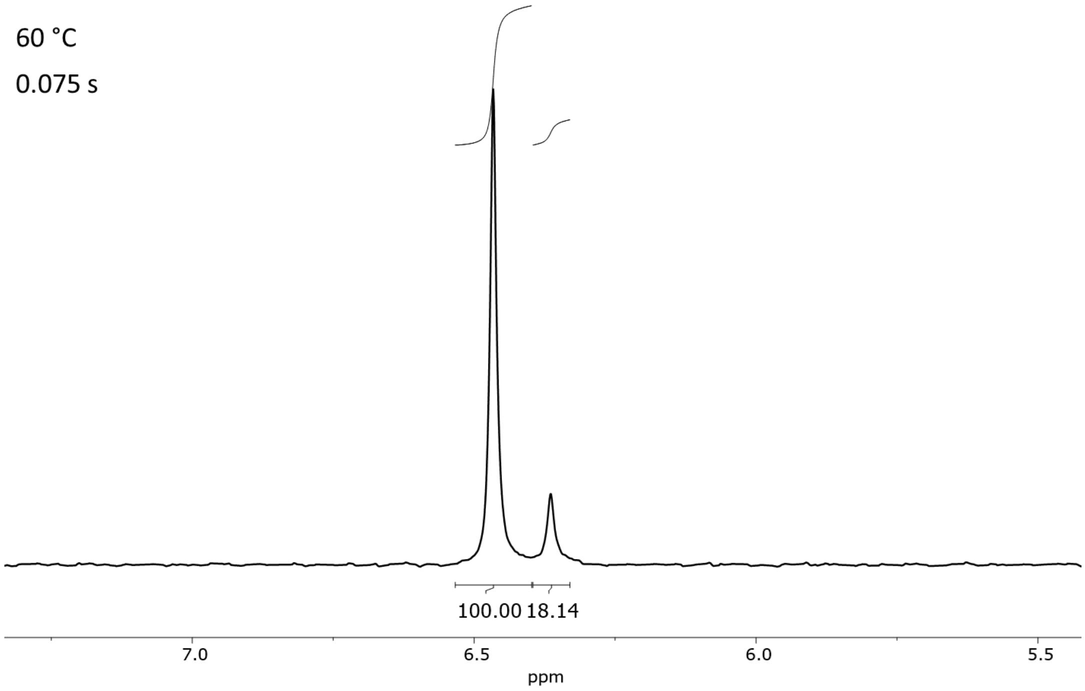

Figure SI56. 1D NOESY experiment of **$H_2$2/V1** (500 MHz, 1:3, 2 mM: 6 mM, $CDCl_3$:CD_3CN, 1:1, v/v) at 60 °C and a mix time of 0.075 s.

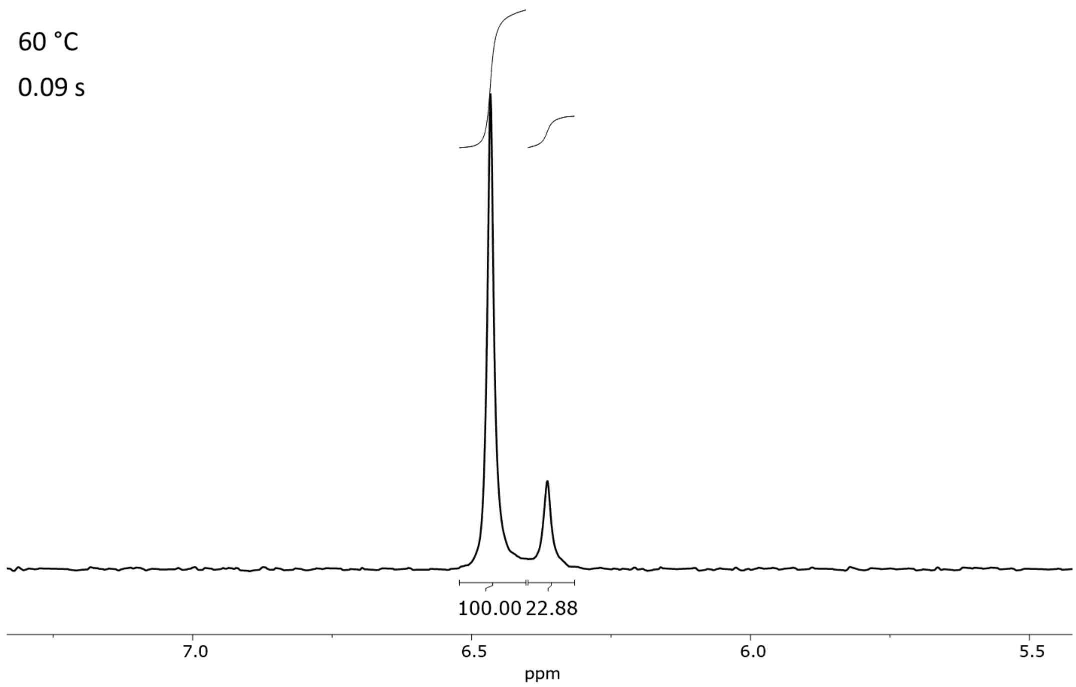

Figure SI57. 1D NOESY experiment of **$H_2$2/V1** (500 MHz, 1:3, 2 mM: 6 mM, $CDCl_3$:CD_3CN, 1:1, v/v) at 60 °C and a mix time of 0.09 s.

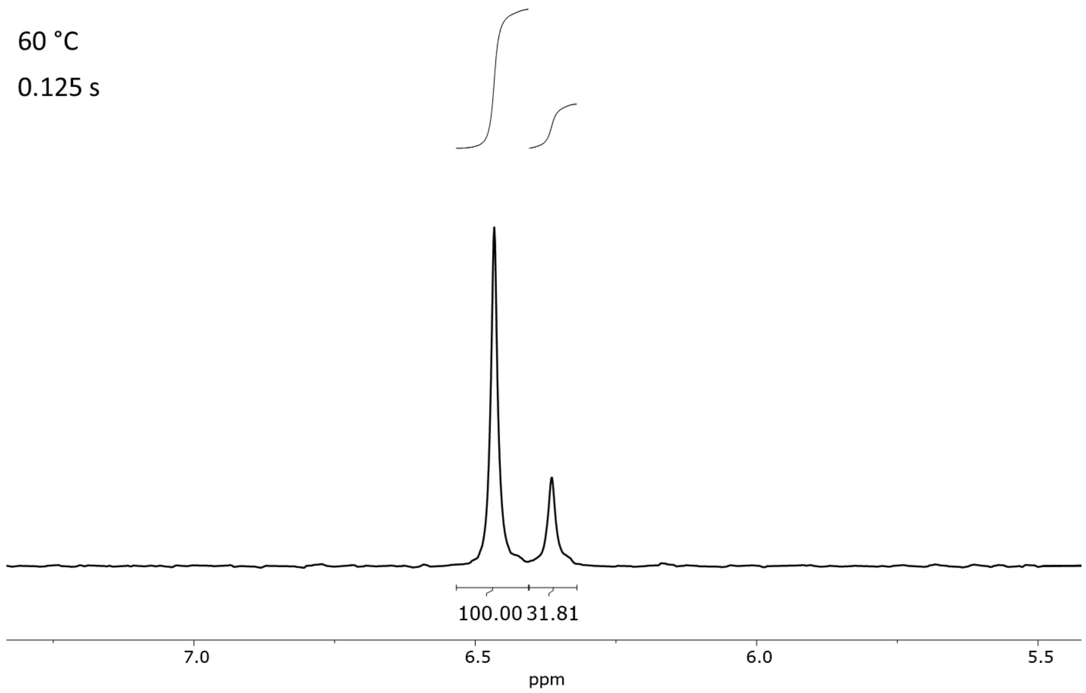

Figure SI58. 1D NOESY experiment of **$H_2$2/V1** (500 MHz, 1:3, 2 mM: 6 mM, $CDCl_3$:CD_3CN, 1:1, v/v) at 60 °C and a mix time of 0.125 s.

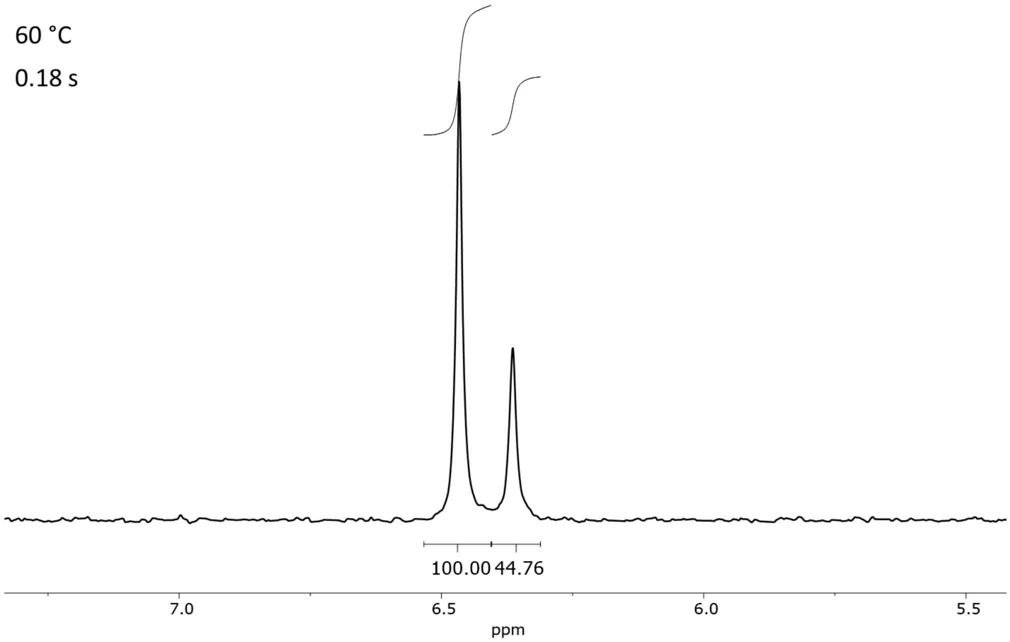

Figure SI59. 1D NOESY experiment of **$H_2$2/V1** (500 MHz, 1:3, 2 mM: 6 mM, $CDCl_3$:CD_3CN, 1:1, v/v) at 60 °C and a mix time of 0.18 s.

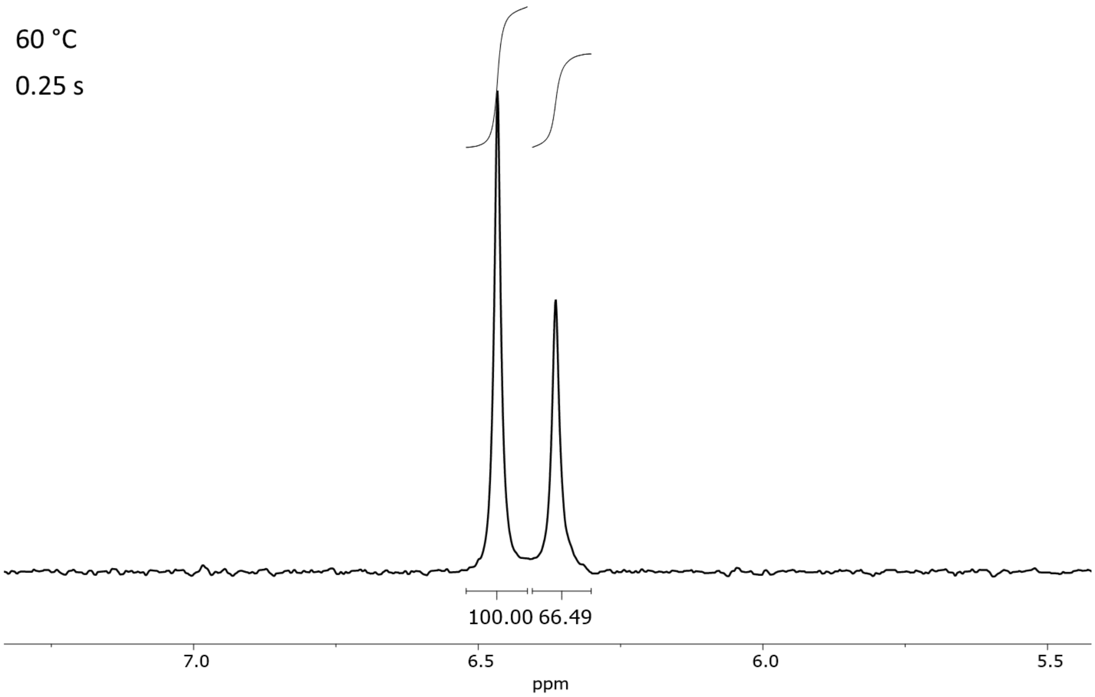

Figure SI60. 1D NOESY experiment of **$H_2$2/V1** (500 MHz, 1:3, 2 mM: 6 mM, $CDCl_3$:CD_3CN, 1:1, v/v) at 60 °C and a mix time of 0.25 s.

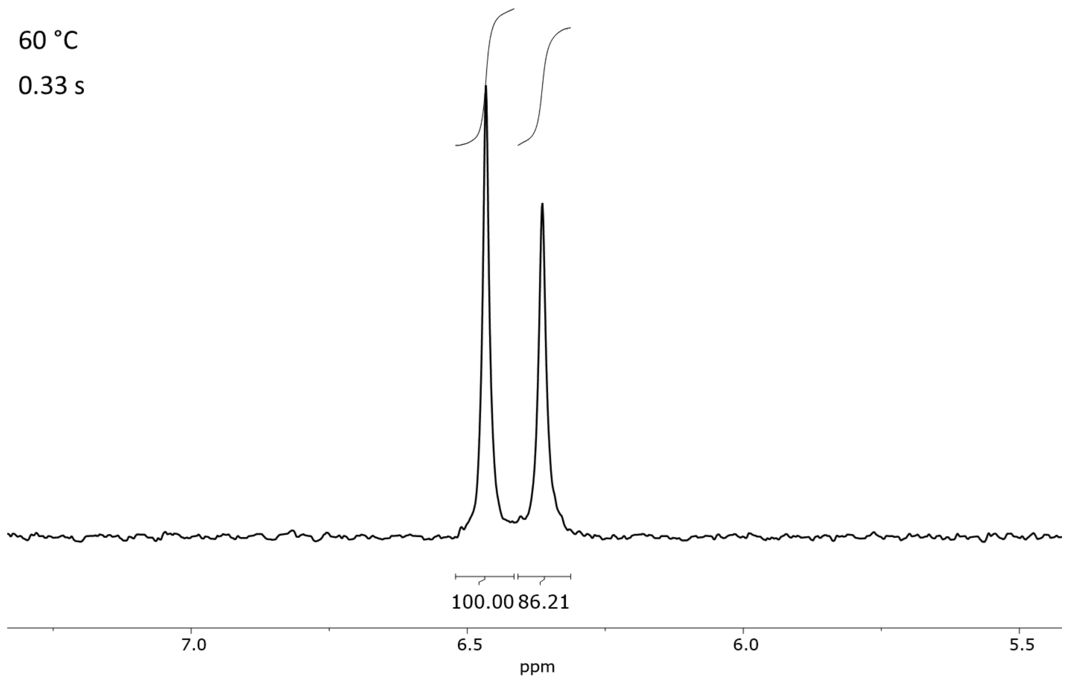

Figure SI61. 1D NOESY experiment of **$H_2$2/V1** (500 MHz, 1:3, 2 mM: 6 mM, $CDCl_3$:CD_3CN, 1:1, v/v) at 60 °C and a mix time of 0.33 s.

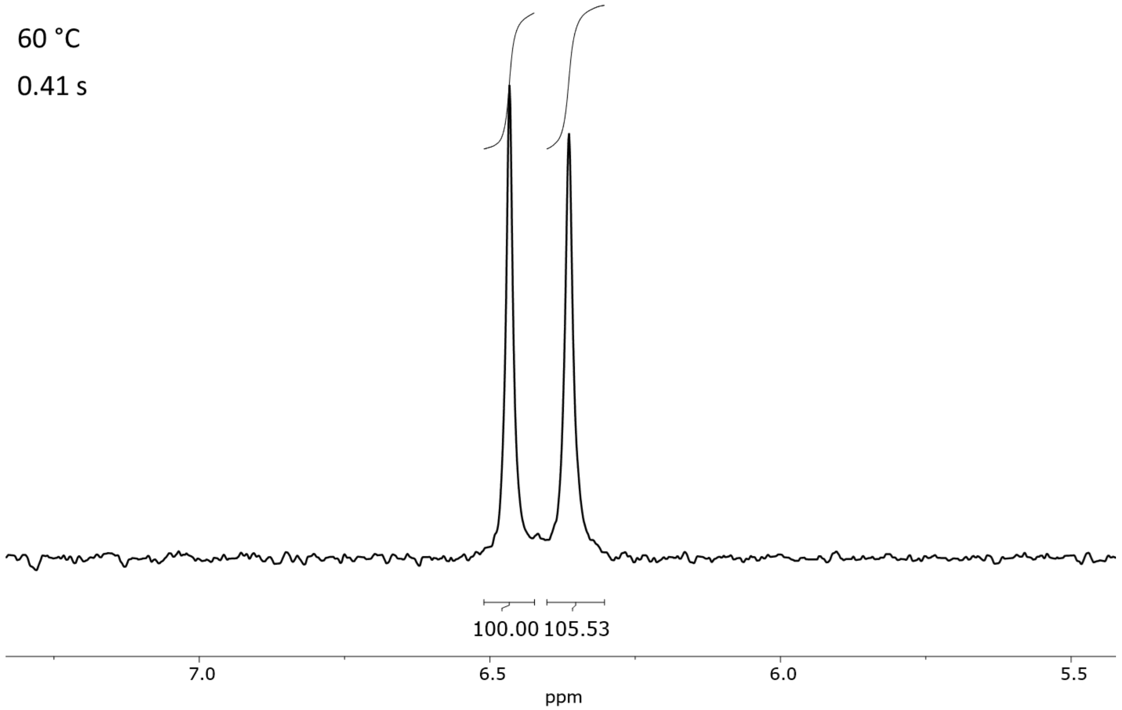

Figure SI62. 1D NOESY experiment of **$H_2$2/V1** (500 MHz, 1:3, 2 mM: 6 mM, $CDCl_3$:CD_3CN, 1:1, v/v) at 60 °C and a mix time of 0.41 s.

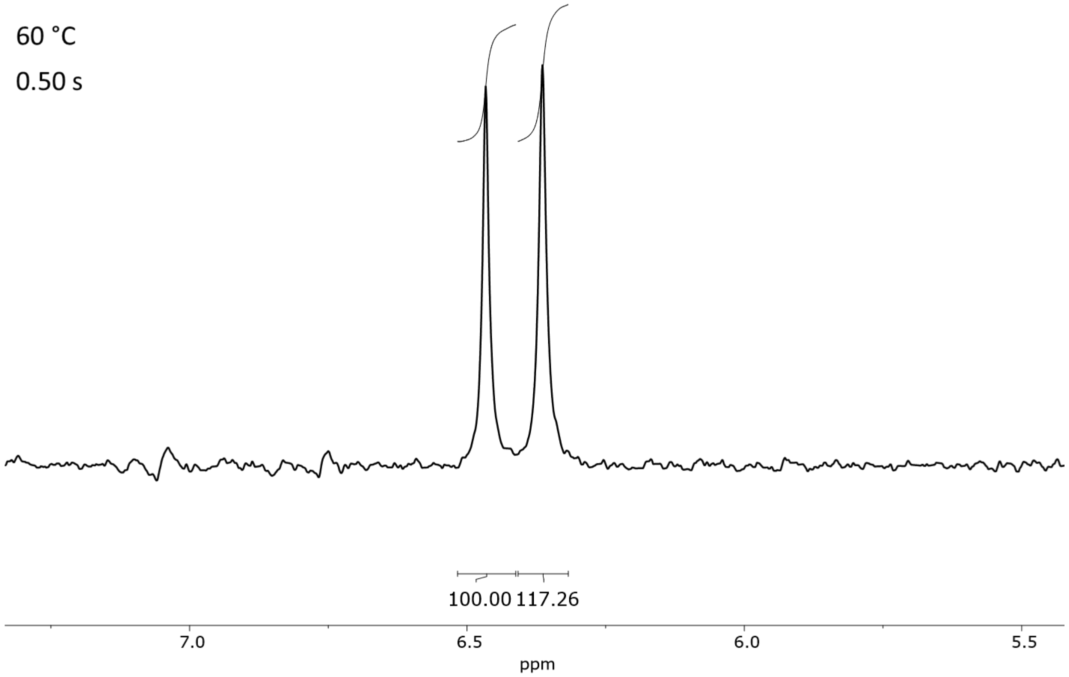

Figure SI63. 1D NOESY experiment of **$H_2$2/V1** (500 MHz, 1:3, 2 mM: 6 mM, $CDCl_3$:CD_3CN, 1:1, v/v) at 60 °C and a mix time of 0.50 s.

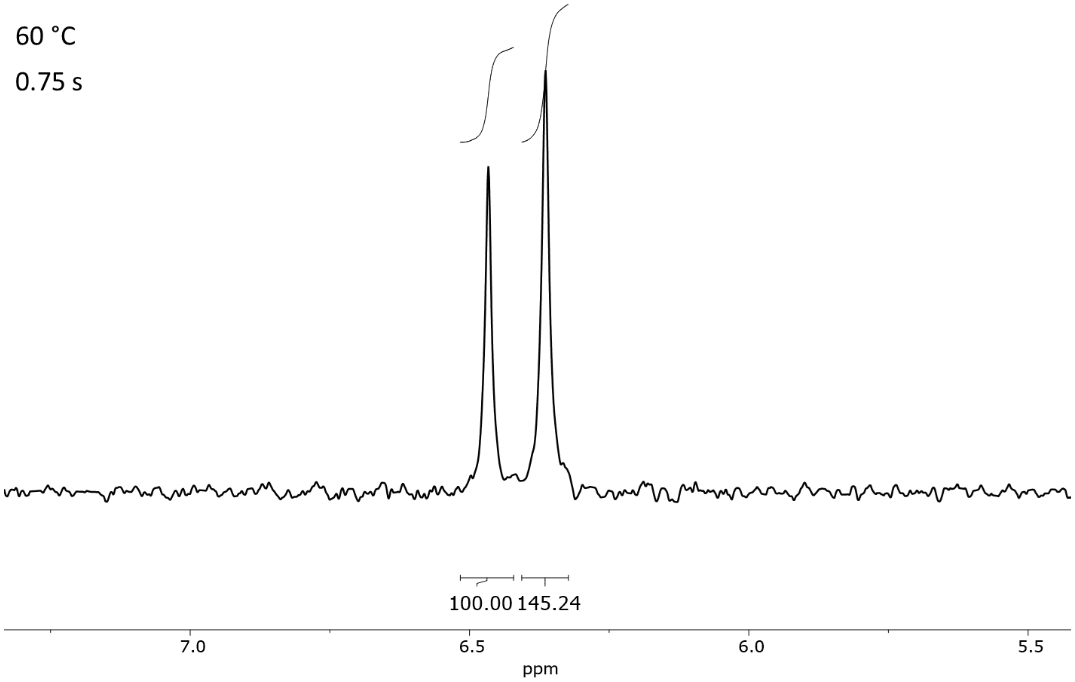

Figure SI64. 1D NOESY experiment of **$H_2$2/V1** (500 MHz, 1:3, 2 mM: 6 mM, $CDCl_3$:CD_3CN, 1:1, v/v) at 60 °C and a mix time of 0.75 s.

Table SI8. Overview of 1D EXSY data for **$H_2$2/V1** at 60 °C. The integrals are shown as well as the decrease in concentration of the (irradiated) minor abundant species and the increase of the major abundant species.

60 °C		Integrals		Percentages		Concentrations	
No. scans	Time (s)	Minor	Major	Minor%	Major%	[Minor] (mM)	[Major] (mM)
512	0.03	100	6.97	93.484154	6.515846	0.6919697	0.0482303
512	0.045	100	10.08	90.843023	9.156977	0.6724201	0.0677799
512	0.06	100	14.19	87.573343	12.42666	0.6482179	0.0919821
512	0.075	100	18.14	84.645336	15.35466	0.6265448	0.1136552
512	0.09	100	22.88	81.380208	18.61979	0.6023763	0.1378237
512	0.125	100	31.81	75.866778	24.13322	0.5615659	0.1786341
512	0.18	100	44.76	69.079856	30.92014	0.5113291	0.2288709
512	0.25	100	66.49	60.063667	39.93633	0.4445913	0.2956087
512	0.33	100	86.21	53.702809	46.29719	0.3975082	0.3426918
512	0.41	100	105.53	48.654698	51.3453	0.3601421	0.3800579
512	0.5	100	117.26	46.027801	53.9722	0.3406978	0.3995022
512	0.75	100	145.24	40.776382	59.22362	0.3018268	0.4383732

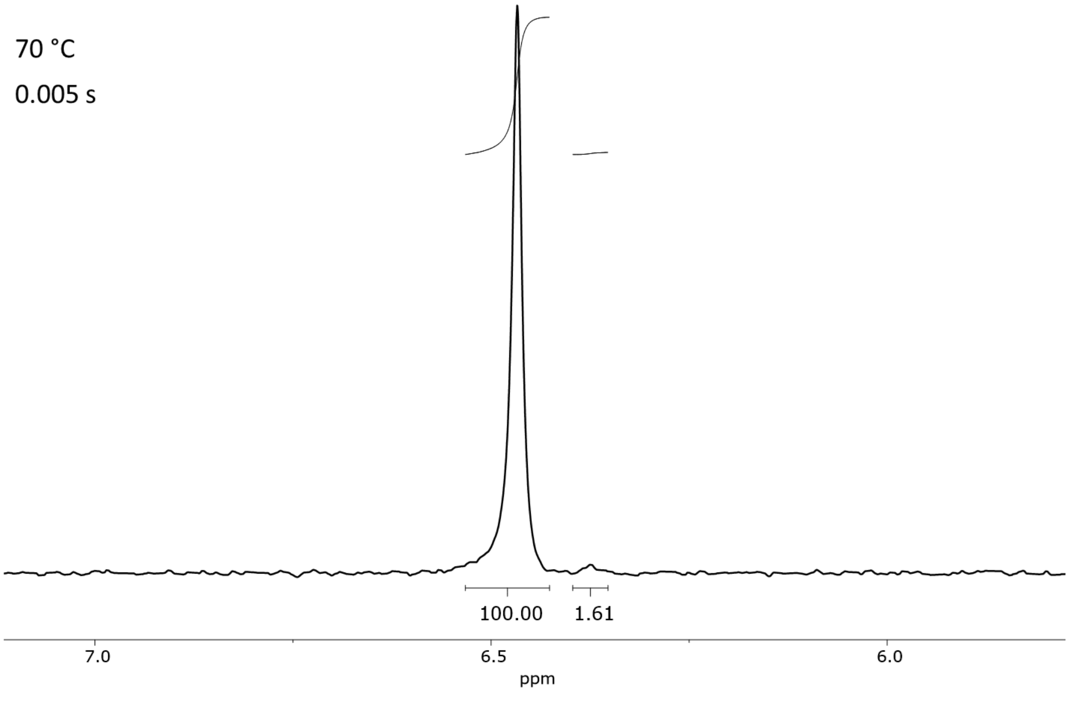

Figure SI65. 1D NOESY experiment of **$H_2$2/V1** (500 MHz, 1:3, 2 mM: 6 mM, $CDCl_3$:CD_3CN, 1:1, v/v) at 70 °C and a mix time of 0.005 s.

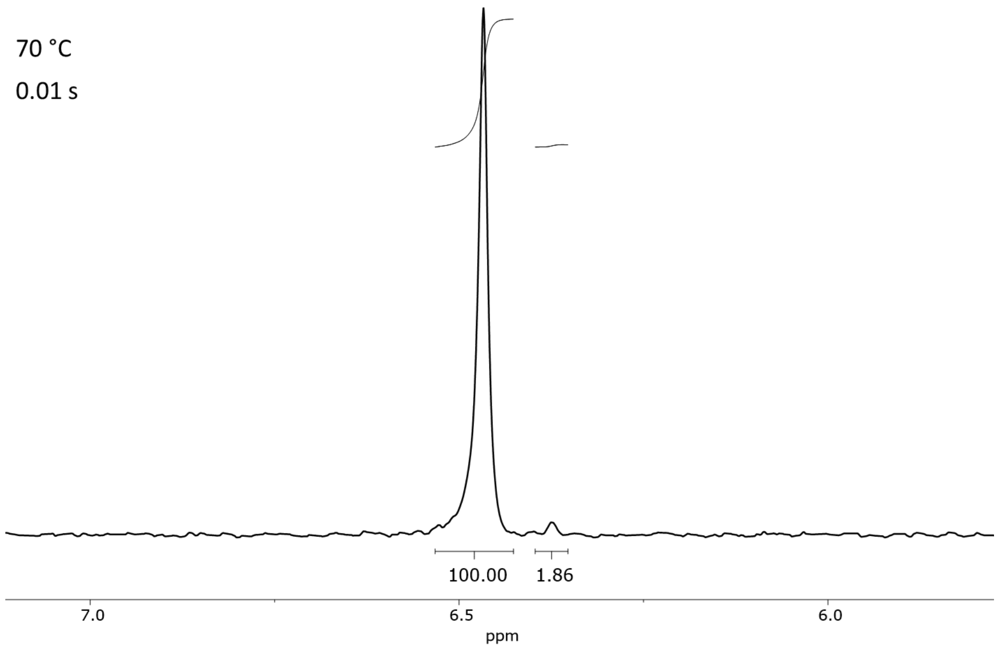

Figure SI66. 1D NOESY experiment of **$H_2$2/V1** (500 MHz, 1:3, 2 mM: 6 mM, $CDCl_3$:CD_3CN, 1:1, v/v) at 70 °C and a mix time of 0.01 s.

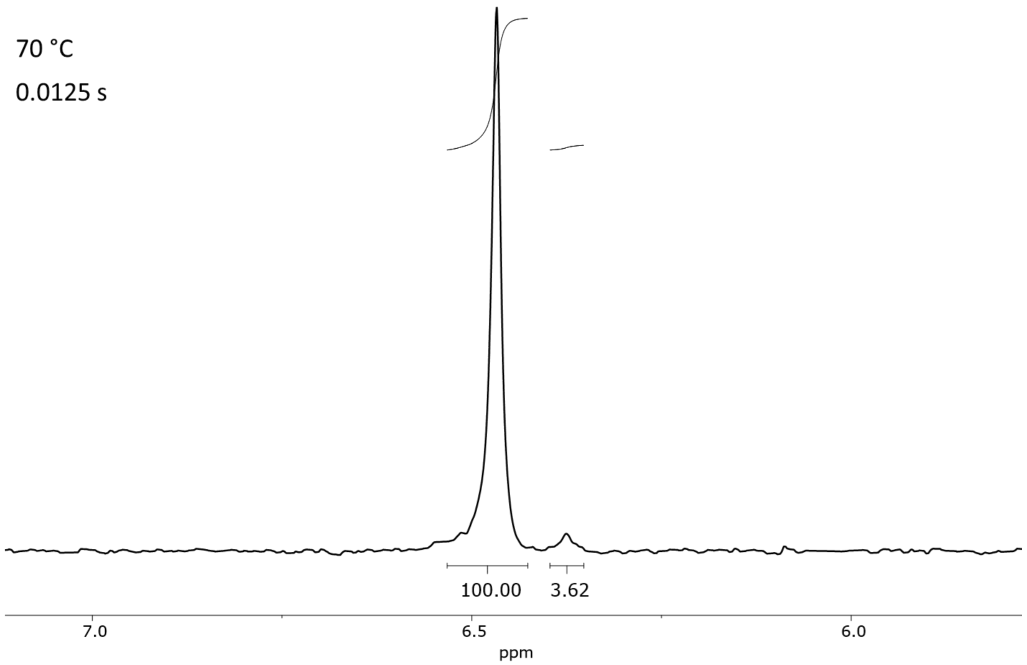

Figure SI67. 1D NOESY experiment of **$H_2$2/V1** (500 MHz, 1:3, 2 mM: 6 mM, $CDCl_3$:CD_3CN, 1:1, v/v) at 70 °C and a mix time of 0.0125 s.

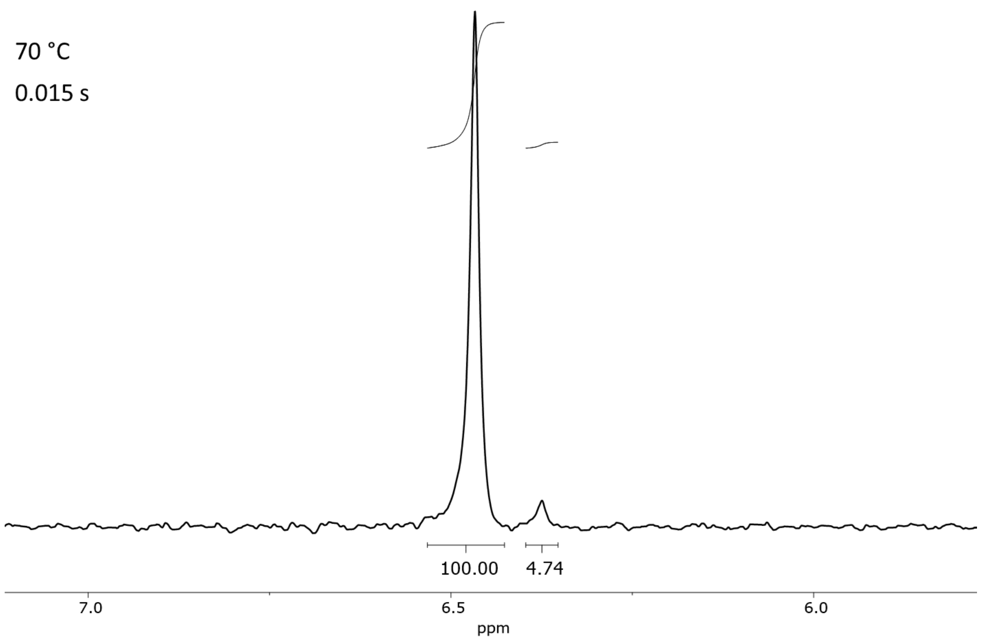

Figure SI68. 1D NOESY experiment of **$H_2$2/V1** (500 MHz, 1:3, 2 mM: 6 mM, $CDCl_3$:CD_3CN, 1:1, v/v) at 70 °C and a mix time of 0.015 s.

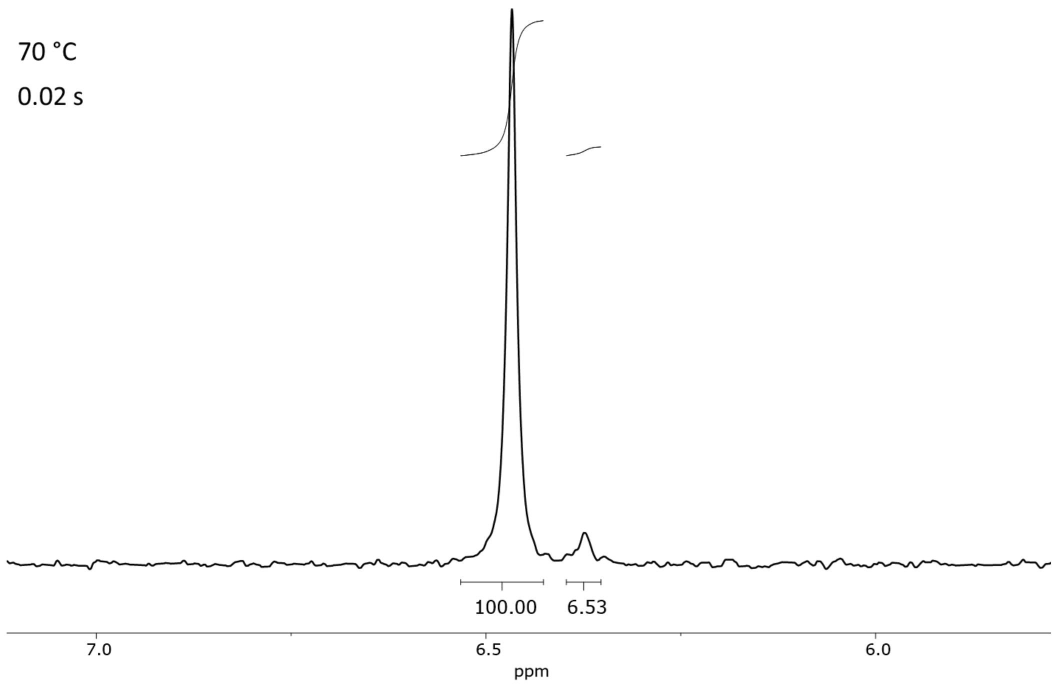

Figure SI69. 1D NOESY experiment of **$H_2$2/V1** (500 MHz, 1:3, 2 mM: 6 mM, $CDCl_3$:CD_3CN, 1:1, v/v) at 70 °C and a mix time of 0.02 s.

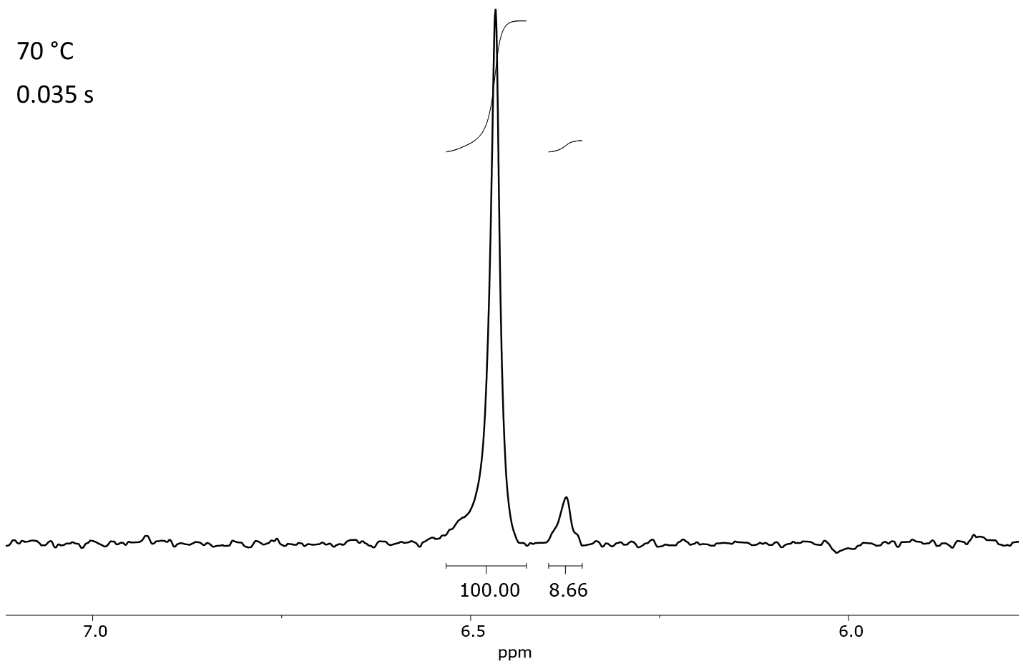

Figure SI70. 1D NOESY experiment of **$H_2$2/V1** (500 MHz, 1:3, 2 mM: 6 mM, $CDCl_3$:CD_3CN, 1:1, v/v) at 70 °C and a mix time of 0.035 s.

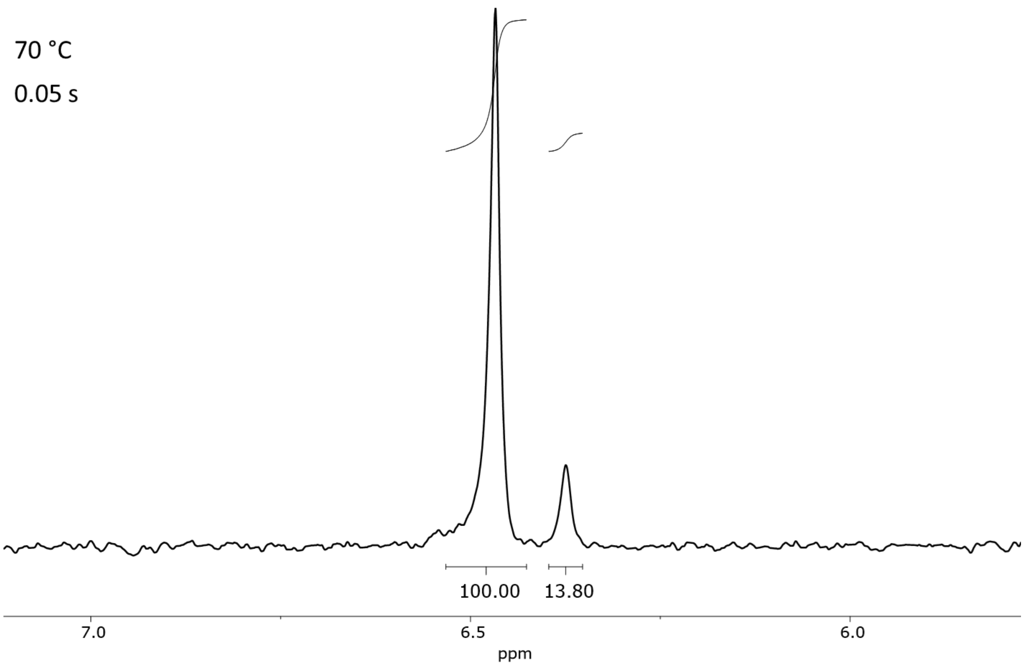

Figure SI71. 1D NOESY experiment of **$H_2$2/V1** (500 MHz, 1:3, 2 mM: 6 mM, $CDCl_3$:CD_3CN, 1:1, v/v) at 70 °C and a mix time of 0.05 s.

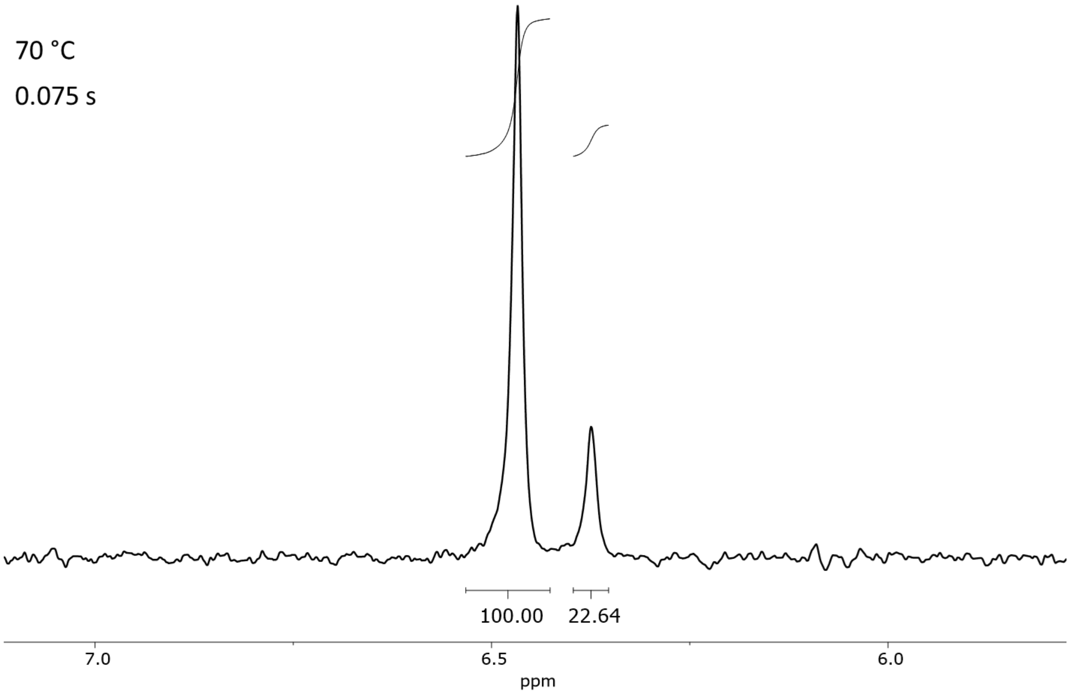

Figure SI72. 1D NOESY experiment of **H$_2$2/V1** (500 MHz, 1:3, 2 mM: 6 mM, $CDCl_3$:CD_3CN, 1:1, v/v) at 70 °C and a mix time of 0.075 s.

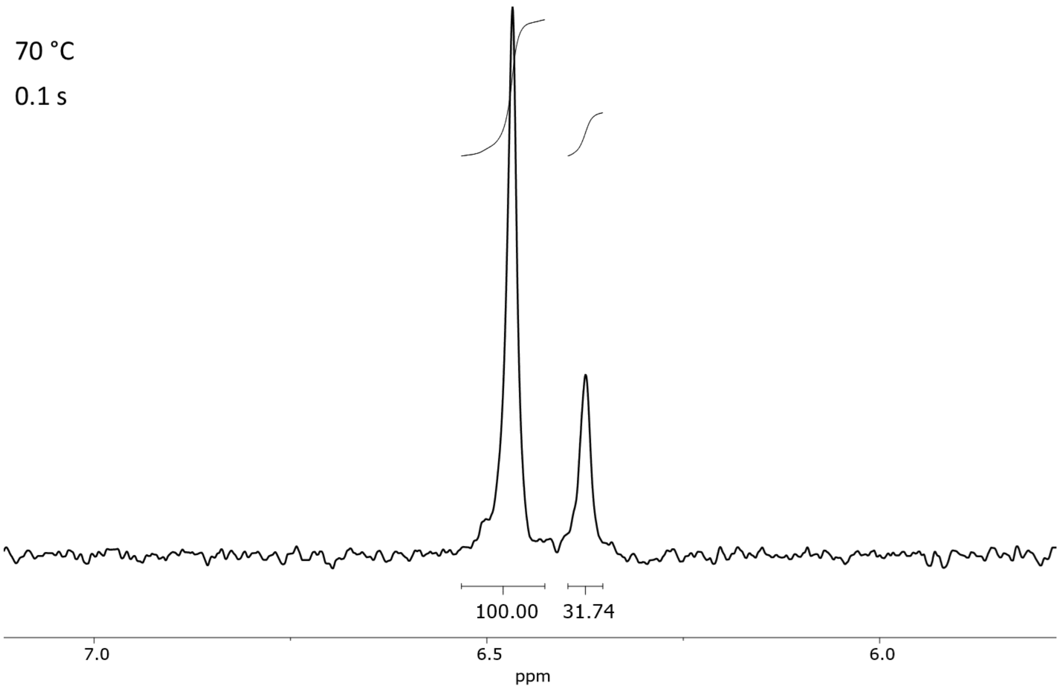

Figure SI73. 1D NOESY experiment of **H$_2$2/V1** (500 MHz, 1:3, 2 mM: 6 mM, $CDCl_3$:CD_3CN, 1:1, v/v) at 70 °C and a mix time of 0.1 s.

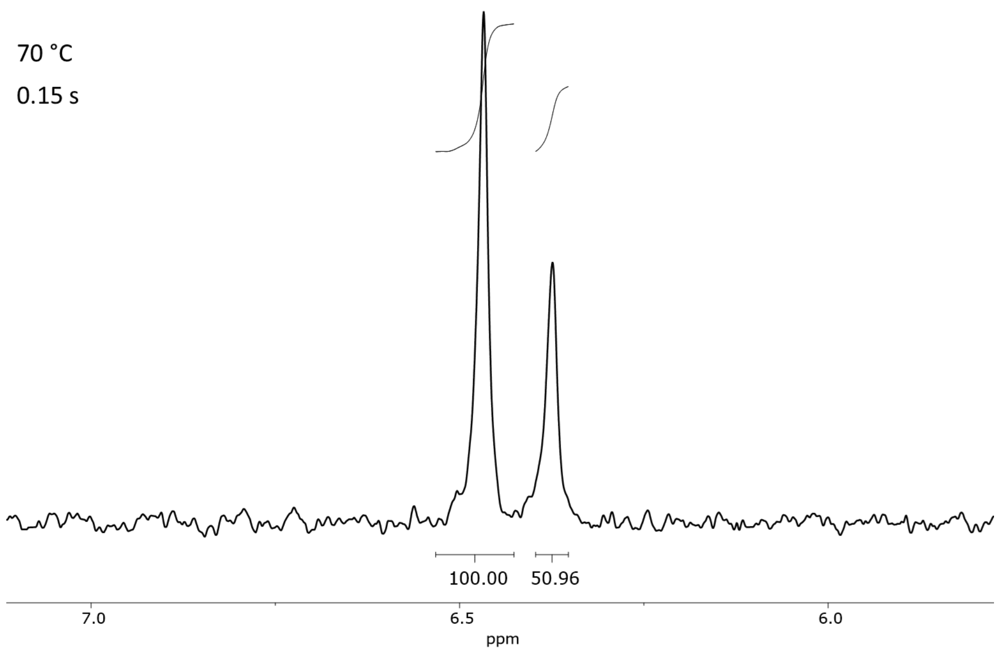

Figure SI74. 1D NOESY experiment of **$H_2$2/V1** (500 MHz, 1:3, 2 mM: 6 mM, $CDCl_3$:CD_3CN, 1:1, v/v) at 70 °C and a mix time of 0.15 s.

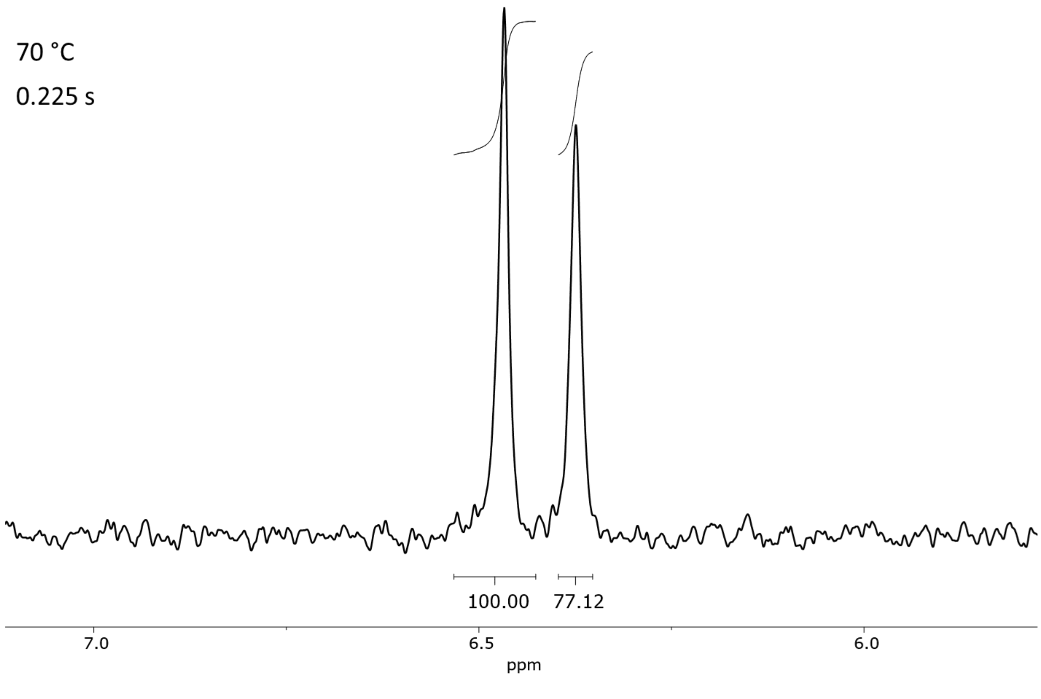

Figure SI75. 1D NOESY experiment of **$H_2$2/V1** (500 MHz, 1:3, 2 mM: 6 mM, $CDCl_3$:CD_3CN, 1:1, v/v) at 70 °C and a mix time of 0.225 s.

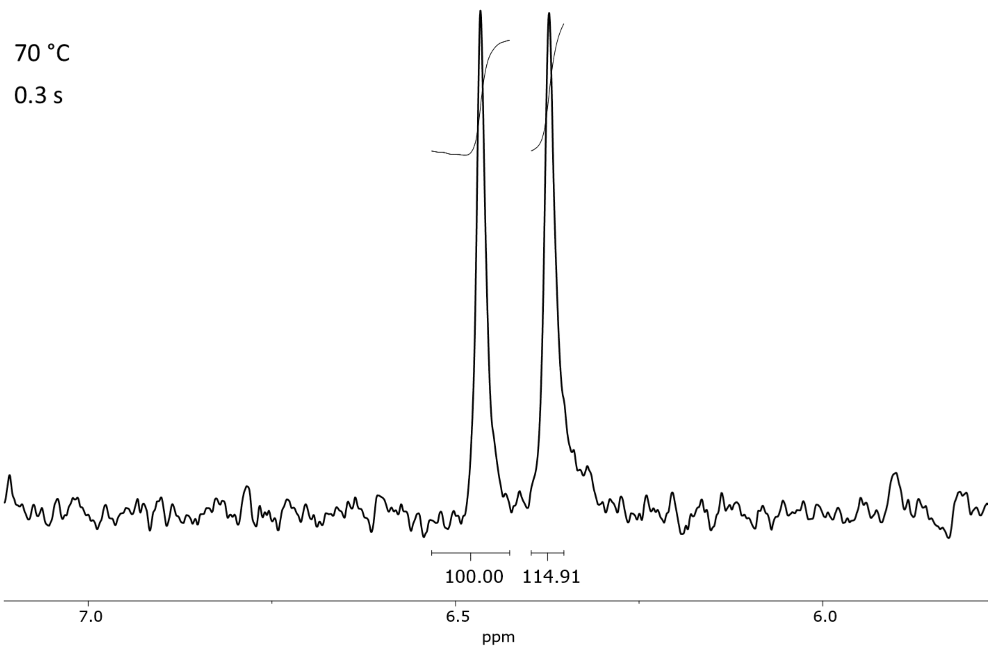

Figure SI76. 1D NOESY experiment of **$H_2$2/V1** (500 MHz, 1:3, 2 mM: 6 mM, $CDCl_3$:CD_3CN, 1:1, v/v) at 70 °C and a mix time of 0.3 s.

Table SI9. Overview of 1D EXSY data for **$H_2$2/V1** at 70 °C. The integrals are shown as well as the decrease in concentration of the (irradiated) minor abundant species and the increase of the major abundant species.

70 °C		Integrals		Percentages		Concentrations	
No. scans	Time (s)	Minor	Major	Minor%	Major%	[Minor] (mM)	[Major] (mM)
512	0.005	100	1.61	98.41551	1.58449	0.765476	0.012324
512	0.01	100	1.86	98.17396	1.826036	0.763597	0.014203
512	0.0125	100	3.62	96.50647	3.493534	0.750627	0.027173
256	0.015	100	4.74	95.47451	4.525492	0.742601	0.035199
256	0.02	100	6.53	93.87027	6.129729	0.730123	0.047677
256	0.035	100	8.66	92.03019	7.969814	0.715811	0.061989
256	0.05	100	13.8	87.87346	12.12654	0.68348	0.09432
128	0.075	100	22.64	81.53947	18.46053	0.634214	0.143586
128	0.1	100	31.74	75.90709	24.09291	0.590405	0.187395
64	0.15	100	50.96	66.24271	33.75729	0.515236	0.262564
64	0.225	100	77.12	56.4589	43.5411	0.439137	0.338663
64	0.3	100	114.91	46.53111	53.46889	0.361919	0.415881

Fits of the 1D EXSY studies of all systems following the change in orientation of the guest in the host

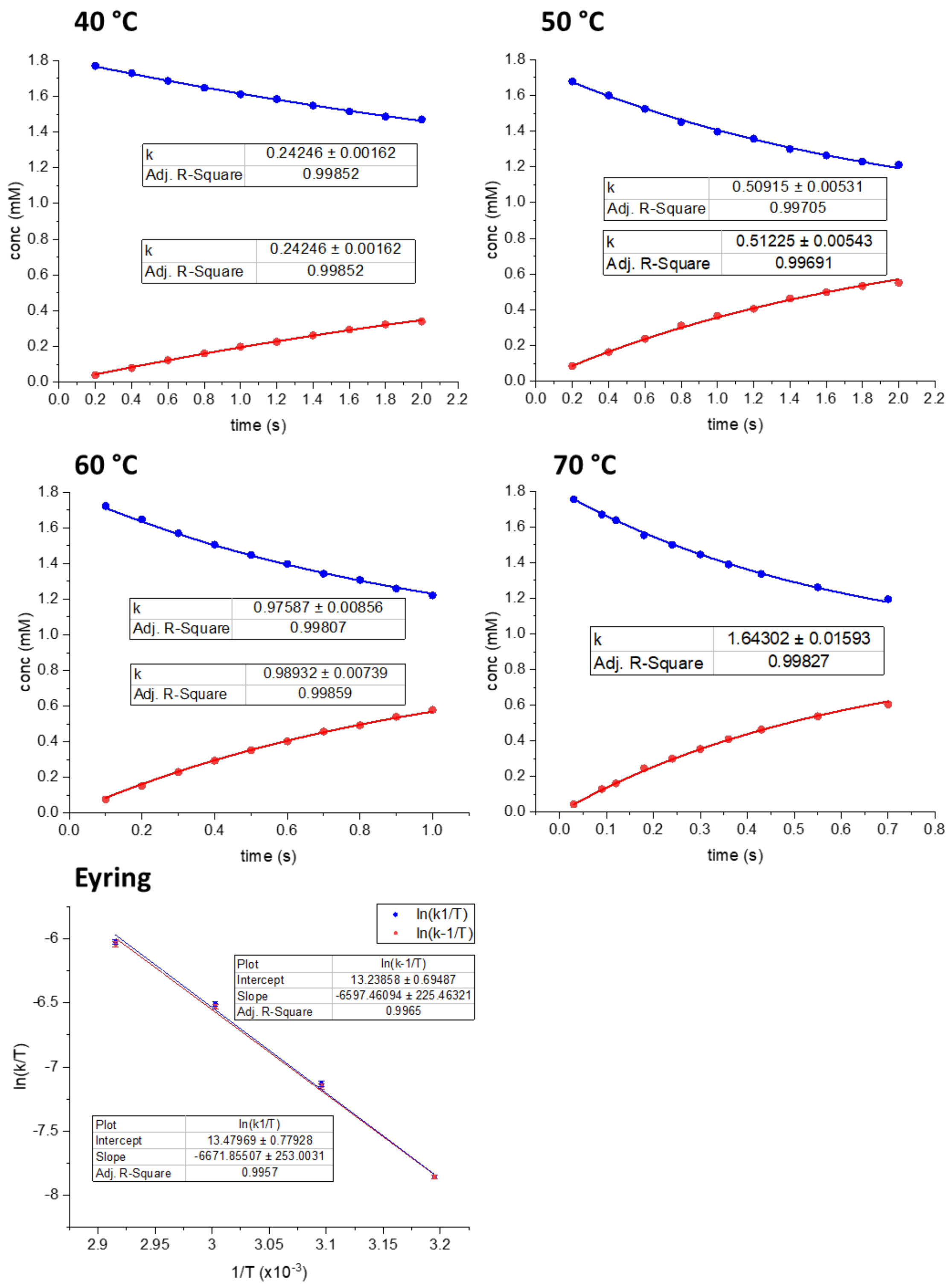

Figure SI77. $H_2$1/V1 (following orientation 1 to orientation 2; host : guest = 1:3, 2 mM : 6 mM).

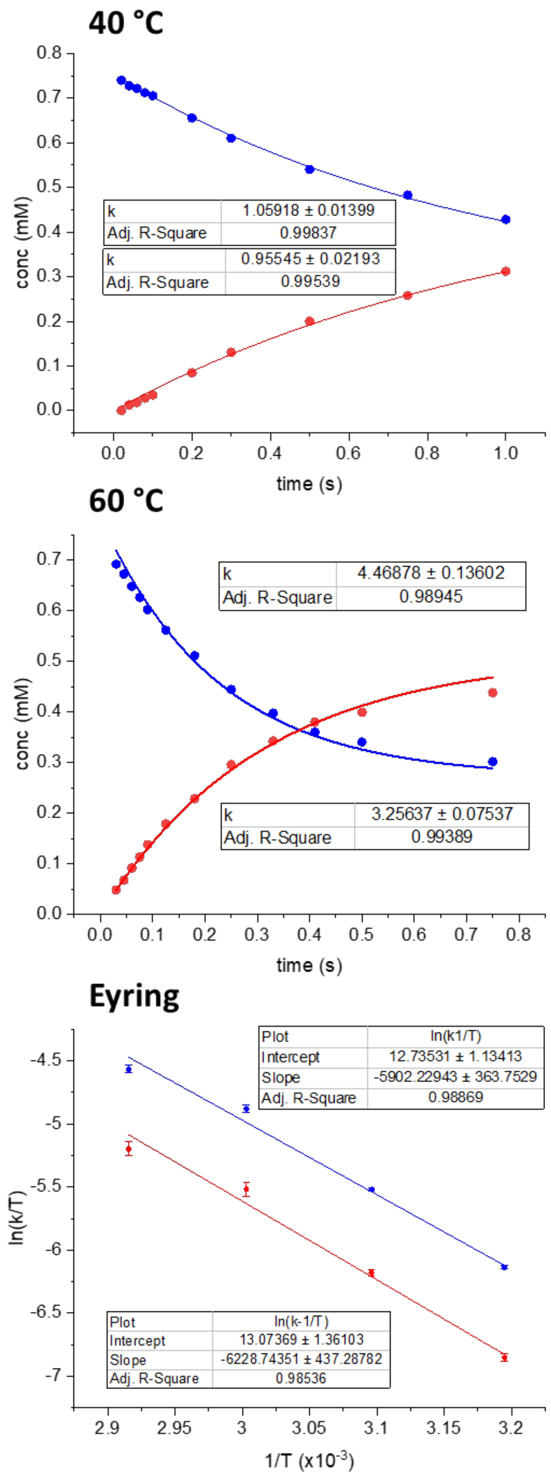

Figure SI78. H_2**2**/**V1** (following the minor abundant complex to the major abundant complex; host : guest = 1:3, 2 mM : 6 mM).

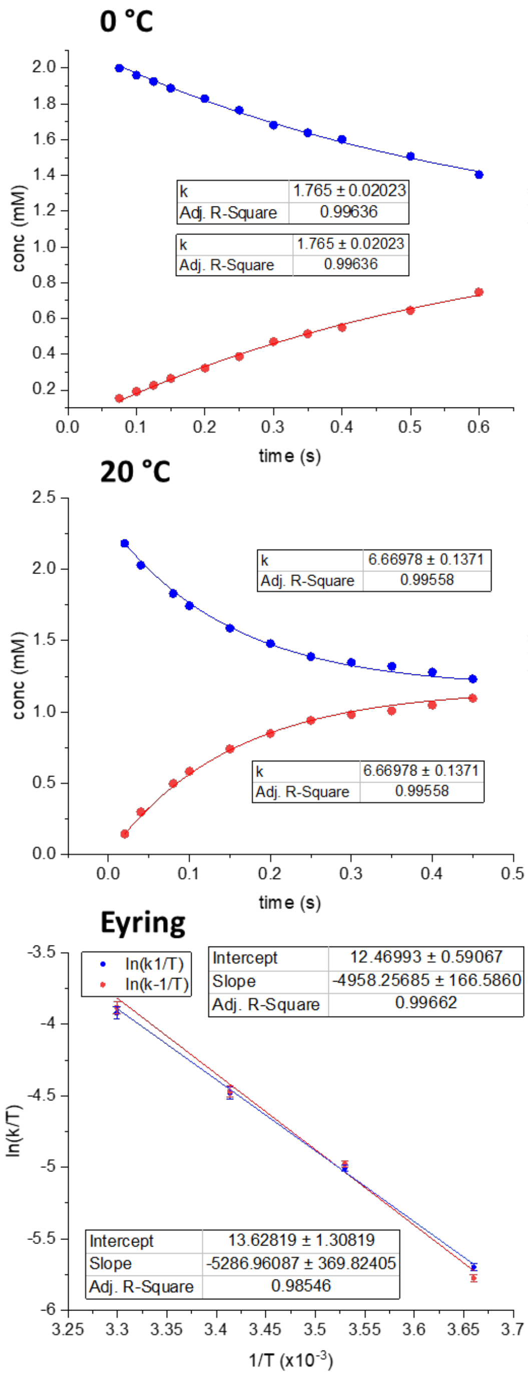

Figure SI79. H_23/V1 (following orientation 1 to orientation 2; host : guest = 1:3, 2 mM : 6 mM).

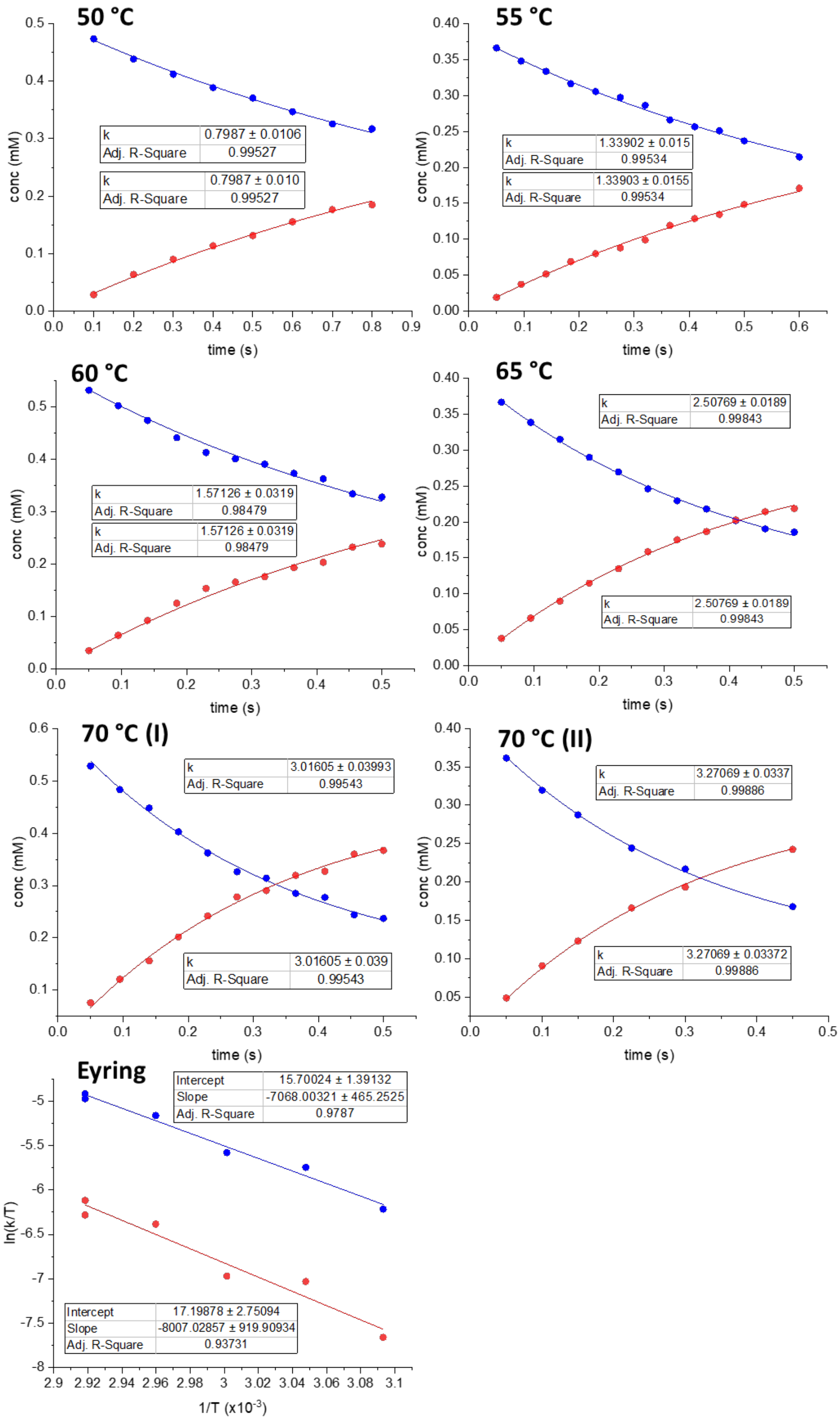

Figure SI80. $\mathbf{H_24/V1}$ (following the minor abundant complex to the major abundant complex; host : guest = 1:3, 2 mM : 6 mM).

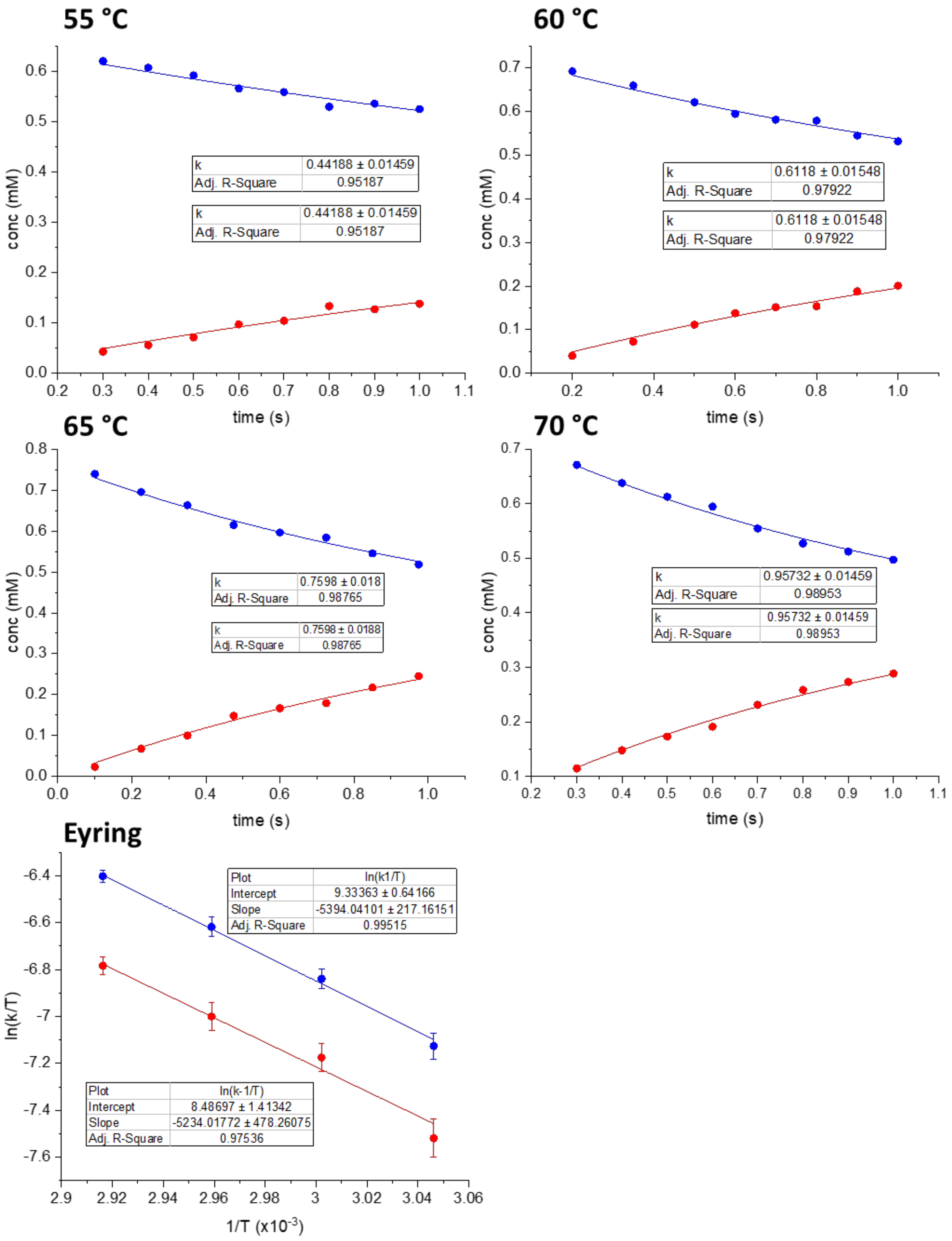

Figure SI81. $H_2$5/V1 (following the minor abundant complex to the major abundant complex; host : guest = 1:3, 2 mM : 6 mM).

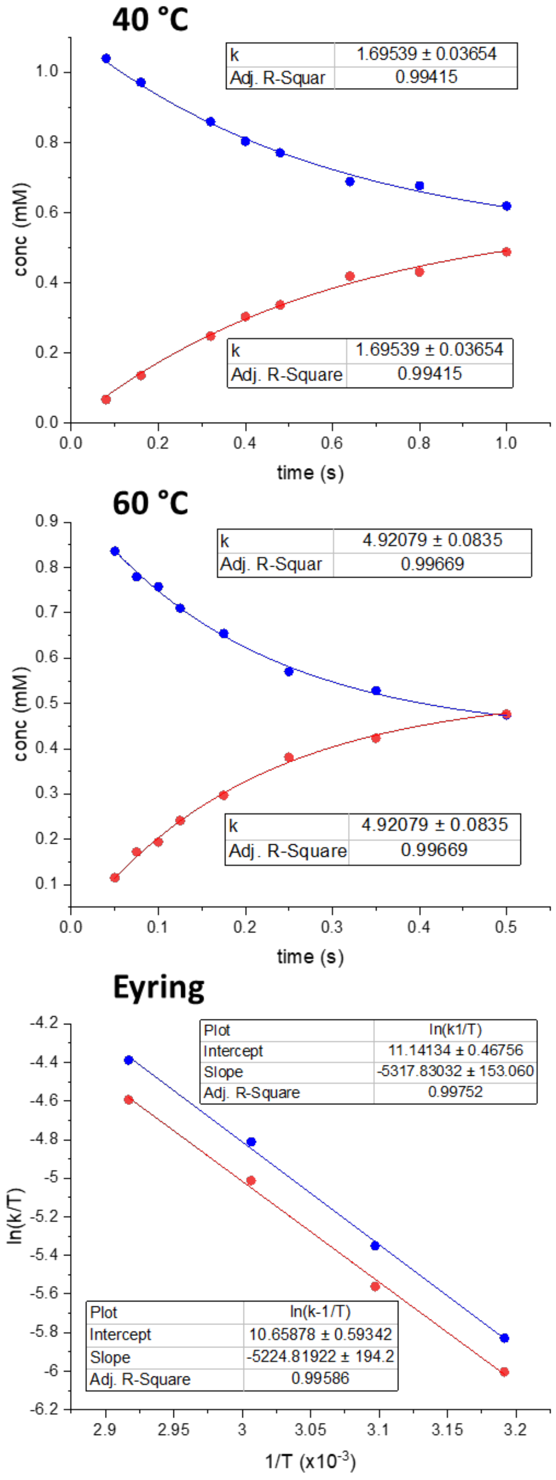

Figure SI82. $H_2$2/V2 (following the minor abundant complex to the major abundant complex; host : guest = 1:3, 2 mM : 6 mM).

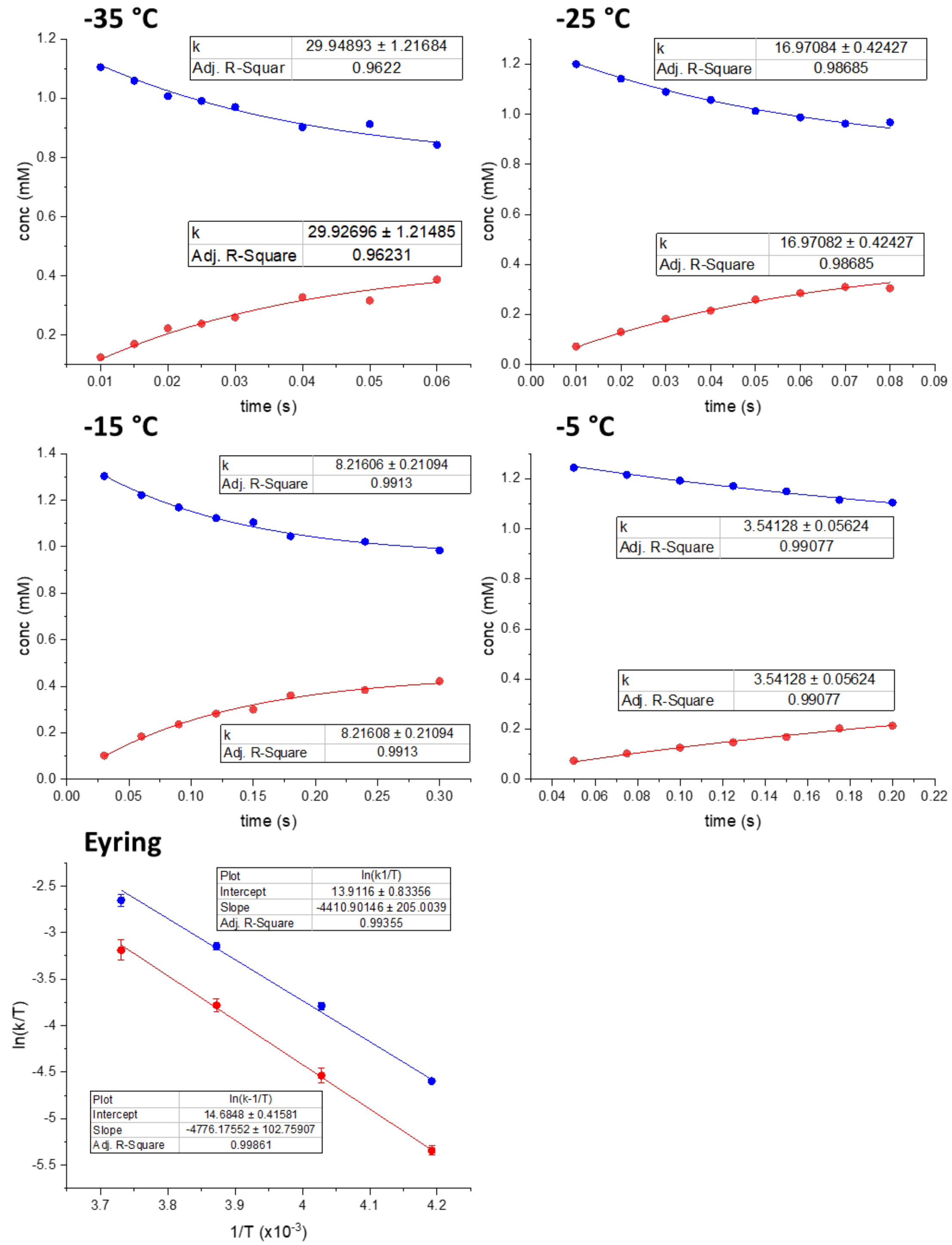

Figure SI83. H_2**2**/**V3** (following the major abundant complex to the minor abundant complex; host : guest = 1:3, 2 mM : 6 mM).

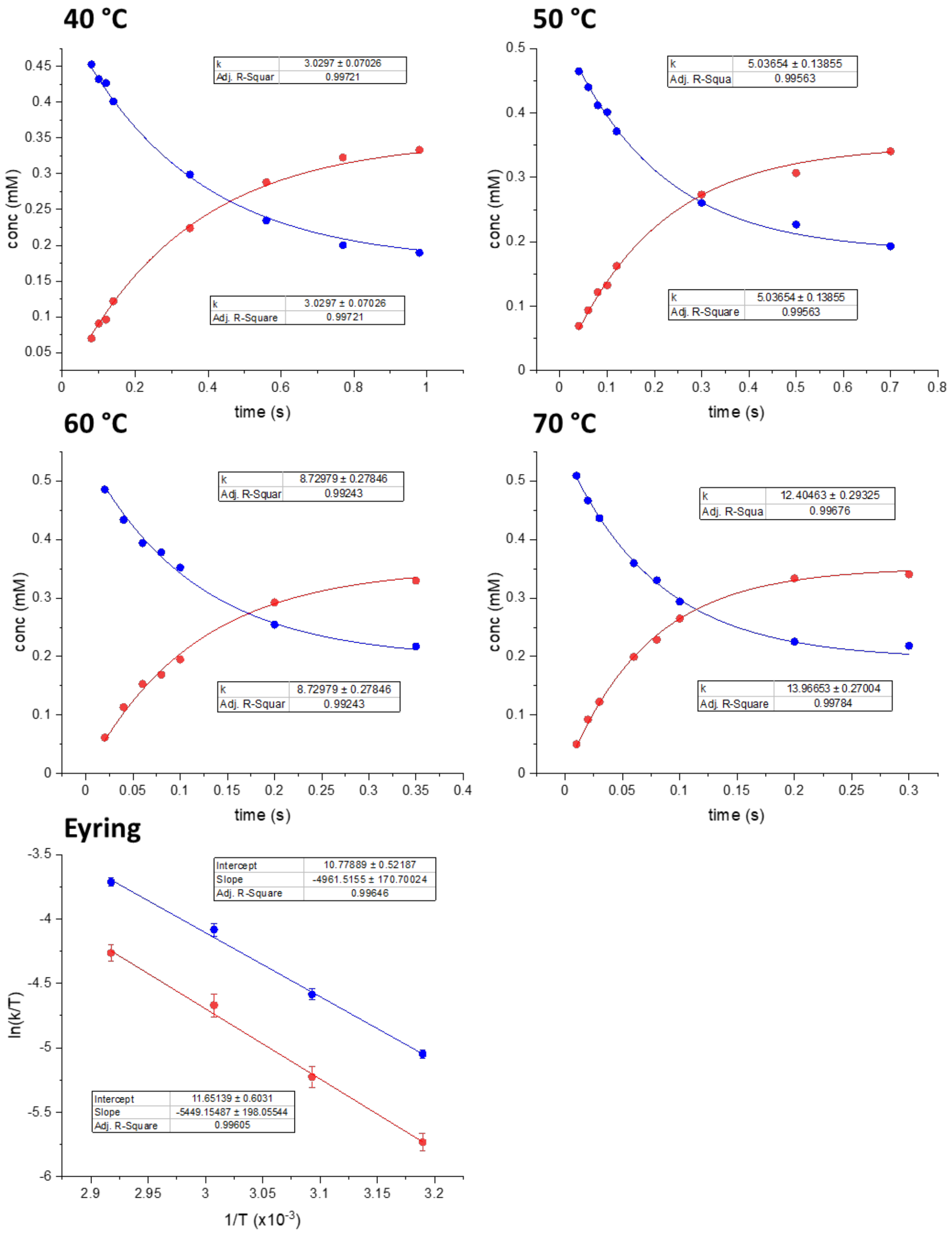

Figure SI84. $H_2$2/V4 (following the minor abundant complex to the major abundant complex; host : guest = 1:3, 2 mM : 6 mM).

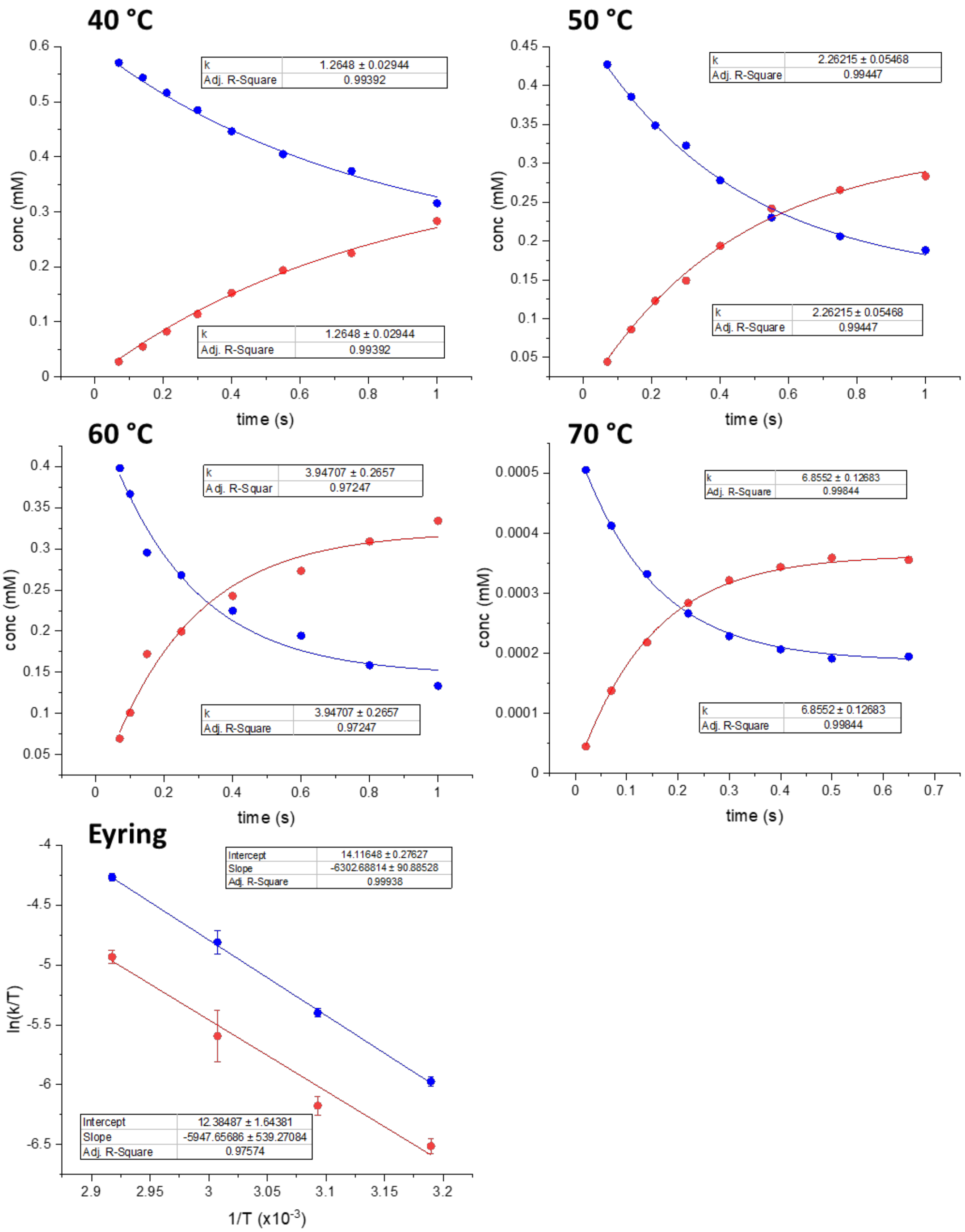

Figure SI85. $H_2$2/V5 (following the minor abundant complex to the major abundant complex; host : guest = 1:3, 2 mM : 6 mM).

Fits of the 1D EXSY studies of all systems following the conversion of bound host to free host: initial rates

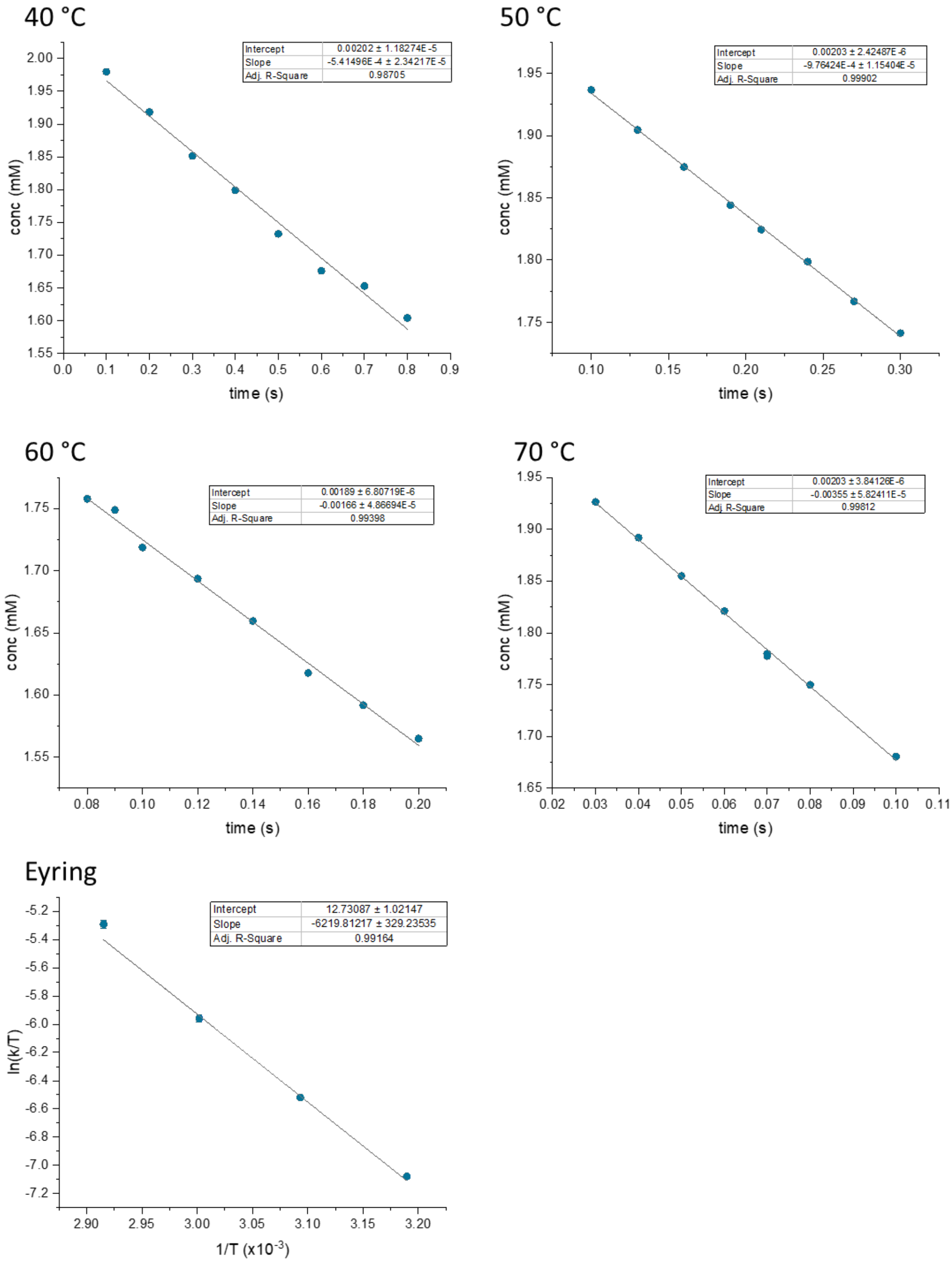

Figure SI86. $H_2$1/V1 (bound to free; 2:1, 4 mM : 2 mM).

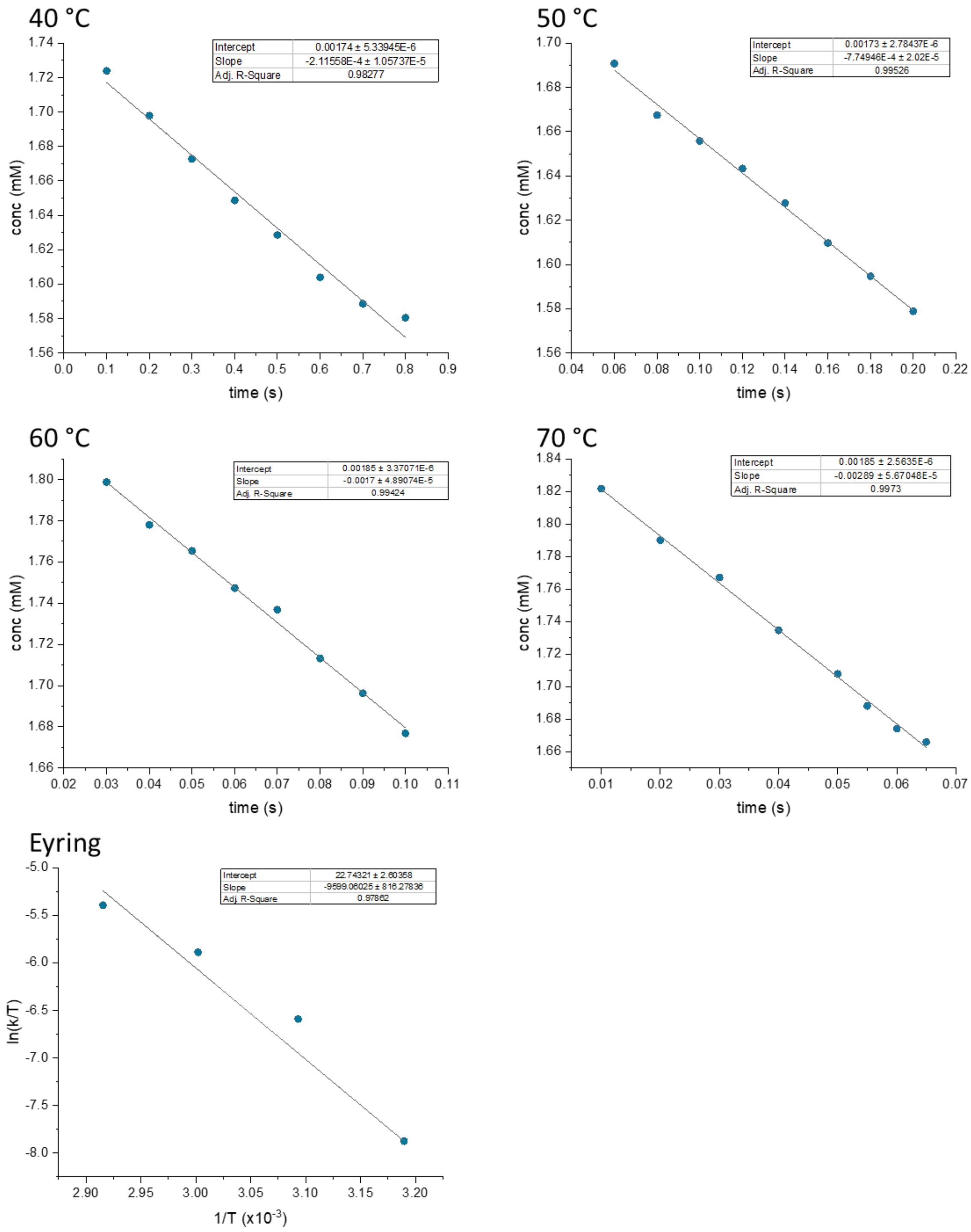

Figure SI87. $H_2$1/V1 (free to bound; 2:1, 4 mM : 2 mM).

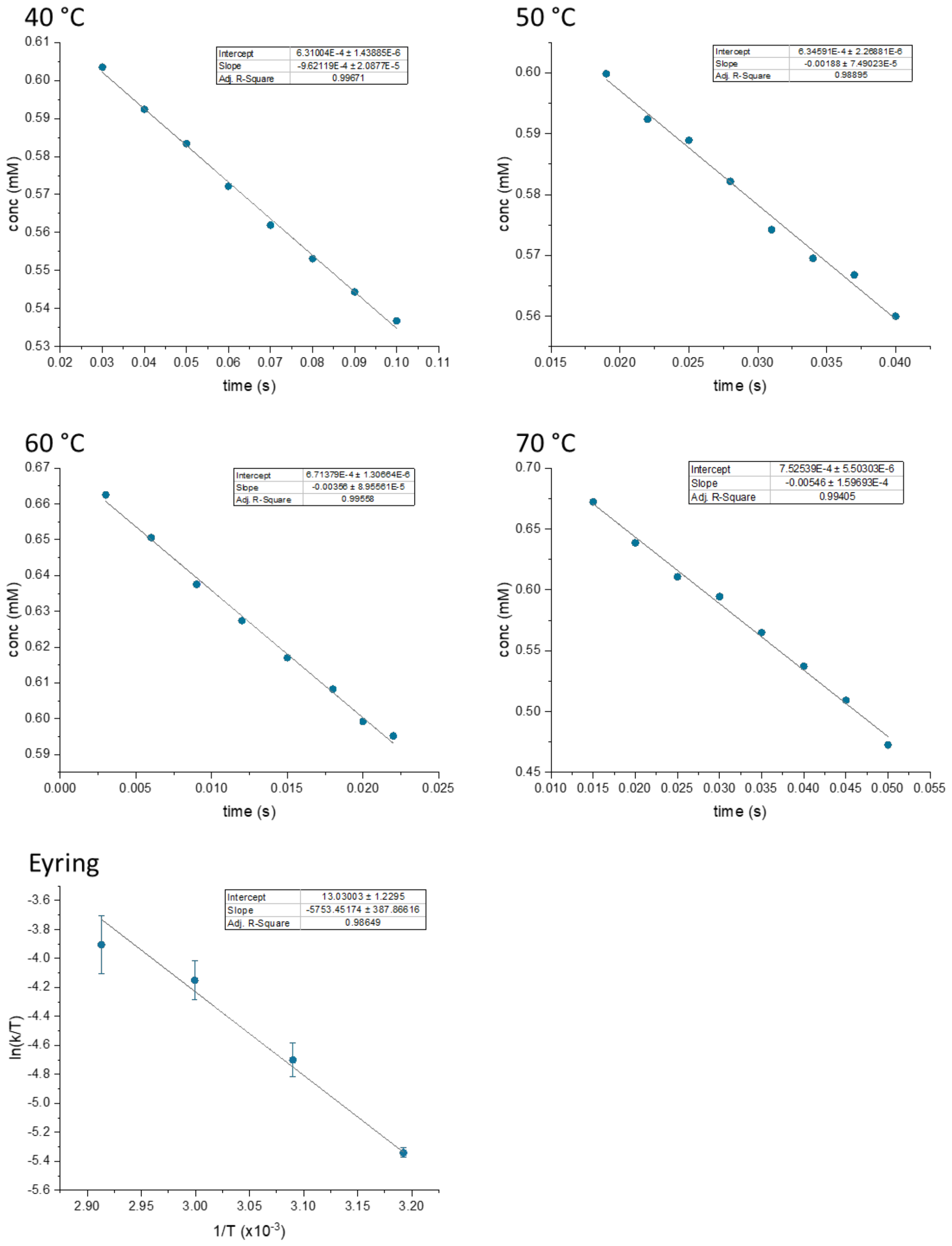

Figure SI88. $H_2$2/V1 (major to free; 2:1, 4 mM : 2 mM).

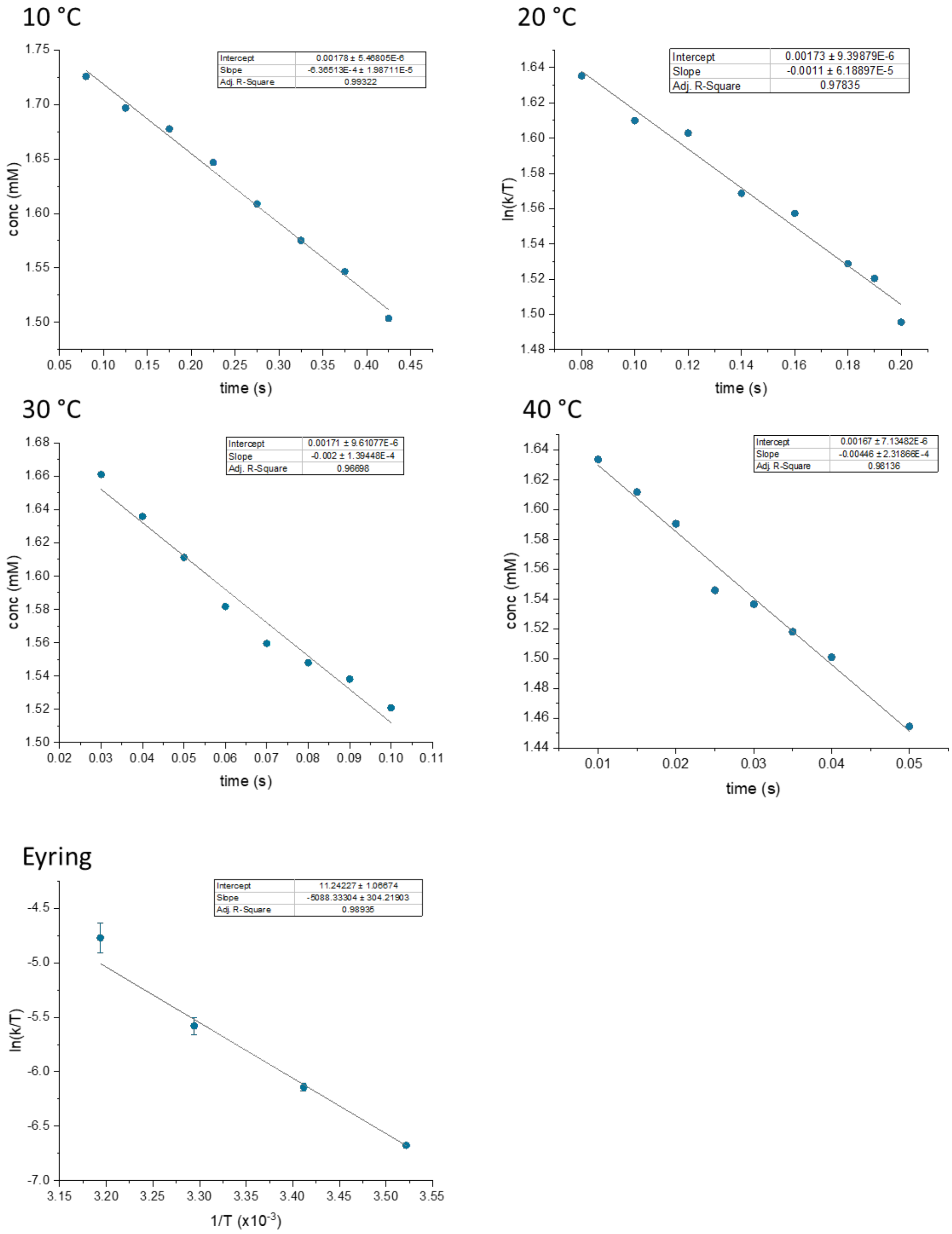

Figure SI89. $H_2$4/V1 (major to free; 2:1, 4 mM : 2 mM).

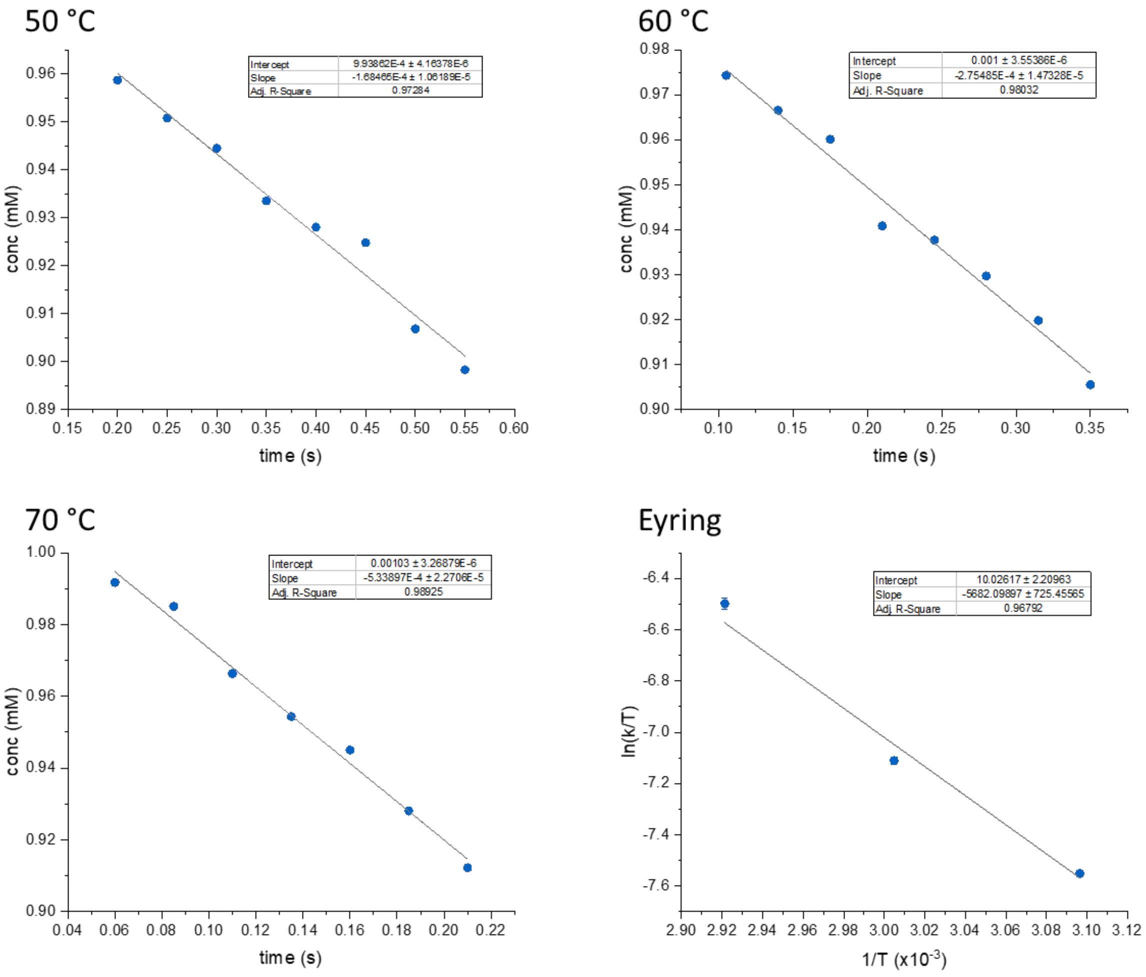

Figure SI90. $H_2$5/V1 (major to free; 2:1, 4 mM : 2 mM).

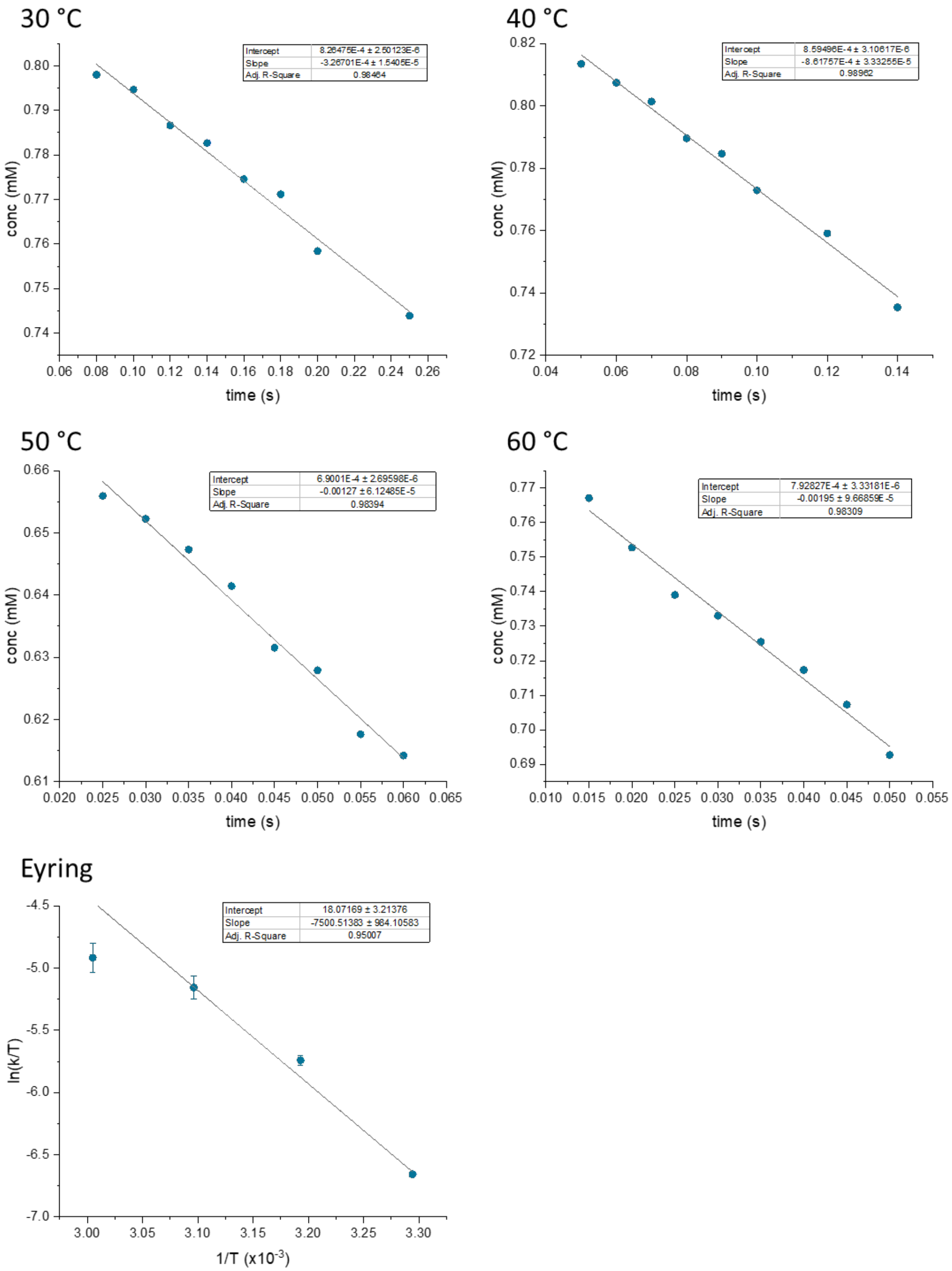

Figure SI91. $H_2$2/V2 (major to free; 2:1, 4 mM : 2 mM).

Overview of chemical shifts of different host/guest complexes

Table SI10. Chemical shifts of the 4,4'-bipyridyl protons for different host/guest systems, with **V1** as the guest and varying hosts (500 MHz, $CDCl_3$:CD_3CN, 1:1, v/v, host : guest, 1:3, 2 mM : 6 mM). The major and minor abundant complexes are denoted by (maj) and (min), respectively.

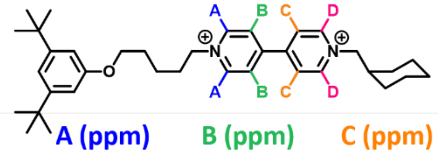

Host	A (ppm)	B (ppm)	C (ppm)	D (ppm)
Free **V1**	8.94	8.40	8.40	8.86
H_2**1**	4.82	3.16	5.30	7.82
H_2**2** (maj)	4.45	2.72	5.58	8.32
H_2**2** (min)	5.00	3.55	5.34	7.67
H_2**3**	4.66	2.92	5.55	8.19
H_2**4** (maj)	4.47	2.51	5.51	8.49
H_2**4** (min)	5.32	2.78	5.44	8.43
H_2**5** (maj)	4.33	2.77	5.59	7.32
H_2**5** (min)	5.28	3.41	4.94	8.20

Table SI11. Chemical shifts of the 4,4'-bipyridyl protons for different host/guest systems, with host H_2**2** and varying guests (500 MHz, $CDCl_3$:CD_3CN, 1:1, v/v, host : guest, 1:3, 2 mM : 6 mM). The major and minor abundant complexes are denoted by (maj) and (min), respectively.

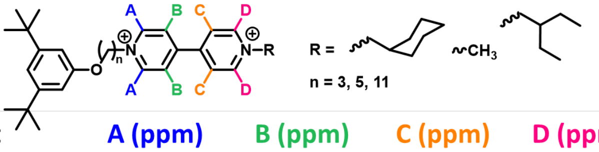

Host	A (ppm)	B (ppm)	C (ppm)	D (ppm)
Free **V1**	8.94	8.40	8.40	8.86
H_2**2** (maj)	4.45	2.72	5.58	8.32
H_2**2** (min)	5.00	3.55	5.34	7.67
Free **V2**	8.97	8.46	8.46	8.87
H_2**2** (maj)	4.57	2.52	5.59	8.33
H_2**2** (min)	4.87	4.04	5.90	7.90
Free **V3**	8.94	8.43	8.40	8.86
H_2**2** (maj)	4.18	2.65	3.52	8.25
H_2**2** (min)	7.20	4.81	5.90	5.20
Free **V4**	8.92	8.36	8.36	8.86
H_2**2** (maj)	4.31	2.69	5.60	8.37
H_2**2** (min)	7.86	5.47	3.47	4.80
Free **V5**	8.89	8.36	8.36	8.82
H_2**2** (maj)	4.40	2.68	5.57	8.32
H_2**2** (min)	7.60	5.25	3.50	5.01

Determination of the guest orientations by 2D ROESY and HSQC

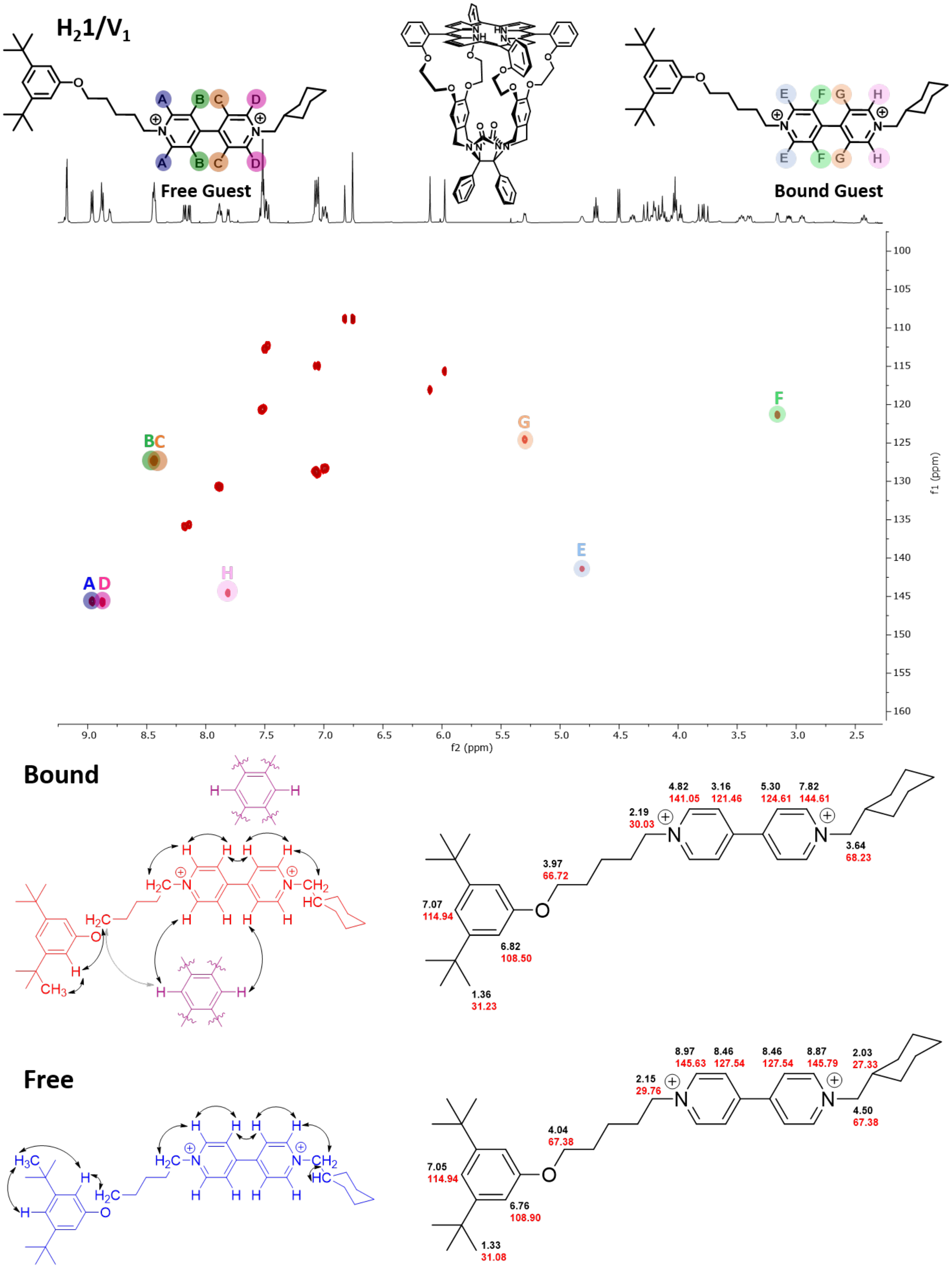

Figure SI92. Top: ^{13}C-^{1}H HSQC of **H_21/V1** (^{1}H: 500 MHz, ^{13}C: 125 MHz, $CDCl_3$:CD_3CN, 1:1, v/v, host : guest, 1:3, 2 mM : 6 mM) in which the protons of the free and bound guests are shown in corresponding colors; bottom: the assigned shifts in ppm for the free and bound guests with the corresponding NOE interactions that were measured by 2D ROESY.

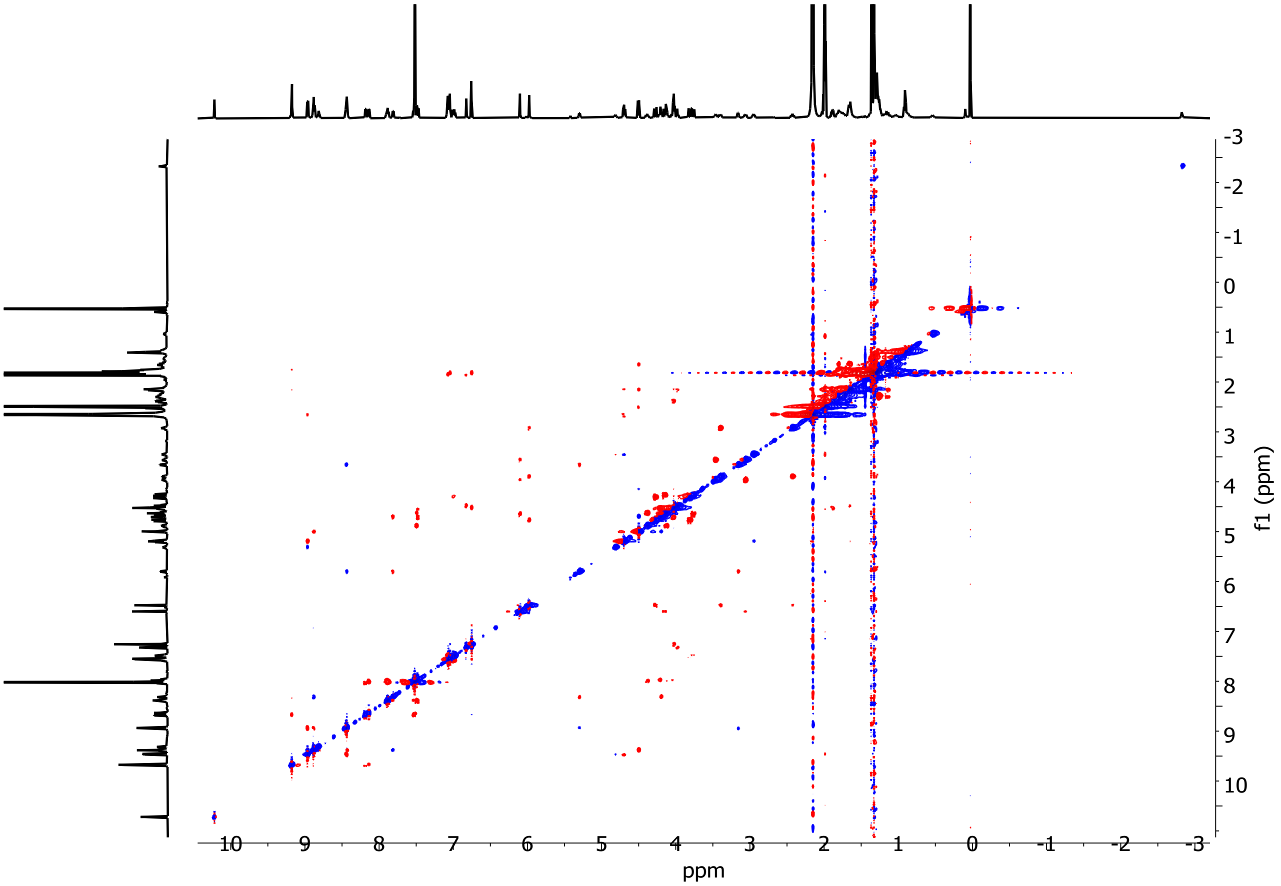

Figure SI93. Full ^{1}H-^{1}H 2D ROESY spectrum of **H$_2$1/V1** (^{1}H: 500 MHz, $CDCl_3$:CD_3CN, 1:1, v/v, host : guest, 1:3, 2 mM : 6 mM, T = 298 K). Exchange cross peaks generally have the same color as the diagonal, which is in this case blue.

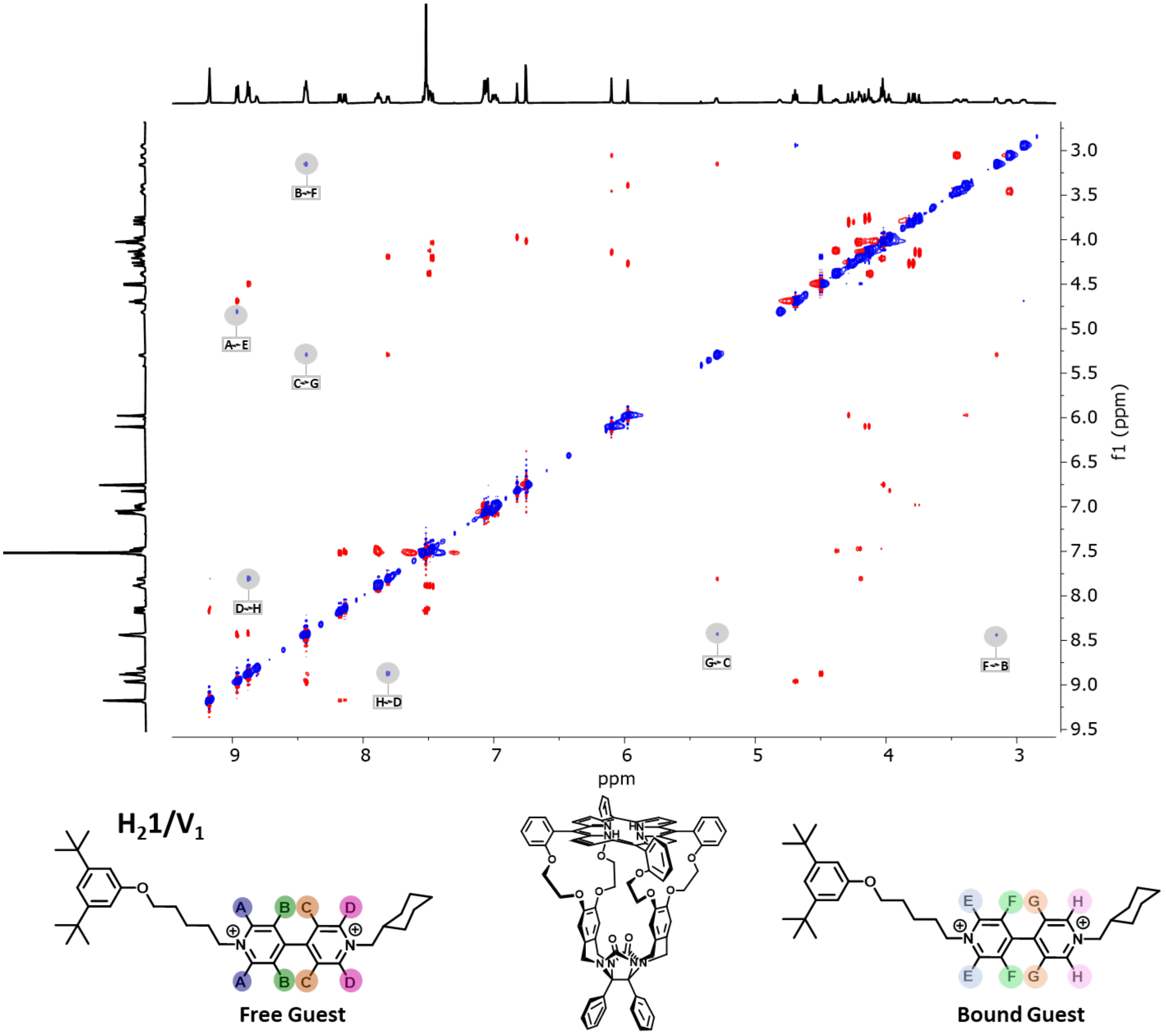

Figure SI94. Zoomed region of ^1H-^1H 2D ROESY spectrum of **$H_2$1/V1** (^1H: 500 MHz, $CDCl_3$:CD_3CN, 1:1, v/v, host : guest, 1:3, 2 mM : 6 mM, T = 298 K) showing relevant guest exchange interactions indicated with A-H. ^1H-^1H 2D ROESY spectrum. Exchange cross peaks generally have the same color as the diagonal, which is in this case blue.

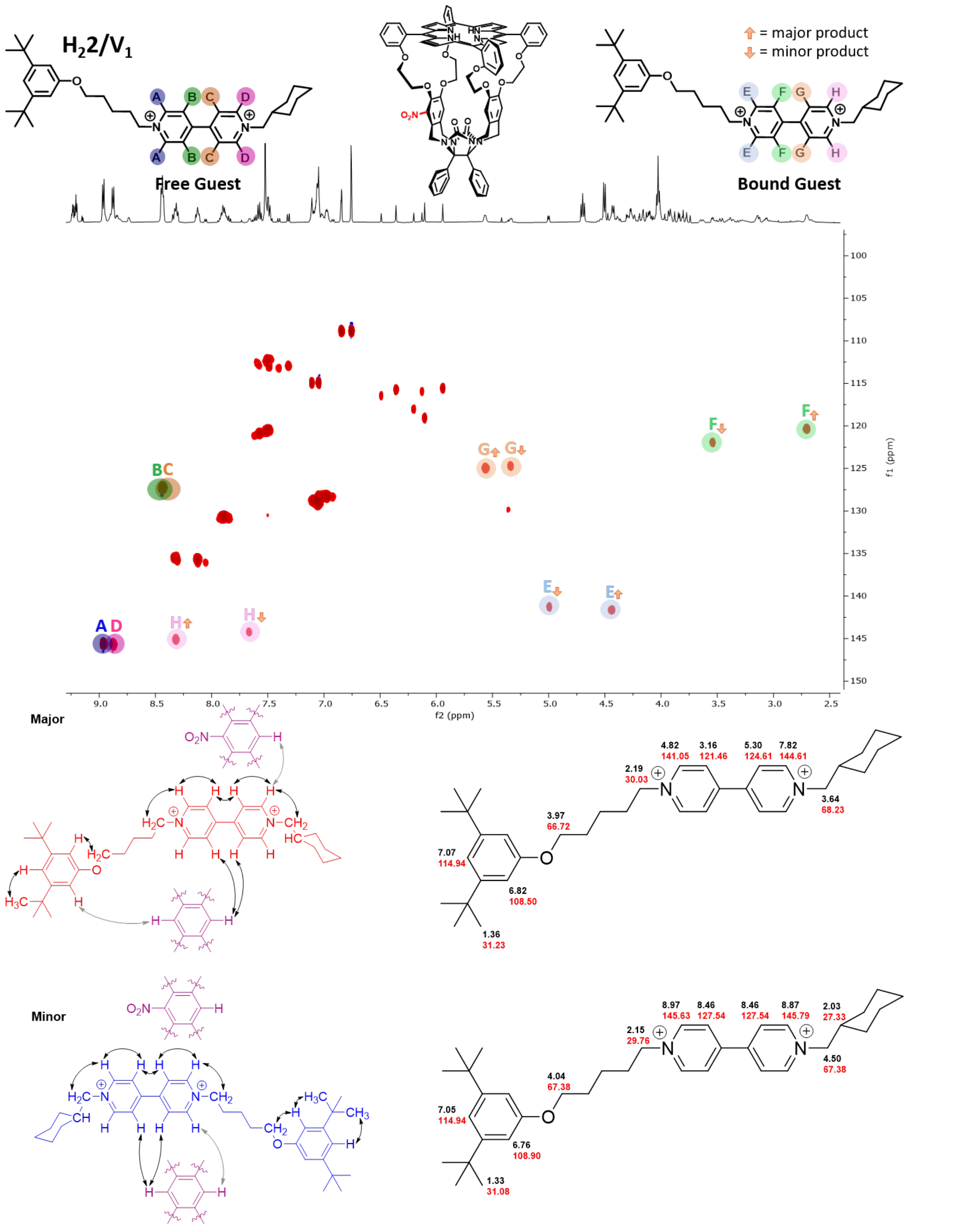

Figure SI95. Top: ^{13}C-^{1}H HSQC of **$H_2$2/V1** (^{1}H: 500 MHz, ^{13}C: 125 MHz, $CDCl_3$:CD_3CN, 1:1, v/v, host : guest, 1:3, 2 mM : 6 mM) in which the protons of the free and bound guests are shown in corresponding colors. The shifts of both major and minor abundant complex are indicated as well; bottom: assigned shifts in ppm for the free guest and bound guest with the corresponding NOE interactions that could be measured by 2D ROESY.

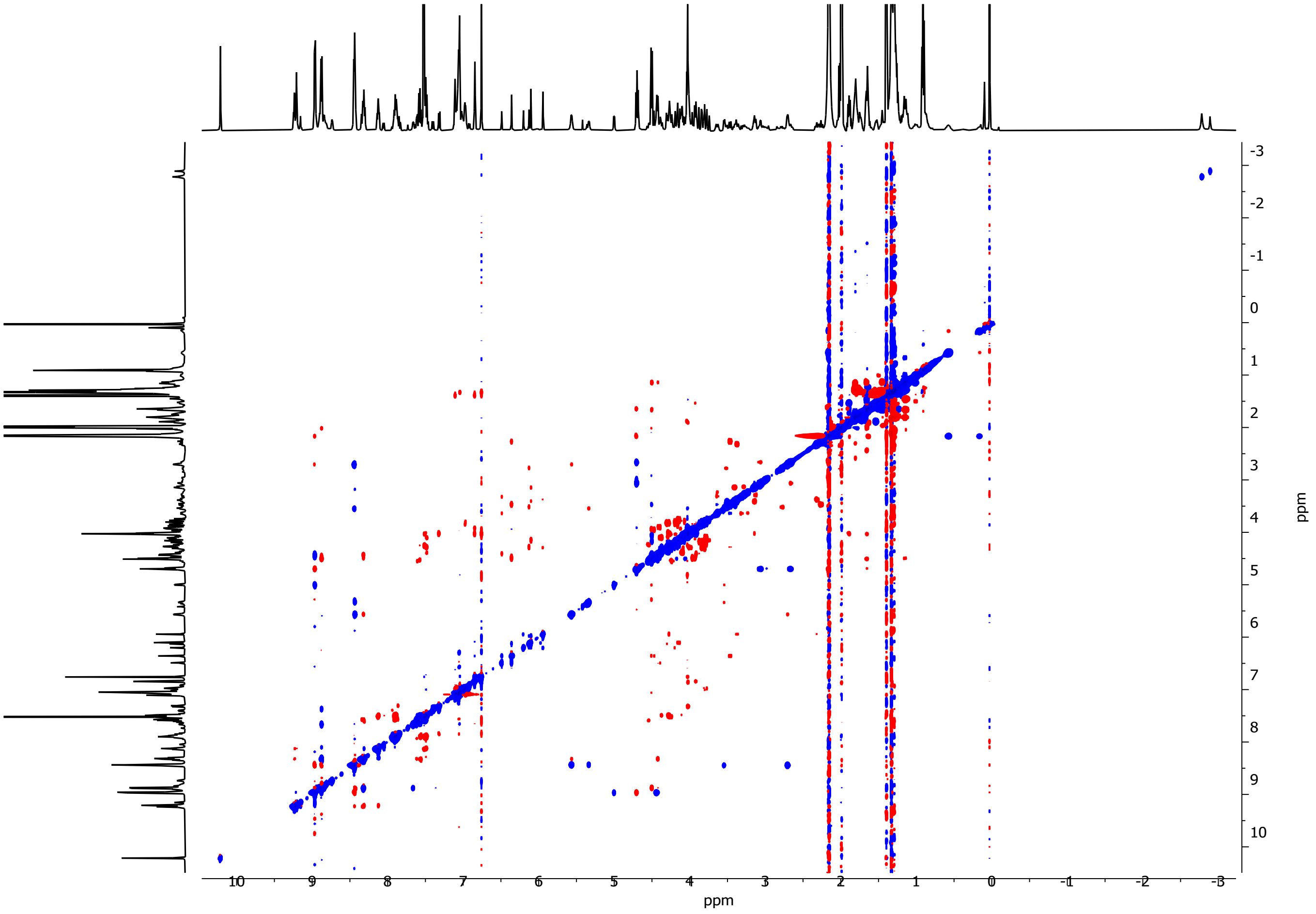

Figure SI96. Full ^{1}H-^{1}H 2D ROESY spectrum of **H₂2/V1** (^{1}H: 500 MHz, $CDCl_3$:CD_3CN, 1:1, v/v, host : guest, 1:3, 2 mM : 6 mM, T = 298 K). Exchange cross peaks generally have the same color as the diagonal, which is in this case blue.

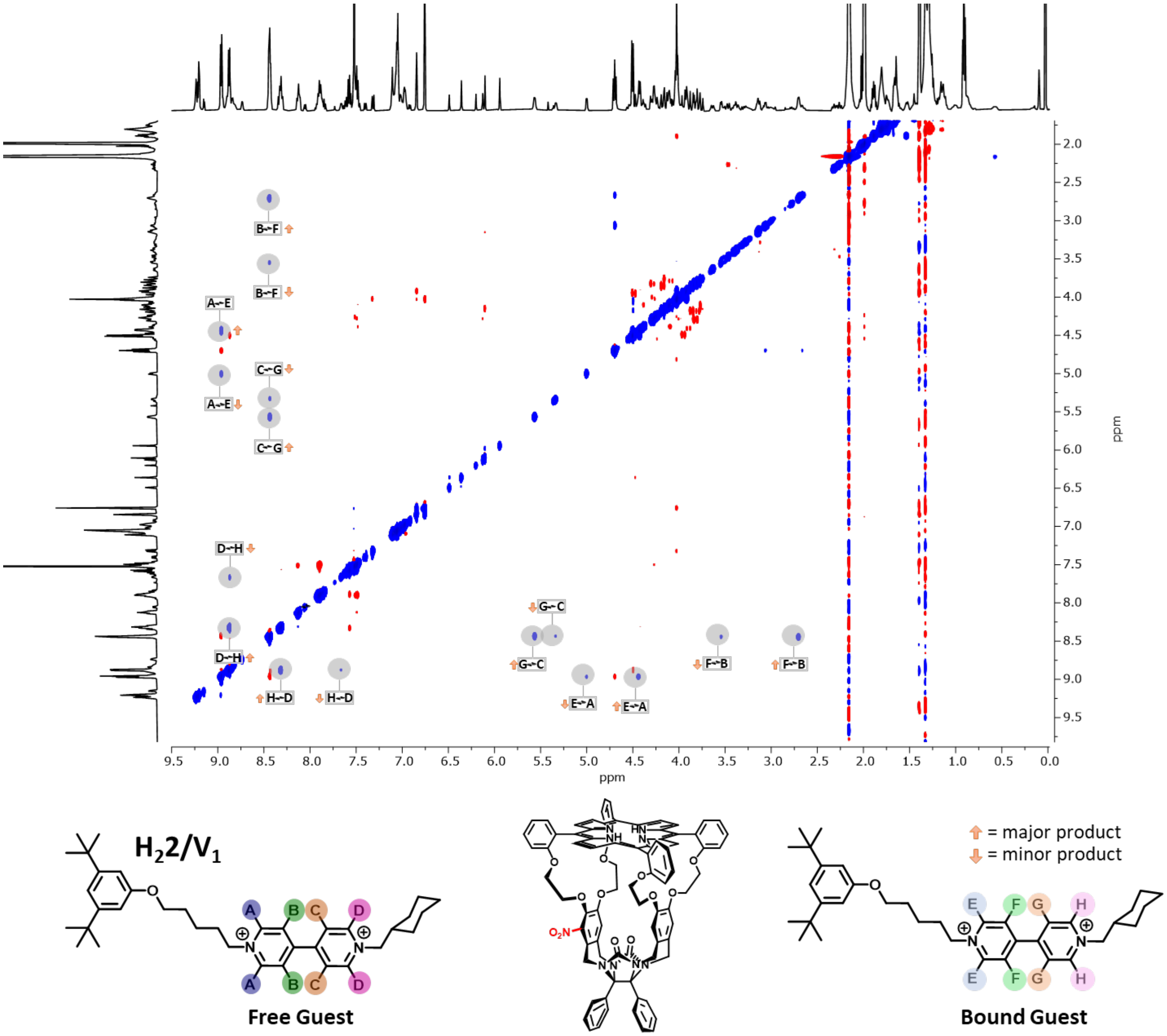

Figure SI97. Zoomed region of ^{1}H-^{1}H 2D ROESY spectrum of **$H_2$2/V1** (^{1}H: 500 MHz, $CDCl_3$:CD_3CN, 1:1, v/v, host : guest, 1:3, 2 mM : 6 mM, T = 298 K) showing relevant guest exchange interactions indicated with A-H. ^{1}H-^{1}H 2D ROESY spectrum. Exchange cross peaks generally have the same color as the diagonal, which is in this case blue.

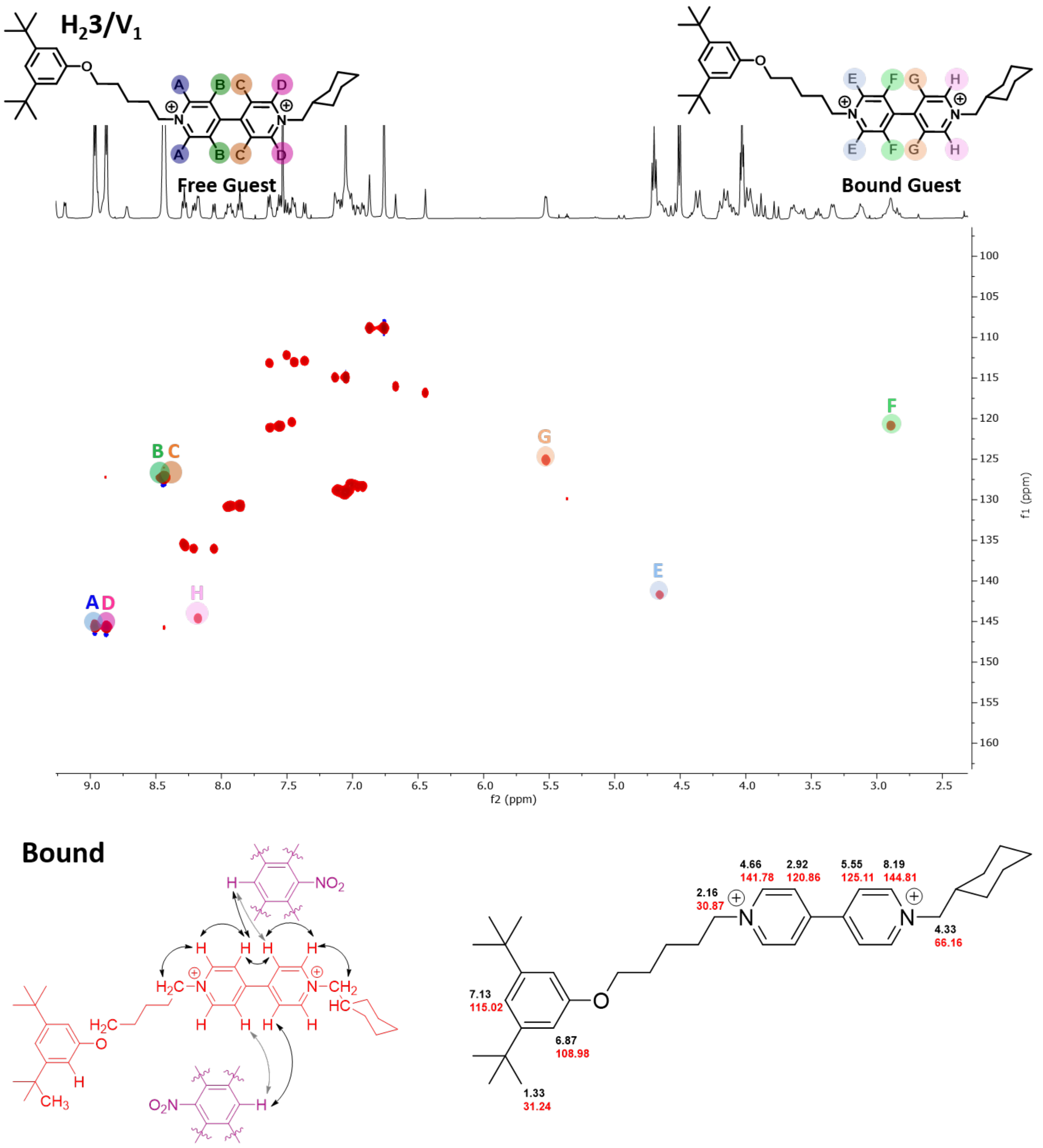

Figure SI98. Top: ^{13}C-^{1}H HSQC of **H$_2$3/V1** (^{1}H: 500 MHz, ^{13}C: 125 MHz, $CDCl_3$:CD_3CN, 1:1, v/v, host : guest, 1:3, 2 mM : 6 mM) in which the protons of the free and bound guests are shown in corresponding colors; bottom: assigned shifts in ppm for the free guest and bound guests with the corresponding NOE interactions that could be measured by 2D ROESY.

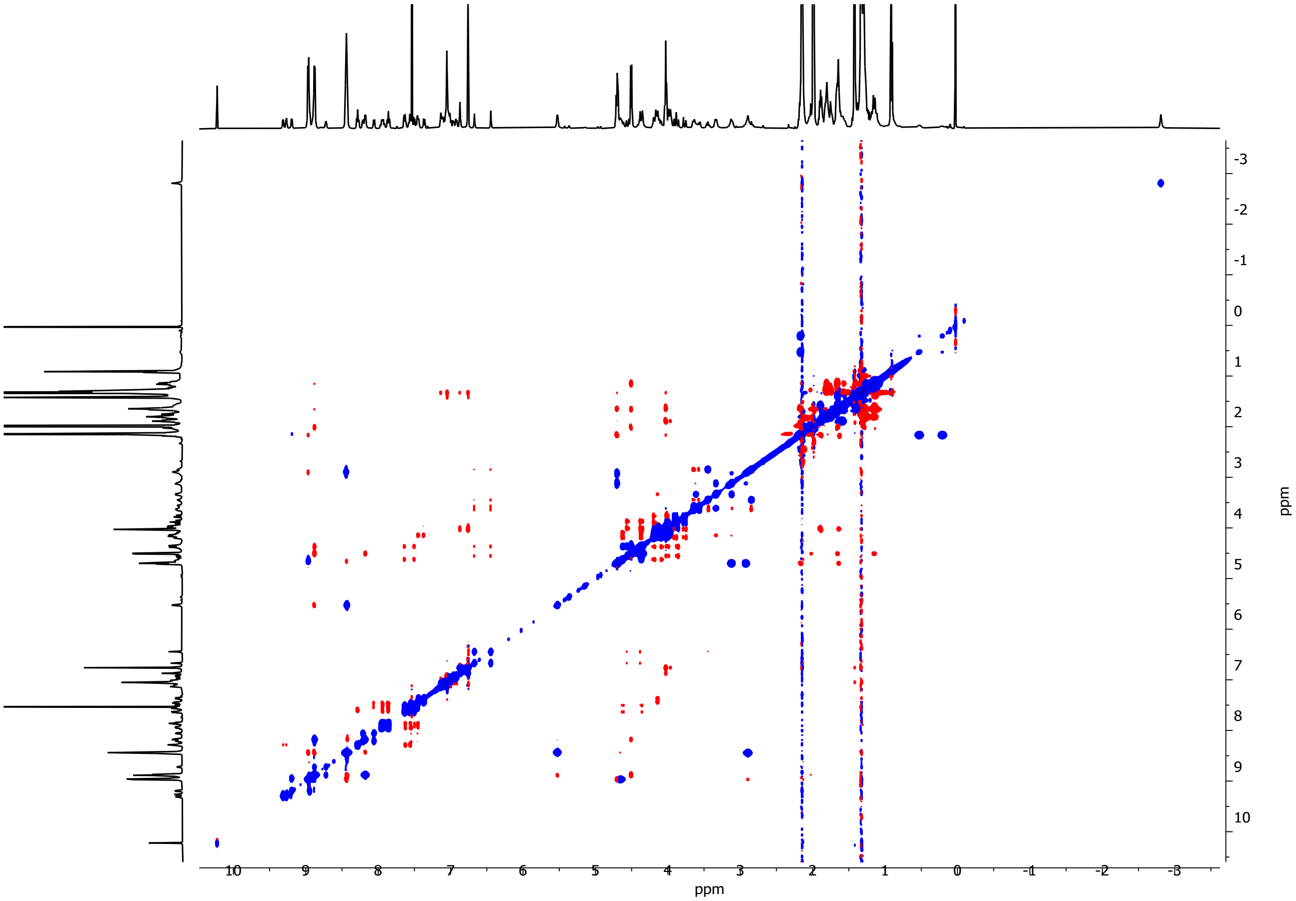

Figure SI99. Full ^{1}H-^{1}H 2D ROESY spectrum of **$H_2$3/V1** (^{1}H: 500 MHz, $CDCl_3$:CD_3CN, 1:1, v/v, host : guest, 1:3, 2 mM : 6 mM, T = 298 K). Exchange cross peaks generally have the same color as the diagonal, which is in this case blue.

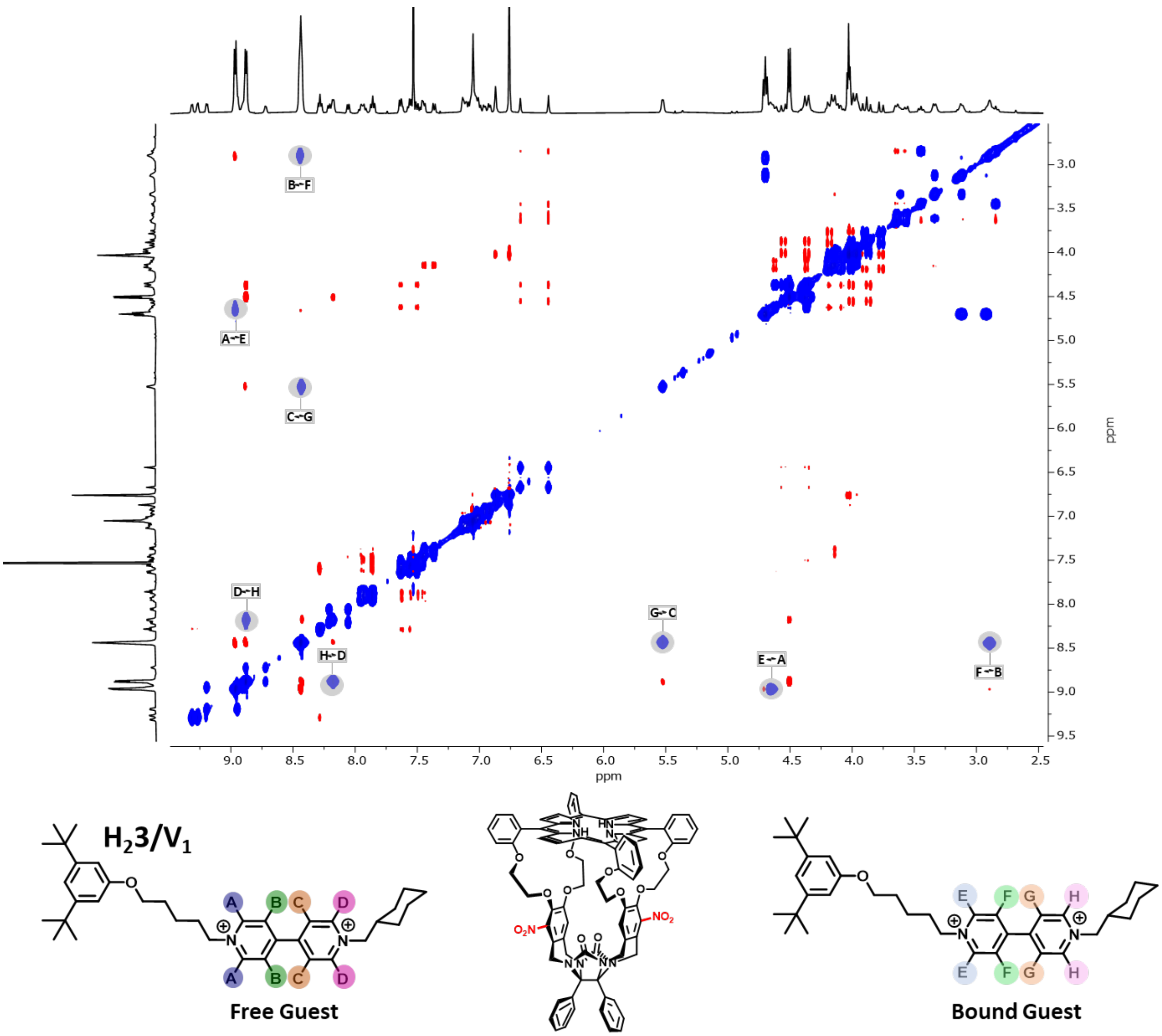

Figure SI100. Zoomed region of ^1H-^1H 2D ROESY spectrum of **$H_2$3/V1** (^1H: 500 MHz, $CDCl_3$:CD_3CN, 1:1, v/v, host : guest, 1:3, 2 mM : 6 mM, T = 298 K) showing relevant guest exchange interactions indicated with A-H. ^1H-^1H 2D ROESY spectrum. Exchange cross peaks generally have the same color as the diagonal, which is in this case blue.

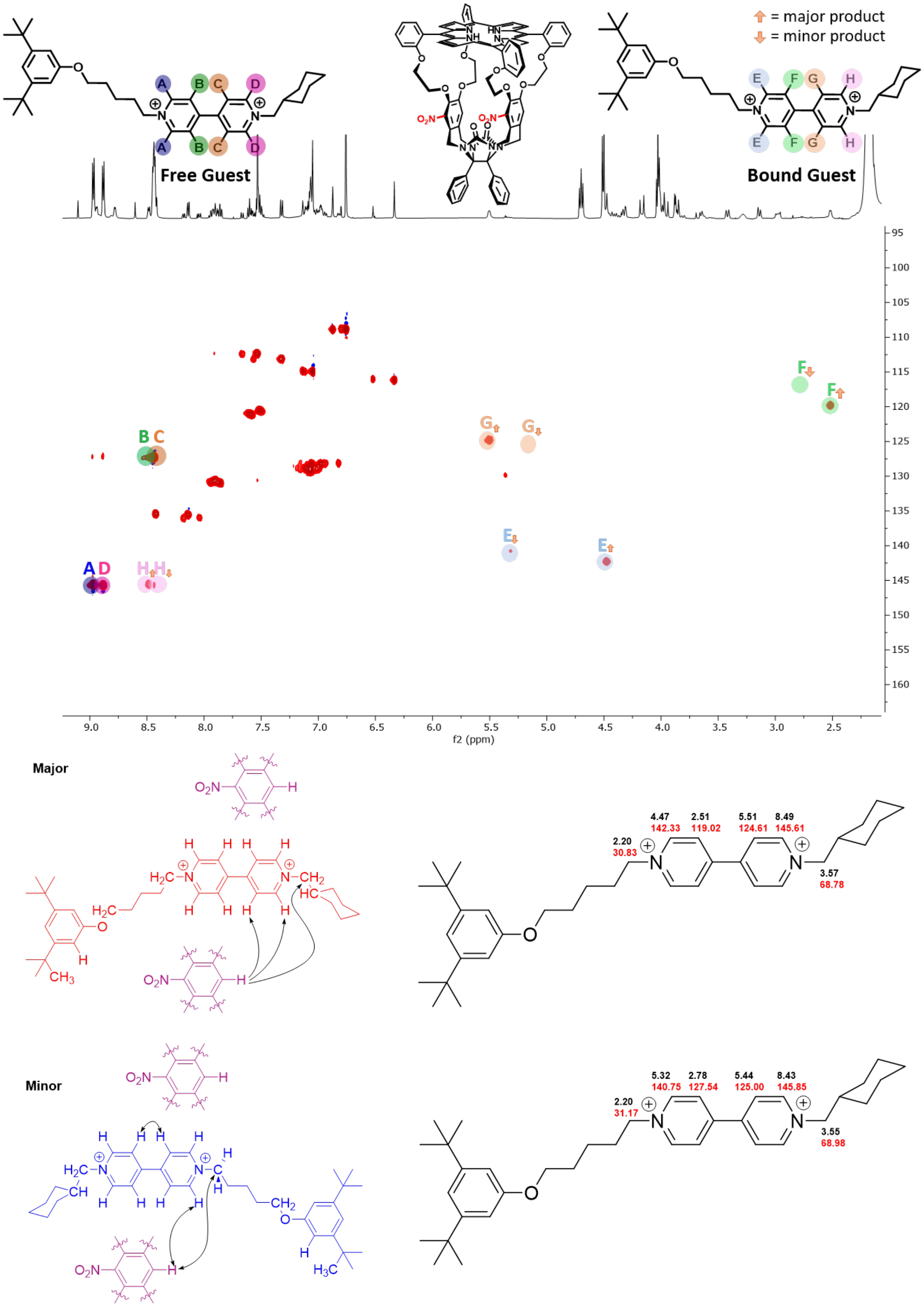

Figure SI101. Top: ^{13}C-^{1}H HSQC of **H₂4/V1** (^{1}H: 500 MHz, ^{13}C: 125 MHz, $CDCl_3$:CD_3CN, 1:1, v/v, host : guest, 1:3, 2 mM : 6 mM) in which the protons of the free and bound guests are shown in corresponding colors. The shifts of both major and minor species are indicated as well; bottom: assigned shifts in ppm for the free and bound guests with the corresponding ROE interactions that could be measured by 2D ROESY.

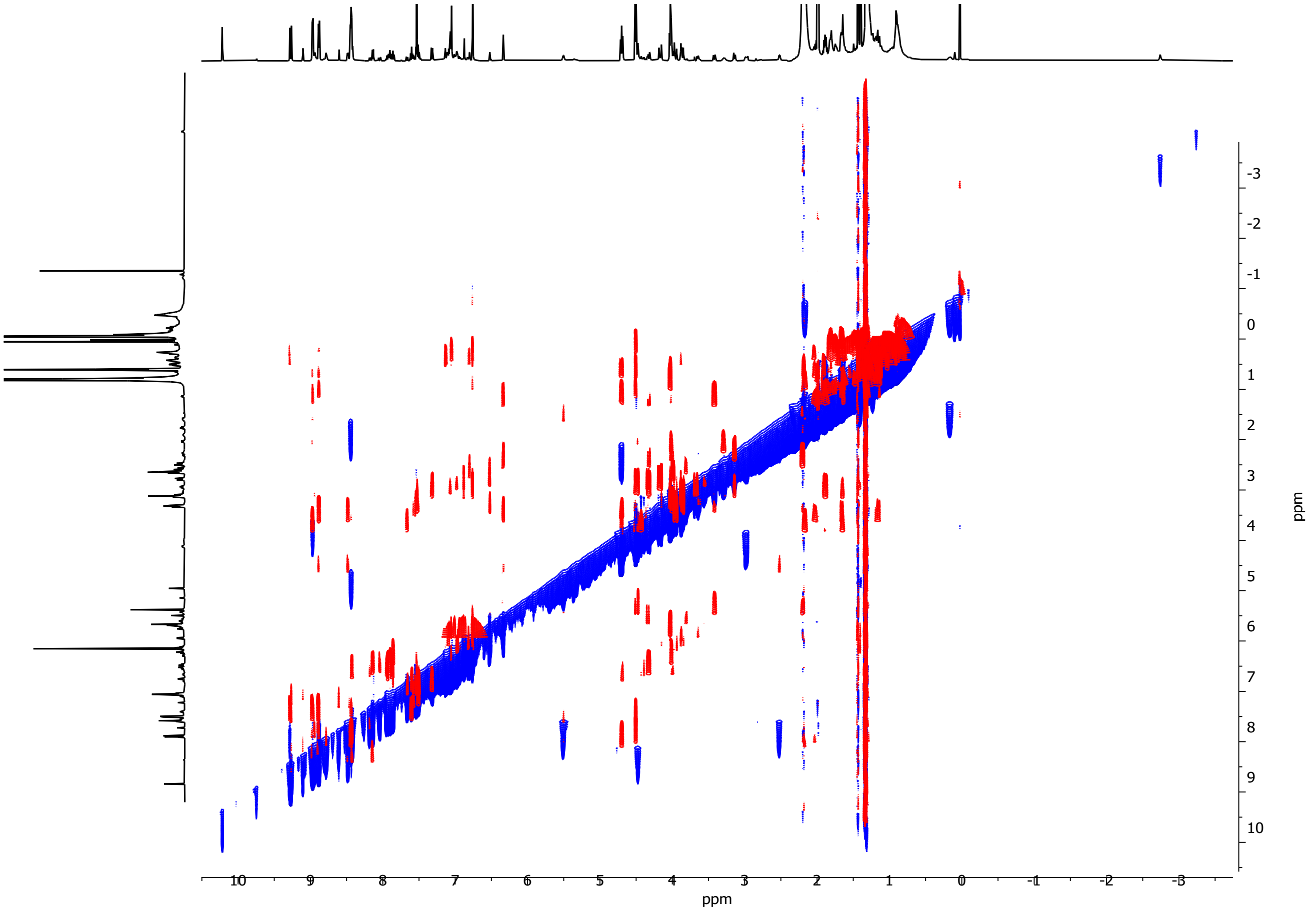

Figure SI102. Full ^{1}H-^{1}H 2D ROESY spectrum of **$H_2$4/V1** (^{1}H: 500 MHz, $CDCl_3$:CD_3CN, 1:1, v/v, host : guest, 1:3, 2 mM : 6 mM, T = 298 K). Exchange cross peaks generally have the same color as the diagonal, which is in this case blue.

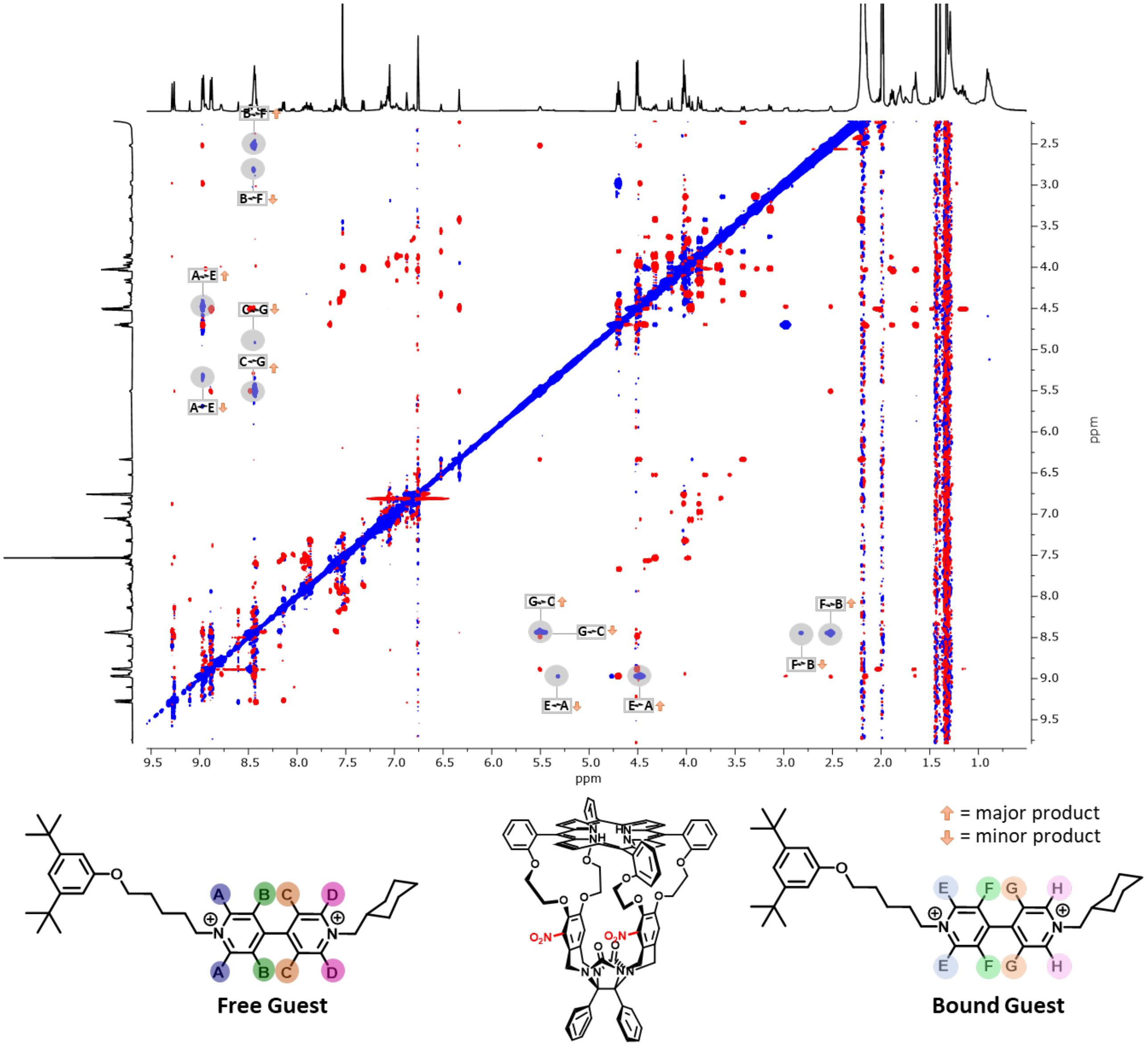

Figure SI103. Zoomed region of ^1H-^1H 2D ROESY spectrum of **$H_2$4/V1** (^1H: 500 MHz, $CDCl_3$:CD_3CN, 1:1, v/v, host : guest, 1:3, 2 mM : 6 mM, T = 298 K) showing relevant guest exchange interactions indicated with A-H. ^1H-^1H 2D ROESY spectrum. Exchange cross peaks generally have the same color as the diagonal, which is in this case blue.

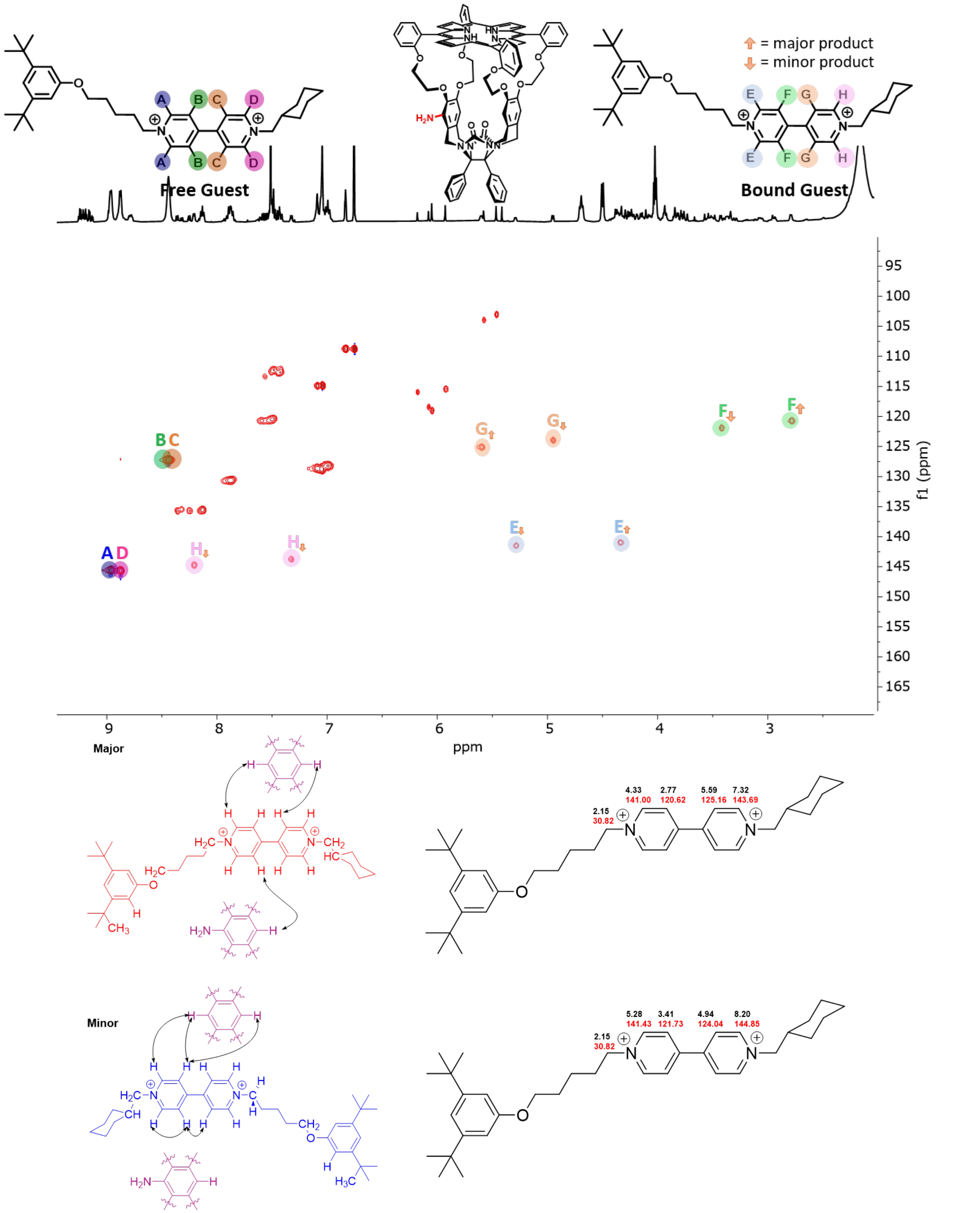

Figure SI104. Top: ^{13}C-^{1}H HSQC of **H_25/V1** (^{1}H: 500 MHz, ^{13}C: 125 MHz, $CDCl_3$:CD_3CN, 1:1, v/v, host : guest, 1:3, 2 mM : 6 mM) in which the protons of the free and bound guests are shown in corresponding colors. The shifts of both major and minor abundant complexes are indicated as well; bottom: assigned shifts in ppm for the free guest and bound guests with the corresponding ROE interactions that could be measured by 2D ROESY.

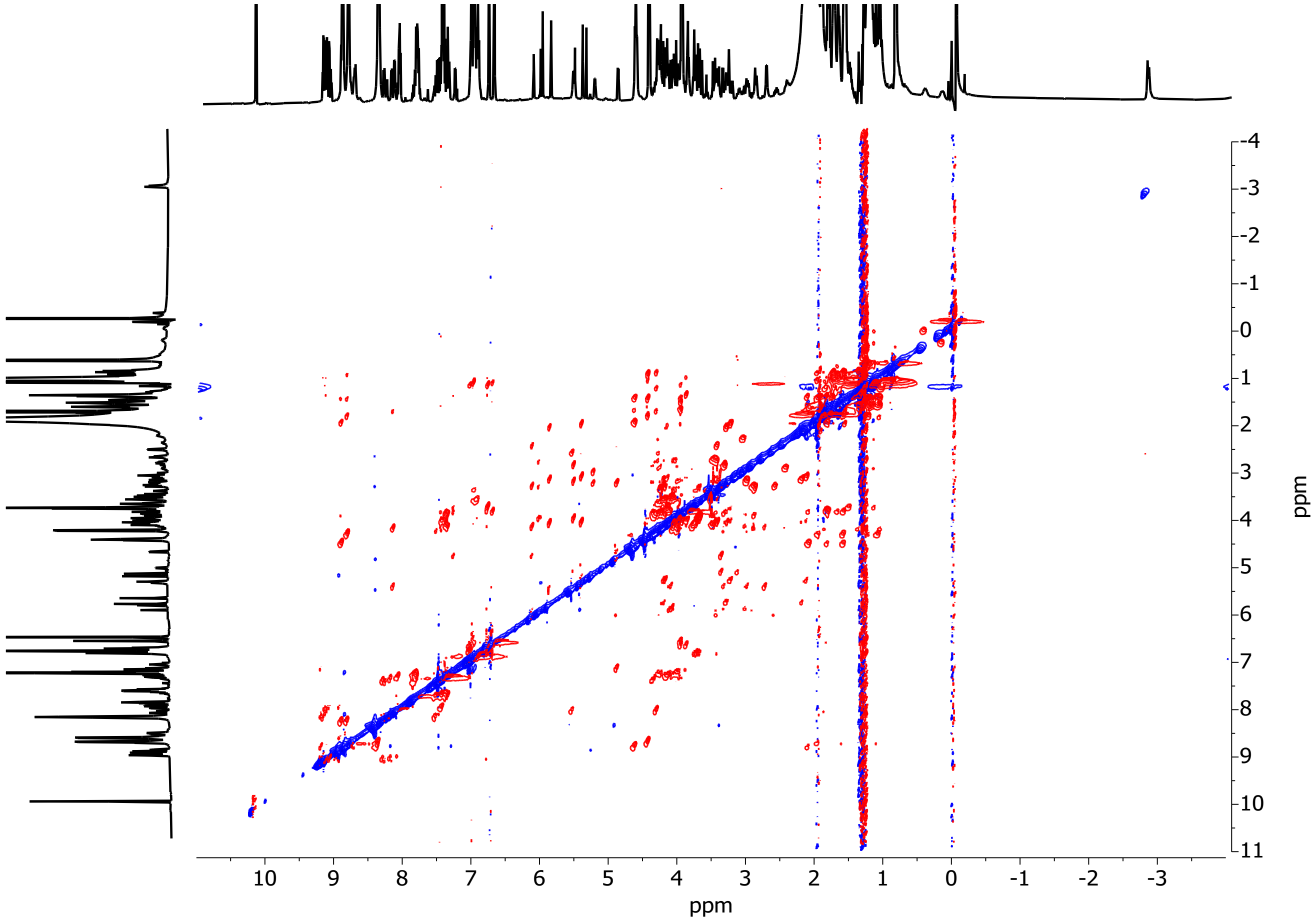

Figure SI105. Full ^{1}H-^{1}H 2D ROESY spectrum of **H_25/V1** (^{1}H: 500 MHz, $CDCl_3$:CD_3CN, 1:1, v/v, host : guest, 1:3, 2 mM : 6 mM, T = 298 K). Exchange cross peaks generally have the same color as the diagonal, which is in this case blue.

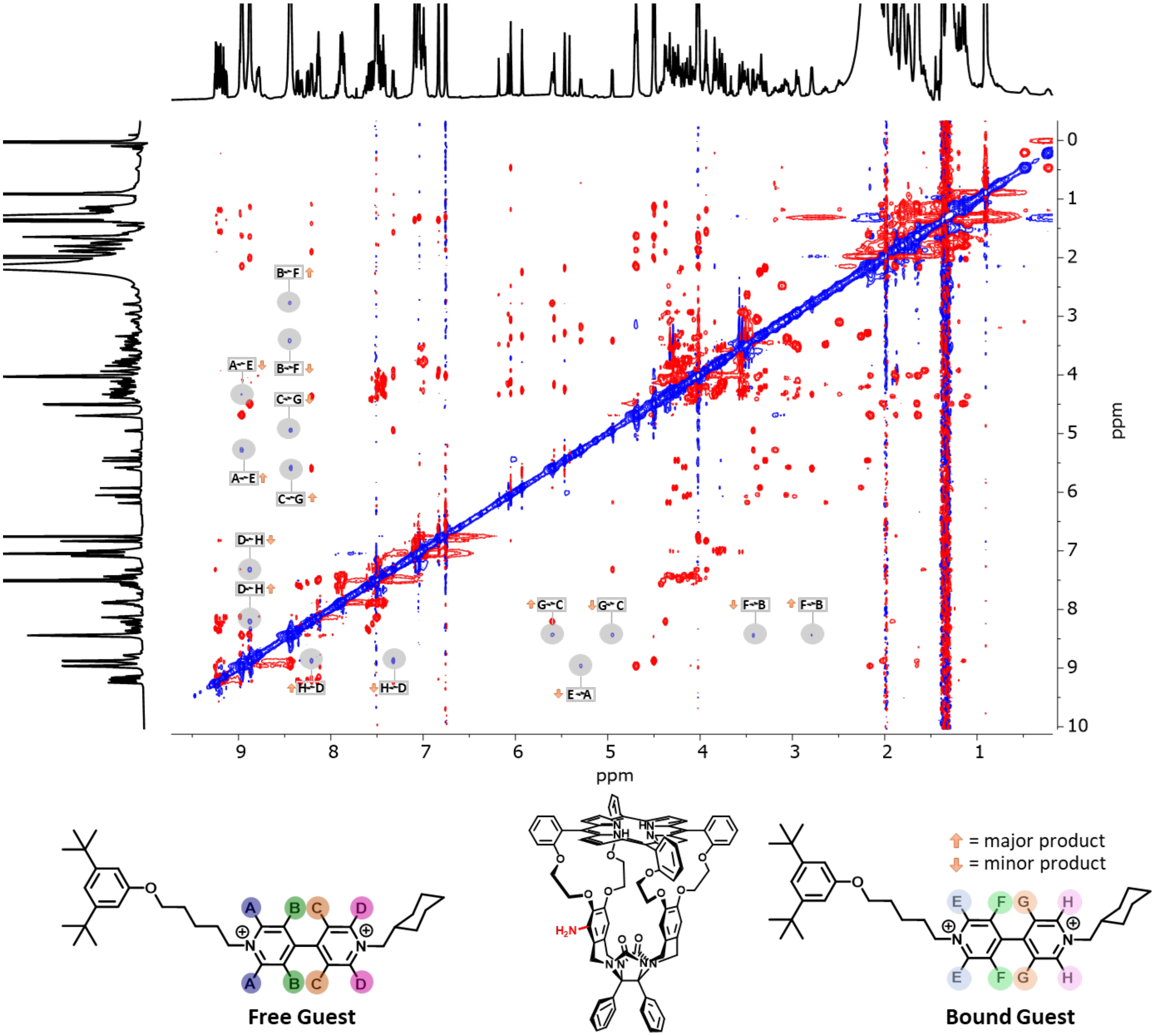

Figure SI106. Zoomed region of ^{1}H-^{1}H 2D ROESY spectrum of **H$_2$5/V1** (^{1}H: 500 MHz, $CDCl_3$:CD_3CN, 1:1, v/v, host : guest, 1:3, 2 mM : 6 mM, T = 298 K) showing relevant guest exchange interactions indicated with A-H. ^{1}H-^{1}H 2D ROESY spectrum. Exchange cross peaks generally have the same color as the diagonal, which is in this case blue.

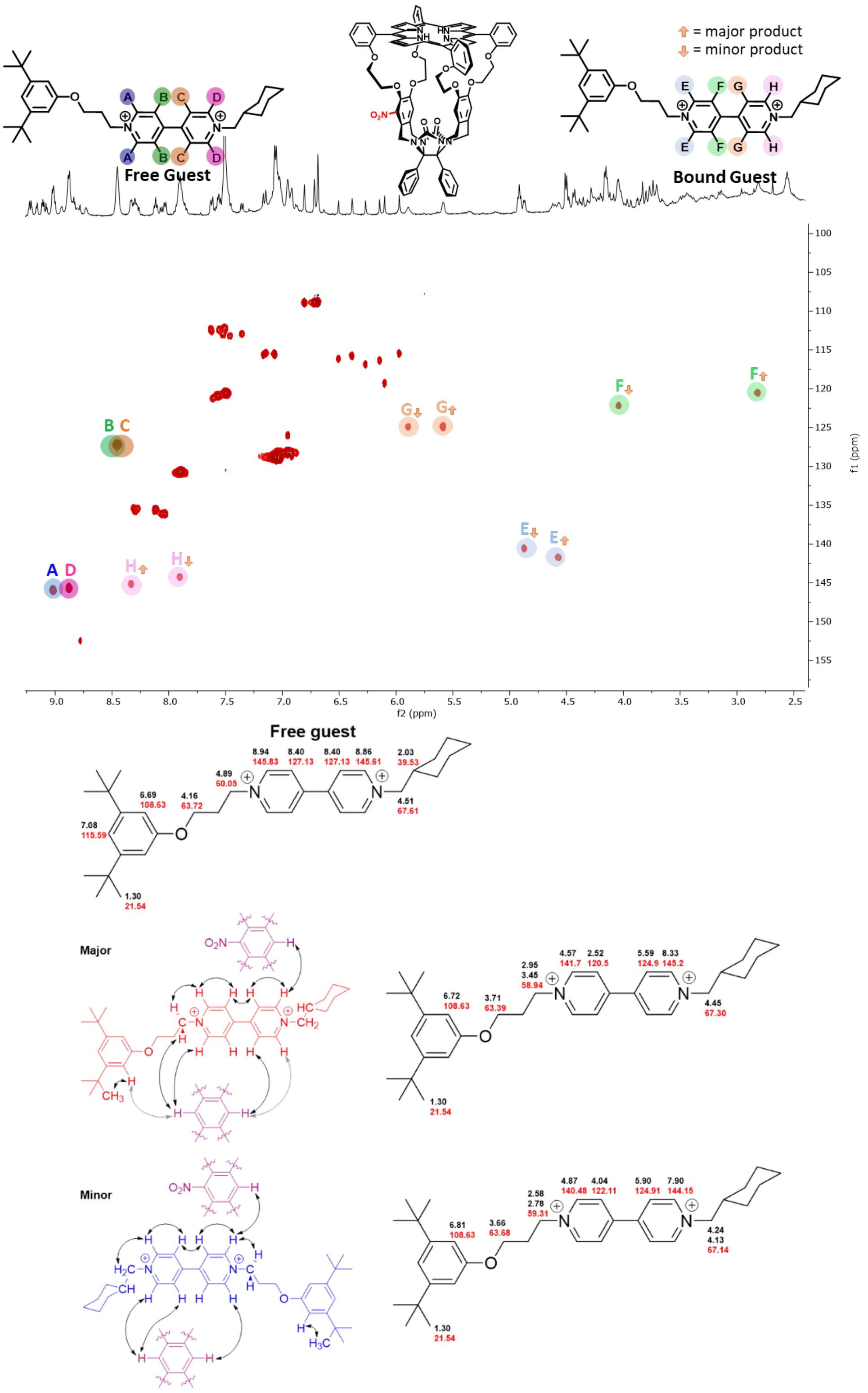

Figure SI107. Top: ^{13}C-^{1}H HSQC of **$H_2$2/V2** (^{1}H: 500 MHz, ^{13}C: 125 MHz, $CDCl_3$:CD_3CN, 1:1, v/v, host : guest, 1:3, 2 mM : 6 mM) in which the protons of the free and bound guests are shown in corresponding colors. The shifts of both major and minor abundant complexes are indicated as well; bottom: assigned shifts in ppm for the free and bound guests with the corresponding ROE interactions that could be measured by 2D ROESY.

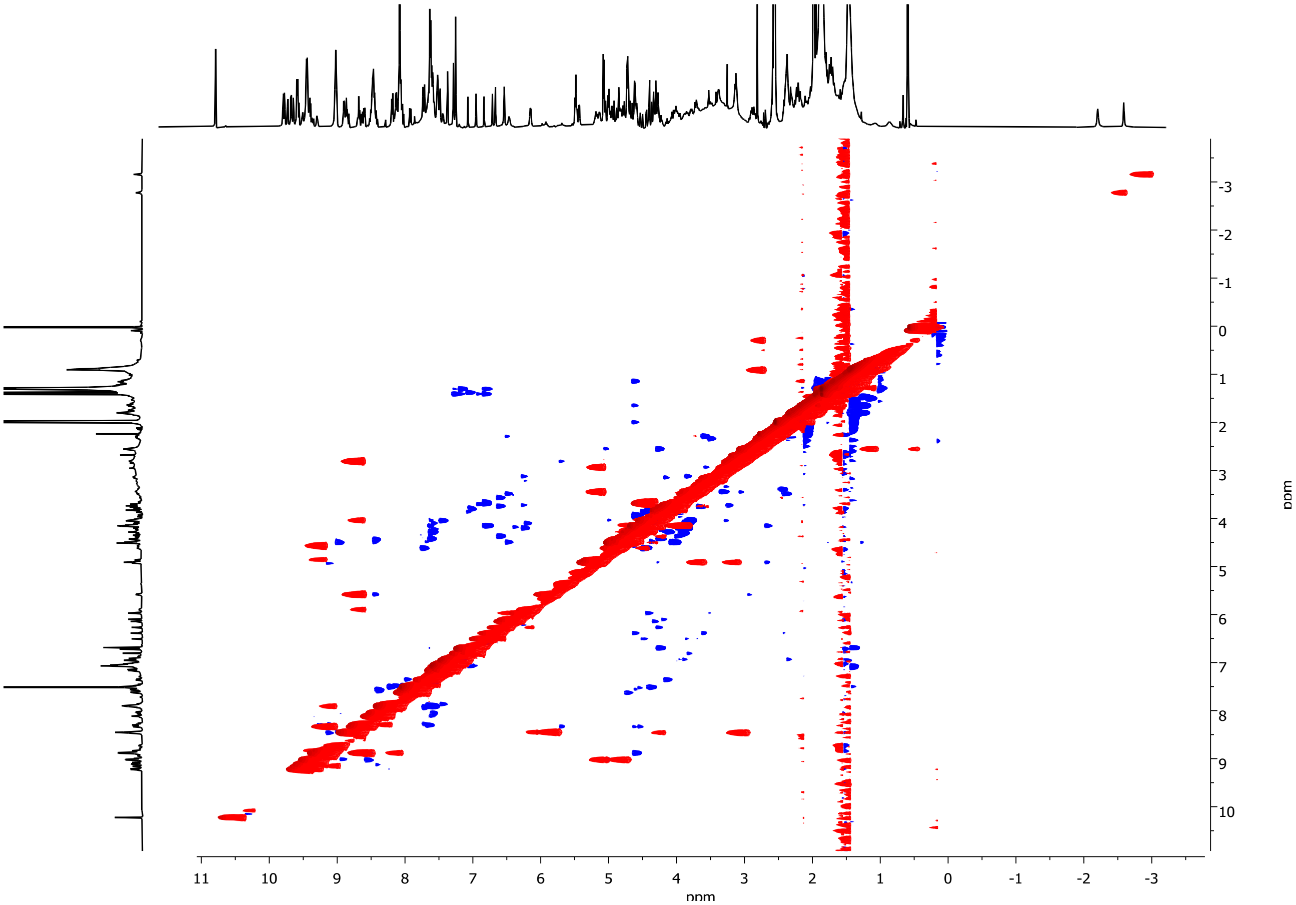

Figure SI108. Full ^{1}H-^{1}H 2D ROESY spectrum of **$H_2$2/V2** (^{1}H: 500 MHz, $CDCl_3$:CD_3CN, 1:1, v/v, host : guest, 1:3, 2 mM : 6 mM, T = 298 K). Exchange cross peaks generally have the same color as the diagonal, which is in this case blue.

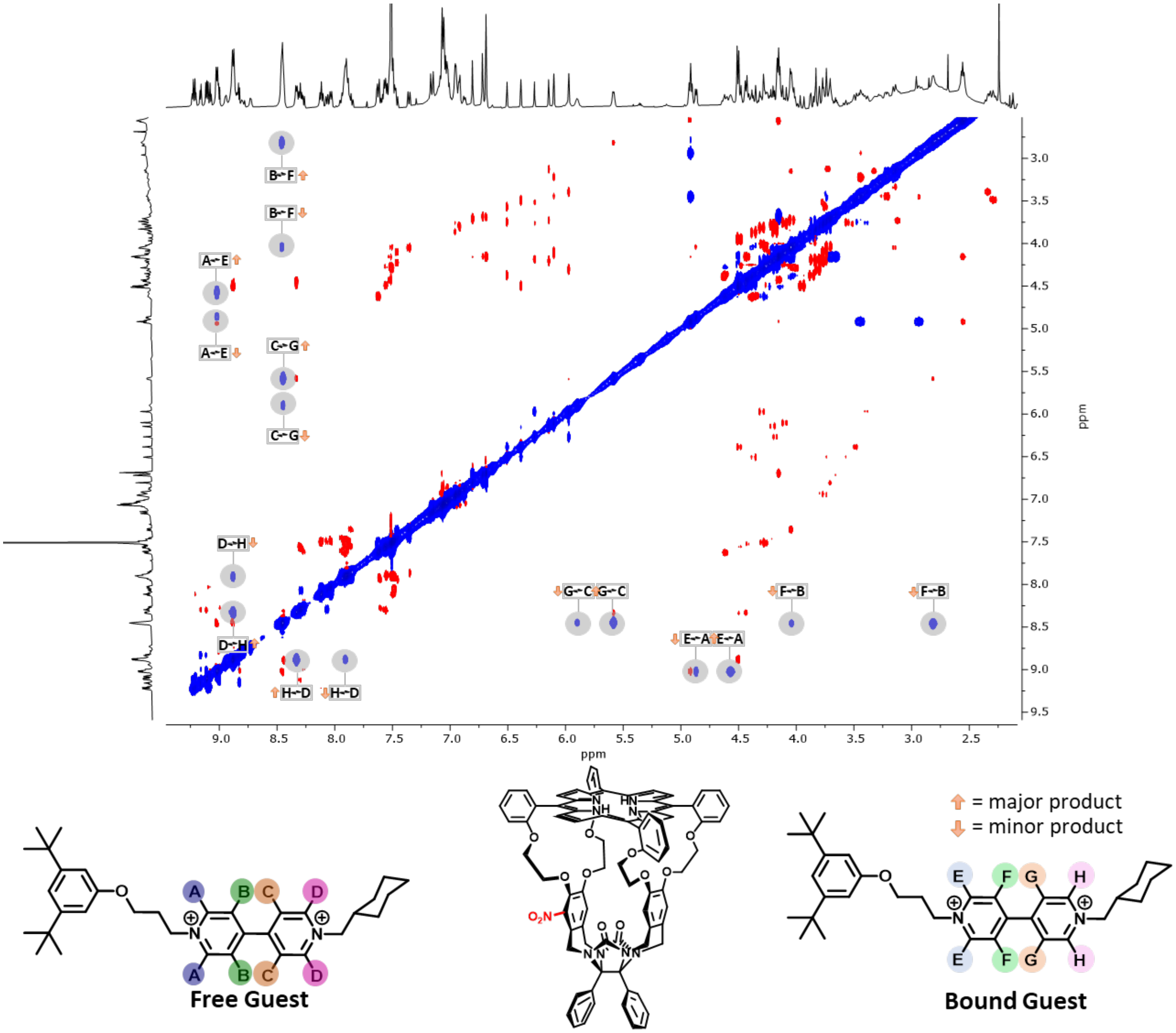

Figure SI109. Zoomed region of ^{1}H-^{1}H 2D ROESY spectrum of **$H_2$2/V2** (^{1}H: 500 MHz, $CDCl_3$:CD_3CN, 1:1, v/v, host : guest, 1:3, 2 mM : 6 mM, T = 298 K) showing relevant guest exchange interactions indicated with A – H. ^{1}H-^{1}H 2D ROESY spectrum. Exchange cross peaks generally have the same color as the diagonal, which is in this case blue.

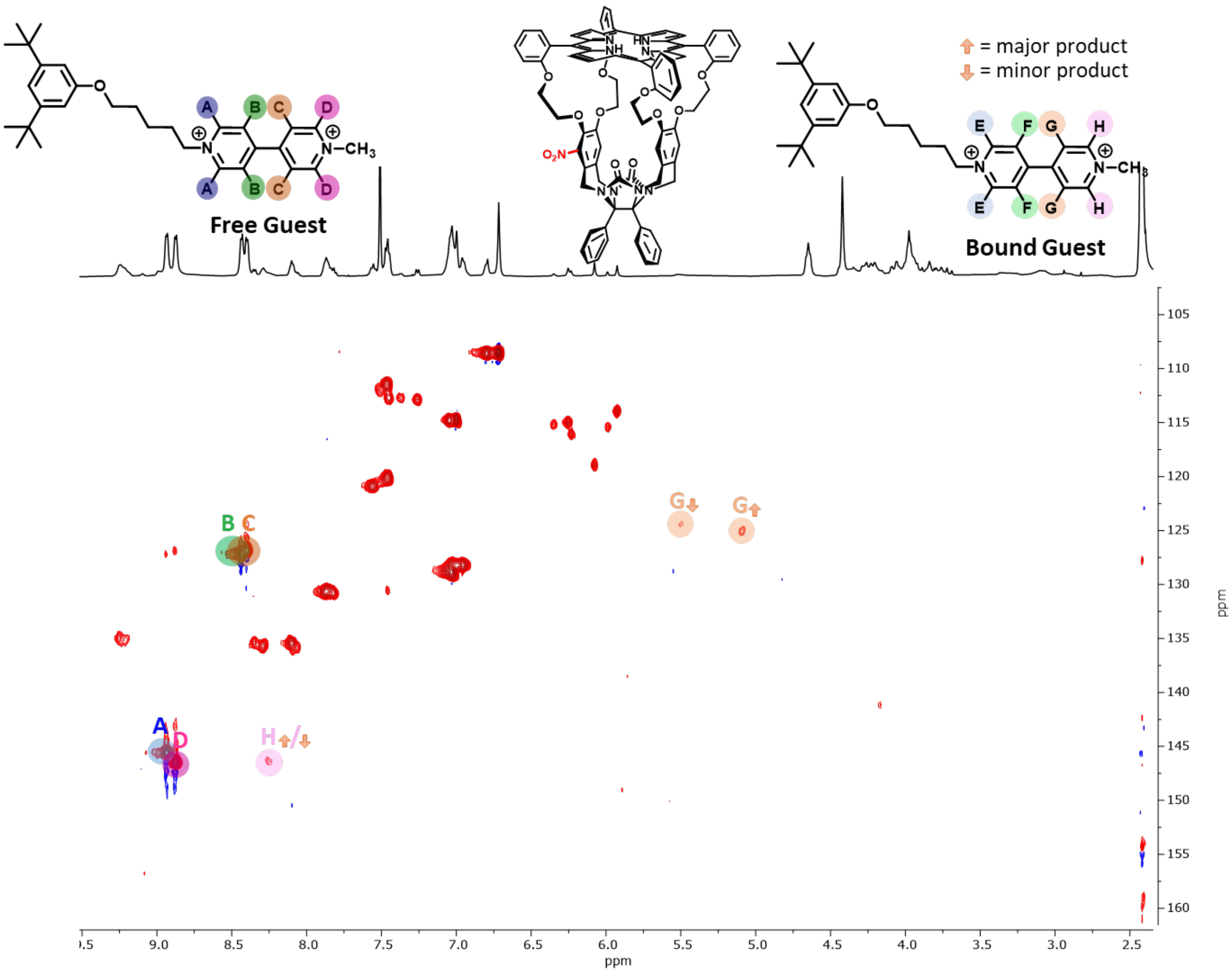

Figure SI110. Top: ^{13}C-^{1}H HSQC of **$H_2$2/V3** (^{1}H: 500 MHz, ^{13}C: 125 MHz, $CDCl_3$:CD_3CN, 1:1, v/v, host : guest, 1:3, 2 mM : 6 mM) in which the protons of the free and bound guests are shown in corresponding colors. The shifts of both major and minor abundant complexes are indicated as well. Due to fast exchange, even at -30 °C limited HSQC cross peaks were visible as well as ROE correlations.

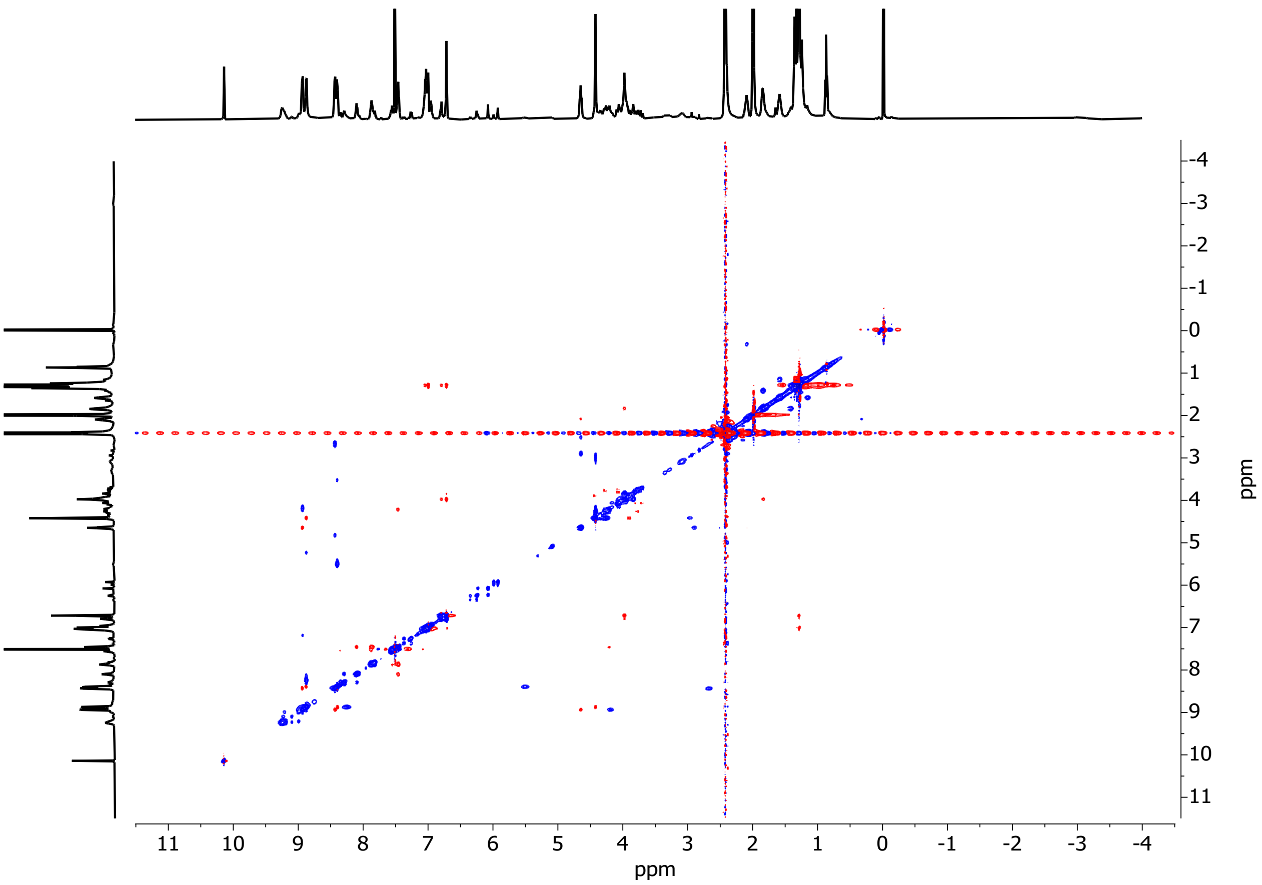

Figure SI111. Full ^{1}H-^{1}H 2D ROESY spectrum of **$H_2$2/V2** (^{1}H: 500 MHz, $CDCl_3$:CD_3CN, 1:1, v/v, host : guest, 1:3, 2 mM : 6 mM, T = 243 K). Exchange cross peaks generally have the same color as the diagonal, which is in this case blue.

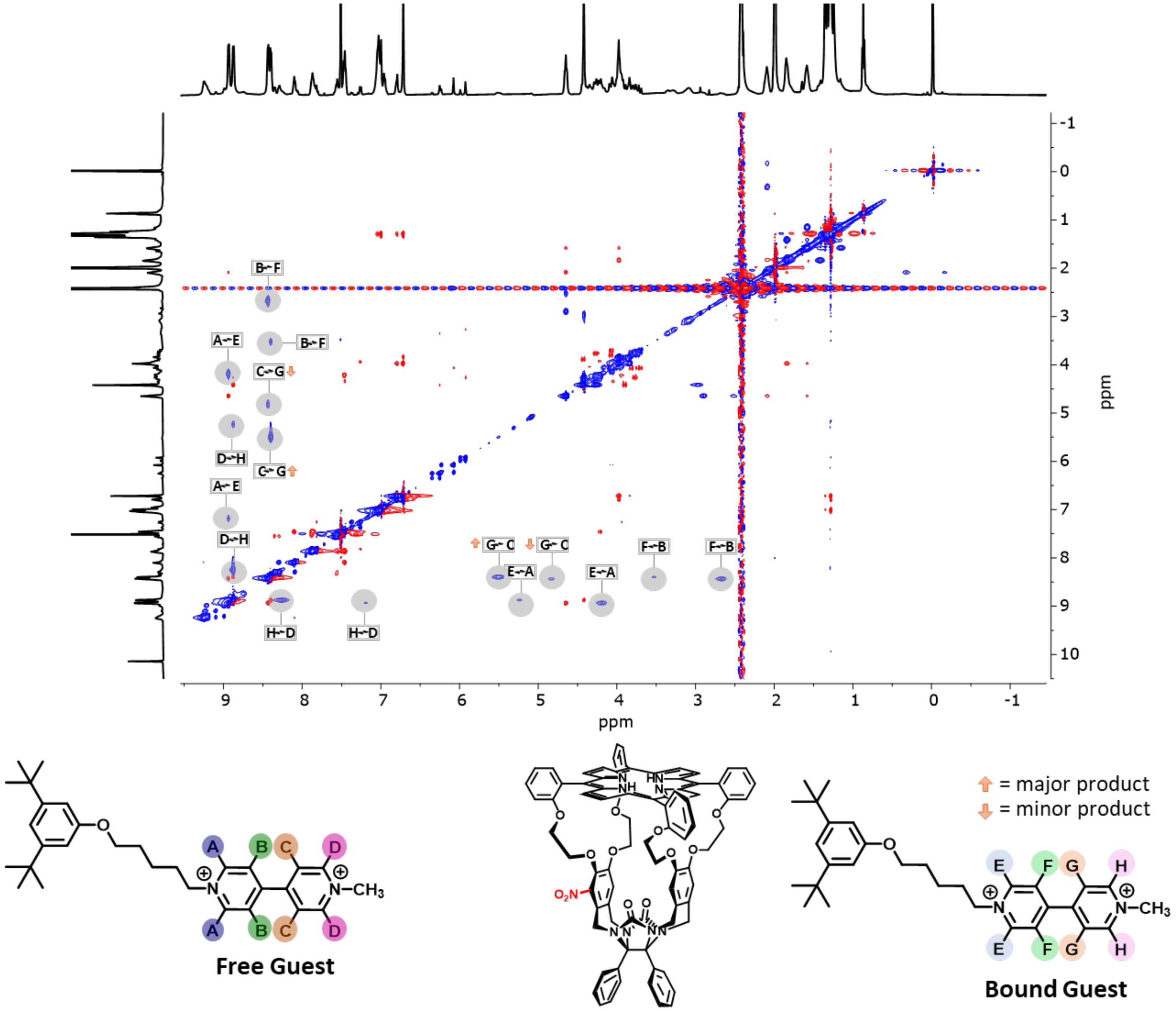

Figure SI112. Zoomed region of ^{1}H-^{1}H 2D ROESY spectrum of **$H_2$2/V3** (^{1}H: 500 MHz, $CDCl_3$:CD_3CN, 1:1, v/v, host : guest, 1:3, 2 mM : 6 mM, T = 243 K) showing relevant guest exchange interactions indicated with A-H. ^{1}H-^{1}H 2D ROESY spectrum. Exchange cross peaks generally have the same color as the diagonal, which is in this case blue.

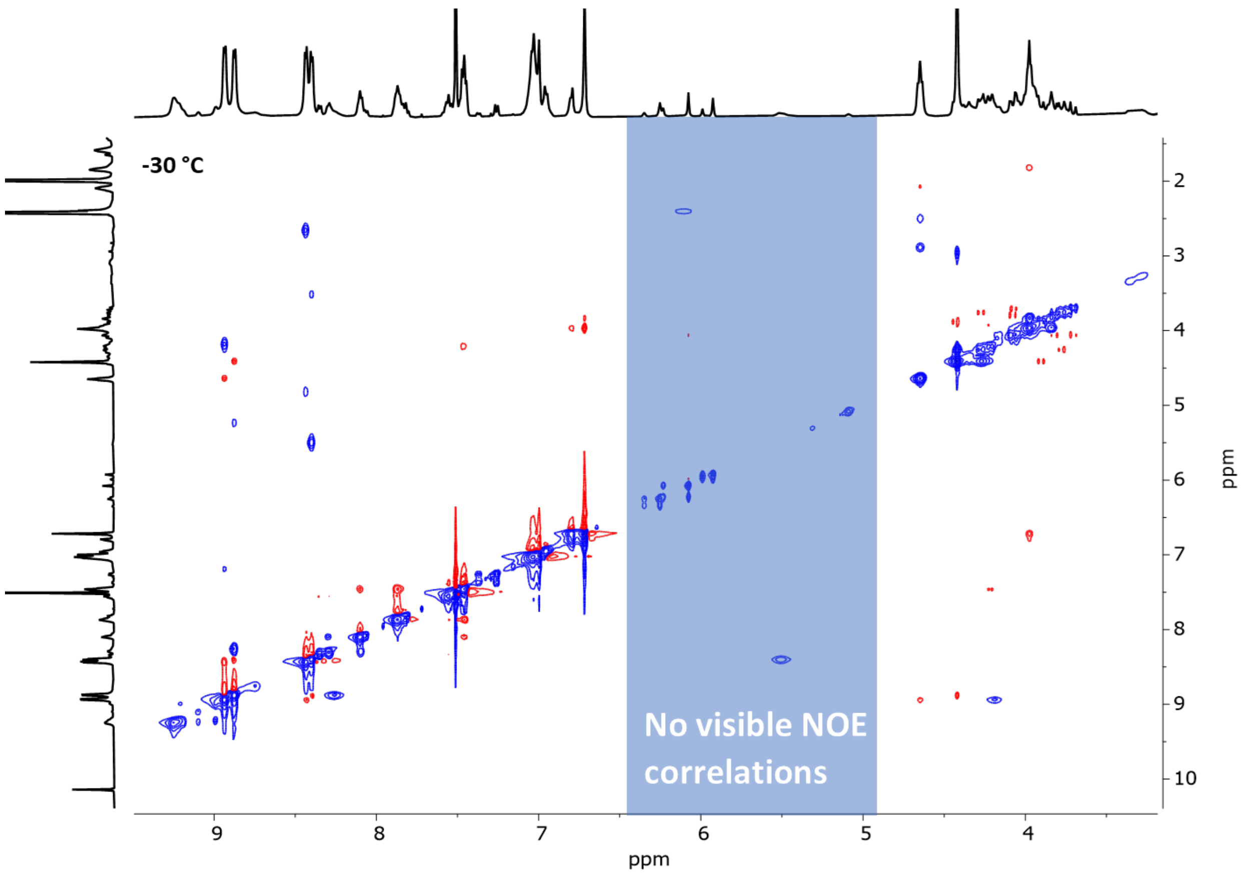

Figure SI113. ^{1}H-^{1}H ROESY of **$H_2$2/V3** (^{1}H: 500 MHz, $CDCl_3$:CD_3CN, 1:1, v/v, host : guest, 1:3, 2 mM : 6 mM, 243 K) in which the region where the prominent ROE correlations for the xylylene sidewalls and the 4,4'-bipyridine moiety should occur is indicated in blue. These peaks are very broad due to fast exchange and therefore show no ROE correlations. The mixture could not be cooled down further, because the region of the melting point of acetonitrile (−45 °C) was reached. Hence, the definitive orientation of the guests in the major and minor abundant complex of **$H_2$2/V3** could not be determined by 2D ROESY.

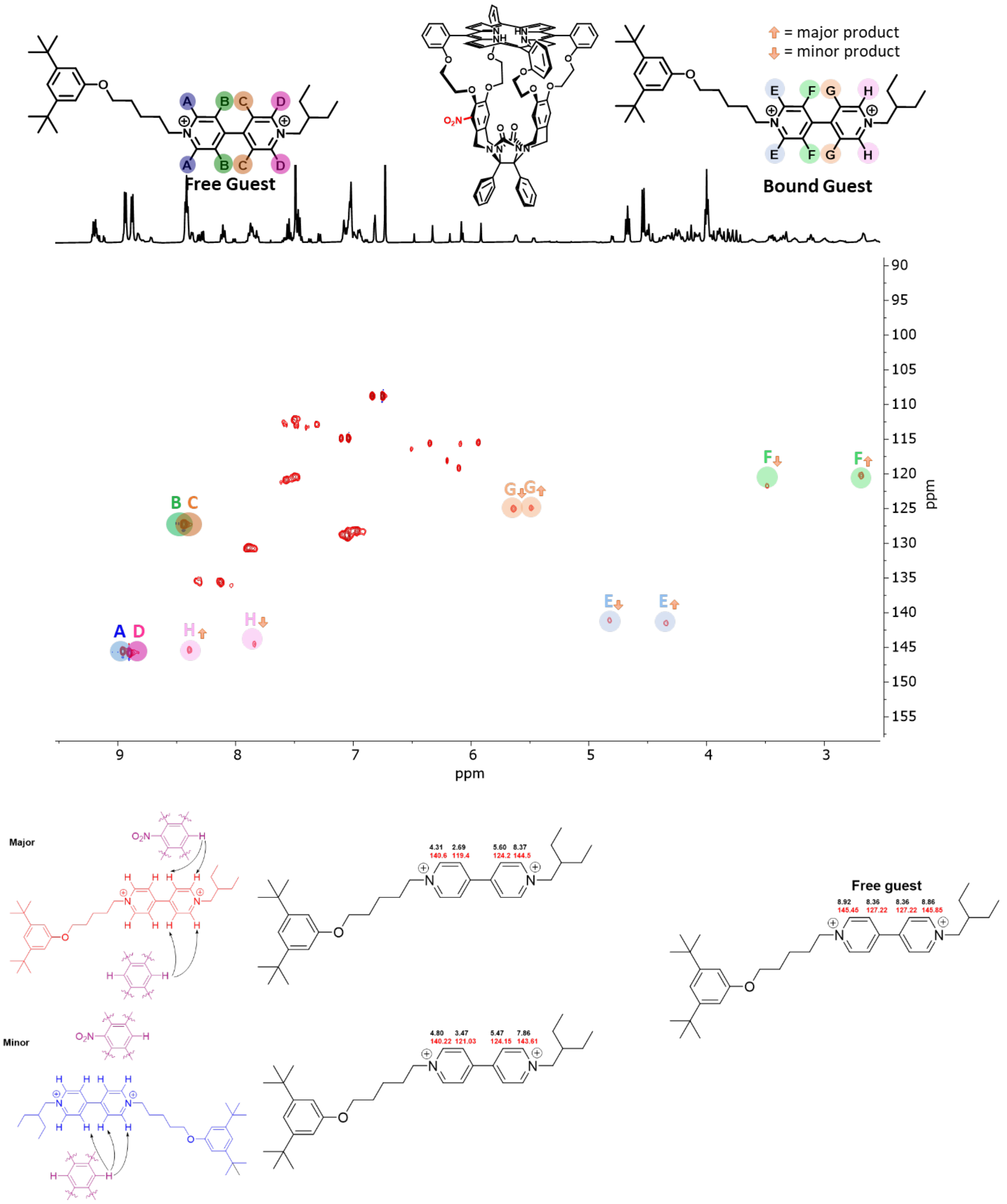

Figure SI114. Top: ^{13}C-^{1}H HSQC of **$H_2$2/V4** (^{1}H: 500 MHz, ^{13}C: 125 MHz, $CDCl_3$:CD_3CN, 1:1, v/v, host : guest, 1:3, 2 mM : 6 mM) in which the protons of the free and bound guests are shown in corresponding colors. The shifts of both major and minor abundant complexes are indicated as well; bottom: assigned shifts in ppm for the free and bound guests with the corresponding ROE interactions that could be measured by 2D ROESY.

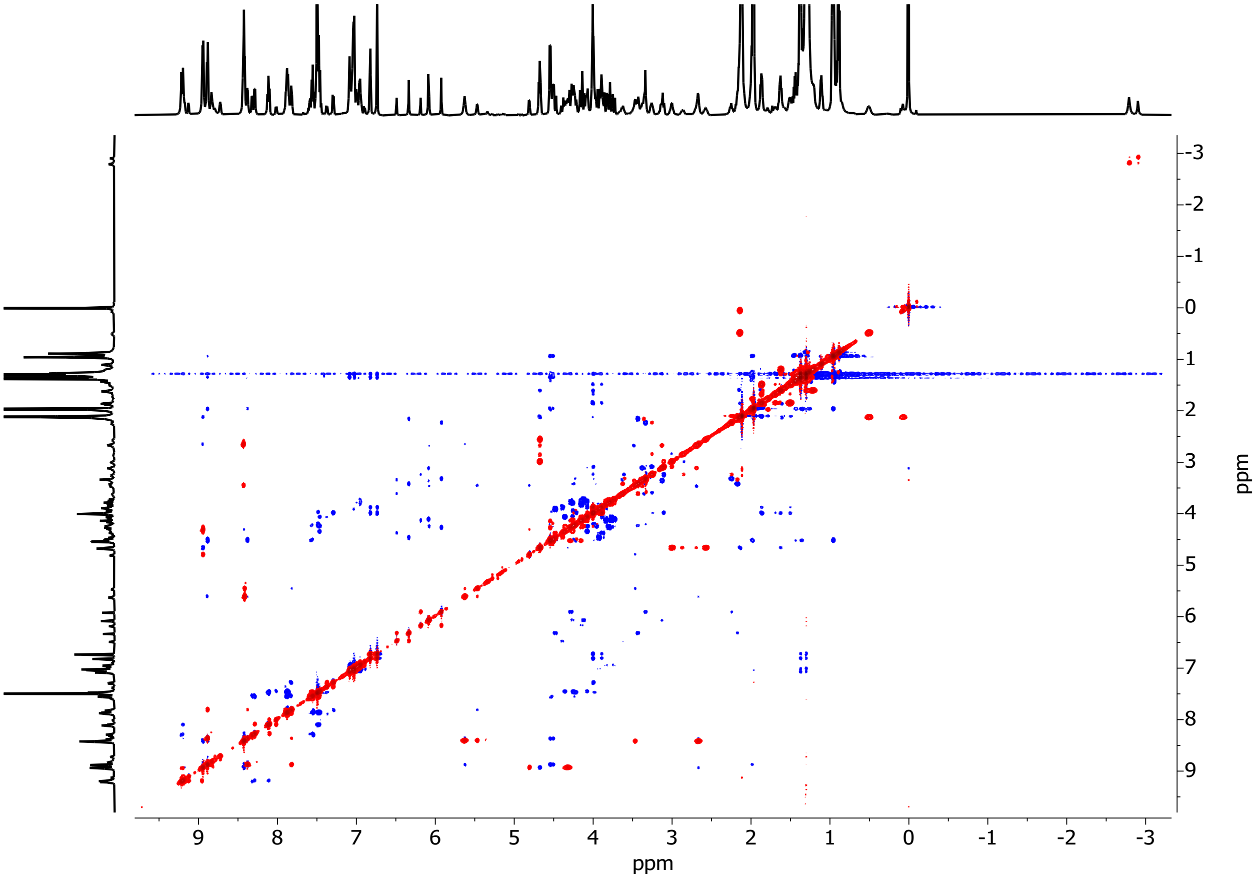

Figure SI115. Full ^{1}H-^{1}H 2D ROESY spectrum of **$H_2$2/V4** (^{1}H: 600 MHz, $CDCl_3$:CD_3CN, 1:1, v/v, host : guest, 1:3, 2 mM : 6 mM, T = 298 K). Exchange cross peaks generally have the same color as the diagonal, which is in this case blue.

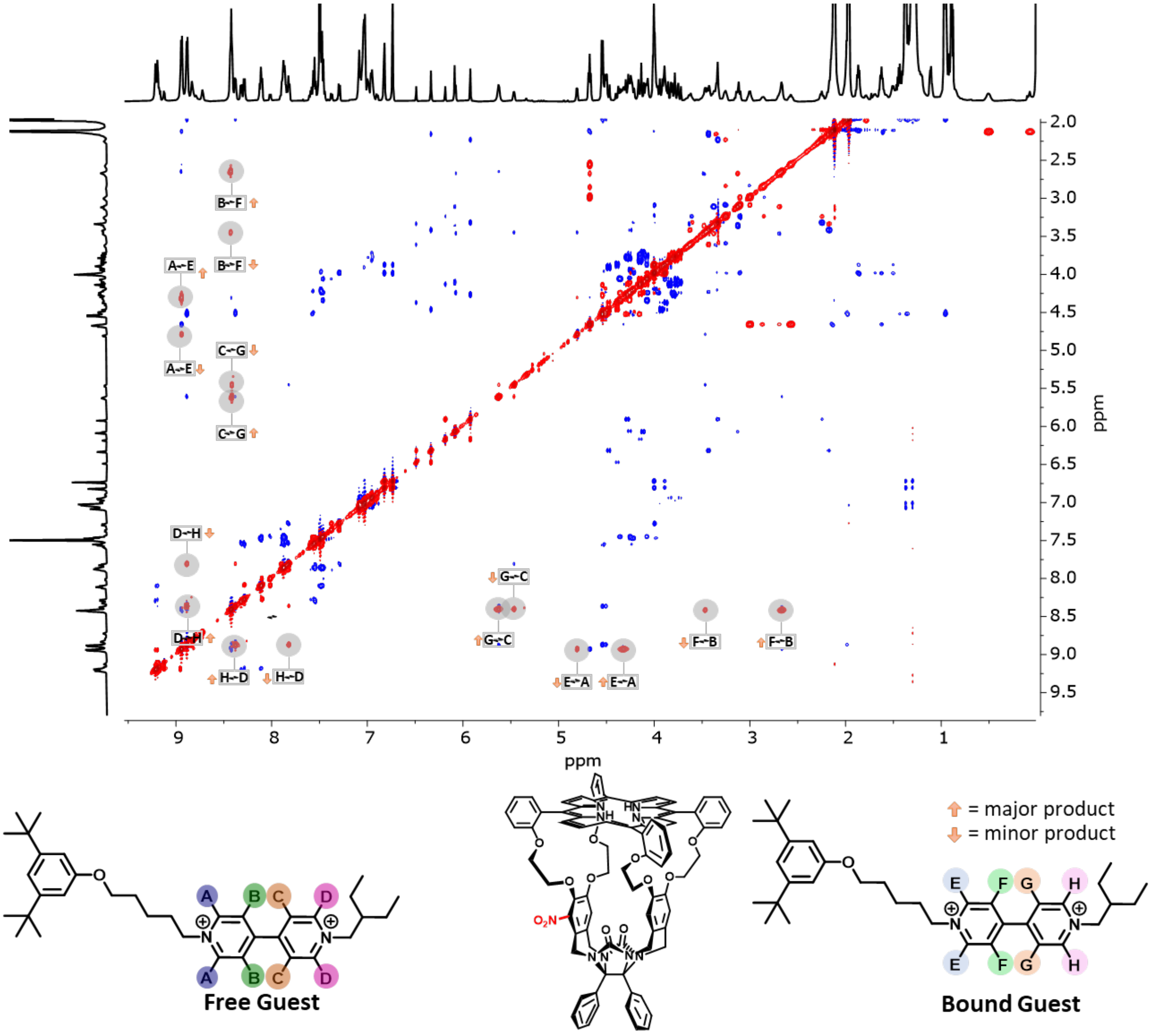

Figure SI116. Zoomed region of ^1H-^1H 2D ROESY spectrum of **H₂2/V4** (^1H: 600 MHz, $CDCl_3$:CD_3CN, 1:1, v/v, host : guest, 1:3, 2 mM : 6 mM, T = 243 K) showing relevant guest exchange interactions indicated with A-H. ^1H-^1H 2D ROESY spectrum. Exchange cross peaks generally have the same color as the diagonal, which is in this case blue.

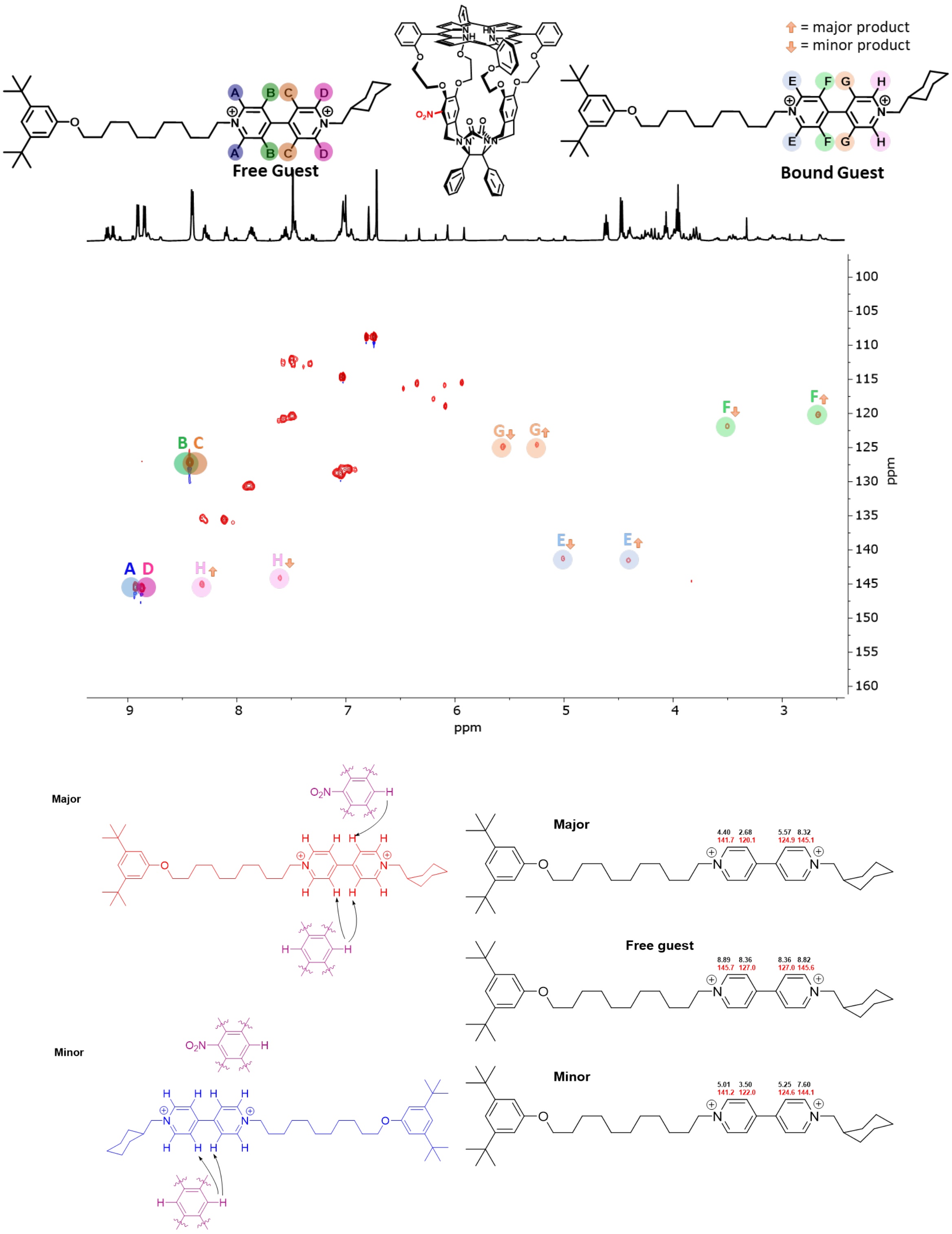

Figure SI117. Top: ^{13}C-^{1}H HSQC of **$H_2$2/V5** (^{1}H: 500 MHz, ^{13}C: 125 MHz, $CDCl_3$:CD_3CN, 1:1, v/v, host : guest, 1:3, 2 mM : 6 mM) in which the protons of the free and bound guests are shown in corresponding colors. The shifts of both major and minor abundant complexes are indicated as well; bottom: assigned shifts in ppm for the free and bound guests with the corresponding ROE interactions that could be measured by 2D ROESY.

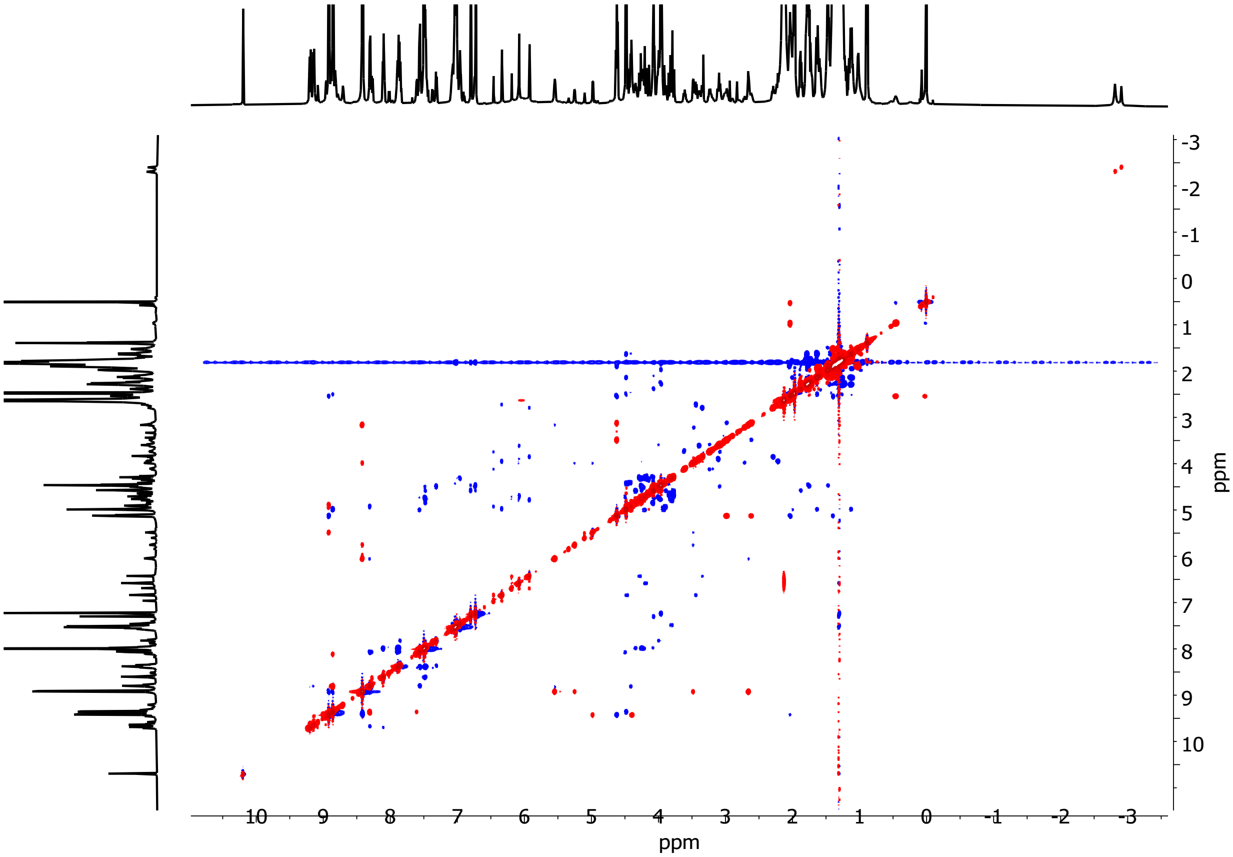

Figure SI118. Full ^{1}H-^{1}H 2D ROESY spectrum of **H$_2$2/V5** (^{1}H: 500 MHz, $CDCl_3$:CD_3CN, 1:1, v/v, host : guest, 1:3, 2 mM : 6 mM, T = 298 K). Exchange cross peaks generally have the same color as the diagonal, which is in this case blue.

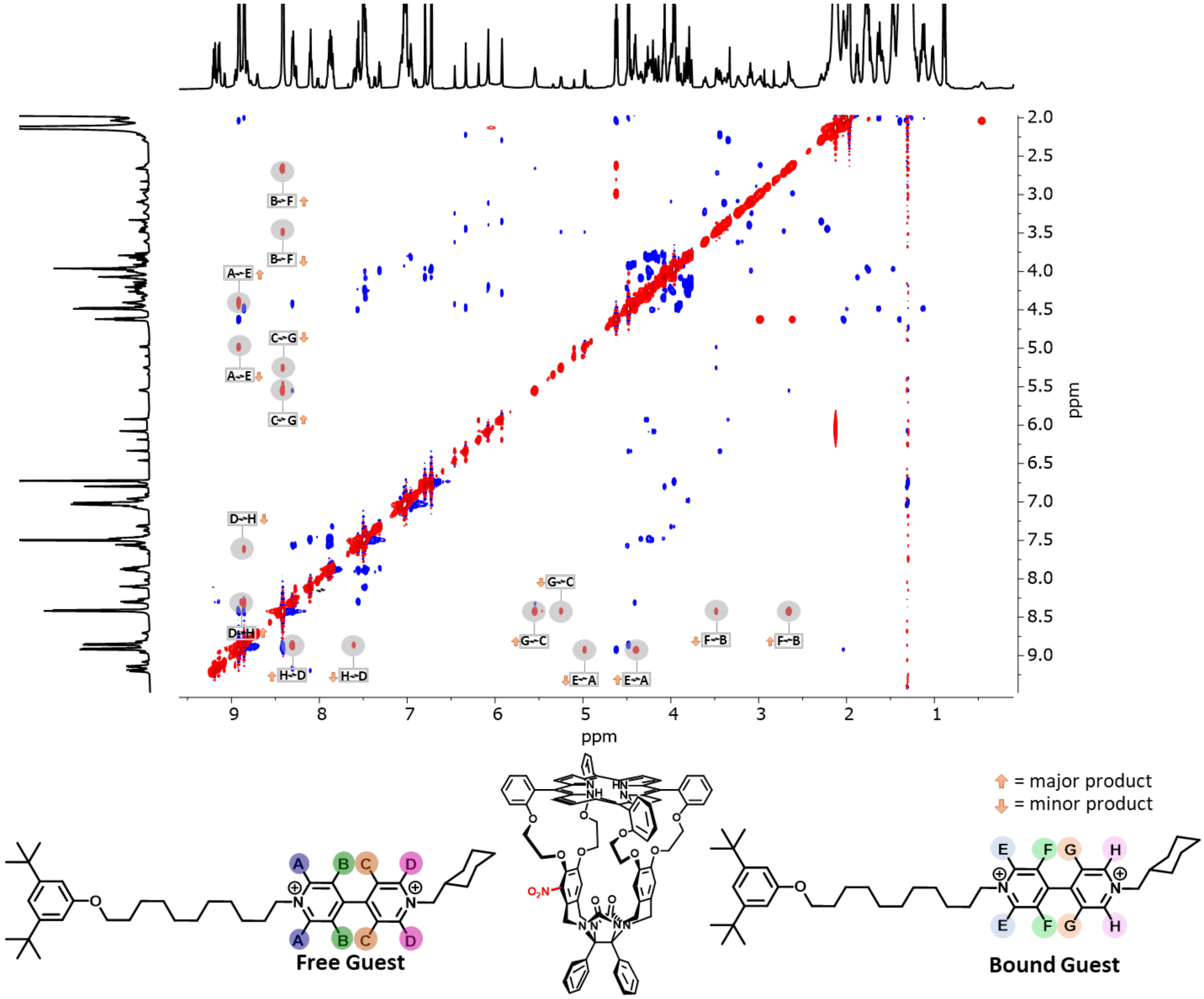

Figure SI119. Zoomed region of ^{1}H-^{1}H 2D ROESY spectrum of **$H_2$2/V5** (^{1}H: 600 MHz, $CDCl_3$:CD_3CN, 1:1, v/v, host : guest, 1:3, 2 mM : 6 mM, T = 243 K) showing relevant guest exchange interactions indicated with A-H. ^{1}H-^{1}H 2D ROESY spectrum. Exchange cross peaks generally have the same color as the diagonal, which is in this case blue.

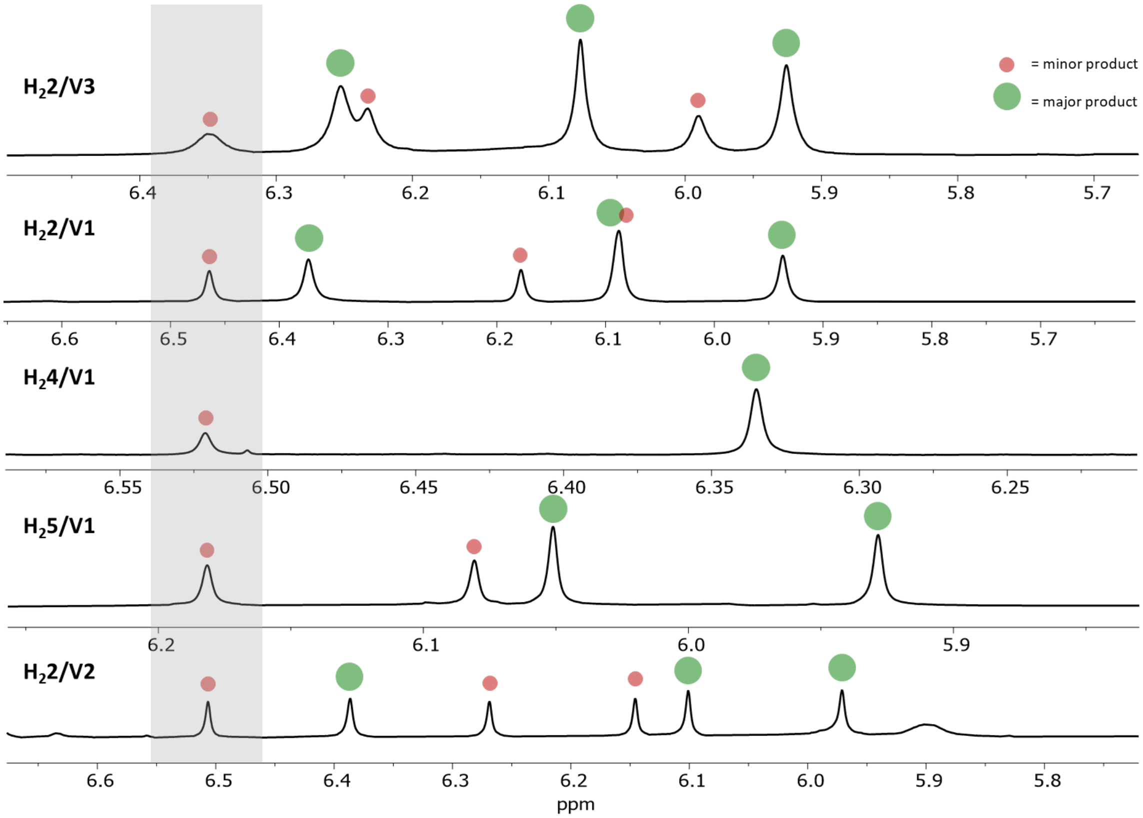

Figure SI120. Stacked ^{1}H NMR spectra (500 MHz, $CDCl_3$:CD_3CN, 1:1, v/v, host : guest, 1:3, 2 mM : 6 mM) of the different host/guest complexes, in which the shifts of the xylylene sidewall protons are shown. In all host/guest combinations the minor abundant complex shows a proton signal that is most downfield compared to the other visible proton signals of the sidewalls. This trend in all spectra indicates that the guest orientations in the major and minor abundant complexes in **$H_2$2/V3** are the same as in the other host-guest combinations, for which the orientations could be determined by 2D ROESY.

Evidence for directional versus rotational or positional exchange

We can confidently state that the different species we observe result from directional exchange rather than rotational exchange because of the number of signals that we observe in the ^{1}H NMR spectrum. If there was rotational isomerism, we would expect 8 4,4'-bipyridine ^{1}H signals for each species (major abundant complex and minor abundant complex) that integrate in a ratio of 1:1 to the xylylene sidewall-protons. Instead, we observe 4 signals that integrate 2 to 1 to the sidewall protons, which is inconsistent with rotational isomerism (see Figure SI121).

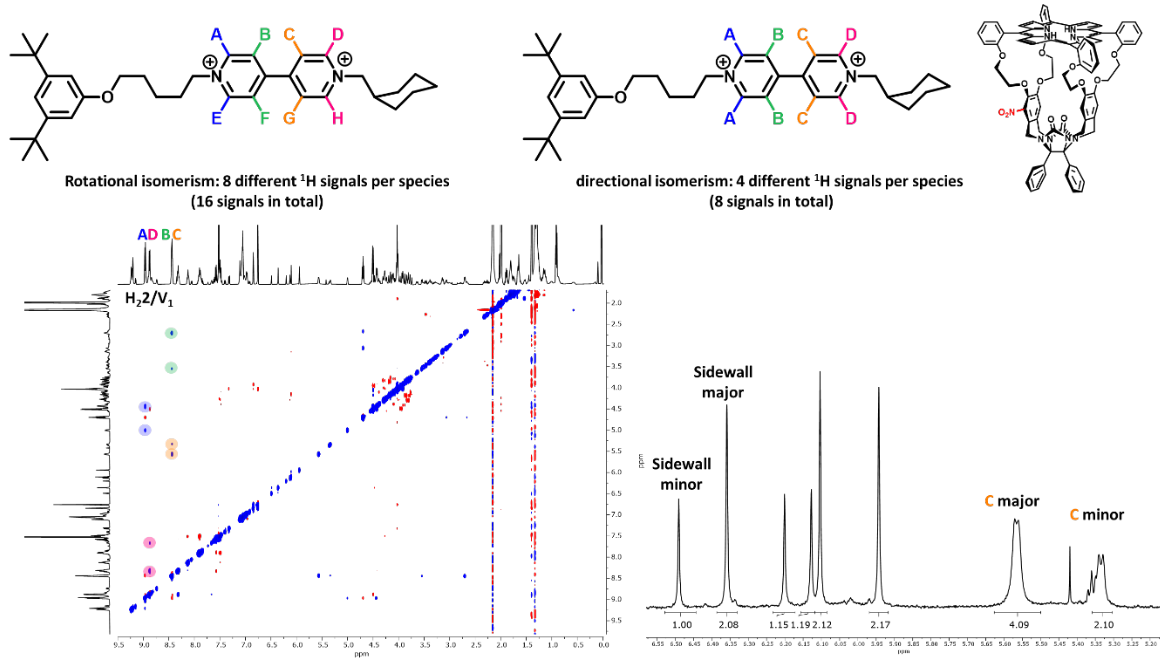

Figure SI121. Top: the hypothetical structure of **V1** if rotational isomerism was present and the structure of **V1** if only directional isomerism occurs. Bottom left: ^{1}H-^{1}H 2D ROESY spectrum of **$H_2$2/V1** (^{1}H: 500 MHz, $CDCl_3$:CD_3CN, 1:1, v/v, host : guest, 1:3, 2 mM : 6 mM, T = 243 K) in which 8 exchange peaks are visible from the guest compound; bottom right: ^{1}H NMR spectrum of $H_2$2/V1 (^{1}H: 500 MHz, $CDCl_3$:CD_3CN, 1:1, v/v, host : guest, 1:3, 2 mM : 6 mM, T = 243 K) in which the xylylene sidewall protons are indicated and integrate in a 1:2 ratio to the bound viologen signals as expected for directional isomerism.

Furthermore, for positional isomerism to occur a deeply bound guest and a weakly bound guest should be observed (Figure SI122). We see that this is not the case since the ROE data shows the same set of side walls interacting with either the head group or the linker, which does not support a system with positional isomerism (see Figure SI104). Furthermore, if the guest is weakly bound, then the shielding effects of the cavity greatly diminish. Therefore, 3 sets of downfield peaks and 1 set of upfield peaks would be expected in the presence of positional isomerism. Instead, we observe the opposite: 3 sets of upfield (6 ppm – 2 ppm) ^{1}H signals for the 4,4'-bipyridine and 1 set which is more downfield (~8 ppm) (see Table SI11).

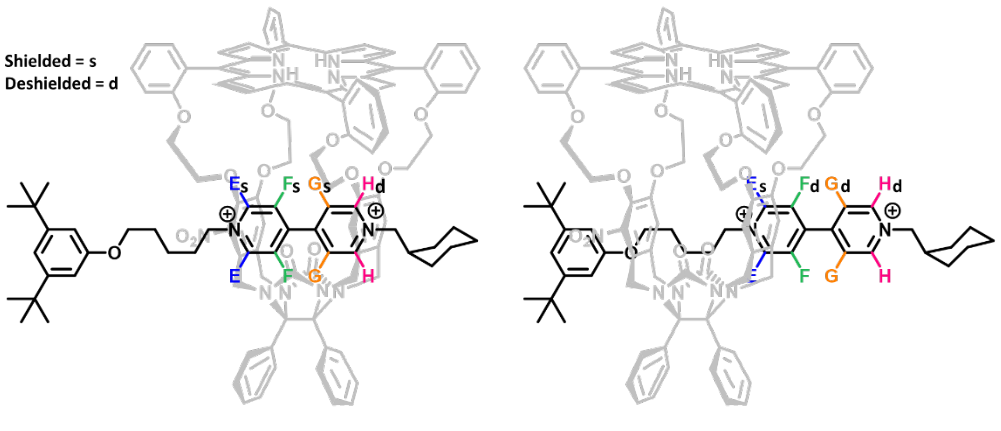

Figure SI122. Left: structure of the deeply bound guest (**V1**) in **H₂2** where the different 4,4'-bipyridyl protons are indicated with s (shielded) and d (deshielded), right: structure of the weakly bound guest creating a positional isomer where the different 4,4'-bipyridyl protons are indicated with s (shielded) and d (deshielded).

References

[1] S. F. M. van Dongen, S. Cantekin, J. A. A. W. Elemans, A. E. Rowan, R. J. M. Nolte, *Chem. Soc. Rev.* **2013**, *43*, 99–122.

[2] C. P. Leroy, *2,2-Dimethyl-{11 -Aryloxy-Alkanoic Acids and Salts and Esters Thereof*, **n.d.**, DE1925423 (A1).

[3] R. G. E. Coumans, J. A. A. W. Elemans, A. E. Rowan, R. J. M. Nolte, *Chem. – Eur. J.* **2013**, *19*, 7758–7770.

[4] P. J. Gilissen, A. Swartjes, B. Spierenburg, J. P. J. Bruekers, P. Tinnemans, P. B. White, F. P. J. T. Rutjes, R. J. M. Nolte, J. A. A. W. Elemans, *Tetrahedron* **2019**, *75*, 4640–4647.

[5] S. Varghese, B. Spierenburg, A. Swartjes, P. B. White, P. Tinnemans, J. A. A. W. Elemans, R. J. M. Nolte, *Org. Lett.* **2018**, *20*, 3719–3722.

[6] A. Cooksy, *Physical Chemistry*, Pearson Education, **2013**.

[7] M. H. Haindl, J. Hioe, R. M. Gschwind, *J. Am. Chem. Soc.* **2015**, *137*, 12835–12842.

[8] "Supramolecular.org - Binding Constant Calculators | Supramolecular," can be found under http://supramolecular.org/, **n.d.**

[9] D. C. Harris, *Quantitative Chemical Analysis*, W. H. Freeman, **2010**.
